# Therapeutic effects of *Euphorbia Pekinensis* and *Glycyrrhiza glabra* on Hepatocellular Carcinoma Ascites Partially Via Regulating the Frk-Arhgdib-Inpp5d-Avpr2-Aqp4 Signal Axis

**DOI:** 10.1038/srep41925

**Published:** 2017-02-06

**Authors:** Yanqiong Zhang, Chen Yan, Yuting Li, Xia Mao, Weiwei Tao, Yuping Tang, Ya Lin, Qiuyan Guo, Jingao Duan, Na Lin

**Affiliations:** 1 Institute of Chinese Materia Medica, China Academy of Chinese Medical Sciences, Beijing 100700, China; 2 Guangzhou University of Chinese Medicine, Guangzhou 510006, China; 3 Jiangsu Collaborative Innovation Center of Chinese Medicinal Resources Industrialization, National and Local Collaborative Engineering Center of Chinese Medicinal Resources Industrialization and Formulae Innovative Medicine; Nanjing University of Chinese Medicine, Nanjing 210023, China; 4 College of Pharmacy, Fujian University of Traditional Chinese Medicine, Fuzhou 350122, China

## Abstract

To clarify unknown rationalities of herbaceous compatibility of *Euphorbia Pekinensis* (DJ) and *Glycyrrhiza glabra* (GC) acting on hepatocellular carcinoma (HCC) ascites, peritoneum transcriptomics profiling of 15 subjects, including normal control (Con), HCC ascites mouse model (Mod), DJ-alone, DJ/GC-synergy and DJ/GC-antagonism treatment groups were performed on OneArray platform, followed by differentially expressed genes (DEGs) screening. DEGs between Mod and Con groups were considered as HCC ascites-related genes, and those among different drug treatment and Mod groups were identified as DJ/GC-combination-related genes. Then, an interaction network of HCC ascites-related gene-DJ/GC combination-related gene-known therapeutic target gene for ascites was constructed. Based on nodes’ degree, closeness, betweenness and k-coreness, the Frk-Arhgdib-Inpp5d-Avpr2-Aqp4 axis with highly network topological importance was demonstrated to be a candidate target of DJ/GC combination acting on HCC ascites. Importantly, both qPCR and western blot analyses verified this regulatory effects based on HCC ascites mice *in vivo* and M-1 collecting duct cells *in vitro*. Collectively, different combination designs of DJ and GC may lead to synergistic or antagonistic effects on HCC ascites partially via regulating the Frk-Arhgdib-Inpp5d-Avpr2-Aqp4 axis, implying that global gene expression profiling combined with network analysis can offer an effective way to understand pharmacological mechanisms of traditional Chinese medicine prescriptions.

Malignant ascites represents one of the most frequent complications at the end stage of a variety of human cancers, such as hepatocellular carcinoma (HCC)^1^. Patients with advanced HCC frequently suffer from malignant ascites developed from the invasion of HCC to the peritoneum^2^. Approximately half of patients with this complications have clinical syndromes of ascites at the initial stage of their diagnosis with cancers^3^. The onset and aggravation of malignant ascites may lead to abnormally abdominal distension, peritoneal effusion and oppression on the thoracic cavity, which may seriously deteriorate patients’ quality of life and may be usually a poor prognostic sign^4^. Clinically, various therapeutic strategies, such as simple ascites drainage and diuretic medicine for symptomatic relief, and chemotherapy and surgical management for treating associated malignancies, have been used to relieve this complication^5^. However, no satisfactory efficiency of these therapeutic modalities has been achieved, due to their short efficacy, limited response rates, as well as inevitable adverse effects such as intestinal obstruction, sepsis and infection^6^. Therefore, more effective and safe therapeutic modalities are urgently demanded to alleviate patients with malignant hepatic ascites and prolong their survival.

Traditional Chinese medicine (TCM) is an essential component of comprehensive medicinal system with abundant clinical practice. TCM aims to reverse the imbalance of the whole body and has received increasing popularity due to its definite therapeutic effects and low toxicity^7^. Notably, several Chinese herbs and TCM formulae have a wide application in malignant ascites therapy. *Euphorbia Pekinensis* (DJ), the radix of *Euphorbia pekinensis Rup*., is a well-known Chinese herb specialized in diuresis and swelling reduction. DJ has been extensively used in the treatment of oedema, malignant ascites and urinary retention for several centuries. It is also contained in many prescriptions for malignant ascites therapy, such as Ji Dai formula and modified Shi Zao decoction^8^,^9^. Extracts of DJ have been revealed to exhibit numerous bioactivities including anti-virus and anti-inflammatory effects^10^. Another Chinese herb, *Glycyrrhiza glabra* (Licorice, Gancao, GC), the root of *Glycyrrhiza uralensis Fisch*. or *Glycyrrhiza glabra L*., *Leguminosae*, has been considered as “the king of medicine” with various bioactivities and great medical values based on clinical trials for a long time. Functionally, GC can guide other herbs to the corresponding meridians and harmonize the properties of different medicines in a TCM formula^11^. Its active ingredients possess anti-inflammatory, anti-angiogenesis and anti-cancer pharmacological activities. Among them, glabridin, liquiritigenin and Glycyrrhiza polysaccharide have been proved to induce tumor cell death in HCC cells^11^,^12^,^13^. According to the allegation of “eighteen antagonistic medicaments” in Chinese medicinal literature, DJ and GC should not be used together due to their antagonistic actions. Nevertheless, growing experimental and clinical evidence show that DJ and GC may exhibit synergistic or antagonistic actions in different combination designs^14^,^15^. In the current study, we aimed to clarify the rationalities of the herbaceous compatibility of DJ and GC acting on HCC ascites, which have not been fully elucidated.

Chinese herbs, as multi-component and multi-target drugs, can exert therapeutic effectiveness through modulating the whole molecular network of the body system by using their active components^16^. As great advancement of complex network theory and technologies in recent years, the network pharmacology-based approaches have been considered as useful tools to explain and evaluate the rationality and compatibility of Chinese herbs or prescriptions at a system level. The emerging methodologies of this research field are considered with the purpose of finding multiple nodes which can be influenced by herb combinations for interfering with robust disease phenotypes and fewer adverse effects^17^. In our recent studies, the mechanisms of action and scientific evidence of several herb pairs and herbal formulae have been explored based on TCM theory and practice, the available databases and computational tools in TCM network pharmacology^18^,^19^,^20^,^21^,^22^,^23^,^24^,^25^. More recently, we have integrated drug target prediction, network analysis and experimental validation to find that another herbal pair of *Euphorbia kansui* (GS) and GC may exert synergistic or antagonistic effects on HCC ascites under different combination designs, partially through regulating the Adrb1-Pik3cg-Avpr2 signal axis^21^, highlighting the efficiency of network-based approaches to the pharmacological research of herb pairs. In addition, global gene expression profiling constitutes an important landmark for discovering novel disease-related genes and therapeutic targets for drugs. Therefore, we here initially performed peritoneum transcriptomics profiling in 15 subjects (3 per group, 5 groups), including normal control (Con), HCC ascites mouse model (Mod), DJ-alone, DJ/GC-synergy and DJ/GC-antagonism treatment groups on the OneArray microarray platform, followed by differentially expressed genes (DEGs) screening using the R package. After interaction network of DEGs was constructed by STRING, we predicted the candidate targets implicated into the combinatory effects of the herbal pair DJ and GC by calculating network topological features and performing functional enrichment analysis, followed by a series of *in vivo* and *in vitro* experimental validations (Fig. 1).

## Results and Discussion

### Identification of HCC ascites-related genes

**Figure 2:**
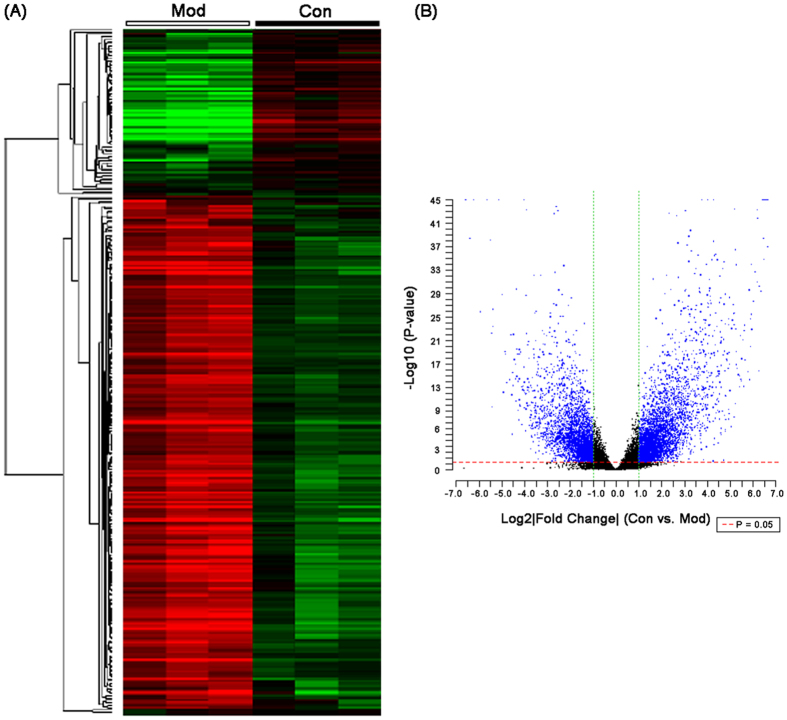
Unsupervised hierarchical clustering analysis (**A**) and volcanno plot (**B**) of all dysregulated genes in hepatocellular carcinoma ascites mice and normal control mice.

The global gene expression profile was available for 3 HCC ascites mice and 3 normal controls. After data processing and DEG screening, 2252 annotated genes were differentially expressed (>2-fold, P < 0.05), including 1418 upregulated and 834 downregulated genes in peritoneum tissues of HCC ascites mice compared to the normal mice (Supplementary Table S1). In addition, unsupervised hierarchical clustering analysis (Fig. 2A) and volcano plot (Fig. 2B) of all dysregulated genes showed a good differentiation of normal and HCC ascites samples. Pathway enrichment analysis showed that the upregulated genes in HCC ascites mice were significantly associated with Chemokine signaling pathway (Bonferroni corrected P value = 8.34E-09), Fc gamma R-mediated phagocytosis (Bonferroni corrected P value = 6.48E-06), DNA replication (Bonferroni corrected P value = 3.53E-05), Toll-like receptor signaling pathway (Bonferroni corrected P value = 9.70E-05), Cell cycle (Bonferroni corrected P value = 5.02E-04), Natural killer cell mediated cytotoxicity (Bonferroni corrected P value = 0.01), Cytokine-cytokine receptor interaction (Bonferroni corrected P value = 0.02) and B cell receptor signaling pathway (Bonferroni corrected P value = 0.03), while the downregulated genes were involved into MAPK signaling pathway (Bonferroni corrected P value = 0.007).

### Identification of DJ/GC combination-related genes

After comparing the gene expression profiles among DJ/GC-synergy, DJ/GC-antagonism, DJ-alone treatment groups with that of HCC ascites mouse model group, we identified 86 DJ/GC combination-related genes (Supplementary Table S2), including 25 genes upregulated in HCC ascites mouse model compared to normal control, but downregulated after the treatment of DJ alone; 35 genes upregulated in HCC ascites mouse model compared to normal control, but downregulated after the treatment of DJ/GC synergy combination; 4 genes upregulated in both HCC ascites mouse model and DJ/GC-antagonism compared to normal control; 10 genes downregulated in HCC ascites mouse model compared to normal control, but upregulated after the treatment of DJ alone; 26 genes downregulated in HCC ascites mouse model compared to normal control, but upregulated after the treatment of DJ/GC synergy combination; 1 gene downregulated in both HCC ascites mouse model and DJ/GC-antagonism compared to normal control.

### Identification of hub genes in the network of HCC ascites-related gene-DJ/GC combination-related gene-known therapeutic target gene for ascites

The network of HCC ascites-related gene-DJ/GC combination-related gene-known therapeutic target gene for ascites was constructed based on the links among HCC ascites-related genes, DJ/GC combination-related genes and known therapeutic targets for ascites, to shed light on the combination principles of herbal pair DJ and GC on the HCC ascites. The network consisted of 799 nodes and 6903 edges (Supplementary Table S3).

The network hubs with extremely high levels of degree often tend to encode essential genes, thus, we identified hubs according to the node degree in the network of HCC ascites-related gene-DJ/GC combination-related gene-known therapeutic target gene for ascites. As a result, there were 629 hubs, the degree values of which were more than twice the median degree of all nodes in the network. Functionally, these hub genes were significantly associated with various cancer-related and malignant ascites-related pathways, including Chemokine signaling pathway (Bonferroni corrected P value = 1.01E-09), Cell cycle (Bonferroni corrected P value = 1.79E-09), Toll-like receptor signaling pathway (Bonferroni corrected P value = 2.44E-07), Natural killer cell mediated cytotoxicity (Bonferroni corrected P value = 9.77E-07), DNA replication (Bonferroni corrected P value = 1.08E-06), Cytokine-cytokine receptor interaction (Bonferroni corrected P value = 1.31E-05), TNF signaling pathway (Bonferroni corrected P value = 5.65E-05), NF-kappa B signaling pathway (Bonferroni corrected P value = 8.73E-05), NOD-like receptor signaling pathway (Bonferroni corrected P value = 0.0001), Metabolic pathways (Bonferroni corrected P value = 0.0003), Primary immunodeficiency (Bonferroni corrected P value = 0.0005), Hepatitis B (Bonferroni corrected P value = 0.0007), MAPK signaling pathway (Bonferroni corrected P value = 0.0015), p53 signaling pathway (Bonferroni corrected P value = 0.0027), VEGF signaling pathway (Bonferroni corrected P value = 0.0034) and Complement and coagulation cascades (Bonferroni corrected P value = 0.0291).

**Figure 3:**
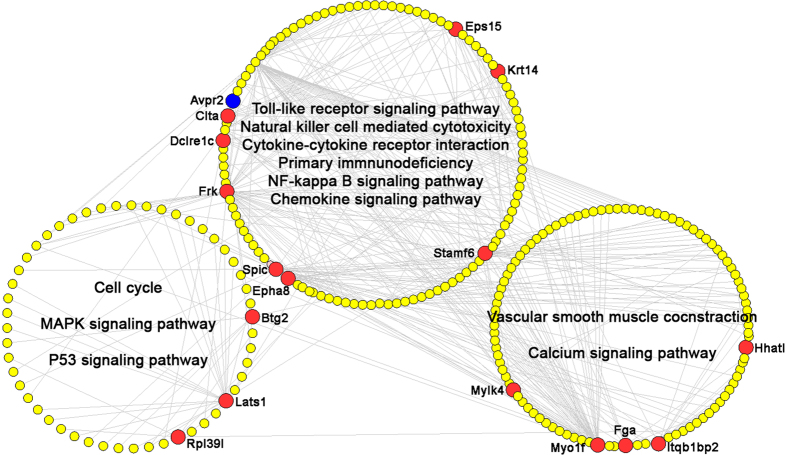
Three functional modules of hub interaction network based on a Markov clustering algorithm. Yellow nodes refer to hepatocellular carcinoma ascites related genes; Red nodes refer to DJ/GC combination-related genes; Blue nodes refer to known therapeutic target gene for ascites.

After that, we constructed the hub interaction network, which consisted of 263 nodes and 384 edges, using the direct interactions among hubs (Supplementary Table S4). It has been reported that modularity may be another important aspect of an interaction network. Nodes with highly interconnection are often implicated into the same biological modules or pathways. Using a Markov clustering algorithm, we divided the hub interaction network into 3 functional modules (Fig. 3). As the largest lymphoid organ with unique immunological properties in the body, the liver can perform efficient innate defence against intestinal microbe and toxins, facilitate a tolerance rather than immunoreactivity, and provide for apoptotic disposal of redundant lymphocytes^26^. As a primary malignancy of the liver, HCC is originated from a damaged, cirrhotic liver. Growing evidence suggest that the development and progression of HCC may be associated with an imbalanced immune system, such as changes in the number and/or function of immune cells, cytokine levels, and the expression of inhibitory receptors or their ligands^27^. Our data here demonstrated that the biggest functional module of hubs in the network of HCC ascites-related gene-DJ/GC combination-related gene-known therapeutic target gene for ascites was related to the immunological properties of the liver, since the hub genes in this module were involved into toll-like receptor signaling pathway, natural killer cell mediated cytotoxicity, cytokine-cytokine receptor interaction, primary immunodeficiency, NF-kappa B signaling pathway and chemokine signaling pathway. Notably, the other two modules were respectively associated with cancer progression (Cell cycle, MAPK signaling pathway and P53 signaling pathway) and with the formation as well as the treatment of malignant ascites (Vascular smooth muscle contraction). These findings highlight the important roles of these hub genes in reversing the dysregulated molecular network during the progression of malignant ascites.

### Combination principles of the herbal pair DJ and GC in its action on HCC ascites

After calculating four topological features, i.e., ‘Degree,’ ‘Node betweenness’, ‘Closeness’ and ‘K value’, of each node in the hub interaction network, 192 major hubs which had higher topological feature values than the corresponding medians were identified. Among them, Frk and Myolf were DJ/GC combination-related genes based on the above DEGs screening. More interestingly, Frk and Avpr2 (known therapeutic target for ascites) belonged to the same functional module (Fig. 3), implying the possible role of Frk in the treatment of ascites.

**Figure 4:**
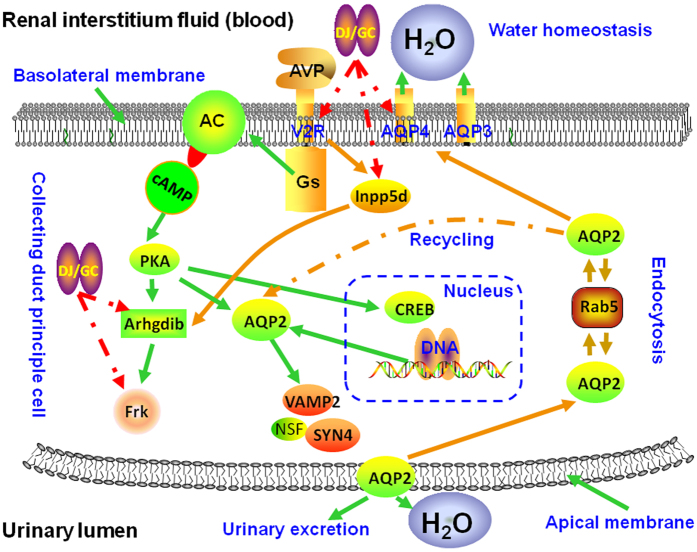
Illustration on the involvement of *Euphorbia Pekinensis* (DJ) and *Glycyrrhiza glabra* (GC) herbal pair in the pathway of vasopressin-regulated water re-absorption via regulating the Frk-Arhgdib-Inpp5d-Avpr2-Aqp4 signal axis.

Followed by the construction of Frk-Avpr2 sub-network using the links between Frk/Avpr2 and other major hubs, we found that the Frk-Arhgdib-Inpp5d-Avpr2-Aqp4 signal axis was significantly associated with the pathway of vasopressin-regulated water re-absorption (Bonferroni corrected P value = 0.005, Fig. 4), which has been indicated to play a major role in fluid retention in cirrhosis and to be a common therapeutic target pathway of various anti-ascites drugs^28^,^29^,^30^,^31^. Thus, we hypothesized that the therapeutic effect of the herb pair DJ and GC might be exerted by targeting the Frk-Arhgdib-Inpp5d-Avpr2-Aqp4 signal axis in order to regulate the process of vasopressin-mediated water re-absorption.

### Independent Experimental Validation

To validate the above hypothesis, we performed following independent experimental validations based on HCC ascites mouse model.

#### Synergistic and antagonistic effects of DJ and GC with different combinational ratios acting on HCC malignant ascites

**Figure 5:**
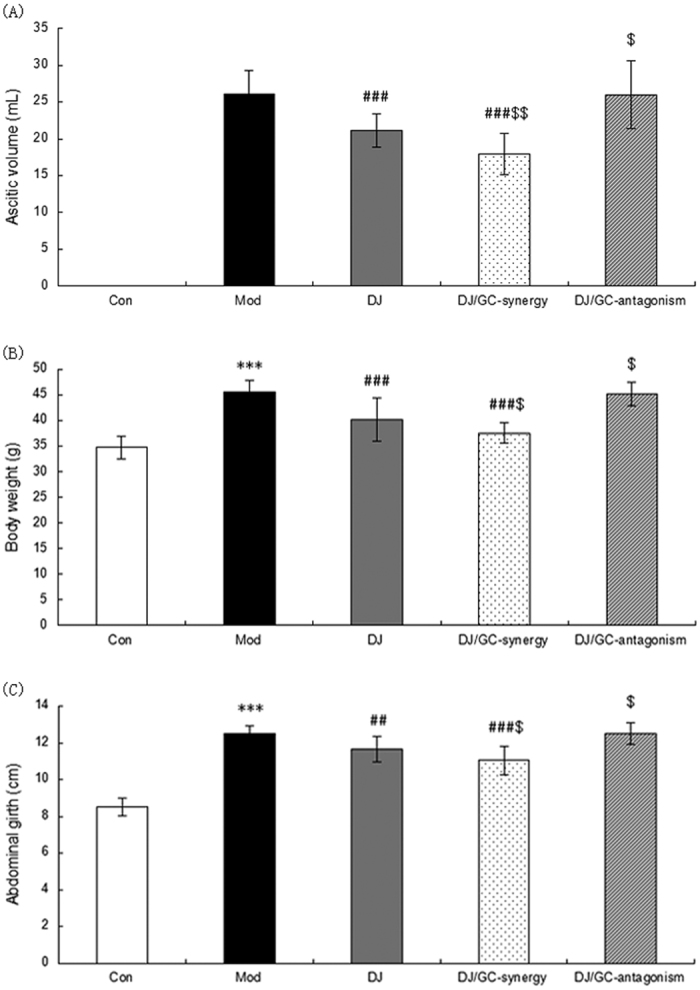
Changes in ascites volumes (**A**), body weights (**B**), and abdominal circumferences (**C**) in the H22 HCC ascites model group (n = 10), *Euphorbia Pekinensis* (DJ) alone treatment group (n = 10), DJ/*Glycyrrhiza glabra* (GC)_synergy group (DJ/GC_synergy, n = 10) and DJ/GC_antagonism group (DJ/GC_antagonism, n = 10). The data are represented as the means ± the S.E. ‘*’^,^‘**’ and ‘***’ P < 0.05, 0.01 and 0.001 compared with the normal control group, respectively; ‘^#^’^,^‘^##^’ and ‘^###^’ P < 0.05, 0.01 and 0.001 compared with the model group, respectively; ‘^$^’^,^‘^$$^’ and ‘^$$$^’ P < 0.05, 0.01 and 0.001 compared with the DJ-alone group, respectively.

Compared to normal mice, the ascites volumes (Fig. 5A), body weights (Fig. 5B) and abdominal circumferences (Fig. 5C) were significantly increased in HCC ascites mice (all P < 0.001), which was all markedly reversed by the treatment of DJ alone and DJ/GC synergy combination (all P < 0.01). This reduction trend was more obvious in DJ/GC synergy group. However, no differences with statistical significance were observed between DJ/GC antagonism and model groups (P > 0.05). Compared to DJ-alone treatment group, DJ/GC synergy combination could enhance the therapeutic effects of DJ on the ascites volumes (Fig. 5A, P < 0.01), body weights (Fig. 5B, P < 0.05) and abdominal circumferences (Fig. 5C, P < 0.05), while DJ/GC antagonism combination aggravated the malignant phenotypes of HCC ascites mice (all P < 0.05).

#### Toxicities of DJ and GC with different combinational ratios to the liver and kidney tissues of mice with HCC malignant ascites

**Figure 6:**
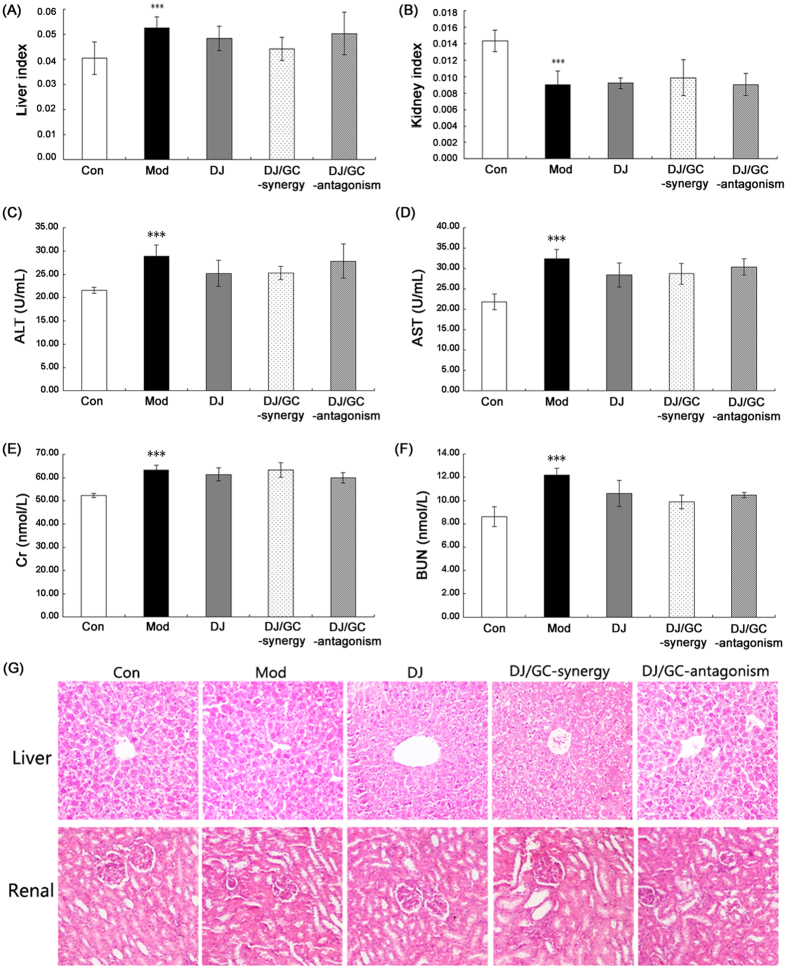
Evaluation of the liver and kidney toxicities in the H22 HCC ascites model group (n = 10), *Euphorbia Pekinensis* (DJ) alone treatment group (n = 10), DJ/*Glycyrrhiza glabra* (GC)_synergy group (DJ/GC_synergy, n = 10) and DJ/GC_antagonism group (DJ/GC_antagonism, n = 10). (**A**) Liver index in different groups; (**B**) Kidney index in different groups; (**C**–**F**) Serum levels of AST, ALT, Cr and BUN, respectively, in different groups; The data are represented as the means ± the S.E. ‘*’^,^‘**’ and ‘***’ P < 0.05, 0.01 and 0.001 compared with the normal control group, respectively; ‘^#^’^,^‘^##^’ and ‘^###^’ P < 0.05, 0.01 and 0.001 compared with the model group, respectively; ‘^$^’^,^‘^$$^’ and ‘^$$$^’ P < 0.05, 0.01 and 0.001 compared with the DJ-alone group, respectively. (**G**) Evaluation of the liver and kidney sections stained with Hematoxylin-eosin (H&E) in different groups (H&E staining, 200 × magnification).

According to Chinese medicinal literature, the herbal pair DJ and GC belongs to “eighteen incompatible medicaments” as mentioned above. Thus, it is necessary to evaluate the toxicity of DJ/GC combinations to liver and kidney tissues. As shown in Fig. 6A, the liver index in HCC ascites mice were significant higher than that in normal mice (P < 0.001), which were reversed by the treatment of both DJ and DJ/GC synergy combination, although without significant differences. In addition, Fig. 6B showed that the kidney index in HCC ascites mice were obviously reduced when compared with normal mice (P < 0.001). Both DJ and DJ/GC synergy combination had a tendency to increase the kidney index in HCC ascites. Moreover, the serum levels of ALT, AST, Cr and BUN were measured to evaluate the changes of hepatic and kidney functions in different groups. As shown in Fig. 6C–F, the serum levels of ALT, AST, Cr and BUN in HCC ascites mice were all markedly increased compared to normal mice (all P < 0.001). However, the administrations of DJ alone and DJ/GC combinations could not influence the changes of ALT, AST, Cr and BUN levels in sera of HCC ascites mice in their response to DJ alone or any combination designs of DJ and GC in comparison with model groups (all P > 0.05). Moreover, histopathological examination was performed to confirm the evidence from biochemical analyses. Figure 6G showed the normal hepatic structure of normal mice characterized with normal lobular architecture and radiating hepatic cords. Severe hepatocytes swelling, diffused and enlarged vacant spaces in cells were observed in HCC ascites mice. Regarding to the kidney tissues, abnormal alterations were shown in the glomerulus and renal tubes in HCC ascites mice. Notably, the treatment of DJ and DJ/GC combinations could ameliorate the damage of HCC malignant ascites to the liver and kidney tissues.

#### DJ/GC combination attenuates HCC ascites partially via regulating the Frk-Arhgdib-Inpp5d-Avpr2-Aqp4 signal axis

Frk (Fyn-related kinase), a member of the Src non-receptor tyrosine kinase family, acts as a versatile signal transduction molecule that involves in tissue- or cell-type specific tumor progression^32^. It is predominantly expressed in epithelial tissues including liver and kidney. *In vitro* and *in vivo* studies have revealed the inhibitory effects of Frk on the growth, invasion and colony formation of various human cancer types, including HCC^33^. Chen *et al*.^34^ found that the expression level of FRK in HCC tissue samples were markedly increased, and enforced expression of FRK promoted cell invasion of Hep3B and HepG2 cell lines, implying that it might act as a positive regulator of invasiveness in liver cancer cells. Arhgdib (Rho GDP dissociation inhibitor beta, RhoGDI2), as a Rho guanosine diphosphate dissociation inhibitor, is mainly expressed in hematopoietic cells. Accumulating studies have been reported that Arhgdib may be implicated in cell migration and invasion^35^, and may play a role in the regulation of lymphocyte activation and survival, which is related to the immune function of the human bodies^36^. Interestingly, Arhgdib has been identified as a proto-oncogene that is upregulated in human cancers. Fang *et al*.^37^ reported that Arhgdib could promote HCC growth and invasion, implying its potential as a novel HCC therapeutic target candidate. Inpp5d (Src homology 2–containing 5-inositol phosphatase), a member of the inositol 5′-phosphatase family, is expressed by hematopoietic cells and acts as an important inhibitory molecule in the regulation of signals in lymphocytes and myeloid cells^38^. It plays an important role in the immune system partially through the activation effects of phosphatidylinositol 3-kinase^39^. Notably, Inpp5d expression was down-regulated in HCC tissues and exerted an inhibitory role in intracellular insulin signaling^40^. Avpr2 (receptor of arginine-vasopressin), primarily located at kidney and coupled to extracellular signal regulated kinase activation^41^, plays a role in enhancing the concentration of intracellular cyclic adenosine monophosphate, which leads to the enhancement of water permeability reversely^42^. Growing evidence show that Avpr2 antagonists, such as satavaptan and tolvaptan, can ameliorate the severity of ascites in cirrhosis^43^,^44^. Aqp4 (aquaporin 4) belongs to the family of AQPs, integral membrane proteins, and has been indicated to control cellular water flow. Gating of the vasopressin-regulated water channel by conformational changes induced by phosphorylation or protein-protein interactions is an established regulatory mechanism for AQPs^45^. Wright *et al*.^46^ found that the excretion of Aqp4 might be markedly enhanced in patients with liver cirrhosis.

**Figure 7:**
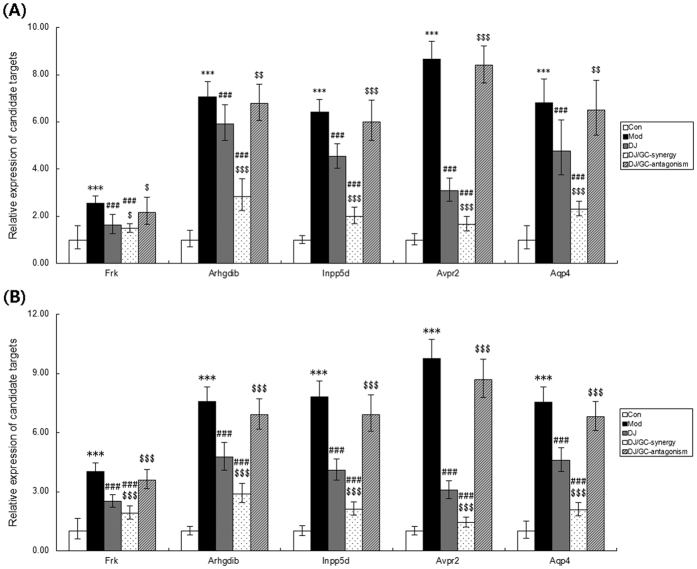
Expressions of Frk, Arhgdib, Inpp5d, Avpr2 and Aqp4 mRNA levels in the kidney tissues of the H22 HCC ascites model group (n = 10), *Euphorbia Pekinensis* (DJ) alone treatment group (n = 10), DJ/*Glycyrrhiza glabra* (GC)_synergy group (DJ/GC_synergy, n = 10) and DJ/GC_antagonism group (DJ/GC_antagonism, n = 10) as detected by qRT-PCR analysis. (**A**) The relative expression of Frk, Arhgdib, Inpp5d, Avpr2 and Aqp4 mRNA levels in different groups using GAPDH as an internal control. (**B**) The relative expression of Frk, Arhgdib, Inpp5d, Avpr2 and Aqp4 mRNA levels in different groups using RPL13A as an internal control. The data are represented as the means ± the S.E. ‘*’^,^‘**’ and ‘***’ P < 0.05, 0.01 and 0.001 compared with the normal control group, respectively; ‘^#^^,^‘^##^’ and ‘^###^’ P < 0.05, 0.01 and 0.001 compared with the model group, respectively; ‘^$^’^,^‘^$$^’ and ‘^$$$^’ P < 0.05, 0.01 and 0.001 compared with the DJ-alone group, respectively.

**Figure 8:**
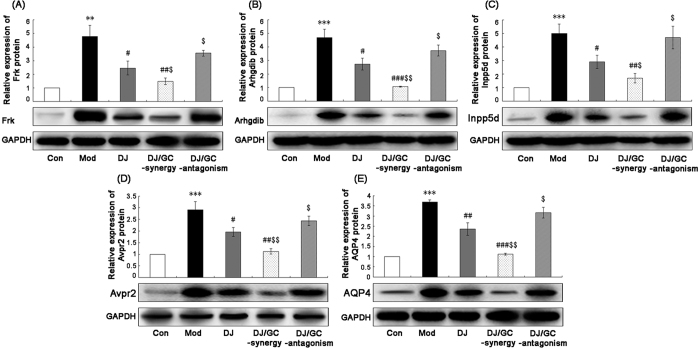
Expressions of Frk (**A**), Arhgdib (**B**), Inpp5d (**C**), Avpr2 (**D**) and Aqp4 (**E**) protein levels in the kidney tissues of the H22 HCC ascites model group (n = 10), *Euphorbia Pekinensis* (DJ) alone treatment group (n = 10), DJ/*Glycyrrhiza glabra* (GC)_synergy group (DJ/GC_synergy, n = 10) and DJ/GC_antagonism group (DJ/GC_antagonism, n = 10) as detected by western blot analysis. The data are represented as the means ± the S.E. ‘*’^,^‘**’ and ‘***’ P < 0.05, 0.01 and 0.001 compared with the normal control group, respectively; ‘^#^’^,^‘^##^’ and ‘^###^’ P < 0.05, 0.01 and 0.001 compared with the model group, respectively; ‘^$^’^,^‘^$$^’ and ‘^$$$^’ P < 0.05, 0.01 and 0.001 compared with the DJ-alone group, respectively. Lanes 1–5 in the western blot denote the normal control, HCC ascites model, DJ, DJ/GC_synergy and DJ/GC_antagonism groups, respectively.

In the current study, our data from quantitative PCR analysis based on two internal reference genes (Fig. 7) found that the expression levels of Frk, Arhgdib, Inpp5d, Avpr2 and Aqp4 mRNAs in kidney tissues of HCC ascites mice were all significantly higher than those in normal controls (all P < 0.001). After the administrations of DJ and DJ/GC-synergy combination, their expression was effectively reduced. In contrast, the treatment of DJ/GC-antagonism combination did not exert this regulatory effects. These findings were consistent with the results of Western blot analysis at protein levels (Fig. 8).

**Figure 9:**
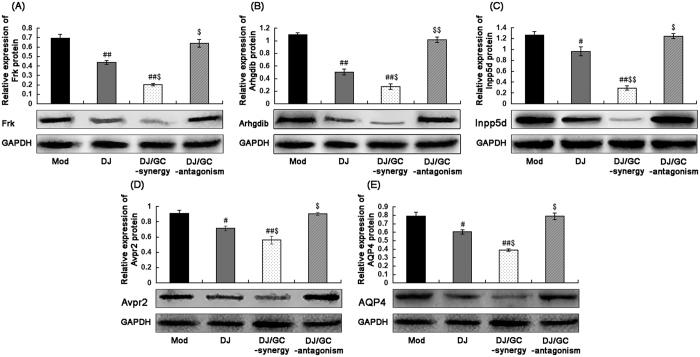
Expressions of Frk (**A**), Arhgdib (**B**), Inpp5d (**C**), Avpr2 (**D**) and Aqp4 (**E**) protein levels in the M-1 collecting duct cell (transgenic for SV40 early region) model group, *Euphorbia Pekinensis* (DJ) alone treatment group, DJ/*Glycyrrhiza glabra* (GC)_synergy group (DJ/GC_synergy) and DJ/GC_antagonism group (DJ/GC_antagonism) as detected by western blot analysis. The data are represented as the means ± the S.E. ‘^#^’^,^‘^##^’ and ‘^###^’ P < 0.05, 0.01 and 0.001 compared with the model group, respectively; ‘^$^’^,^‘^$$^’ and ‘^$$$^’ P < 0.05, 0.01 and 0.001 compared with the DJ-alone group, respectively. Lanes 1–4 in the western blot denote model, DJ, DJ/GC_synergy and DJ/GC_antagonism groups, respectively.

Similarly, Frk, Arhgdib, Inpp5d, Avpr2 and Aqp4 protein expression in M-1 collecting duct cells (transgenic for SV40 early region) *in vitro* were all significantly decreased following the treatment of DJ and DJ/GC-synergy combination (all P < 0.05, Fig. 9), while had no distinct changes in DJ/GC-antagonism group (all P > 0.05, Fig. 9).

In conclusion, our data provide a convincing evidence that the different combination designs of DJ and GC may lead to synergistic or antagonistic effects on HCC ascites partially via regulating the Frk-Arhgdib-Inpp5d-Avpr2-Aqp4 signal axis, implying that global gene expression profiling combined with network analysis and experimental validations can offer an effective way to understand the pharmacological mechanisms of TCM prescriptions.

## Materials and Methods

### Ethics statement

The study was approved by the Research Ethics Committee of Institute of Chinese Materia Medica, China Academy of Chinese Medical Sciences, Beijing, China. All animal procedures have been performed in accordance with the guidelines for the use and care of animals of the Center for Laboratory Animal Care, China Academy of Chinese Medical Sciences.

### Cells

The murine H22 HCC ascitic cell line and M-1 collecting duct cell line were both obtained from the China Infrastructure of Cell Line Resource (Beijing, China). The H22 cells were cultured in RPMI-1640 medium (Gibco, Grand Island, NY, USA), supplemented with 10% FCS, 2 mM L-glutamine, 100 IU/mL penicillin and 100 μg/mL streptomycin at 37 °C in 5% CO_2_. The M-1 cells were cultured in a 1:1 mixture of Dulbecco’s modified Eagle’s medium (Gibco, Grand Island, NY, USA) and Ham’s F12 medium (Gibco, Grand Island, NY, USA) with 2.5 mM Lglutamine adjusted to contain 15 mM HEPES, 0.5 mM sodium pyruvate and 1.2 g/L sodium bicarbonate supplemented with 0.005 mM dexamethasone and 5% fetal bovine serum at 37 °C in 5% CO_2_.

### Animals

Male Kunming mice (4–6 weeks of age and 18–22 g of weight) were purchased from Charles River Laboratories (production license No: SCXK 2012-0001, MA, USA). The mice were maintained under specific-pathogen-free conditions with a constant temperature of 24 ± 1 °C (mean ± SEM) and with a 12-hour light/dark cycle, and were allowed ad libitum access to pellet food and water.

### Construction of H22 HCC ascites mouse model

To construct the H22 HCC ascites mouse model, H22 cells were intraperitoneally inoculated into mice according to the protocol identical to previously published material^21^. Please see details in Supplementary File S1-section 1.

### Drug preparation

Following herbs DJ (Lot: 110522) and GC (Lot: 120713) were purchased from Nanjing university of Chinese medicine, Nanjing, China, and authenticated by Professor Jinao Duan based on the qualitative and quantitative standards recorded in Chinese Pharmacopoeia 2010 edition. Major chemical components of DJ and GC detected by Ultra-performance liquid chromatography tandem mass-spectrometry (UPLC-MS/MS) were listed in Supplementary Table S5. Taking DJ at a weight of 11.58 g and DJ/GC were blended at a weight ratio 1:0.5, 1:1, 1:2, 1:4, 1:0 and 0:1 separately. The mixtures were soaked with eight times of water (g/v) at 25 °C for 30 min, followed by the decoction with ten times of distilled water for 2 h. Then, the residues were extracted and decocted again with the same volume of water for 1 h. The two supernatants were combined and distillated to a designated volume of 600 mL, obtaining liquid medicines at different concentrations (0.0289, 0.0386, 0.0579, 0.0965, 0.0193, 0.0772 g/mL), and kept at 4 °C prior to use.

### Screening DJ/GC-synergy and DJ/GC-antagonism combinations acting on HCC ascites

In this study, 120 male Kunming mice were divided into eight groups equally (15 per group): normal control (Con), H22 HCC ascites model (Mod), DJ/GC combination (DJ/GC = 1:0.5, 1:1, 1:2, 1:4), the DJ alone treatment (DJ-alone) and GC alone treatment (GC-alone) groups. Weight, abdominal circumferences and ascites volume were measured for screening DJ/GC-synergy and DJ/GC-antagonism combinations which exerted the most obvious synergistic and antagonistic effects in the treatment of HCC malignant ascites. As shown in Supplementary Figure S1, DJ and GC combination, with 1/2 and 1/4 ratio (DJ/GC) had the most obvious synergistic and antagonistic effects, respectively. Thus, we selected the two combination ratios as DJ/GC-synergy and DJ/GC-antagonism groups, respectively.

### Gene expression profiles

Peritoneum tissues were collected from mice in five groups (Con, Mod, DJ-alone, DJ/GC-synergy and DJ/GC-antagonism groups), immersed in Trizol (Invitrogen, CA, USA) and frozen in liquid nitrogen immediately for further microarray detection. Fluorescent RNA targets were prepared from total RNA samples that were pooled from peritoneum tissues using Amino Allyl MessageAmp TM II aRNA Amplification Kit (AM1753, Life Technologies, USA) and Cy5 dyes (Amersham Pharmacia, Piscataway, NJ, USA). Then, the fluorescent aRNA targets were hybridized to the Mouse OneArrayR v2 (Phalanx Biotech Group, Taiwan; containing 27295 DNA oligonucleotide probes), scanned with an Agilent’s High-Resolution C Scanner (Agilent Technologies, CA, USA) and finally analyzed by GenePix 4.1 software (Molecular Devices). The spots with log2 ratio ≥1 or log2 ratio ≤−1 and P-value < 0.05 are tested for further analysis. The gene expression microarray data of GSE76817 (http://www.ncbi.nlm.nih.gov/geo/query/acc.cgi?acc=GSE76817) were obtained from the National Center of Biotechnology Information (NCBI) Gene Expression Omnibus (GEO, http://www.ncbi.nlm.nih.gov/geo/).

### DEG screening

Significant DEGs of Con vs. Mod groups, Mod vs. DJ-alone treatment groups, Mod vs. DJ/GC-synergy treatment groups, Mod vs. DJ/GC-antagonism treatment groups, DJ-alone vs. DJ/GC-synergy treatment groups and DJ-alone vs. DJ/GC-antagonism treatment groups were identified using the criteria of P value < 0.05 and |log2 fold change (FC)| ≥ 1. The hierarchical clustering analysis was performed for the identified DEGs according to the heat map package in R (version 1.0.2, R Core Team, Vienna, Austria). Cluster analysis of the DEGs was performed by Cluster 3.0, based on Euclidean distance.

### Network construction and analysis

The interaction network of HCC ascites-related gene-DJ/GC combination-related gene-known therapeutic target gene for ascites was constructed based on the public database STRING (Search Tool for Known and Predicted Protein-Protein Interactions, version 10.0, http://string-db.org/)^47^ with the median value of combined scores of all interactions as a threshold. Then, Navigator software (Version 2.2.1) was applied to visualize the interaction network.

Four topological features, node’s degree, betweenness, closeness and k-coreness, were calculated to evaluate the importance of a node in the interaction network. The definitions of these network topological features are described in Supplementary File S1-section 2.

### Pathway enrichment analysis

Pathway enrichment analysis was performed using pathway data obtained from the FTP service of KEGG^48^ (http://www.genome.jp/kegg/, Last updated: Oct 16, 2012).

### Liver and kidney indexes

Liver and kidney tissues were dissected from different groups, and were then blotted dry with filter papers and weighted using the electronic balance (Satorius, Goettingen, Germany). The liver and kidney indexes were calculated as the following formula: Liver/kidney indexes = [Liver/kidney weight (g)/body weight (g)] × 100%

### Serum biochemical analysis

Concentrations of liver and kidney injury markers, including alanine aminotransferase (ALT, for liver), aspartate transaminase (AST, for liver), urine creatinine (Cr, for kidney) and blood urea nitrogen (BUN, for kidney), were analyzed using standardized kinetic and fixed-rate colorimetric procedures under common hospital laboratory equipment. All experiments were done in triplicate using fresh serum.

### Histopathological observation

To evaluate the histopathological changes of liver and kidney tissues in different groups, the paraformaldehyde fixed tissues were embedded in paraffin and sectioned into a thickness of 4 μm. Hematoxylin and eosin (H&E) staining was performed and the stained areas were viewed under an optical microscope (Carl Zeiss AG, Jena, Germany). All sections were assessed by two experienced observers who were blinded to the background of this study.

### Quantitative PCR

Gene expression levels of candidate targets in kidney tissues obtained from different groups were detected by quantitative PCR analysis. SYBR Green 1 kit was applied in accordance with the manufacturer’s protocol (Roche, Mannheim, Germany). In brief, total RNA was extracted from peritoneal membrane using the TRIzol Reagent (Invitrogen, CA, USA). Then, reverse transcription of RNA to cDNA was achieved by pooling 2 μg of total RNA using ABI High-Capacity cDNA Kits (Applied Biosystems, CA, USA). The RT products were diluted 10-fold and 10 μL was used for PCR analysis using a CFX Connect real-time system (Bio-Rad Laboratories, Hercules, CA, USA). House-keeping genes glyceraldehyde-3-phosphate dehydrogenase (GAPDH) and ribosomal protein L13a (RPL13A) were used as reference genes. The primer sequences using in this study were listed in Supplementary Table S6. Each sample was tested in triplicate. For data analysis, the 2^−ΔΔCt^ method was used for qualifying the relative expression levels of candidate genes^49^.

### Western blot analysis

The western blot protocol was performed in accordance with our previous studies^50^,^51^. The following antibodies were used: SHIP antibody (rabbit monoclonal antibody, dilution 1:1000, Abcam, Cambridge, UK), FRK antibody (rabbit polyclonal antibody, dilution 1:1000, Abcam, Cambridge, UK), Aquaporin 4 antibody (rabbit polyclonal antibody, dilution 1:1000, Abcam, Cambridge, UK), AVPR V2 antibody (rabbit polyclonal antibody, dilution 1:1000, Abcam, Cambridge, UK) and D4 GDI antibody (rabbit monoclonal antibody, dilution 1:1000, Abcam, Cambridge, UK). All experiments were done in triplicate. The mean normalized protein expressions ± SEs were calculated from independent experiments.

### Statistical analysis

All statistical analyses were performed by the SPSS software version 11.0 (SPSS Inc., Chicago, IL, USA) for Windows. Our data were expressed as means ± standard deviation. One-Way ANOVA followed by LSD test was used for the analysis of all parameters. P value less than 0.05 was considered to be statistically significant.

## Additional Information

**How to cite this article**: Zhang, Y. *et al*. Therapeutic effects of *Euphorbia Pekinensis* and *Glycyrrhiza glabra* on Hepatocellular Carcinoma Ascites Partially Via Regulating the Frk-Arhgdib-Inpp5d-Avpr2-Aqp4 Signal Axis. *Sci. Rep.* **7**, 41925; doi: 10.1038/srep41925 (2017).

**Publisher's note:** Springer Nature remains neutral with regard to jurisdictional claims in published maps and institutional affiliations.

## Supplementary Material

Supplementary Files

## Figures and Tables

**Figure 1:**
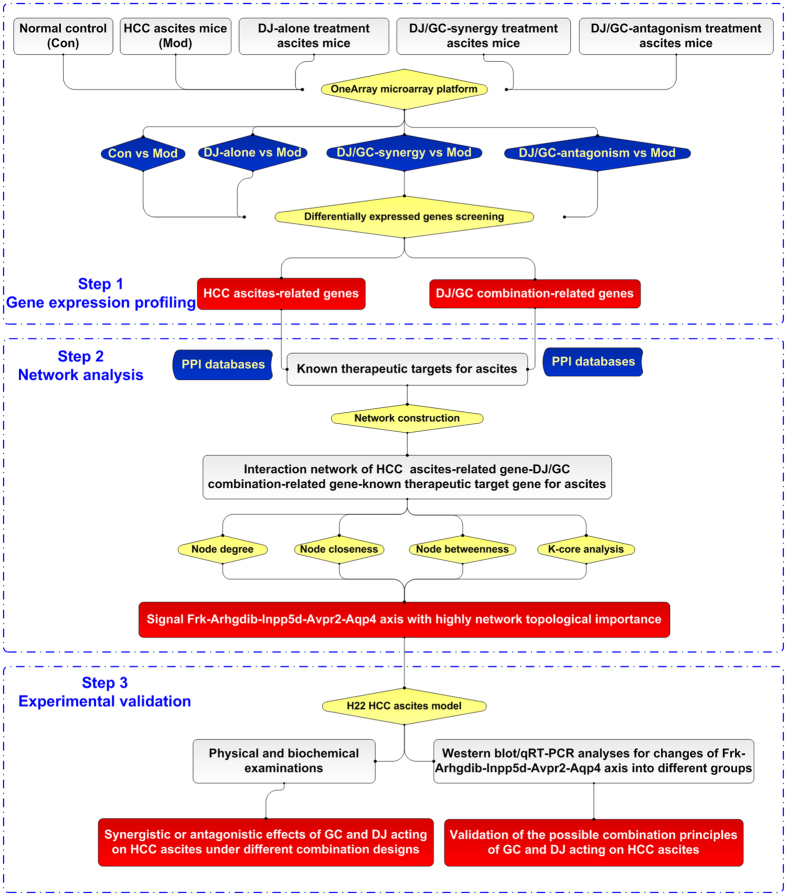
A schematic diagram for clarifying the rationalities of the herbaceous compatibility of *Euphorbia Pekinensis* (DJ) and *Glycyrrhiza glabra* (GC) herbal pair acting on hepatocellular carcinoma ascites via the integration of transcriptional regulatory network and experimental validation.
