# Supplementary material for: Therapeutic effects of Euphorbia Pekinensis and Glycyrrhiza glabra on Hepatocellular Carcinoma Ascites Partially Via Regulating the Frk-Arhgdib-Inpp5d-Avpr2-Aqp4 Signal Axis
Source: Sci Rep. 2017 Feb 6;7:41925. doi: 10.1038/srep41925 (PMC5292954; doi:10.1038/srep41925)
Supplement: Supplementary Files [file srep41925-s1.pdf]

Supplementary Files for "Therapeutic effects of Euphorbia Pekinensis and Glycyrrhiza glabra on Hepatocellular Carcinoma Ascites Partially Via Regulating the Frk-Arhgdib-Inpp5d-Avpr2-Aqp4 Signal Axis"

Authors: Yanqiong ZHANG, Chen YAN, Yuting LI, Xia MAO, Weiwei TAO, Yuping TANG, Ya LIN, Qiuyan GUO, Jingao DUAN, Na LIN

File S1:

Section 1 Construction of H22 HCC ascites mouse model

The experimental protocol was approved by Medical Experimental Animal Care Committee of Institute of Chinese Materia Medica, China Academy of Chinese Medical Sciences.

Cells

The murine H22 HCC ascitic cell line was obtained from the Institute of Biochemistry and Cell Biology of the Chinese Academy of Sciences (Shanghai, China). The cells were cultured in RPMI-1640 medium (Gibco, Grand Island, NY, USA), supplemented with 10% FCS, 2 mM L-glutamine, 100 IU/mL penicillin and 100 μg/mL streptomycin at 37°C in 5% CO_2.

Animals

Male Kunming mice (4-6 weeks of age and 18-22g of weight) were purchased from Charles River Laboratories (production license No: SCXK 2012-0001, MA, USA). The mice were maintained under specific-pathogen-free conditions with a constant temperature of 24±1°C (mean±SEM) and with a 12-hour light/dark cycle, and were allowed ad libitum access to pellet food and water.

Model construction

To construct the H22 HCC ascites mouse model, the needle was inserted into the left lower abdomen, and H22 cells were inoculated intraperitoneally. Each mouse was inoculated with 1×10^{7} H22 cells. The procedure was not associated with mortality or morbidity.

Section 2 Defining network topological feature set

For each node i in interaction network, we defined four measures for assessing its topological property: (1) 'Degree' is defined as the number of links to node i; (2) 'Node betweenness' is defined as the number of shortest paths between pairs of nodes that run through node i. (3) ‘Closeness’ is defined as the inverse of the farness which is the sum of node i distances to all other nodes. The Closeness centrality can be regarded as a measure of how long it will take to spread information from node i to all other nodes sequentially. Degree, node, betweenness and closeness centralities can measure a node’s topological importance in the network. The larger a node’s degree/ node betweenness /closeness centrality is, the more important the node is in the interaction network [1]. (4) K-core analysis is an iterative process in which the nodes are removed from the networks in order of least-connected [2]. The core of maximum order is defined as the main core or the highest k-core of the network. A k-core sub-network of the original network can be generated by recursively deleting vertices from the network whose degree is less than k. This results in a series of sub-networks that gradually reveal the globally central region of the original network. On this basis,

'K value' is used to measure the centrality of node i.

References:

[1]. Wang Y, Liu Z, Li C, Li D, Ouyang Y, Yu J, Guo S, He F, Wang W. Drug target prediction based on the herbs components: the study on the multitargets pharmacological mechanism of qishenkeli acting on the coronary heart disease. *Evid. Based Complement Alternat. Med.* 2012 (2012) 698531.

[2]. Wuchty S, Almaas E. Evolutionary cores of domain co-occurrence networks. *BMC Evol. Biol*. 5 (2005) 24.

Figure S1

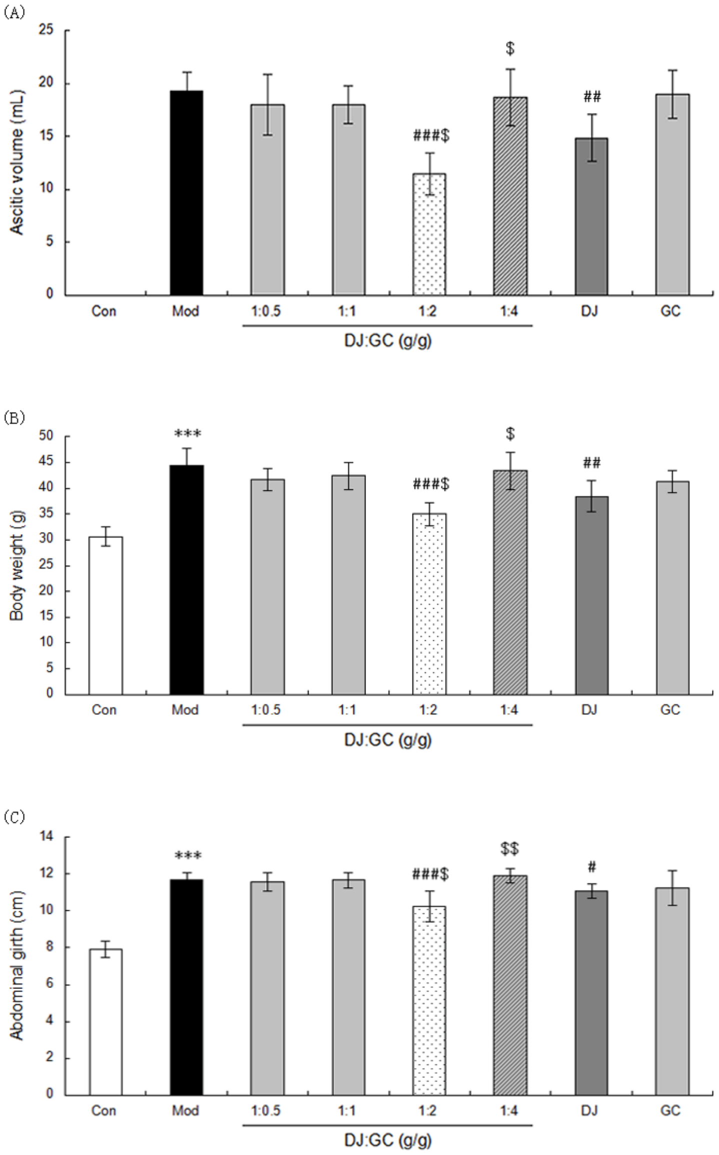

Figure S1 Changes in ascites volumes (A), body weights (B), and abdominal circumferences (C) in normal control (Con, n=15), H22 HCC ascites model (Mod, n=15), DJ/GC combination (DJ/GC=1:0.5, 1:1, 1:2, 1:4, n=15), the DJ alone treatment (DJ-alone, n=15) and GC alone treatment (GC-alone, n=15) groups. The data are represented as the means ± the S.E. '***' P<0.001 compared with Con group, '#', '##' and '###' P<0.05, 0.01 and 0.001 compared with Mod group; '$', '$$' and '$$$' P<0.05, 0.01 and 0.001 compared with DJ-alone group.

Table S1 Dysregulated genes in peritoneum tissues of hepatocellular carcinoma ascites mice compared to the normal mice

Gene symbol	Gene name	Expression pattern (Mod vs. Con)
Dbp	D site albumin promoter binding protein	Downregulated
Krt14	keratin 14	Downregulated
BC089597	cDNA sequence BC089597	Downregulated
Sgca	sarcoglycan, alpha (dystrophin-associated glycoprotein)	Downregulated
Abcb4	ATP-binding cassette, sub-family B (MDR/TAP), member 4	Downregulated
Cdh15	cadherin 15	Downregulated
Hspa1l	heat shock protein 1-like	Downregulated
Pla2g4e	phospholipase A2, group IVE	Downregulated
Calcr	calcitonin receptor	Downregulated
Tecta	tectorin alpha	Downregulated
Pcsk6	proprotein convertase subtilisin/kexin type 6	Downregulated
Stac3	SH3 and cysteine rich domain 3	Downregulated
Neu2	neuraminidase 2	Downregulated
Murc	muscle-related coiled-coil protein	Downregulated
AI464131	expressed sequence AI464131	Downregulated
Asb14	ankyrin repeat and SOCS box-containing 14	Downregulated
Rimkla	ribosomal modification protein rimK-like family member A	Downregulated
Emid2	EMI domain containing 2	Downregulated
Dusp28	dual specificity phosphatase 28	Downregulated
Mfsd3	major facilitator superfamily domain containing 3	Downregulated
Dnajc12	DnaJ (Hsp40) homolog, subfamily C, member 12	Downregulated
2210011C24Rik	RIKEN cDNA 2210011C24 gene	Downregulated
Plk5	polo-like kinase 5	Downregulated
Pygm	muscle glycogen phosphorylase	Downregulated
Zmynd10	zinc finger, MYND domain containing 10	Downregulated
Wipf3	WAS/WASL interacting protein family, member 3	Downregulated
Dnajb5	DnaJ (Hsp40) homolog, subfamily B, member 5	Downregulated
Mgam	maltase-glucoamylase	Downregulated
Ucp3	uncoupling protein 3 (mitochondrial, proton carrier)	Downregulated
Slc16a3	solute carrier family 16 (monocarboxylic acid transporters), member 3	Downregulated
Ky	kyphoscoliosis peptidase	Downregulated
Mylk2	myosin, light polypeptide kinase 2, skeletal muscle	Downregulated
0610040B10Rik	RIKEN cDNA 0610040B10 gene	Downregulated
Msc	musculin	Downregulated
Hfe2	hemochromatosis type 2 (juvenile) (human homolog)	Downregulated
Kcna7	potassium voltage-gated channel, shaker-related subfamily, member 7	Downregulated
Grtp1	GH regulated TBC protein 1	Downregulated
Rpl3l	ribosomal protein L3-like	Downregulated
Svs3b	seminal vesicle secretory protein 3B	Downregulated
Mypn	myopalladin	Downregulated
Zic1	zinc finger protein of the cerebellum 1	Downregulated
Ptges3l	prostaglandin E synthase 3 (cytosolic)-like	Downregulated
Mutyh	mutY homolog (E. coli)	Downregulated
Ociad2	OCIA domain containing 2	Downregulated
Synb	syncytin b	Downregulated
Camk2a	calcium/calmodulin-dependent protein kinase II alpha	Downregulated
Plekhb1	pleckstrin homology domain containing, family B (evectins) member 1	Downregulated
Polr3gl	polymerase (RNA) III (DNA directed) polypeptide G like	Downregulated
Plekhh1	pleckstrin homology domain containing, family H (with MyTH4 domain) member 1	Downregulated
Trim69	tripartite motif-containing 69	Downregulated
2310034G01Rik	RIKEN cDNA 2310034G01 gene	Downregulated
Egf	epidermal growth factor	Downregulated
Smtnl2	smoothelin-like 2	Downregulated
Slc2a4	solute carrier family 2 (facilitated glucose transporter), member 4	Downregulated
Sdr39u1	short chain dehydrogenase/reductase family 39U, member 1	Downregulated
Vat1l	vesicle amine transport protein 1 homolog-like (T. californica)	Downregulated
Asb10	ankyrin repeat and SOCS box-containing 10	Downregulated
Gal	galanin	Downregulated
Myom1	myomesin 1	Downregulated
5830454E08Rik	RIKEN cDNA 5830454E08 gene	Downregulated
Ppp1r3c	protein phosphatase 1, regulatory (inhibitor) subunit 3C	Downregulated
Myadml2	myeloid-associated differentiation marker-like 2	Downregulated
Fam162b	family with sequence similarity 162, member B	Downregulated
Hoxc5	homeobox C5	Downregulated
Oxld1	oxidoreductase like domain containing 1	Downregulated
Myom2	myomesin 2	Downregulated
Mbp	myelin basic protein	Downregulated
Fhit	fragile histidine triad gene	Downregulated
Wfikkn2	WAP, follistatin/kazal, immunoglobulin, kunitz and netrin domain containing 2	Downregulated
Angptl6	angiopoietin-like 6	Downregulated
Prkag3	protein kinase, AMP-activated, gamma 3 non-catatlytic subunit	Downregulated
Bcas3	breast carcinoma amplified sequence 3	Downregulated
A930018P22Rik	RIKEN cDNA A930018P22 gene	Downregulated
Klhl31	kelch-like 31 (Drosophila)	Downregulated
Igsf1	immunoglobulin superfamily, member 1	Downregulated
Ctsf	cathepsin F	Downregulated
Dmpk	dystrophia myotonica-protein kinase	Downregulated
Casq2	calsequestrin 2	Downregulated
Sh3rf2	SH3 domain containing ring finger 2	Downregulated
Chst4	carbohydrate (chondroitin 6/keratan) sulfotransferase 4	Downregulated
Fyco1	FYVE and coiled-coil domain containing 1	Downregulated
Megf10	multiple EGF-like-domains 10	Downregulated
2310042D19Rik	RIKEN cDNA 2310042D19 gene	Downregulated
Sirt3	sirtuin 3 (silent mating type information regulation 2, homolog) 3 (S. cerevisiae)	Downregulated
Ddit4l	DNA-damage-inducible transcript 4-like	Downregulated
Pifo	primary cilia formation	Downregulated
Uts2r	urotensin 2 receptor	Downregulated
Tectb	tectorin beta	Downregulated
Ldoc1l	leucine zipper, down-regulated in cancer 1-like	Downregulated
Yipf7	Yip1 domain family, member 7	Downregulated
Lrrc20	leucine rich repeat containing 20	Downregulated
Iffo1	intermediate filament family orphan 1	Downregulated
Wbscr17	Williams-Beuren syndrome chromosome region 17 homolog (human)	Downregulated
Ace	angiotensin I converting enzyme (peptidyl-dipeptidase A) 1	Downregulated
Lrrc38	leucine rich repeat containing 38	Downregulated
Trp63	transformation related protein 63	Downregulated

Rnf123	ring finger protein 123	Downregulated
Hist2h3c2	histone cluster 2, H3c2	Downregulated
Cd59a	CD59a antigen	Downregulated
Myod1	myogenic differentiation 1	Downregulated
2900005J15Rik	RIKEN cDNA 2900005J15 gene	Downregulated
Tob1	transducer of ErbB-2.1	Downregulated
Emc9	ER membrane protein complex subunit 9	Downregulated
Creld1	cysteine-rich with EGF-like domains 1	Downregulated
Pacrg	PARK2 co-regulated	Downregulated
1810013D10Rik	RIKEN cDNA 1810013D10 gene	Downregulated
Pitx2	paired-like homeodomain transcription factor 2	Downregulated
Amigo1	adhesion molecule with Ig like domain 1	Downregulated
9530091C08Rik	RIKEN cDNA 9530091C08 gene	Downregulated
Cmbl	carboxymethylenebutenolidase-like (Pseudomonas)	Downregulated
Gpd1	glycerol-3-phosphate dehydrogenase 1 (soluble)	Downregulated
Zfp358	zinc finger protein 358	Downregulated
Hif1an	hypoxia-inducible factor 1, alpha subunit inhibitor	Downregulated
Idh3a	isocitrate dehydrogenase 3 (NAD+) alpha	Downregulated
2310015D24Rik	RIKEN cDNA 2310015D24 gene	Downregulated
Wnt9a	wingless-type MMTV integration site 9A	Downregulated
Kcnb1	potassium voltage gated channel, Shab-related subfamily, member 1	Downregulated
Riiad1	regulatory subunit of type II PKA R-subunit (RIIa) domain containing 1	Downregulated
Myoz3	myozenin 3	Downregulated
2310007L24Rik	RIKEN cDNA 2310007L24 gene	Downregulated
Sobp	sine oculis-binding protein homolog (Drosophila)	Downregulated
Endog	endonuclease G	Downregulated
Ache	acetylcholinesterase	Downregulated
Spsb4	splA/ryanodine receptor domain and SOCS box containing 4	Downregulated
Obsl1	obscurin-like 1	Downregulated
Tspan7	tetraspanin 7	Downregulated
Zfp3	zinc finger protein 3	Downregulated
Itgb1bp2	integrin beta 1 binding protein 2	Downregulated
Pdzd3	PDZ domain containing 3	Downregulated
Atp1b2	ATPase, Na+/K+ transporting, beta 2 polypeptide	Downregulated
Atp6v0e2	ATPase, H+ transporting, lysosomal V0 subunit E2	Downregulated
Serinc4	serine incorporator 4	Downregulated
Pip	prolactin induced protein	Downregulated
6430571L13Rik	RIKEN cDNA 6430571L13 gene	Downregulated
St8sia5	ST8 alpha-N-acetyl-neuraminide alpha-2,8-sialyltransferase 5	Downregulated
Asb2	ankyrin repeat and SOCS box-containing 2	Downregulated
Cited4	Cbp/p300-interacting transactivator, with Glu/Asp-rich carboxy-terminal domain, 4	Downregulated
Myo18b	myosin XVIIIb	Downregulated
Mapk12	mitogen-activated protein kinase 12	Downregulated
5033430I15Rik	RIKEN cDNA 5033430I15 gene	Downregulated
Vegfb	vascular endothelial growth factor B	Downregulated
Wfs1	Wolfram syndrome 1 homolog (human)	Downregulated
Engase	endo-beta-N-acetylglucosaminidase	Downregulated
Cacna1s	calcium channel, voltage-dependent, L type, alpha 1S subunit	Downregulated
Abhd1	abhydrolase domain containing 1	Downregulated
Lypd6	LY6/PLAUR domain containing 6	Downregulated
Fzd9	frizzled homolog 9 (Drosophila)	Downregulated
Wfdc1	WAP four-disulfide core domain 1	Downregulated
Tas1r1	taste receptor, type 1, member 1	Downregulated
Lama2	laminin, alpha 2	Downregulated
Krtap20-2	keratin associated protein 20-2	Downregulated
Spink3	serine peptidase inhibitor, Kazal type 3	Downregulated
Ppp1r1a	protein phosphatase 1, regulatory (inhibitor) subunit 1A	Downregulated
Lynx1	Ly6/neurotoxin 1	Downregulated
P2rx5	purinergic receptor P2X, ligand-gated ion channel, 5	Downregulated
Dcun1d4	DCN1, defective in cullin neddylation 1, domain containing 4 (S. cerevisiae)	Downregulated
Tmem143	transmembrane protein 143	Downregulated
Themis3	thymocyte selection associated family member 3	Downregulated
Aif1l	allograft inflammatory factor 1-like	Downregulated
Fam53a	family with sequence similarity 53, member A	Downregulated
Kcnj12	potassium inwardly-rectifying channel, subfamily J, member 12	Downregulated
Epdr1	ependymin related protein 1 (zebrafish)	Downregulated
Kif20a	kinesin family member 20A	Downregulated
Slc25a12	solute carrier family 25 (mitochondrial carrier, Aralar), member 12	Downregulated
Phkg1	phosphorylase kinase gamma 1	Downregulated
Vgll2	vestigial like 2 homolog (Drosophila)	Downregulated
Pnp	purine-nucleoside phosphorylase	Downregulated
Cisd1	CDGSH iron sulfur domain 1	Downregulated
Extl1	exostoses (multiple)-like 1	Downregulated
Pla2g2d	phospholipase A2, group IID	Downregulated
Hhatl	hedgehog acyltransferase-like	Downregulated
Slc45a3	solute carrier family 45, member 3	Downregulated
Hoxc6	homeobox C6	Downregulated
Eepd1	endonuclease/exonuclease/phosphatase family domain containing 1	Downregulated
Prrc2b	proline-rich coiled-coil 2B	Downregulated
Myl3	myosin, light polypeptide 3	Downregulated
Pgm2	phosphoglucomutase 2	Downregulated
Myom3	myomesin family, member 3	Downregulated
Kbtbd13	kelch repeat and BTB (POZ) domain containing 13	Downregulated
Smyd1	SET and MYND domain containing 1	Downregulated
Fndc9	fibronectin type III domain containing 9	Downregulated
Flywch2	FLYWCH family member 2	Downregulated
Pxmp2	peroxisomal membrane protein 2	Downregulated
Reep1	receptor accessory protein 1	Downregulated
Nme5	NME/NM23 family member 5	Downregulated
Chrnb1	cholinergic receptor, nicotinic, beta polypeptide 1 (muscle)	Downregulated
Prss12	protease, serine, 12 neurotrypsin (motopsin)	Downregulated
Lgi3	leucine-rich repeat LGI family, member 3	Downregulated
Rsph1	radial spoke head 1 homolog (Chlamydomonas)	Downregulated
Amotl1	angiomotin-like 1	Downregulated
Aamdc	adipogenesis associated Mth938 domain containing	Downregulated
Chst10	carbohydrate sulfotransferase 10	Downregulated
Inpp5j	inositol polyphosphate 5-phosphatase J	Downregulated
Myh14	myosin, heavy polypeptide 14	Downregulated

Cdk16	cyclin-dependent kinase 16	Downregulated
Scrg1	scrapie responsive gene 1	Downregulated
Fsd2	fibronectin type III and SPRY domain containing 2	Downregulated
Sync	syncoilin	Downregulated
Hspb3	heat shock protein 3	Downregulated
Ppp1r27	protein phosphatase 1, regulatory subunit 27	Downregulated
Lmod3	leiomodin 3 (fetal)	Downregulated
Igf2	insulin-like growth factor 2	Downregulated
Cmya5	cardiomyopathy associated 5	Downregulated
Emid1	EMI domain containing 1	Downregulated
Opn1mw	opsin 1 (cone pigments), medium-wave-sensitive (color blindness, deutan)	Downregulated
Ramp1	receptor (calcitonin) activity modifying protein 1	Downregulated
1110006G14Rik	RIKEN cDNA 1110006G14 gene	Downregulated
Ttn	titin	Downregulated
Gabrr2	gamma-aminobutyric acid (GABA) C receptor, subunit rho 2	Downregulated
Dnaic1	dynein, axonemal, intermediate chain 1	Downregulated
Tmem246	transmembrane protein 246	Downregulated
Ptpru	protein tyrosine phosphatase, receptor type, U	Downregulated
Cdkn1c	cyclin-dependent kinase inhibitor 1C (P57)	Downregulated
Srl	sarcalumenin	Downregulated
Tpd52l1	tumor protein D52-like 1	Downregulated
Kcnc1	potassium voltage gated channel, Shaw-related subfamily, member 1	Downregulated
Shisa4	shisa homolog 4 (Xenopus laevis)	Downregulated
Sypl2	synaptophysin-like 2	Downregulated
Fam160a1	family with sequence similarity 160, member A1	Downregulated
Slc23a3	solute carrier family 23 (nucleobase transporters), member 3	Downregulated
Igfbp5	insulin-like growth factor binding protein 5	Downregulated
Dusp8	dual specificity phosphatase 8	Downregulated
Mettl22	methyltransferase like 22	Downregulated
Srpk3	serine/arginine-rich protein specific kinase 3	Downregulated
Lurap1	leucine rich adaptor protein 1	Downregulated
Dhrs7c	dehydrogenase/reductase (SDR family) member 7C	Downregulated
Alpk3	alpha-kinase 3	Downregulated
Olfr1320	olfactory receptor 1320	Downregulated
Prss43	protease, serine, 43	Downregulated
Krt81	keratin 81	Downregulated
Nfatc2	nuclear factor of activated T cells, cytoplasmic, calcineurin dependent 2	Downregulated
Gtdc2	glycosyltransferase-like domain containing 2	Downregulated
Bola3	bolA-like 3 (E. coli)	Downregulated
Fndc5	fibronectin type III domain containing 5	Downregulated
Rhot2	ras homolog gene family, member T2	Downregulated
A930005H10Rik	RIKEN cDNA A930005H10 gene	Downregulated
Dusp27	dual specificity phosphatase 27 (putative)	Downregulated
Lrrc24	leucine rich repeat containing 24	Downregulated
Ceacam19	carcinoembryonic antigen-related cell adhesion molecule 19	Downregulated
1110020A21Rik	RIKEN cDNA 1110020A21 gene	Downregulated
Bcorl1	BCL6 co-repressor-like 1	Downregulated
Atg9a	autophagy related 9A	Downregulated
Kif5a	kinesin family member 5A	Downregulated
Usp13	ubiquitin specific peptidase 13 (isopeptidase T-3)	Downregulated
Cish	cytokine inducible SH2-containing protein	Downregulated
C1qtnf9	C1q and tumor necrosis factor related protein 9	Downregulated
Cand2	cullin-associated and neddylation-dissociated 2 (putative)	Downregulated
Fam131a	family with sequence similarity 131, member A	Downregulated
Entpd2	ectonucleoside triphosphate diphosphohydrolase 2	Downregulated
Dexi	dexamethasone-induced transcript	Downregulated
Pfkfb4	6-phosphofructo-2-kinase/fructose-2,6-biphosphatase 4	Downregulated
Fgfbp1	fibroblast growth factor binding protein 1	Downregulated
Xpnpep2	X-prolyl aminopeptidase (aminopeptidase P) 2, membrane-bound	Downregulated
Slc25a26	solute carrier family 25 (mitochondrial carrier, phosphate carrier), member 26	Downregulated
Slc37a4	solute carrier family 37 (glucose-6-phosphate transporter), member 4	Downregulated
Slc38a3	solute carrier family 38, member 3	Downregulated
Prmt8	protein arginine N-methyltransferase 8	Downregulated
Myoc	myocilin	Downregulated
Amhr2	anti-Mullerian hormone type 2 receptor	Downregulated
Flad1	RFad1, flavin adenine dinucleotide synthetase, homolog (yeast)	Downregulated
Mylk2	myosin, light polypeptide kinase 2, skeletal muscle	Downregulated
Trp53inp2	transformation related protein 53 inducible nuclear protein 2	Downregulated
Csnka2ip	casein kinase 2, alpha prime interacting protein	Downregulated
Prss36	protease, serine, 36	Downregulated
Slc25a19	solute carrier family 25 (mitochondrial thiamine pyrophosphate carrier), member 19	Downregulated
Speg	SPEG complex locus	Downregulated
C030006K11Rik	RIKEN cDNA C030006K11 gene	Downregulated
P2rx6	purinergic receptor P2X, ligand-gated ion channel, 6	Downregulated
Ank3	ankyrin 3, epithelial	Downregulated
Plin4	perilipin 4	Downregulated
Fgf6	fibroblast growth factor 6	Downregulated
Enho	energy homeostasis associated	Downregulated
Rab3a	RAB3A, member RAS oncogene family	Downregulated
Gm4980	predicted gene 4980	Downregulated
1110008P14Rik	RIKEN cDNA 1110008P14 gene	Downregulated
Tmem42	transmembrane protein 42	Downregulated
Fhod3	formin homology 2 domain containing 3	Downregulated
Efcab12	EF-hand calcium binding domain 12	Downregulated
Sugp2	SURP and G patch domain containing 2	Downregulated
Chrm5	cholinergic receptor, muscarinic 5	Downregulated
Frzb	frizzled-related protein	Downregulated
Jph2	junctophilin 2	Downregulated
Osgin1	oxidative stress induced growth inhibitor 1	Downregulated
Stradb	STE20-related kinase adaptor beta	Downregulated
Mpp3	membrane protein, palmitoylated 3 (MAGUK p55 subfamily member 3)	Downregulated
Vamp1	vesicle-associated membrane protein 1	Downregulated
Fgf13	fibroblast growth factor 13	Downregulated
Evc	Ellis van Creveld gene syndrome	Downregulated
Idnk	idnK gluconokinase homolog (E. coli)	Downregulated
Dexi	dexamethasone-induced transcript	Downregulated
2310016G11Rik	RIKEN cDNA 2310016G11 gene	Downregulated
Alpk2	alpha-kinase 2	Downregulated

Acta2	actin, alpha 2, smooth muscle, aorta	Downregulated
Barx2	BarH-like homeobox 2	Downregulated
Pacsin3	protein kinase C and casein kinase substrate in neurons 3	Downregulated
Bves	blood vessel epicardial substance	Downregulated
Abcb9	ATP-binding cassette, sub-family B (MDR/TAP), member 9	Downregulated
Bcas1	breast carcinoma amplified sequence 1	Downregulated
Gpc1	glypican 1	Downregulated
Synm	synemin, intermediate filament protein	Downregulated
Zbtb44	zinc finger and BTB domain containing 44	Downregulated
Plb1	phospholipase B1	Downregulated
Eomes	eomesodermin homolog (Xenopus laevis)	Downregulated
Atp1a4	ATPase, Na+/K+ transporting, alpha 4 polypeptide	Downregulated
Tmem201	transmembrane protein 201	Downregulated
Pcif1	PDX1 C-terminal inhibiting factor 1	Downregulated
Mamstr	MEF2 activating motif and SAP domain containing transcriptional regulator	Downregulated
Ppm1l	protein phosphatase 1 (formerly 2C)-like	Downregulated
Pnp	purine-nucleoside phosphorylase	Downregulated
Dpf3	D4, zinc and double PHD fingers, family 3	Downregulated
Map3k12	mitogen-activated protein kinase kinase kinase 12	Downregulated
Pfkm	phosphofructokinase, muscle	Downregulated
Mlip	muscular LMNA-interacting protein	Downregulated
Synpo2l	synaptopodin 2-like	Downregulated
Grip2	glutamate receptor interacting protein 2	Downregulated
Klhl32	kelch-like 32 (Drosophila)	Downregulated
1110065P20Rik	RIKEN cDNA 1110065P20 gene	Downregulated
Tox2	TOX high mobility group box family member 2	Downregulated
Fam71a	family with sequence similarity 71, member A	Downregulated
Prr23a	proline rich 23A	Downregulated
Olfr981	olfactory receptor 981	Downregulated
Fam189a2	family with sequence similarity 189, member A2	Downregulated
Cyp4f39	cytochrome P450, family 4, subfamily f, polypeptide 39	Downregulated
Kcng2	potassium voltage-gated channel, subfamily G, member 2	Downregulated
Cplx2	complexin 2	Downregulated
Cav3	caveolin 3	Downregulated
Tnnc1	troponin C, cardiac/slow skeletal	Downregulated
Ntf3	neurotrophin 3	Downregulated
Tnni1	troponin I, skeletal, slow 1	Downregulated
Lrrc14b	leucine rich repeat containing 14B	Downregulated
P4htm	prolyl 4-hydroxylase, transmembrane (endoplasmic reticulum)	Downregulated
Ppapdc3	phosphatidic acid phosphatase type 2 domain containing 3	Downregulated
Rbm24	RNA binding motif protein 24	Downregulated
Fhl3	four and a half LIM domains 3	Downregulated
Tub	tubby candidate gene	Downregulated
Tnnt1	troponin T1, skeletal, slow	Downregulated
Ppara	peroxisome proliferator activated receptor alpha	Downregulated
Crybb1	crystallin, beta B1	Downregulated
Mpz	myelin protein zero	Downregulated
Cap2	CAP, adenylate cyclase-associated protein, 2 (yeast)	Downregulated
Prdm8	PR domain containing 8	Downregulated
Myog	myogenin	Downregulated
Cdh11	cadherin 11	Downregulated
Tuba8	tubulin, alpha 8	Downregulated
Eef1a2	eukaryotic translation elongation factor 1 alpha 2	Downregulated
Ret	ret proto-oncogene	Downregulated
Ryr1	ryanodine receptor 1, skeletal muscle	Downregulated
Fgf22	fibroblast growth factor 22	Downregulated
Tmod1	tropomodulin 1	Downregulated
Mapt	microtubule-associated protein tau	Downregulated
Nepn	nephrocan	Downregulated
Mylpf	myosin light chain, phosphorylatable, fast skeletal muscle	Downregulated
Mlxipl	MLX interacting protein-like	Downregulated
Fgfr4	fibroblast growth factor receptor 4	Downregulated
Myl6b	myosin, light polypeptide 6B	Downregulated
Rxrg	retinoid X receptor gamma	Downregulated
Psg16	pregnancy specific glycoprotein 16	Downregulated
Helt	helt bHLH transcription factor	Downregulated
Rbm38	RNA binding motif protein 38	Downregulated
Col6a2	collagen, type VI, alpha 2	Downregulated
Bmp7	bone morphogenetic protein 7	Downregulated
Sall4	sal-like 4 (Drosophila)	Downregulated
Scn1b	sodium channel, voltage-gated, type I, beta	Downregulated
Thrsp	thyroid hormone responsive	Downregulated
Mtfp1	mitochondrial fission process 1	Downregulated
Akr1a1	aldo-keto reductase family 1, member A1 (aldehyde reductase)	Downregulated
Bdkrb1	bradykinin receptor, beta 1	Downregulated
Kiss1	KiSS-1 metastasis-suppressor	Downregulated
Mpp5	membrane protein, palmitoylated 5 (MAGUK p55 subfamily member 5)	Downregulated
Sugp2	SURP and G patch domain containing 2	Downregulated
Lepr	leptin receptor	Downregulated
Nr1d1	nuclear receptor subfamily 1, group D, member 1	Downregulated
Rhoa	ras homolog gene family, member A	Downregulated
Ptx4	pentraxin 4	Downregulated
Hspb2	heat shock protein 2	Downregulated
S100b	S100 protein, beta polypeptide, neural	Downregulated
Tmem232	transmembrane protein 232	Downregulated
Olfr1176	olfactory receptor 1176	Downregulated
Olfr447	olfactory receptor 447	Downregulated
Mettl21c	methyltransferase like 21C	Downregulated
Tmem74b	transmembrane protein 74b	Downregulated
Olfr553	olfactory receptor 553	Downregulated
Slco5a1	solute carrier organic anion transporter family, member 5A1	Downregulated
6030419C18Rik	RIKEN cDNA 6030419C18 gene	Downregulated
Gm889	predicted gene 889	Downregulated
Ccdc62	coiled-coil domain containing 62	Downregulated
Chadl	chondroadherin-like	Downregulated
Tmem225	transmembrane protein 225	Downregulated
Ndufaf6	NADH dehydrogenase (ubiquinone) complex I, assembly factor 6	Downregulated
Fam212b	family with sequence similarity 212, member B	Downregulated

Tmem52	transmembrane protein 52	Downregulated
AI316807	expressed sequence AI316807	Downregulated
Fam57b	family with sequence similarity 57, member B	Downregulated
Cystm1	cysteine-rich transmembrane module containing 1	Downregulated
C2cd4a	C2 calcium-dependent domain containing 4A	Downregulated
Cacna2d1	calcium channel, voltage-dependent, alpha2/delta subunit 1	Downregulated
2300009A05Rik	RIKEN cDNA 2300009A05 gene	Downregulated
Fscn2	fascin homolog 2, actin-bundling protein, retinal (Strongylocentrotus purpuratus)	Downregulated
Mgl2	macrophage galactose N-acetyl-galactosamine specific lectin 2	Downregulated
Ncmap	noncompact myelin associated protein	Downregulated
Gm266	predicted gene 266	Downregulated
Klhl33	kelch-like 33 (Drosophila)	Downregulated
Metrn	meteorin, glial cell differentiation regulator	Downregulated
Ndufs6	NADH dehydrogenase (ubiquinone) Fe-S protein 6	Downregulated
Tmem233	transmembrane protein 233	Downregulated
Tmem8b	transmembrane protein 8B	Downregulated
Atp2b3	ATPase, Ca++ transporting, plasma membrane 3	Downregulated
Atpaf1	ATP synthase mitochondrial F1 complex assembly factor 1	Downregulated
H1fx	H1 histone family, member X	Downregulated
Olfr750	olfactory receptor 750	Downregulated
Itm2a	integral membrane protein 2A	Downregulated
Prx	periaxin	Downregulated
Akap6	A kinase (PRKA) anchor protein 6	Downregulated
1190005I06Rik	RIKEN cDNA 1190005I06 gene	Downregulated
St3gal3	ST3 beta-galactoside alpha-2,3-sialyltransferase 3	Downregulated
Odf3l1	outer dense fiber of sperm tails 3-like 1	Downregulated
Asb15	ankyrin repeat and SOCS box-containing 15	Downregulated
Snai3	snail homolog 3 (Drosophila)	Downregulated
Casq1	calsequestrin 1	Downregulated
Lamtor5	late endosomal/lysosomal adaptor, MAPK and MTOR activator 5	Downregulated
Trim7	tripartite motif-containing 7	Downregulated
Cd163	CD163 antigen	Downregulated
Ndufaf5	NADH dehydrogenase (ubiquinone) complex I, assembly factor 5	Downregulated
Kcp	kielin/chordin-like protein	Downregulated
Sfrp5	secreted frizzled-related sequence protein 5	Downregulated
Art5	ADP-ribosyltransferase 5	Downregulated
Sh3gl3	SH3-domain GRB2-like 3	Downregulated
Tceal5	transcription elongation factor A (SII)-like 5	Downregulated
Homer2	homer homolog 2 (Drosophila)	Downregulated
C1qtnf4	C1q and tumor necrosis factor related protein 4	Downregulated
Podxl2	podocalyxin-like 2	Downregulated
Rragd	Ras-related GTP binding D	Downregulated
Jsrp1	junctional sarcoplasmic reticulum protein 1	Downregulated
Bckdk	branched chain ketoacid dehydrogenase kinase	Downregulated
Pla2g16	phospholipase A2, group XVI	Downregulated
Uqcr11	ubiquinol-cytochrome c reductase, complex III subunit XI	Downregulated
D10Bwg1379e	DNA segment, Chr 10, Brigham & Women's Genetics 1379 expressed	Downregulated
Ston2	stonin 2	Downregulated
Wnk2	WNK lysine deficient protein kinase 2	Downregulated
Sspn	sarcospan	Downregulated
Cox6a2	cytochrome c oxidase subunit VIa polypeptide 2	Downregulated
Msmp	microseminoprotein, prostate associated	Downregulated
Pld5	phospholipase D family, member 5	Downregulated
Exoc3l4	exocyst complex component 3-like 4	Downregulated
Lrtm2	leucine-rich repeats and transmembrane domains 2	Downregulated
1700106J16Rik	RIKEN cDNA 1700106J16 gene	Downregulated
Rgag1	retrotransposon gag domain containing 1	Downregulated
Lingo3	leucine rich repeat and Ig domain containing 3	Downregulated
Gadl1	glutamate decarboxylase-like 1	Downregulated
Itga7	integrin alpha 7	Downregulated
Gys1	glycogen synthase 1, muscle	Downregulated
Nme6	NME/NM23 nucleoside diphosphate kinase 6	Downregulated
Rasgef1c	RasGEF domain family, member 1C	Downregulated
Ccdc30	coiled-coil domain containing 30	Downregulated
Agap1	ArfGAP with GTPase domain, ankyrin repeat and PH domain 1	Downregulated
Ehbp1l1	EH domain binding protein 1-like 1	Downregulated
Dtna	dystrobrevin alpha	Downregulated
Phospho1	phosphatase, orphan 1	Downregulated
Popdc2	popeye domain containing 2	Downregulated
Tecr	trans-2,3-enoyl-CoA reductase	Downregulated
Plekhh3	pleckstrin homology domain containing, family H (with MyTH4 domain) member 3	Downregulated
Fitm1	fat storage-inducing transmembrane protein 1	Downregulated
Ubac1	ubiquitin associated domain containing 1	Downregulated
Ndufs3	NADH dehydrogenase (ubiquinone) Fe-S protein 3	Downregulated
Hist2h2aa2|Hist2h2aa1	histone cluster 2, H2aa2|histone cluster 2, H2aa1	Downregulated
Sh3bgr	SH3-binding domain glutamic acid-rich protein	Downregulated
Sema6c	sema domain, transmembrane domain (TM), and cytoplasmic domain, (semaphorin) 6C	Downregulated
Hes3	hairy and enhancer of split 3 (Drosophila)	Downregulated
Pfkfb1	6-phosphofructo-2-kinase/fructose-2,6-biphosphatase 1	Downregulated
Sptb	spectrin beta, erythrocytic	Downregulated
Dusp13	dual specificity phosphatase 13	Downregulated
Pdk2	pyruvate dehydrogenase kinase, isoenzyme 2	Downregulated
Plin5	perilipin 5	Downregulated
Adssl1	adenylosuccinate synthetase like 1	Downregulated
Ldb3	LIM domain binding 3	Downregulated
Obscn	obscurin, cytoskeletal calmodulin and titin-interacting RhoGEF	Downregulated
Mmel1	membrane metallo-endopeptidase-like 1	Downregulated
Sel1l2	sel-1 suppressor of lin-12-like 2 (C. elegans)	Downregulated
Mybph	myosin binding protein H	Downregulated
Usp2	ubiquitin specific peptidase 2	Downregulated
Car14	carbonic anhydrase 14	Downregulated
Slc2a12	solute carrier family 2 (facilitated glucose transporter), member 12	Downregulated
Lrrc30	leucine rich repeat containing 30	Downregulated
Thap3	THAP domain containing, apoptosis associated protein 3	Downregulated
Plekha6	pleckstrin homology domain containing, family A member 6	Downregulated
Nudt8	nudix (nucleoside diphosphate linked moiety X)-type motif 8	Downregulated
Rapsn	receptor-associated protein of the synapse	Downregulated
Frat2	frequently rearranged in advanced T cell lymphomas 2	Downregulated

Slc41a3	solute carrier family 41, member 3	Downregulated
G0s2	G0/G1 switch gene 2	Downregulated
Klhl8	kelch-like 8 (Drosophila)	Downregulated
Gamt	guanidinoacetate methyltransferase	Downregulated
Dyrk1b	dual-specificity tyrosine-(Y)-phosphorylation regulated kinase 1b	Downregulated
Cox5b	cytochrome c oxidase subunit Vb	Downregulated
Tst	thiosulfate sulfurtransferase, mitochondrial	Downregulated
Ptges2	prostaglandin E synthase 2	Downregulated
Smarcd3	SWI/SNF related, matrix associated, actin dependent regulator of chromatin, subfamily d, mem	Downregulated
Mus81	MUS81 endonuclease homolog (yeast)	Downregulated
Crb3	crumbs homolog 3 (Drosophila)	Downregulated
Bcam	basal cell adhesion molecule	Downregulated
Scube2	signal peptide, CUB domain, EGF-like 2	Downregulated
Cenpv	centromere protein V	Downregulated
Trim72	tripartite motif-containing 72	Downregulated
Coq9	coenzyme Q9 homolog (yeast)	Downregulated
Dgat2	diacylglycerol O-acyltransferase 2	Downregulated
Cuedc1	CUE domain containing 1	Downregulated
Slc25a29	solute carrier family 25 (mitochondrial carrier, palmitoylcarnitine transporter), member 29	Downregulated
Trim54	tripartite motif-containing 54	Downregulated
Slc35e1	solute carrier family 35, member E1	Downregulated
Hoxc10	homeobox C10	Downregulated
Inha	inhibin alpha	Downregulated
Map2k6	mitogen-activated protein kinase kinase 6	Downregulated
Fam20c	family with sequence similarity 20, member C	Downregulated
Ufsp1	UFM1-specific peptidase 1	Downregulated
Fam222b	family with sequence similarity 222, member B	Downregulated
Hpn	hepsin	Downregulated
Mfap4	microfibrillar-associated protein 4	Downregulated
Defb25	defensin beta 25	Downregulated
Cib2	calcium and integrin binding family member 2	Downregulated
Wdr70	WD repeat domain 70	Downregulated
Kcnc4	potassium voltage gated channel, Shaw-related subfamily, member 4	Downregulated
Pmp2	peripheral myelin protein 2	Downregulated
Gm3646	predicted gene 3646	Downregulated
Oplah	5-oxoprolinase (ATP-hydrolysing)	Downregulated
Mdga1	MAM domain containing glycosylphosphatidylinositol anchor 1	Downregulated
Zdhhc8	zinc finger, DHHC domain containing 8	Downregulated
Mchr1	melanin-concentrating hormone receptor 1	Downregulated
Sec31b	Sec31 homolog B (S. cerevisiae)	Downregulated
Colq	collagen-like tail subunit (single strand of homotrimer) of asymmetric acetylcholinesterase	Downregulated
Angptl2	angiopoietin-like 2	Downregulated
Fxyd1	FXYD domain-containing ion transport regulator 1	Downregulated
Cacng6	calcium channel, voltage-dependent, gamma subunit 6	Downregulated
Foxo6	forkhead box O6	Downregulated
Odf3l2	outer dense fiber of sperm tails 3-like 2	Downregulated
Ckmt2	creatine kinase, mitochondrial 2	Downregulated
Neurl2	neuralized-like 2 (Drosophila)	Downregulated
Gm7325	predicted gene 7325	Downregulated
Scn4b	sodium channel, type IV, beta	Downregulated
Asb12	ankyrin repeat and SOCS box-containing 12	Downregulated
Defb6	defensin beta 6	Downregulated
Mylk4	myosin light chain kinase family, member 4	Downregulated
Gstm7	glutathione S-transferase, mu 7	Downregulated
Clec2e	C-type lectin domain family 2, member e	Downregulated
Tmem38a	transmembrane protein 38A	Downregulated
Klk1b16	kallikrein 1-related peptidase b16	Downregulated
Unc5a	unc-5 homolog A (C. elegans)	Downregulated
Fbxo40	F-box protein 40	Downregulated
Whamm	WAS protein homolog associated with actin, golgi membranes and microtubules	Downregulated
Adprhl1	ADP-ribosylhydrolase like 1	Downregulated
Dupd1	dual specificity phosphatase and pro isomerase domain containing 1	Downregulated
Iqca	IQ motif containing with AAA domain	Downregulated
Lgi4	leucine-rich repeat LGI family, member 4	Downregulated
Tmem8c	transmembrane protein 8C	Downregulated
Mrpl55	mitochondrial ribosomal protein L55	Downregulated
Cd59b	CD59b antigen	Downregulated
Car11	carbonic anhydrase 11	Downregulated
Clcn1	chloride channel 1	Downregulated
Ldhd	lactate dehydrogenase D	Downregulated
Smtnl1	smoothelin-like 1	Downregulated
Ube2d1	ubiquitin-conjugating enzyme E2D 1	Downregulated
Dusp22	dual specificity phosphatase 22	Downregulated
Capn11	calpain 11	Downregulated
Slc25a34	solute carrier family 25, member 34	Downregulated
Acvr2b	activin receptor IIB	Downregulated
Josd2	Josephin domain containing 2	Downregulated
Drp2	dystrophin related protein 2	Downregulated
Atp5o	ATP synthase, H+ transporting, mitochondrial F1 complex, O subunit	Downregulated
Zfp667	zinc finger protein 667	Downregulated
Sars2	seryl-aminoacyl-tRNA synthetase 2	Downregulated
Clic3	chloride intracellular channel 3	Downregulated
Rtn2	reticulon 2 (Z-band associated protein)	Downregulated
Gmpr	guanosine monophosphate reductase	Downregulated
Camk2b	calcium/calmodulin-dependent protein kinase II, beta	Downregulated
Dlgap4	discs, large homolog-associated protein 4 (Drosophila)	Downregulated
Arfgap2	ADP-ribosylation factor GTPase activating protein 2	Downregulated
Cacnb1	calcium channel, voltage-dependent, beta 1 subunit	Downregulated
Nkain1	Na+/K+ transporting ATPase interacting 1	Downregulated
Ampd1	adenosine monophosphate deaminase 1	Downregulated
Ppp2r3a	protein phosphatase 2, regulatory subunit B'', alpha	Downregulated
Smcr7	Smith-Magenis syndrome chromosome region, candidate 7 homolog (human)	Downregulated
Acsm2	acyl-CoA synthetase medium-chain family member 2	Downregulated
Zfp385a	zinc finger protein 385A	Downregulated
Phka1	phosphorylase kinase alpha 1	Downregulated
Kdm3b	KDM3B lysine (K)-specific demethylase 3B	Downregulated
Rilp	Rab interacting lysosomal protein	Downregulated
Adamts13	a disintegrin-like and metallopeptidase (reprolysin type) with thrombospondin type 1 motif, 13	Downregulated

Aimp2	aminoacyl tRNA synthetase complex-interacting multifunctional protein 2	Downregulated
Apobec2	apolipoprotein B mRNA editing enzyme, catalytic polypeptide 2	Downregulated
Pcx	pyruvate carboxylase	Downregulated
Slc9a2	solute carrier family 9 (sodium/hydrogen exchanger), member 2	Downregulated
Hist1h4i	histone cluster 1, H4i	Downregulated
Iglon5	IgLON family member 5	Downregulated
Kif1c	kinesin family member 1C	Downregulated
Coq10a	coenzyme Q10 homolog A (yeast)	Downregulated
Trim55	tripartite motif-containing 55	Downregulated
Asphd1	aspartate beta-hydroxylase domain containing 1	Downregulated
1700020A23Rik	RIKEN cDNA 1700020A23 gene	Downregulated
Popdc2	popeye domain containing 2	Downregulated
Use1	unconventional SNARE in the ER 1 homolog (S. cerevisiae)	Downregulated
Aimp2	aminoacyl tRNA synthetase complex-interacting multifunctional protein 2	Downregulated
Hspb2	heat shock protein 2	Downregulated
Tmem25	transmembrane protein 25	Downregulated
2310047D07Rik	RIKEN cDNA 2310047D07 gene	Downregulated
2310010M20Rik	RIKEN cDNA 2310010M20 gene	Downregulated
Hist1h1e	histone cluster 1, H1e	Downregulated
Sct	secretin	Downregulated
Hrc	histidine rich calcium binding protein	Downregulated
Cilp2	cartilage intermediate layer protein 2	Downregulated
Cort	cortistatin	Downregulated
2310022A10Rik	RIKEN cDNA 2310022A10 gene	Downregulated
Fam198a	family with sequence similarity 198, member A	Downregulated
Map3k10	mitogen-activated protein kinase kinase kinase 10	Downregulated
Tmem88b	transmembrane protein 88B	Downregulated
Crip3	cysteine-rich protein 3	Downregulated
Pbxip1	pre B cell leukemia transcription factor interacting protein 1	Downregulated
H2-M10.4	histocompatibility 2, M region locus 10.4	Downregulated
Scn4a	sodium channel, voltage-gated, type IV, alpha	Downregulated
Cdh4	cadherin 4	Downregulated
Olfr378	olfactory receptor 378	Downregulated
Hepacam2	HEPACAM family member 2	Downregulated
Trpt1	tRNA phosphotransferase 1	Downregulated
Rab11fip3	RAB11 family interacting protein 3 (class II)	Downregulated
Nudt18	nudix (nucleoside diphosphate linked moiety X)-type motif 18	Downregulated
Rgma	RGM domain family, member A	Downregulated
Zswim7	zinc finger SWIM-type containing 7	Downregulated
Bhmt2	betaine-homocysteine methyltransferase 2	Downregulated
Acsl6	acyl-CoA synthetase long-chain family member 6	Downregulated
Macrod1	MACRO domain containing 1	Downregulated
Ppm1j	protein phosphatase 1J	Downregulated
Fasn	fatty acid synthase	Downregulated
Fahd2a	fumarylacetoacetate hydrolase domain containing 2A	Downregulated
Ecsit	ECSIT homolog (Drosophila)	Downregulated
Sgcg	sarcoglycan, gamma (dystrophin-associated glycoprotein)	Downregulated
Wdtc1	WD and tetratricopeptide repeats 1	Downregulated
Fuom	fucose mutarotase	Downregulated
Dcaf11	DDB1 and CUL4 associated factor 11	Downregulated
Lmod1	leiomodin 1 (smooth muscle)	Downregulated
Cox7a1	cytochrome c oxidase subunit VIIa 1	Downregulated
Clcnka	chloride channel Ka	Downregulated
Mrgprh	MAS-related GPR, member H	Downregulated
Ip6k3	inositol hexaphosphate kinase 3	Downregulated
Gpt	glutamic pyruvic transaminase, soluble	Downregulated
Ablim2	actin-binding LIM protein 2	Downregulated
Oscar	osteoclast associated receptor	Downregulated
Rtl1	retrotransposon-like 1	Downregulated
Cyb5rl	cytochrome b5 reductase-like	Downregulated
Ak1	adenylate kinase 1	Downregulated
Cst13	cystatin 13	Downregulated
Tef	thyrotroph embryonic factor	Downregulated
Kcng4	potassium voltage-gated channel, subfamily G, member 4	Downregulated
Pkdcc	protein kinase domain containing, cytoplasmic	Downregulated
Pecr	peroxisomal trans-2-enoyl-CoA reductase	Downregulated
Sbk1	SH3-binding kinase 1	Downregulated
Hoxd9	homeobox D9	Downregulated
Stbd1	starch binding domain 1	Downregulated
Cxxc5	CXXC finger 5	Downregulated
Myh8	myosin, heavy polypeptide 8, skeletal muscle, perinatal	Downregulated
Col22a1	collagen, type XXII, alpha 1	Downregulated
Mapre3	microtubule-associated protein, RP/EB family, member 3	Downregulated
Cyhr1	cysteine and histidine rich 1	Downregulated
Tpm2	tropomyosin 2, beta	Downregulated
Tcea3	transcription elongation factor A (SII), 3	Downregulated
Prickle3	prickle homolog 3 (Drosophila)	Downregulated
Prkab2	protein kinase, AMP-activated, beta 2 non-catalytic subunit	Downregulated
Ank1	ankyrin 1, erythroid	Downregulated
Nr4a1	nuclear receptor subfamily 4, group A, member 1	Downregulated
Kcnj11	potassium inwardly rectifying channel, subfamily J, member 11	Downregulated
Rap1gap	Rap1 GTPase-activating protein	Downregulated
Pvalb	parvalbumin	Downregulated
Capn3	calpain 3	Downregulated
Hoxc9	homeobox C9	Downregulated
Mapk8ip1	mitogen-activated protein kinase 8 interacting protein 1	Downregulated
0610011F06Rik	RIKEN cDNA 0610011F06 gene	Downregulated
Eya1	eyes absent 1 homolog (Drosophila)	Downregulated
Dusp23	dual specificity phosphatase 23	Downregulated
4933436C20Rik	RIKEN cDNA 4933436C20 gene	Downregulated
Pnpla3	patatin-like phospholipase domain containing 3	Downregulated
Myl7	myosin, light polypeptide 7, regulatory	Downregulated
Nhlrc1	NHL repeat containing 1	Downregulated
Hist1h4h	histone cluster 1, H4h	Downregulated
Osbpl5	oxysterol binding protein-like 5	Downregulated
Dok7	docking protein 7	Downregulated
Mmp15	matrix metallopeptidase 15	Downregulated
Kcne1l	potassium voltage-gated channel, Isk-related family, member 1-like, pseudogene	Downregulated

Klf15	Kruppel-like factor 15	Downregulated
Capn3	calpain 3	Downregulated
Clta	clathrin, light polypeptide (Lca)	Downregulated
Mb	myoglobin	Downregulated
Abhd11	abhydrolase domain containing 11	Downregulated
Trpt1	tRNA phosphotransferase 1	Downregulated
Trim46	tripartite motif-containing 46	Downregulated
Gm8273	predicted gene 8273	Downregulated
Gm12538	predicted gene 12538	Downregulated
Gm5860	predicted gene 5860	Downregulated
Gm6307	predicted gene 6307	Downregulated
7530428D23Rik	RIKEN cDNA 7530428D23 gene	Downregulated
Igfn1	immunoglobulin-like and fibronectin type III domain containing 1	Downregulated
Gm10440	predicted gene 10440	Downregulated
2310040G24Rik	RIKEN cDNA 2310040G24 gene	Downregulated
Ovol3	OVO homolog-like 3 (Drosophila)	Downregulated
Gm6567	predicted gene 6567	Downregulated
B230311B06Rik	RIKEN cDNA B230311B06 gene	Downregulated
Gm1078	predicted gene 1078	Downregulated
Gm9507	predicted gene 9507	Downregulated
LOC101055810	uncharacterized LOC101055810	Downregulated
D630033O11Rik	RIKEN cDNA D630033O11 gene	Downregulated
3425401B19Rik	RIKEN cDNA 3425401B19 gene	Downregulated
AI197445	expressed sequence AI197445	Downregulated
1110054M08Rik	RIKEN cDNA 1110054M08 gene	Downregulated
LOC101056249	uncharacterized LOC101056249	Downregulated
Gm4544	predicted gene 4544	Downregulated
Prob1	proline rich basic protein 1	Downregulated
Omt2a	oocyte maturation, alpha	Downregulated
2310002L09Rik	RIKEN cDNA 2310002L09 gene	Downregulated
Synpo	synaptopodin	Downregulated
Etnk2	ethanolamine kinase 2	Downregulated
Degs2	degenerative spermatocyte homolog 2 (Drosophila), lipid desaturase	Downregulated
Rpl19	ribosomal protein L19	Downregulated
Dtna	dystrobrevin alpha	Downregulated
Obscn	obscurin, cytoskeletal calmodulin and titin-interacting RhoGEF	Downregulated
Cstf3	cleavage stimulation factor, 3' pre-RNA, subunit 3	Downregulated
Rtn2	reticulon 2 (Z-band associated protein)	Downregulated
Arl2bp	ADP-ribosylation factor-like 2 binding protein	Downregulated
Grin1	glutamate receptor, ionotropic, NMDA1 (zeta 1)	Downregulated
Rpl3l	ribosomal protein L3-like	Downregulated
Rpl3l	ribosomal protein L3-like	Downregulated
Ptpla	protein tyrosine phosphatase-like (proline instead of catalytic arginine), member a	Downregulated
Capzb	capping protein (actin filament) muscle Z-line, beta	Downregulated
Naca	nascent polypeptide-associated complex alpha polypeptide	Downregulated
Sgca	sarcoglycan, alpha (dystrophin-associated glycoprotein)	Downregulated
Birc5	baculoviral IAP repeat-containing 5	Downregulated
Sts	steroid sulfatase	Downregulated
Snrpn|Snurf|Gm5802	small nuclear ribonucleoprotein N|SNRPN upstream reading frame|predicted gene 5802	Downregulated
Fem1a	feminization 1 homolog a (C. elegans)	Downregulated
Myh7|LOC100862557	myosin, heavy polypeptide 7, cardiac muscle, beta|myosin-6-like	Downregulated
Fbxo31	F-box protein 31	Downregulated
Dnaja4	DnaJ (Hsp40) homolog, subfamily A, member 4	Downregulated
Ramp1	receptor (calcitonin) activity modifying protein 1	Downregulated
1190007I07Rik	RIKEN cDNA 1190007I07 gene	Downregulated
Bckdha	branched chain ketoacid dehydrogenase E1, alpha polypeptide	Downregulated
Gfra3	glial cell line derived neurotrophic factor family receptor alpha 3	Downregulated
Rbfox1	RNA binding protein, fox-1 homolog (C. elegans) 1	Downregulated
Ehbp1l1	EH domain binding protein 1-like 1	Downregulated
Rps6ka2	ribosomal protein S6 kinase, polypeptide 2	Downregulated
2700046G09Rik	RIKEN cDNA 2700046G09 gene	Downregulated
Nkain1	Na+/K+ transporting ATPase interacting 1	Downregulated
Synm	synemin, intermediate filament protein	Downregulated
Unc45b	unc-45 homolog B (C. elegans)	Downregulated
Agt	angiotensinogen (serpin peptidase inhibitor, clade A, member 8)	Downregulated
Arx	aristaless related homeobox	Downregulated
Homer2	homer homolog 2 (Drosophila)	Downregulated
Lrtm1	leucine-rich repeats and transmembrane domains 1	Downregulated
Six2	sine oculis-related homeobox 2	Downregulated
A530095I07Rik	RIKEN cDNA A530095I07 gene	Downregulated
Arfgap2	ADP-ribosylation factor GTPase activating protein 2	Downregulated
Tusc2	tumor suppressor candidate 2	Downregulated
2310015D24Rik	RIKEN cDNA 2310015D24 gene	Downregulated
Pde4d	phosphodiesterase 4D, cAMP specific	Downregulated
0610009L18Rik	RIKEN cDNA 0610009L18 gene	Downregulated
Adamts8	a disintegrin-like and metallopeptidase (reprolysin type) with thrombospondin type 1 motif, 8	Downregulated
Cacna2d1	calcium channel, voltage-dependent, alpha2/delta subunit 1	Downregulated
Gprin1	G protein-regulated inducer of neurite outgrowth 1	Downregulated
Actc1	actin, alpha, cardiac muscle 1	Downregulated
Stim1	stromal interaction molecule 1	Downregulated
Ren2|Ren1|LOC100044656	renin 2 tandem duplication of Ren1|renin 1 structural|renin-1-like	Downregulated
Hemk1	HemK methyltransferase family member 1	Downregulated
Cst6	cystatin E/M	Downregulated
Ldhb	lactate dehydrogenase B	Downregulated
Mfsd3	major facilitator superfamily domain containing 3	Downregulated
Rangrf	RAN guanine nucleotide release factor	Downregulated
Ubac1	ubiquitin associated domain containing 1	Downregulated
Myoz3	myozenin 3	Downregulated
Gm21685|Entpd4	predicted gene, 21685|ectonucleoside triphosphate diphosphohydrolase 4	Downregulated
Gm10516	predicted gene 10516	Downregulated
Fsbp|Rad54b	fibrinogen silencer binding protein|RAD54 homolog B (S. cerevisiae)	Downregulated
LOC101055685	FERM and PDZ domain-containing protein 2-like	Downregulated
Fbxl22	F-box and leucine-rich repeat protein 22	Downregulated
Proser2	proline and serine rich 2	Downregulated
Kcnh2	potassium voltage-gated channel, subfamily H (eag-related), member 2	Downregulated
Ndufaf5	NADH dehydrogenase (ubiquinone) complex I, assembly factor 5	Downregulated
Aqp4	aquaporin 4	Downregulated
Cpt1b|BC090627	carnitine palmitoyltransferase 1b, muscle|cDNA sequence BC090627	Downregulated

Lrrc30	leucine rich repeat containing 30	Downregulated
2810416G20Rik|LOC101056	RIKEN cDNA 2810416G20 gene|uncharacterized LOC101056638	Downregulated
Vstm2b	V-set and transmembrane domain containing 2B	Downregulated
Srf	serum response factor	Downregulated
Osbpl10	oxysterol binding protein-like 10	Downregulated
Sec31b	Sec31 homolog B (S. cerevisiae)	Downregulated
Ppp1r14c|Gm14057	protein phosphatase 1, regulatory (inhibitor) subunit 14c|Ppp1r14c pseudogene	Downregulated
9630033F20Rik	RIKEN cDNA 9630033F20 gene	Downregulated
Htr3a	5-hydroxytryptamine (serotonin) receptor 3A	Downregulated
Myo18b	myosin XVIIIb	Downregulated
Casq1	calsequestrin 1	Downregulated
0610038B21Rik	RIKEN cDNA 0610038B21 gene	Downregulated
Neurl1a	neuralized homolog 1A (Drosophila)	Downregulated
Lynx1	Ly6/neurotoxin 1	Downregulated
Gria1	glutamate receptor, ionotropic, AMPA1 (alpha 1)	Downregulated
Tbx1	T-box 1	Downregulated
4930556A20Rik	RIKEN cDNA 4930556A20 gene	Downregulated
Gm9895	predicted gene 9895	Downregulated
A930003A15Rik	RIKEN cDNA A930003A15 gene	Downregulated
Trp63	transformation related protein 63	Downregulated
Ldb3	LIM domain binding 3	Downregulated
Asph	aspartate-beta-hydroxylase	Downregulated
Pdlim7	PDZ and LIM domain 7	Downregulated
Dcaf17	DDB1 and CUL4 associated factor 17	Downregulated
Dlgap1	discs, large (Drosophila) homolog-associated protein 1	Downregulated
Ldb3	LIM domain binding 3	Downregulated
Otof	otoferlin	Downregulated
Cacnb1	calcium channel, voltage-dependent, beta 1 subunit	Downregulated
Ccdc125	coiled-coil domain containing 125	Downregulated
Lrrc56	leucine rich repeat containing 56	Downregulated
Camk2a	calcium/calmodulin-dependent protein kinase II alpha	Downregulated
Popdc2	popeye domain containing 2	Downregulated
Nags	N-acetylglutamate synthase	Downregulated
A930013B10Rik	RIKEN cDNA A930013B10 gene	Downregulated
1700111N16Rik	RIKEN cDNA 1700111N16 gene	Downregulated
Mir598	microRNA 598	Downregulated
Gm16119	predicted gene 16119	Downregulated
Rs5-8s1	5.8S ribosomal RNA	Downregulated
Gm5144	predicted gene 5144	Downregulated
Mir195	microRNA 195	Downregulated
2310050B05Rik	RIKEN cDNA 2310050B05 gene	Downregulated
9430021M05Rik	RIKEN cDNA 9430021M05 gene	Downregulated
Stim1	stromal interaction molecule 1	Downregulated
Adamts8	a disintegrin-like and metallopeptidase (reprolysin type) with thrombospondin type 1 motif, 8	Downregulated
Eda	ectodysplasin-A	Downregulated
Gprc5c	G protein-coupled receptor, family C, group 5, member C	Downregulated
Usp51	ubiquitin specific protease 51	Downregulated
Cpt1b|BC090627	carnitine palmitoyltransferase 1b, muscle|cDNA sequence BC090627	Downregulated
Neurl1a	neuralized homolog 1A (Drosophila)	Downregulated
Ly6h	lymphocyte antigen 6 complex, locus H	Downregulated
Ptprj	protein tyrosine phosphatase, receptor type, J	Downregulated
Camta2	calmodulin binding transcription activator 2	Downregulated
Plk2	polo-like kinase 2	Upregulated
Cd96	CD96 antigen	Upregulated
Dlx4	distal-less homeobox 4	Upregulated
Il33	interleukin 33	Upregulated
Trem2	triggering receptor expressed on myeloid cells 2	Upregulated
Cxcl9	chemokine (C-X-C motif) ligand 9	Upregulated
Cdca5	cell division cycle associated 5	Upregulated
Nckap1l	NCK associated protein 1 like	Upregulated
Prss16	protease, serine, 16 (thymus)	Upregulated
9130206I24Rik	RIKEN cDNA 9130206I24 gene	Upregulated
Lck	lymphocyte protein tyrosine kinase	Upregulated
Tex13	testis expressed gene 13	Upregulated
LOC101056558|Apol10b	apolipoprotein L3-like|apolipoprotein L 10B	Upregulated
Exosc8	exosome component 8	Upregulated
Sla	src-like adaptor	Upregulated
Rsad2	radical S-adenosyl methionine domain containing 2	Upregulated
Cd27	CD27 antigen	Upregulated
Oasl1	2'-5' oligoadenylate synthetase-like 1	Upregulated
Cstb	cystatin B	Upregulated
Slc25a24	solute carrier family 25 (mitochondrial carrier, phosphate carrier), member 24	Upregulated
Iqgap1	IQ motif containing GTPase activating protein 1	Upregulated
Lgals8	lectin, galactose binding, soluble 8	Upregulated
Gpr65	G-protein coupled receptor 65	Upregulated
Cd48	CD48 antigen	Upregulated
Naip6	NLR family, apoptosis inhibitory protein 6	Upregulated
Casc5	cancer susceptibility candidate 5	Upregulated
Cenpl	centromere protein L	Upregulated
Apol11b	apolipoprotein L 11b	Upregulated
Racgap1	Rac GTPase-activating protein 1	Upregulated
Slc20a1	solute carrier family 20, member 1	Upregulated
Asns	asparagine synthetase	Upregulated
Zfp800	zinc finger protein 800	Upregulated
Ctsw	cathepsin W	Upregulated
Gda	guanine deaminase	Upregulated
Pnrc2	proline-rich nuclear receptor coactivator 2	Upregulated
Dhfr	dihydrofolate reductase	Upregulated
Cfb	complement factor B	Upregulated
Cysltr1	cysteinyl leukotriene receptor 1	Upregulated
Ptpn18	protein tyrosine phosphatase, non-receptor type 18	Upregulated
Klrg2	killer cell lectin-like receptor subfamily G, member 2	Upregulated
Id2	inhibitor of DNA binding 2	Upregulated
Gbp3	guanylate binding protein 3	Upregulated
Slc12a7	solute carrier family 12, member 7	Upregulated
Msn	moesin	Upregulated
Stk17b	serine/threonine kinase 17b (apoptosis-inducing)	Upregulated
Guca1a	guanylate cyclase activator 1a (retina)	Upregulated

Zbtb32	zinc finger and BTB domain containing 32	Upregulated
Wbp5	WW domain binding protein 5	Upregulated
Naip2	NLR family, apoptosis inhibitory protein 2	Upregulated
Rerg	RAS-like, estrogen-regulated, growth-inhibitor	Upregulated
Laptm5	lysosomal-associated protein transmembrane 5	Upregulated
Ctsz	cathepsin Z	Upregulated
Lyar	Ly1 antibody reactive clone	Upregulated
Unc13d	unc-13 homolog D (C. elegans)	Upregulated
Capg	capping protein (actin filament), gelsolin-like	Upregulated
Mettl10	methyltransferase like 10	Upregulated
Cdca3	cell division cycle associated 3	Upregulated
Cdc7	cell division cycle 7 (S. cerevisiae)	Upregulated
Ralb	v-ral simian leukemia viral oncogene homolog B (ras related)	Upregulated
Dram1	DNA-damage regulated autophagy modulator 1	Upregulated
Mak	male germ cell-associated kinase	Upregulated
Dmxl1	Dmx-like 1	Upregulated
Slc15a3	solute carrier family 15, member 3	Upregulated
Dis3	DIS3 mitotic control homolog (S. cerevisiae)	Upregulated
Lgals9	lectin, galactose binding, soluble 9	Upregulated
Kif22	kinesin family member 22	Upregulated
Lrmp	lymphoid-restricted membrane protein	Upregulated
Pif1	PIF1 5'-to-3' DNA helicase homolog (S. cerevisiae)	Upregulated
Bst1	bone marrow stromal cell antigen 1	Upregulated
Slc39a10	solute carrier family 39 (zinc transporter), member 10	Upregulated
Mfsd1	major facilitator superfamily domain containing 1	Upregulated
Sqle	squalene epoxidase	Upregulated
Kif4	kinesin family member 4	Upregulated
Ms4a8a	membrane-spanning 4-domains, subfamily A, member 8A	Upregulated
Rab1	RAB1, member RAS oncogene family	Upregulated
Tbxas1	thromboxane A synthase 1, platelet	Upregulated
Orc6	origin recognition complex, subunit 6	Upregulated
Slc39a6	solute carrier family 39 (metal ion transporter), member 6	Upregulated
Pik3cg	phosphoinositide-3-kinase, catalytic, gamma polypeptide	Upregulated
Sepp1	selenoprotein P, plasma, 1	Upregulated
Fbxo15	F-box protein 15	Upregulated
Zfp160	zinc finger protein 160	Upregulated
Mlf1ip	myeloid leukemia factor 1 interacting protein	Upregulated
Epha2	Eph receptor A2	Upregulated
Tex19.2	testis expressed gene 19.2	Upregulated
Tns4	tensin 4	Upregulated
Cmpk2	cytidine monophosphate (UMP-CMP) kinase 2, mitochondrial	Upregulated
Gipc2	GIPC PDZ domain containing family, member 2	Upregulated
Sirt1	sirtuin 1 (silent mating type information regulation 2, homolog) 1 (S. cerevisiae)	Upregulated
Emp1	epithelial membrane protein 1	Upregulated
2810408I11Rik	RIKEN cDNA 2810408I11 gene	Upregulated
Dlx1	distal-less homeobox 1	Upregulated
Pla2g7	phospholipase A2, group VII (platelet-activating factor acetylhydrolase, plasma)	Upregulated
Ccl7	chemokine (C-C motif) ligand 7	Upregulated
Dusp4	dual specificity phosphatase 4	Upregulated
Adam9	a disintegrin and metallopeptidase domain 9 (meltrin gamma)	Upregulated
Tex11	testis expressed gene 11	Upregulated
Brca2	breast cancer 2	Upregulated
Cd93	CD93 antigen	Upregulated
Prps2	phosphoribosyl pyrophosphate synthetase 2	Upregulated
Lyve1	lymphatic vessel endothelial hyaluronan receptor 1	Upregulated
Isl2	insulin related protein 2 (islet 2)	Upregulated
Otx1	orthodenticle homolog 1 (Drosophila)	Upregulated
Ppm1h	protein phosphatase 1H (PP2C domain containing)	Upregulated
5730508B09Rik	RIKEN cDNA 5730508B09 gene	Upregulated
Cxadr	coxsackie virus and adenovirus receptor	Upregulated
Bora	bora, aurora kinase A activator	Upregulated
Fcgr4	Fc receptor, IgG, low affinity IV	Upregulated
Dock2	dedicator of cyto-kinesis 2	Upregulated
Clhc1	clathrin heavy chain linker domain containing 1	Upregulated
Tex19.1	testis expressed gene 19.1	Upregulated
Aim1	absent in melanoma 1	Upregulated
Sp100	nuclear antigen Sp100	Upregulated
Ak8	adenylate kinase 8	Upregulated
Wisp2	WNT1 inducible signaling pathway protein 2	Upregulated
Slamf7	SLAM family member 7	Upregulated
Wdhd1	WD repeat and HMG-box DNA binding protein 1	Upregulated
Nadkd1	NAD kinase domain containing 1	Upregulated
Cybb	cytochrome b-245, beta polypeptide	Upregulated
Fam26f	family with sequence similarity 26, member F	Upregulated
Sema4f	sema domain, immunoglobulin domain (Ig), TM domain, and short cytoplasmic domain	Upregulated
Rab32	RAB32, member RAS oncogene family	Upregulated
Fgfr2	fibroblast growth factor receptor 2	Upregulated
Xcl1	chemokine (C motif) ligand 1	Upregulated
Ccl12|LOC100862578	chemokine (C-C motif) ligand 12|c-C motif chemokine 12-like	Upregulated
Tank	TRAF family member-associated Nf-kappa B activator	Upregulated
Lgmn	legumain	Upregulated
Psmb10	proteasome (prosome, macropain) subunit, beta type 10	Upregulated
Prpf39	PRP39 pre-mRNA processing factor 39 homolog (yeast)	Upregulated
Atg3	autophagy related 3	Upregulated
Btk	Bruton agammaglobulinemia tyrosine kinase	Upregulated
Ubd	ubiquitin D	Upregulated
Sirpa	signal-regulatory protein alpha	Upregulated
Ankrd42	ankyrin repeat domain 42	Upregulated
Grhl2	grainyhead-like 2 (Drosophila)	Upregulated
Fcho2	FCH domain only 2	Upregulated
Hat1	histone aminotransferase 1	Upregulated
Igsf6	immunoglobulin superfamily, member 6	Upregulated
Il24	interleukin 24	Upregulated
Gngt2	guanine nucleotide binding protein (G protein), gamma transducing activity polypeptide 2	Upregulated
Syce3	synaptonemal complex central element protein 3	Upregulated
Psmb9	proteasome (prosome, macropain) subunit, beta type 9 (large multifunctional peptidase 2)	Upregulated
Mrc1	mannose receptor, C type 1	Upregulated
Krcc1	lysine-rich coiled-coil 1	Upregulated

Pld4	phospholipase D family, member 4	Upregulated
Cd3d	CD3 antigen, delta polypeptide	Upregulated
Lmnb1	lamin B1	Upregulated
Smim15	small integral membrane protein 15	Upregulated
Sash3	SAM and SH3 domain containing 3	Upregulated
Cntln	centlein, centrosomal protein	Upregulated
Trip13	thyroid hormone receptor interactor 13	Upregulated
Prc1	protein regulator of cytokinesis 1	Upregulated
Tlr7	toll-like receptor 7	Upregulated
Fhl2	four and a half LIM domains 2	Upregulated
Klrd1	killer cell lectin-like receptor, subfamily D, member 1	Upregulated
Avpr1a	arginine vasopressin receptor 1A	Upregulated
Chmp1b	charged multivesicular body protein 1B	Upregulated
Cxcl13	chemokine (C-X-C motif) ligand 13	Upregulated
Inhbe	inhibin beta E	Upregulated
Sh3bp2	SH3-domain binding protein 2	Upregulated
Irgm1	immunity-related GTPase family M member 1	Upregulated
Ccl3	chemokine (C-C motif) ligand 3	Upregulated
Bcar3	breast cancer anti-estrogen resistance 3	Upregulated
Cep192	centrosomal protein 192	Upregulated
Mcoln2	mucolipin 2	Upregulated
Itsn2	intersectin 2	Upregulated
1700026L06Rik	RIKEN cDNA 1700026L06 gene	Upregulated
Maats1	MYCBP-associated, testis expressed 1	Upregulated
Slamf8	SLAM family member 8	Upregulated
Gjb3	gap junction protein, beta 3	Upregulated
Slc2a3	solute carrier family 2 (facilitated glucose transporter), member 3	Upregulated
Slfn2	schlafen 2	Upregulated
Tpp2	tripeptidyl peptidase II	Upregulated
Lpin2	lipin 2	Upregulated
Mdm1	transformed mouse 3T3 cell double minute 1	Upregulated
4930506M07Rik	RIKEN cDNA 4930506M07 gene	Upregulated
Pip4k2a	phosphatidylinositol-5-phosphate 4-kinase, type II, alpha	Upregulated
Parg	poly (ADP-ribose) glycohydrolase	Upregulated
Steap1	six transmembrane epithelial antigen of the prostate 1	Upregulated
Ddx27	DEAD (Asp-Glu-Ala-Asp) box polypeptide 27	Upregulated
Snx7	sorting nexin 7	Upregulated
Gpr171	G protein-coupled receptor 171	Upregulated
Fxyd5	FXYD domain-containing ion transport regulator 5	Upregulated
Cdt1	chromatin licensing and DNA replication factor 1	Upregulated
Fam84a	family with sequence similarity 84, member A	Upregulated
Has1	hyaluronan synthase1	Upregulated
Mis18bp1	MIS18 binding protein 1	Upregulated
Kdm4c	lysine (K)-specific demethylase 4C	Upregulated
Dusp6	dual specificity phosphatase 6	Upregulated
Ifi27l2b	interferon, alpha-inducible protein 27 like 2B	Upregulated
Gpr34	G protein-coupled receptor 34	Upregulated
Cxcr6	chemokine (C-X-C motif) receptor 6	Upregulated
Myo5a	myosin VA	Upregulated
Kcnab2	potassium voltage-gated channel, shaker-related subfamily, beta member 2	Upregulated
Arl11	ADP-ribosylation factor-like 11	Upregulated
Stx11	syntaxin 11	Upregulated
Elf1	E74-like factor 1	Upregulated
Top2a	topoisomerase (DNA) II alpha	Upregulated
Arap2	ArfGAP with RhoGAP domain, ankyrin repeat and PH domain 2	Upregulated
Gfi1b	growth factor independent 1B	Upregulated
Hsp90b1	heat shock protein 90, beta (Grp94), member 1	Upregulated
Hp	haptoglobin	Upregulated
Coro1a	coronin, actin binding protein 1A	Upregulated
Hltf	helicase-like transcription factor	Upregulated
Cenpk	centromere protein K	Upregulated
D15Ertd621e	DNA segment, Chr 15, ERATO Doi 621, expressed	Upregulated
Map3k8	mitogen-activated protein kinase kinase kinase 8	Upregulated
Icam1	intercellular adhesion molecule 1	Upregulated
Hist1h2ab	histone cluster 1, H2ab	Upregulated
Msh6	mutS homolog 6 (E. coli)	Upregulated
Sdc3	syndecan 3	Upregulated
Bub1b	budding uninhibited by benzimidazoles 1 homolog, beta (S. cerevisiae)	Upregulated
Ccl4	chemokine (C-C motif) ligand 4	Upregulated
Niacr1	niacin receptor 1	Upregulated
Troap	trophinin associated protein	Upregulated
Ifit2	interferon-induced protein with tetratricopeptide repeats 2	Upregulated
Hexb	hexosaminidase B	Upregulated
Zc3h12d	zinc finger CCCH type containing 12D	Upregulated
Tbx21	T-box 21	Upregulated
Cela1	chymotrypsin-like elastase family, member 1	Upregulated
Eme1	essential meiotic endonuclease 1 homolog 1 (S. pombe)	Upregulated
Cstf2	cleavage stimulation factor, 3' pre-RNA subunit 2	Upregulated
Snx10	sorting nexin 10	Upregulated
Zfp784	zinc finger protein 784	Upregulated
Rab8b	RAB8B, member RAS oncogene family	Upregulated
Mitd1	MIT, microtubule interacting and transport, domain containing 1	Upregulated
Apobec1	apolipoprotein B mRNA editing enzyme, catalytic polypeptide 1	Upregulated
Plk4	polo-like kinase 4	Upregulated
Lama3	laminin, alpha 3	Upregulated
Calca	calcitonin/calcitonin-related polypeptide, alpha	Upregulated
Rab37	RAB37, member of RAS oncogene family	Upregulated
Klre1	killer cell lectin-like receptor family E member 1	Upregulated
Pdk3	pyruvate dehydrogenase kinase, isoenzyme 3	Upregulated
Il13ra2	interleukin 13 receptor, alpha 2	Upregulated
Npy	neuropeptide Y	Upregulated
Havcr2	hepatitis A virus cellular receptor 2	Upregulated
1700012B07Rik	RIKEN cDNA 1700012B07 gene	Upregulated
Mlkl	mixed lineage kinase domain-like	Upregulated
Ppp1r15b	protein phosphatase 1, regulatory (inhibitor) subunit 15b	Upregulated
Fpr1	formyl peptide receptor 1	Upregulated
Ankrd1	ankyrin repeat domain 1 (cardiac muscle)	Upregulated
Pdcd1	programmed cell death 1	Upregulated

Bhlhe22	basic helix-loop-helix family, member e22	Upregulated
Wsb1	WD repeat and SOCS box-containing 1	Upregulated
Fasl	Fas ligand (TNF superfamily, member 6)	Upregulated
Dnahc2	dynein, axonemal, heavy chain 2	Upregulated
Slc13a3	solute carrier family 13 (sodium-dependent dicarboxylate transporter), member 3	Upregulated
Calml4	calmodulin-like 4	Upregulated
Camk1d	calcium/calmodulin-dependent protein kinase ID	Upregulated
Eif1a	eukaryotic translation initiation factor 1A	Upregulated
Nedd9	neural precursor cell expressed, developmentally down-regulated gene 9	Upregulated
Tnfaip8l2	tumor necrosis factor, alpha-induced protein 8-like 2	Upregulated
Larp4	La ribonucleoprotein domain family, member 4	Upregulated
Trpv2	transient receptor potential cation channel, subfamily V, member 2	Upregulated
Als2cl	ALS2 C-terminal like	Upregulated
Dhx58	DEXH (Asp-Glu-X-His) box polypeptide 58	Upregulated
Cenph	centromere protein H	Upregulated
Cct6b	chaperonin containing Tcp1, subunit 6b (zeta)	Upregulated
Fgl2	fibrinogen-like protein 2	Upregulated
Zfp367	zinc finger protein 367	Upregulated
1600029D21Rik	RIKEN cDNA 1600029D21 gene	Upregulated
Rhox5	reproductive homeobox 5	Upregulated
Incenp	inner centromere protein	Upregulated
1810011H11Rik	RIKEN cDNA 1810011H11 gene	Upregulated
Arhgef39	Rho guanine nucleotide exchange factor (GEF) 39	Upregulated
Fli1	Friend leukemia integration 1	Upregulated
Tmem178	transmembrane protein 178	Upregulated
Col24a1	collagen, type XXIV, alpha 1	Upregulated
Lcn2	lipocalin 2	Upregulated
Cfp	complement factor properdin	Upregulated
Ednrb	endothelin receptor type B	Upregulated
Lig1	ligase I, DNA, ATP-dependent	Upregulated
Enc1	ectodermal-neural cortex 1	Upregulated
Ccl6	chemokine (C-C motif) ligand 6	Upregulated
Dbf4	DBF4 homolog (S. cerevisiae)	Upregulated
Esm1	endothelial cell-specific molecule 1	Upregulated
Birc3	baculoviral IAP repeat-containing 3	Upregulated
Hnrpll	heterogeneous nuclear ribonucleoprotein L-like	Upregulated
Aldh3a2	aldehyde dehydrogenase family 3, subfamily A2	Upregulated
Slc52a3	solute carrier protein family 52, member 3	Upregulated
Sestd1	SEC14 and spectrin domains 1	Upregulated
Kif20b	kinesin family member 20B	Upregulated
Lyplal1	lysophospholipase-like 1	Upregulated
Myo1g	myosin IG	Upregulated
Lrrc15	leucine rich repeat containing 15	Upregulated
Igfbp3	insulin-like growth factor binding protein 3	Upregulated
Utp15	UTP15, U3 small nucleolar ribonucleoprotein, homolog (yeast)	Upregulated
Tyrobp	TYRO protein tyrosine kinase binding protein	Upregulated
Slc25a17	solute carrier family 25 (mitochondrial carrier, peroxisomal membrane protein), member 17	Upregulated
Lat	linker for activation of T cells	Upregulated
Csgalnact1	chondroitin sulfate N-acetylgalactosaminyltransferase 1	Upregulated
Parp12	poly (ADP-ribose) polymerase family, member 12	Upregulated
Cyp7b1	cytochrome P450, family 7, subfamily b, polypeptide 1	Upregulated
Serpinb8	serine (or cysteine) peptidase inhibitor, clade B, member 8	Upregulated
Gnpda2	glucosamine-6-phosphate deaminase 2	Upregulated
Sectm1a	secreted and transmembrane 1A	Upregulated
Plaur	plasminogen activator, urokinase receptor	Upregulated
Gpr35	G protein-coupled receptor 35	Upregulated
Ssfa2	sperm specific antigen 2	Upregulated
Gmnn	geminin	Upregulated
Mki67	antigen identified by monoclonal antibody Ki 67	Upregulated
Tor4a	torsin family 4, member A	Upregulated
1700012B15Rik	RIKEN cDNA 1700012B15 gene	Upregulated
Gnl3	guanine nucleotide binding protein-like 3 (nucleolar)	Upregulated
Fgr	Gardner-Rasheed feline sarcoma viral (Fgr) oncogene homolog	Upregulated
Inpp5d	inositol polyphosphate-5-phosphatase D	Upregulated
Spata6	spermatogenesis associated 6	Upregulated
Mcm4	minichromosome maintenance deficient 4 homolog (S. cerevisiae)	Upregulated
Sgol2	shugoshin-like 2 (S. pombe)	Upregulated
Slfn1	schlafen 1	Upregulated
Kcnn4	potassium intermediate/small conductance calcium-activated channel, subfamily N, member 4	Upregulated
Csf2ra	colony stimulating factor 2 receptor, alpha, low-affinity (granulocyte-macrophage)	Upregulated
Lctl	lactase-like	Upregulated
Fnbp4	formin binding protein 4	Upregulated
Cdca2	cell division cycle associated 2	Upregulated
Dlgap5	discs, large (Drosophila) homolog-associated protein 5	Upregulated
Ly86	lymphocyte antigen 86	Upregulated
Dctpp1	dCTP pyrophosphatase 1	Upregulated
Rad51c	RAD51 homolog c (S. cerevisiae)	Upregulated
Fpgt	fucose-1-phosphate guanylyltransferase	Upregulated
Lin54	lin-54 homolog (C. elegans)	Upregulated
Ugcg	UDP-glucose ceramide glucosyltransferase	Upregulated
Palb2	partner and localizer of BRCA2	Upregulated
1100001G20Rik	RIKEN cDNA 1100001G20 gene	Upregulated
Fam129b	family with sequence similarity 129, member B	Upregulated
Kctd12b	potassium channel tetramerisation domain containing 12b	Upregulated
Mcm5	minichromosome maintenance deficient 5, cell division cycle 46 (S. cerevisiae)	Upregulated
Capsl	calcyphosine-like	Upregulated
Ripk2	receptor (TNFRSF)-interacting serine-threonine kinase 2	Upregulated
Ccdc66	coiled-coil domain containing 66	Upregulated
Prf1	perforin 1 (pore forming protein)	Upregulated
A230046K03Rik	RIKEN cDNA A230046K03 gene	Upregulated
Ms4a6d	membrane-spanning 4-domains, subfamily A, member 6D	Upregulated
Abcg1	ATP-binding cassette, sub-family G (WHITE), member 1	Upregulated
Samd9l	sterile alpha motif domain containing 9-like	Upregulated
Trnt1	tRNA nucleotidyl transferase, CCA-adding, 1	Upregulated
Parpbp	PARP1 binding protein	Upregulated
Ngfrap1	nerve growth factor receptor (TNFRSF16) associated protein 1	Upregulated
Fam111a	family with sequence similarity 111, member A	Upregulated
Syk	spleen tyrosine kinase	Upregulated

Mefv	Mediterranean fever	Upregulated
Camp	cathelicidin antimicrobial peptide	Upregulated
Mastl	microtubule associated serine/threonine kinase-like	Upregulated
Gpr88	G-protein coupled receptor 88	Upregulated
C1qc	complement component 1, q subcomponent, C chain	Upregulated
Lat2	linker for activation of T cells family, member 2	Upregulated
Cenpn	centromere protein N	Upregulated
Ccl8|LOC100503254	chemokine (C-C motif) ligand 8|c-C motif chemokine 8-like	Upregulated
Slc26a5	solute carrier family 26, member 5	Upregulated
Mcm2	minichromosome maintenance deficient 2 mitotin (S. cerevisiae)	Upregulated
Ccl5	chemokine (C-C motif) ligand 5	Upregulated
Ptbp3	polypyrimidine tract binding protein 3	Upregulated
Osmr	oncostatin M receptor	Upregulated
Siglec1	sialic acid binding Ig-like lectin 1, sialoadhesin	Upregulated
Marco	macrophage receptor with collagenous structure	Upregulated
Ikzf1	IKAROS family zinc finger 1	Upregulated
P4ha3	procollagen-proline, 2-oxoglutarate 4-dioxygenase (proline 4-hydroxylase), alpha polypeptide III	Upregulated
Manba	mannosidase, beta A, lysosomal	Upregulated
Soat1	sterol O-acyltransferase 1	Upregulated
Stat2	signal transducer and activator of transcription 2	Upregulated
Asf1b	ASF1 anti-silencing function 1 homolog B (S. cerevisiae)	Upregulated
BC030867	cDNA sequence BC030867	Upregulated
Tbk1	TANK-binding kinase 1	Upregulated
Skap1	src family associated phosphoprotein 1	Upregulated
Lpxn	leupaxin	Upregulated
Dock8	dedicator of cytokinesis 8	Upregulated
Pfas	phosphoribosylformylglycinamidine synthase (FGAR amidotransferase)	Upregulated
Cd274	CD274 antigen	Upregulated
Ggnbp2	gametogenetin binding protein 2	Upregulated
Snx2	sorting nexin 2	Upregulated
Birc5	baculoviral IAP repeat-containing 5	Upregulated
Clec4d	C-type lectin domain family 4, member d	Upregulated
Ptpn7	protein tyrosine phosphatase, non-receptor type 7	Upregulated
Rad54l	RAD54 like (S. cerevisiae)	Upregulated
Fcgr2b	Fc receptor, IgG, low affinity IIb	Upregulated
Tmem255a	transmembrane protein 255A	Upregulated
Ncapg2	non-SMC condensin II complex, subunit G2	Upregulated
Phf11d	PHD finger protein 11D	Upregulated
Il10rb	interleukin 10 receptor, beta	Upregulated
Ydjc	YdjC homolog (bacterial)	Upregulated
Gmcl1	germ cell-less homolog 1 (Drosophila)	Upregulated
Tmem51	transmembrane protein 51	Upregulated
Pdik1l	PDLIM1 interacting kinase 1 like	Upregulated
Tlr4	toll-like receptor 4	Upregulated
Asah1	N-acylsphingosine amidohydrolase 1	Upregulated
Sfrp1	secreted frizzled-related protein 1	Upregulated
P2ry6	pyrimidinergic receptor P2Y, G-protein coupled, 6	Upregulated
Rnaseh2b	ribonuclease H2, subunit B	Upregulated
D630037F22Rik	RIKEN cDNA D630037F22 gene	Upregulated
Cd69	CD69 antigen	Upregulated
Mthfd1l	methylenetetrahydrofolate dehydrogenase (NADP+ dependent) 1-like	Upregulated
Cotl1	coactosin-like 1 (Dictyostelium)	Upregulated
Cdca7	cell division cycle associated 7	Upregulated
A330021E22Rik	RIKEN cDNA A330021E22 gene	Upregulated
C1qb	complement component 1, q subcomponent, beta polypeptide	Upregulated
Ccdc45	coiled-coil domain containing 45	Upregulated
Pwwp2a	PWWP domain containing 2A	Upregulated
Spib	Spi-B transcription factor (Spi-1/PU.1 related)	Upregulated
Grasp	GRP1 (general receptor for phosphoinositides 1)-associated scaffold protein	Upregulated
Fam64a	family with sequence similarity 64, member A	Upregulated
Il18	interleukin 18	Upregulated
Cept1	choline/ethanolaminephosphotransferase 1	Upregulated
Plk1	polo-like kinase 1	Upregulated
Kif4	kinesin family member 4	Upregulated
Itpr3	inositol 1,4,5-triphosphate receptor 3	Upregulated
Fkbp14	FK506 binding protein 14	Upregulated
Fen1	flap structure specific endonuclease 1	Upregulated
Casp3	caspase 3	Upregulated
S100a9	S100 calcium binding protein A9 (calgranulin B)	Upregulated
Tmem191c	transmembrane protein 191C	Upregulated
Fbxl5	F-box and leucine-rich repeat protein 5	Upregulated
Pla2g1b	phospholipase A2, group IB, pancreas	Upregulated
BC005537	cDNA sequence BC005537	Upregulated
Wdr95	WD40 repeat domain 95	Upregulated
Nxt2	nuclear transport factor 2-like export factor 2	Upregulated
Ms4a6d	membrane-spanning 4-domains, subfamily A, member 6D	Upregulated
AB124611	cDNA sequence AB124611	Upregulated
Padi3	peptidyl arginine deiminase, type III	Upregulated
Cenpa	centromere protein A	Upregulated
Cdca8	cell division cycle associated 8	Upregulated
Arpc3	actin related protein 2/3 complex, subunit 3	Upregulated
Uba7	ubiquitin-like modifier activating enzyme 7	Upregulated
Tnfrsf26	tumor necrosis factor receptor superfamily, member 26	Upregulated
Casp8	caspase 8	Upregulated
Snhg8	small nucleolar RNA host gene 8	Upregulated
Fignl1	fidgetin-like 1	Upregulated
Arhgap4	Rho GTPase activating protein 4	Upregulated
C130073F10Rik	RIKEN cDNA C130073F10 gene	Upregulated
Cenpi	centromere protein I	Upregulated
Gpr176	G protein-coupled receptor 176	Upregulated
Pmaip1	phorbol-12-myristate-13-acetate-induced protein 1	Upregulated
9130024F11Rik	RIKEN cDNA 9130024F11 gene	Upregulated
Fam45a	family with sequence similarity 45, member A	Upregulated
Gas2l3	growth arrest-specific 2 like 3	Upregulated
Lrrc25	leucine rich repeat containing 25	Upregulated
4922501C03Rik	RIKEN cDNA 4922501C03 gene	Upregulated
Tespa1	thymocyte expressed, positive selection associated 1	Upregulated
Dnajc2	DnaJ (Hsp40) homolog, subfamily C, member 2	Upregulated

Ahnak	AHNAK nucleoprotein (desmoyokin)	Upregulated
Exoc3l2	exocyst complex component 3-like 2	Upregulated
Ikbke	inhibitor of kappaB kinase epsilon	Upregulated
Tacc3	transforming, acidic coiled-coil containing protein 3	Upregulated
St14	suppression of tumorigenicity 14 (colon carcinoma)	Upregulated
Cep135	centrosomal protein 135	Upregulated
Syce2	synaptonemal complex central element protein 2	Upregulated
Cfdp1	craniofacial development protein 1	Upregulated
Lst1	leukocyte specific transcript 1	Upregulated
Impact	imprinted and ancient	Upregulated
Zfp62	zinc finger protein 62	Upregulated
Irgm2	immunity-related GTPase family M member 2	Upregulated
Igtp	interferon gamma induced GTPase	Upregulated
Sept11	septin 11	Upregulated
Cstf3	cleavage stimulation factor, 3' pre-RNA, subunit 3	Upregulated
Fyb	FYN binding protein	Upregulated
C6	complement component 6	Upregulated
Tfpi	tissue factor pathway inhibitor	Upregulated
Prelid2	PRELI domain containing 2	Upregulated
Cd109	CD109 antigen	Upregulated
Adam12	a disintegrin and metallopeptidase domain 12 (meltrin alpha)	Upregulated
Tlr13	toll-like receptor 13	Upregulated
Ly6e	lymphocyte antigen 6 complex, locus E	Upregulated
Cdca7l	cell division cycle associated 7 like	Upregulated
5430416N02Rik	RIKEN cDNA 5430416N02 gene	Upregulated
Mcm7	minichromosome maintenance deficient 7 (S. cerevisiae)	Upregulated
Rras2	related RAS viral (r-ras) oncogene homolog 2	Upregulated
Stxbp1	syntaxin binding protein 1	Upregulated
Cd52	CD52 antigen	Upregulated
Cacybp	calcyclin binding protein	Upregulated
Usp6nl	USP6 N-terminal like	Upregulated
Plek	pleckstrin	Upregulated
Clspn	claspin	Upregulated
Rrbp1	ribosome binding protein 1	Upregulated
Myef2	myelin basic protein expression factor 2, repressor	Upregulated
Msh6	mutS homolog 6 (E. coli)	Upregulated
2700029M09Rik	RIKEN cDNA 2700029M09 gene	Upregulated
Tnfsf18	tumor necrosis factor (ligand) superfamily, member 18	Upregulated
Clrn1	clarin 1	Upregulated
Yes1	Yamaguchi sarcoma viral (v-yes) oncogene homolog 1	Upregulated
Ltbp2	latent transforming growth factor beta binding protein 2	Upregulated
Spp1	secreted phosphoprotein 1	Upregulated
Uba6	ubiquitin-like modifier activating enzyme 6	Upregulated
Col24a1	collagen, type XXIV, alpha 1	Upregulated
Tmem74	transmembrane protein 74	Upregulated
Emcn	endomucin	Upregulated
H3f3b	H3 histone, family 3B	Upregulated
Prkx	protein kinase, X-linked	Upregulated
Parp9	poly (ADP-ribose) polymerase family, member 9	Upregulated
Itga6	integrin alpha 6	Upregulated
Slc44a2	solute carrier family 44, member 2	Upregulated
Sema5a	sema domain, seven thrombospondin repeats (type 1 and type 1-like), transmembrane domain	Upregulated
Il7r	interleukin 7 receptor	Upregulated
Cyp1b1	cytochrome P450, family 1, subfamily b, polypeptide 1	Upregulated
Klhl6	kelch-like 6 (Drosophila)	Upregulated
9530003J23Rik	RIKEN cDNA 9530003J23 gene	Upregulated
Nemf	nuclear export mediator factor	Upregulated
Azi2	5-azacytidine induced gene 2	Upregulated
Mrps10	mitochondrial ribosomal protein S10	Upregulated
Anln	anillin, actin binding protein	Upregulated
Depdc1b	DEP domain containing 1B	Upregulated
Lcp2	lymphocyte cytosolic protein 2	Upregulated
Arhgap15	Rho GTPase activating protein 15	Upregulated
Fam105a	family with sequence similarity 105, member A	Upregulated
Cd68	CD68 antigen	Upregulated
Sh3rf1	SH3 domain containing ring finger 1	Upregulated
Zc3hav1	zinc finger CCCH type, antiviral 1	Upregulated
Prpf40a	PRP40 pre-mRNA processing factor 40 homolog A (yeast)	Upregulated
Gpr85	G protein-coupled receptor 85	Upregulated
Evi2a	ecotropic viral integration site 2a	Upregulated
Tmem209	transmembrane protein 209	Upregulated
Diap3	diaphanous homolog 3 (Drosophila)	Upregulated
Sfxn1	sideroflexin 1	Upregulated
Larp4	La ribonucleoprotein domain family, member 4	Upregulated
Taf1d	TATA box binding protein (Tbp)-associated factor, RNA polymerase I, D	Upregulated
Vcam1	vascular cell adhesion molecule 1	Upregulated
Gbp5	guanylate binding protein 5	Upregulated
Mertk	c-mer proto-oncogene tyrosine kinase	Upregulated
4930503L19Rik	RIKEN cDNA 4930503L19 gene	Upregulated
Wasf2	WAS protein family, member 2	Upregulated
Rab8b	RAB8B, member RAS oncogene family	Upregulated
Dyrk3	dual-specificity tyrosine-(Y)-phosphorylation regulated kinase 3	Upregulated
Rps24	ribosomal protein S24	Upregulated
Hiatl1	hippocampus abundant transcript-like 1	Upregulated
Wfdc17|LOC100504934	WAP four-disulfide core domain 17|extracellular peptidase inhibitor-like	Upregulated
Phf11d	PHD finger protein 11D	Upregulated
Zfp455	zinc finger protein 455	Upregulated
Gm9733	predicted gene 9733	Upregulated
A830080D01Rik	RIKEN cDNA A830080D01 gene	Upregulated
Zfp955b	zinc finger protein 955B	Upregulated
Ankle1	ankyrin repeat and LEM domain containing 1	Upregulated
Clec4a3	C-type lectin domain family 4, member a3	Upregulated
4930502E18Rik	RIKEN cDNA 4930502E18 gene	Upregulated
Erich1	glutamate rich 1	Upregulated
Tmem245	transmembrane protein 245	Upregulated
Ly6g6e	lymphocyte antigen 6 complex, locus G6E	Upregulated
Herc6	hect domain and RLD 6	Upregulated
Apon	apolipoprotein N	Upregulated

BC016423	cDNA sequence BC016423	Upregulated
Hiat1	hippocampus abundant gene transcript 1	Upregulated
Tmem106a	transmembrane protein 106A	Upregulated
H2-M1	histocompatibility 2, M region locus 1	Upregulated
Ms4a4c	membrane-spanning 4-domains, subfamily A, member 4C	Upregulated
C330007P06Rik	RIKEN cDNA C330007P06 gene	Upregulated
Zfp948	zinc finger protein 948	Upregulated
Ankrd29	ankyrin repeat domain 29	Upregulated
Mthfd2	methylenetetrahydrofolate dehydrogenase (NAD+ dependent), methenyltetrahydrofolate cycloh	Upregulated
Irf8	interferon regulatory factor 8	Upregulated
Csk	c-src tyrosine kinase	Upregulated
Eif2ak4	eukaryotic translation initiation factor 2 alpha kinase 4	Upregulated
Foxm1	forkhead box M1	Upregulated
Cd55	CD55 antigen	Upregulated
Lox	lysyl oxidase	Upregulated
Ptprj	protein tyrosine phosphatase, receptor type, J	Upregulated
Prkcb	protein kinase C, beta	Upregulated
Skp2	S-phase kinase-associated protein 2 (p45)	Upregulated
Stc1	stanniocalcin 1	Upregulated
Cacna1d	calcium channel, voltage-dependent, L type, alpha 1D subunit	Upregulated
Lrrfip1	leucine rich repeat (in FLII) interacting protein 1	Upregulated
Cenpw	centromere protein W	Upregulated
Brca1	breast cancer 1	Upregulated
Ccr6	chemokine (C-C motif) receptor 6	Upregulated
Dr1	down-regulator of transcription 1	Upregulated
Tap1	transporter 1, ATP-binding cassette, sub-family B (MDR/TAP)	Upregulated
Tpx2	TPX2, microtubule-associated protein homolog (Xenopus laevis)	Upregulated
Hist1h2ao	histone cluster 1, H2ao	Upregulated
Cytip	cytohesin 1 interacting protein	Upregulated
Lpcat1	lysophosphatidylcholine acyltransferase 1	Upregulated
Hpse	heparanase	Upregulated
Rp2h	retinitis pigmentosa 2 homolog (human)	Upregulated
Crk	v-crk sarcoma virus CT10 oncogene homolog (avian)	Upregulated
Fermt3	fermitin family homolog 3 (Drosophila)	Upregulated
Arhgap12	Rho GTPase activating protein 12	Upregulated
Dnmt3l	DNA (cytosine-5-)-methyltransferase 3-like	Upregulated
Ptpre	protein tyrosine phosphatase, receptor type, E	Upregulated
Timp1	tissue inhibitor of metalloproteinase 1	Upregulated
Hsp90aa1|LOC101056618	heat shock protein 90, alpha (cytosolic), class A member 1|uncharacterized LOC101056618	Upregulated
Bbs12	Bardet-Biedl syndrome 12 (human)	Upregulated
Gm885	predicted gene 885	Upregulated
Runx3	runt related transcription factor 3	Upregulated
Batf	basic leucine zipper transcription factor, ATF-like	Upregulated
Tuba1c	tubulin, alpha 1C	Upregulated
Arf6	ADP-ribosylation factor 6	Upregulated
Ccr2	chemokine (C-C motif) receptor 2	Upregulated
Tnfrsf11a	tumor necrosis factor receptor superfamily, member 11a	Upregulated
Il6	interleukin 6	Upregulated
Ncf1	neutrophil cytosolic factor 1	Upregulated
Ifit1	interferon-induced protein with tetratricopeptide repeats 1	Upregulated
Serpine1	serine (or cysteine) peptidase inhibitor, clade E, member 1	Upregulated
Cxcl11	chemokine (C-X-C motif) ligand 11	Upregulated
Pik3cd	phosphatidylinositol 3-kinase catalytic delta polypeptide	Upregulated
Tor3a	torsin family 3, member A	Upregulated
Rad51	RAD51 homolog	Upregulated
Ifi30	interferon gamma inducible protein 30	Upregulated
Rnf128	ring finger protein 128	Upregulated
Zeb2	zinc finger E-box binding homeobox 2	Upregulated
Rbl1	retinoblastoma-like 1 (p107)	Upregulated
Ahr	aryl-hydrocarbon receptor	Upregulated
Ywhaq	tyrosine 3-monooxygenase/tryptophan 5-monooxygenase activation protein, theta polypeptide	Upregulated
Irf7	interferon regulatory factor 7	Upregulated
Lxn	latexin	Upregulated
Gna13	guanine nucleotide binding protein, alpha 13	Upregulated
Ezh2	enhancer of zeste homolog 2 (Drosophila)	Upregulated
Suz12	suppressor of zeste 12 homolog (Drosophila)	Upregulated
Hck	hemopoietic cell kinase	Upregulated
Csf2rb	colony stimulating factor 2 receptor, beta, low-affinity (granulocyte-macrophage)	Upregulated
Rap2c	RAP2C, member of RAS oncogene family	Upregulated
Fmr1	fragile X mental retardation syndrome 1	Upregulated
Hells	helicase, lymphoid specific	Upregulated
Zbp1	Z-DNA binding protein 1	Upregulated
Ascl2	achaete-scute complex homolog 2 (Drosophila)	Upregulated
Nrg1	neuregulin 1	Upregulated
Ets1	E26 avian leukemia oncogene 1, 5' domain	Upregulated
Arg1	arginase, liver	Upregulated
Gzmb	granzyme B	Upregulated
Adam8	a disintegrin and metallopeptidase domain 8	Upregulated
Akr1c13	aldo-keto reductase family 1, member C13	Upregulated
Tnfrsf1b	tumor necrosis factor receptor superfamily, member 1b	Upregulated
Ccnb1ip1	cyclin B1 interacting protein 1	Upregulated
Ptpn6	protein tyrosine phosphatase, non-receptor type 6	Upregulated
Ifih1	interferon induced with helicase C domain 1	Upregulated
Kdm2a	lysine (K)-specific demethylase 2A	Upregulated
Tiam2	T cell lymphoma invasion and metastasis 2	Upregulated
Bsn	bassoon	Upregulated
Gsk3a	glycogen synthase kinase 3 alpha	Upregulated
Ifng	interferon gamma	Upregulated
Sstr5	somatostatin receptor 5	Upregulated
Kitl	kit ligand	Upregulated
Klrb1	killer cell lectin-like receptor subfamily B member 1	Upregulated
Il20rb	interleukin 20 receptor beta	Upregulated
Rsrc2	arginine/serine-rich coiled-coil 2	Upregulated
Rad51d	RAD51 homolog D	Upregulated
Oxsr1	oxidative-stress responsive 1	Upregulated
Six1	sine oculis-related homeobox 1	Upregulated
Lamc2	laminin, gamma 2	Upregulated
Satb2	special AT-rich sequence binding protein 2	Upregulated

Slc11a1	solute carrier family 11 (proton-coupled divalent metal ion transporters), member 1	Upregulated
Axl	AXL receptor tyrosine kinase	Upregulated
Arhgap39	Rho GTPase activating protein 39	Upregulated
Bicc1	bicaudal C homolog 1 (Drosophila)	Upregulated
Cd40	CD40 antigen	Upregulated
Tmem90b	transmembrane protein 90B	Upregulated
Gmps	guanine monophosphate synthetase	Upregulated
Gm17365	predicted gene, 17365	Upregulated
Nrp1	neuropilin 1	Upregulated
Runx1	runt related transcription factor 1	Upregulated
Lipg	lipase, endothelial	Upregulated
Ltb	lymphotoxin B	Upregulated
B2m	beta-2 microglobulin	Upregulated
Ripk3	receptor-interacting serine-threonine kinase 3	Upregulated
Slc25a13	solute carrier family 25 (mitochondrial carrier, adenine nucleotide translocator), member 13	Upregulated
Ctbp2	C-terminal binding protein 2	Upregulated
Dlx2	distal-less homeobox 2	Upregulated
Msr1	macrophage scavenger receptor 1	Upregulated
Pla2g4a	phospholipase A2, group IVA (cytosolic, calcium-dependent)	Upregulated
Ccnb2	cyclin B2	Upregulated
Myc	myelocytomatosis oncogene	Upregulated
Ccna2	cyclin A2	Upregulated
Ccr1	chemokine (C-C motif) receptor 1	Upregulated
Tmpo	thymopoietin	Upregulated
Zfp81	zinc finger protein 81	Upregulated
Gins2	GINS complex subunit 2 (Psf2 homolog)	Upregulated
Cdkn3	cyclin-dependent kinase inhibitor 3	Upregulated
5031414D18Rik	RIKEN cDNA 5031414D18 gene	Upregulated
Fam167b	family with sequence similarity 167, member B	Upregulated
2810474O19Rik	RIKEN cDNA 2810474O19 gene	Upregulated
Gm4951	predicted gene 4951	Upregulated
Cox7b2	cytochrome c oxidase subunit VIIb2	Upregulated
Pet2	plasmacytoma expressed transcript 2	Upregulated
AU041133	expressed sequence AU041133	Upregulated
Apol10a	apolipoprotein L 10A	Upregulated
Atp8b4	ATPase, class I, type 8B, member 4	Upregulated
Tmem171	transmembrane protein 171	Upregulated
Gm5431	predicted gene 5431	Upregulated
Cd2ap	CD2-associated protein	Upregulated
Gnai3	guanine nucleotide binding protein (G protein), alpha inhibiting 3	Upregulated
Nlrp1b	NLR family, pyrin domain containing 1B	Upregulated
Podnl1	podocan-like 1	Upregulated
Vsig8	V-set and immunoglobulin domain containing 8	Upregulated
A030009H04Rik	RIKEN cDNA A030009H04 gene	Upregulated
4632434I11Rik	RIKEN cDNA 4632434I11 gene	Upregulated
Galnt9	UDP-N-acetyl-alpha-D-galactosamine:polypeptide N-acetylgalactosaminyltransferase 9	Upregulated
Serpinb9	serine (or cysteine) peptidase inhibitor, clade B, member 9	Upregulated
Zfp961	zinc finger protein 961	Upregulated
Slc4a5	solute carrier family 4, sodium bicarbonate cotransporter, member 5	Upregulated
Tm6sf1	transmembrane 6 superfamily member 1	Upregulated
Slfn4	schlafen 4	Upregulated
Tmem184c	transmembrane protein 184C	Upregulated
Scarf1	scavenger receptor class F, member 1	Upregulated
AU018091	expressed sequence AU018091	Upregulated
Zfp69	zinc finger protein 69	Upregulated
Edem1	ER degradation enhancer, mannosidase alpha-like 1	Upregulated
Vamp7	vesicle-associated membrane protein 7	Upregulated
Serpinb9b	serine (or cysteine) peptidase inhibitor, clade B, member 9b	Upregulated
Nfxl1	nuclear transcription factor, X-box binding-like 1	Upregulated
Gm12250	predicted gene 12250	Upregulated
Rasa1	RAS p21 protein activator 1	Upregulated
Pik3c2a	phosphatidylinositol 3-kinase, C2 domain containing, alpha polypeptide	Upregulated
Ppwd1	peptidylprolyl isomerase domain and WD repeat containing 1	Upregulated
Ctse	cathepsin E	Upregulated
Rgs18	regulator of G-protein signaling 18	Upregulated
Sri	sorcin	Upregulated
Clk1	CDC-like kinase 1	Upregulated
Phf6	PHD finger protein 6	Upregulated
Gm6377	predicted gene 6377	Upregulated
Ifi203	interferon activated gene 203	Upregulated
Orc1	origin recognition complex, subunit 1	Upregulated
Ccl9	chemokine (C-C motif) ligand 9	Upregulated
Cyyr1	cysteine and tyrosine-rich protein 1	Upregulated
Far1	fatty acyl CoA reductase 1	Upregulated
Zc3h14	zinc finger CCCH type containing 14	Upregulated
Actr2	ARP2 actin-related protein 2	Upregulated
Cdc6	cell division cycle 6	Upregulated
Dtl	denticleless homolog (Drosophila)	Upregulated
Pnma5	paraneoplastic antigen family 5	Upregulated
Vmp1	vacuole membrane protein 1	Upregulated
Tyw5	tRNA-yW synthesizing protein 5	Upregulated
Jrkl	jerky homolog-like (mouse)	Upregulated
Anxa3	annexin A3	Upregulated
Dzip3	DAZ interacting protein 3, zinc finger	Upregulated
Vps4b	vacuolar protein sorting 4b (yeast)	Upregulated
Cdc40	cell division cycle 40	Upregulated
Pik3r5	phosphoinositide-3-kinase, regulatory subunit 5, p101	Upregulated
Ptpn22	protein tyrosine phosphatase, non-receptor type 22 (lymphoid)	Upregulated
Cd200r1	CD200 receptor 1	Upregulated
Ap1s3	adaptor-related protein complex AP-1, sigma 3	Upregulated
Taf1b	TATA box binding protein (Tbp)-associated factor, RNA polymerase I, B	Upregulated
Hist1h2ag	histone cluster 1, H2ag	Upregulated
Pkp3	plakophilin 3	Upregulated
Slc39a12	solute carrier family 39 (zinc transporter), member 12	Upregulated
Arhgap25	Rho GTPase activating protein 25	Upregulated
Depdc1a	DEP domain containing 1a	Upregulated
Atad5	ATPase family, AAA domain containing 5	Upregulated
Serpina3g	serine (or cysteine) peptidase inhibitor, clade A, member 3G	Upregulated

Gng2	guanine nucleotide binding protein (G protein), gamma 2	Upregulated
Ctla2b	cytotoxic T lymphocyte-associated protein 2 beta	Upregulated
Pydc3	pyrin domain containing 3	Upregulated
Chtf18	CTF18, chromosome transmission fidelity factor 18	Upregulated
Wdr43	WD repeat domain 43	Upregulated
Zxdb	zinc finger, X-linked, duplicated B	Upregulated
Dtx3l	deltex 3-like (Drosophila)	Upregulated
Pole2	polymerase (DNA directed), epsilon 2 (p59 subunit)	Upregulated
Trim59	tripartite motif-containing 59	Upregulated
Riok2	RIO kinase 2 (yeast)	Upregulated
Dhx9	DEAH (Asp-Glu-Ala-His) box polypeptide 9	Upregulated
Scimp	SLP adaptor and CSK interacting membrane protein	Upregulated
Mgat2	mannoside acetylglucosaminyltransferase 2	Upregulated
Actn1	actinin, alpha 1	Upregulated
Ddx21	DEAD (Asp-Glu-Ala-Asp) box polypeptide 21	Upregulated
Arhgap30	Rho GTPase activating protein 30	Upregulated
Serpina3m	serine (or cysteine) peptidase inhibitor, clade A, member 3M	Upregulated
Twf1	twinfilin, actin-binding protein, homolog 1 (Drosophila)	Upregulated
Mt4	metallothionein 4	Upregulated
Ccbe1	collagen and calcium binding EGF domains 1	Upregulated
Topbp1	topoisomerase (DNA) II binding protein 1	Upregulated
Pyhin1	pyrin and HIN domain family, member 1	Upregulated
E2f8	E2F transcription factor 8	Upregulated
Pvrl4	poliovirus receptor-related 4	Upregulated
Rbm25	RNA binding motif protein 25	Upregulated
Ptprcap	protein tyrosine phosphatase, receptor type, C polypeptide-associated protein	Upregulated
Sass6	spindle assembly 6 homolog (C. elegans)	Upregulated
Hist2h2bb	histone cluster 2, H2bb	Upregulated
Mdc1	mediator of DNA damage checkpoint 1	Upregulated
Nek2	NIMA (never in mitosis gene a)-related expressed kinase 2	Upregulated
Taf1d	TATA box binding protein (Tbp)-associated factor, RNA polymerase I, D	Upregulated
Cttnbp2nl	CTTNBP2 N-terminal like	Upregulated
Hecw2	HECT, C2 and WW domain containing E3 ubiquitin protein ligase 2	Upregulated
Hnrnpm	heterogeneous nuclear ribonucleoprotein M	Upregulated
Dpep2	dipeptidase 2	Upregulated
Arhgap11a	Rho GTPase activating protein 11A	Upregulated
Tmem252	transmembrane protein 252	Upregulated
Bard1	BRCA1 associated RING domain 1	Upregulated
Klhl2	kelch-like 2, Mayven (Drosophila)	Upregulated
Mospd2	motile sperm domain containing 2	Upregulated
March1	membrane-associated ring finger (C3HC4) 1	Upregulated
Fubp1	far upstream element (FUSE) binding protein 1	Upregulated
Cep110	centrosomal protein 110	Upregulated
Map4k1	mitogen-activated protein kinase kinase kinase kinase 1	Upregulated
Amica1	adhesion molecule, interacts with CXADR antigen 1	Upregulated
Myef2	myelin basic protein expression factor 2, repressor	Upregulated
Zdhhc20	zinc finger, DHHC domain containing 20	Upregulated
Serpina3f	serine (or cysteine) peptidase inhibitor, clade A, member 3F	Upregulated
Trim34a	tripartite motif-containing 34A	Upregulated
Acer3	alkaline ceramidase 3	Upregulated
Nanos1	nanos homolog 1 (Drosophila)	Upregulated
Serp1	stress-associated endoplasmic reticulum protein 1	Upregulated
Cd5	CD5 antigen	Upregulated
Ska3	spindle and kinetochore associated complex subunit 3	Upregulated
Odf2l	outer dense fiber of sperm tails 2-like	Upregulated
Cyfip1	cytoplasmic FMR1 interacting protein 1	Upregulated
Gtsf1	gametocyte specific factor 1	Upregulated
Epsti1	epithelial stromal interaction 1 (breast)	Upregulated
Fndc3a	fibronectin type III domain containing 3A	Upregulated
Rab20	RAB20, member RAS oncogene family	Upregulated
Hist1h3g	histone cluster 1, H3g	Upregulated
Acsl5	acyl-CoA synthetase long-chain family member 5	Upregulated
Rfc5	replication factor C (activator 1) 5	Upregulated
Rps6ka1	ribosomal protein S6 kinase polypeptide 1	Upregulated
Rbm27	RNA binding motif protein 27	Upregulated
Ifi44	interferon-induced protein 44	Upregulated
Ankrd32	ankyrin repeat domain 32	Upregulated
Rnf149	ring finger protein 149	Upregulated
Serinc3	serine incorporator 3	Upregulated
Ctla2a	cytotoxic T lymphocyte-associated protein 2 alpha	Upregulated
Procr	protein C receptor, endothelial	Upregulated
Upp1	uridine phosphorylase 1	Upregulated
Pgam1	phosphoglycerate mutase 1	Upregulated
Samhd1	SAM domain and HD domain, 1	Upregulated
Sec1	secretory blood group 1	Upregulated
Rbm3	RNA binding motif protein 3	Upregulated
Actr3	ARP3 actin-related protein 3	Upregulated
Dppa4	developmental pluripotency associated 4	Upregulated
Chd1	chromodomain helicase DNA binding protein 1	Upregulated
Slc7a1	solute carrier family 7 (cationic amino acid transporter, y+ system), member 1	Upregulated
Dusp2	dual specificity phosphatase 2	Upregulated
Rai14	retinoic acid induced 14	Upregulated
Cdc14b	CDC14 cell division cycle 14B	Upregulated
BC147527	cDNA sequence BC147527	Upregulated
Spdl1	spindle apparatus coiled-coil protein 1	Upregulated
I830077J02Rik	RIKEN cDNA I830077J02 gene	Upregulated
Tbc1d8b	TBC1 domain family, member 8B	Upregulated
Lrrc4	leucine rich repeat containing 4	Upregulated
Atp11c	ATPase, class VI, type 11C	Upregulated
Jmjd1c	jumonji domain containing 1C	Upregulated
Psme1	proteasome (prosome, macropain) 28 subunit, alpha	Upregulated
Nup54	nucleoporin 54	Upregulated
Aldh9a1	aldehyde dehydrogenase 9, subfamily A1	Upregulated
Pi4k2b	phosphatidylinositol 4-kinase type 2 beta	Upregulated
Wfdc18	WAP four-disulfide core domain 18	Upregulated
Tlr12	toll-like receptor 12	Upregulated
Phldb2	pleckstrin homology-like domain, family B, member 2	Upregulated
Esco2	establishment of cohesion 1 homolog 2 (S. cerevisiae)	Upregulated

Smek1	SMEK homolog 1, suppressor of mek1 (Dictyostelium)	Upregulated
Slc5a3	solute carrier family 5 (inositol transporters), member 3	Upregulated
Tbc1d10c	TBC1 domain family, member 10c	Upregulated
Il18r1	interleukin 18 receptor 1	Upregulated
Cpt1a	carnitine palmitoyltransferase 1a, liver	Upregulated
Eif5	eukaryotic translation initiation factor 5	Upregulated
Gimap4	GTPase, IMAP family member 4	Upregulated
Ppip5k2	diphosphoinositol pentakisphosphate kinase 2	Upregulated
Rrm2	ribonucleotide reductase M2	Upregulated
C330027C09Rik	RIKEN cDNA C330027C09 gene	Upregulated
Clec9a	C-type lectin domain family 9, member a	Upregulated
BC003331	cDNA sequence BC003331	Upregulated
Loxl4	lysyl oxidase-like 4	Upregulated
Cd180	CD180 antigen	Upregulated
Zcwpw1	zinc finger, CW type with PWWP domain 1	Upregulated
Pion	pigeon homolog (Drosophila)	Upregulated
Ms4a6c	membrane-spanning 4-domains, subfamily A, member 6C	Upregulated
Tubgcp5	tubulin, gamma complex associated protein 5	Upregulated
Rnasel	ribonuclease L (2', 5'-oligoisoadenylate synthetase-dependent)	Upregulated
Top1	topoisomerase (DNA) I	Upregulated
Usp45	ubiquitin specific petidase 45	Upregulated
Gimap8	GTPase, IMAP family member 8	Upregulated
Slc16a7	solute carrier family 16 (monocarboxylic acid transporters), member 7	Upregulated
S100a4	S100 calcium binding protein A4	Upregulated
Cdc25c	cell division cycle 25C	Upregulated
Serpinb1b	serine (or cysteine) peptidase inhibitor, clade B, member 1b	Upregulated
Txk	TXK tyrosine kinase	Upregulated
Suv39h2	suppressor of variegation 3-9 homolog 2 (Drosophila)	Upregulated
Pdcl	phosducin-like	Upregulated
Hmmr	hyaluronan mediated motility receptor (RHAMM)	Upregulated
Naaa	N-acylethanolamine acid amidase	Upregulated
Sco1	SCO cytochrome oxidase deficient homolog 1 (yeast)	Upregulated
Gpsm2	G-protein signalling modulator 2 (AGS3-like, C. elegans)	Upregulated
Eif3e	eukaryotic translation initiation factor 3, subunit E	Upregulated
Rinl	Ras and Rab interactor-like	Upregulated
Smek2	SMEK homolog 2, suppressor of mek1 (Dictyostelium)	Upregulated
Sit1	suppression inducing transmembrane adaptor 1	Upregulated
Hdac9	histone deacetylase 9	Upregulated
Cav2	caveolin 2	Upregulated
Reep4	receptor accessory protein 4	Upregulated
Ccnf	cyclin F	Upregulated
Gm4841	predicted gene 4841	Upregulated
Crim1	cysteine rich transmembrane BMP regulator 1 (chordin like)	Upregulated
Ero1l	ERO1-like (S. cerevisiae)	Upregulated
Rab19	RAB19, member RAS oncogene family	Upregulated
Rbm7	RNA binding motif protein 7	Upregulated
Pbk	PDZ binding kinase	Upregulated
Hmgn5	high-mobility group nucleosome binding domain 5	Upregulated
Terf1	telomeric repeat binding factor 1	Upregulated
Dock4	dedicator of cytokinesis 4	Upregulated
Unc93a	unc-93 homolog A (C. elegans)	Upregulated
Dna2	DNA replication helicase 2 homolog (yeast)	Upregulated
C3ar1	complement component 3a receptor 1	Upregulated
AW112010	expressed sequence AW112010	Upregulated
Ankrd44	ankyrin repeat domain 44	Upregulated
Chl1	cell adhesion molecule with homology to L1CAM	Upregulated
Gm15056	predicted gene 15056	Upregulated
Dnttip2	deoxynucleotidyltransferase, terminal, interacting protein 2	Upregulated
Itgad	integrin, alpha D	Upregulated
Pydc4	pyrin domain containing 4	Upregulated
Serpina3h|Serpina3i	serine (or cysteine) peptidase inhibitor, clade A, member 3H|serine (or cysteine) peptidase inhib	Upregulated
Cd84	CD84 antigen	Upregulated
Fancb	Fanconi anemia, complementation group B	Upregulated
Spred1	sprouty protein with EVH-1 domain 1, related sequence	Upregulated
Pgm1	phosphoglucomutase 1	Upregulated
Mtss1	metastasis suppressor 1	Upregulated
Ccdc68	coiled-coil domain containing 68	Upregulated
Kif18a	kinesin family member 18A	Upregulated
Ascc3	activating signal cointegrator 1 complex subunit 3	Upregulated
Acsbg1	acyl-CoA synthetase bubblegum family member 1	Upregulated
Skap2	src family associated phosphoprotein 2	Upregulated
Wwc1	WW, C2 and coiled-coil domain containing 1	Upregulated
Apoc2	apolipoprotein C-II	Upregulated
Acap1	ArfGAP with coiled-coil, ankyrin repeat and PH domains 1	Upregulated
Slc25a43	solute carrier family 25, member 43	Upregulated
Hpgds	hematopoietic prostaglandin D synthase	Upregulated
Ccdc104	coiled-coil domain containing 104	Upregulated
Dse	dermatan sulfate epimerase	Upregulated
Prrc2c	proline-rich coiled-coil 2C	Upregulated
Haus3	HAUS augmin-like complex, subunit 3	Upregulated
Snx20	sorting nexin 20	Upregulated
Itpka	inositol 1,4,5-trisphosphate 3-kinase A	Upregulated
Papd4	PAP associated domain containing 4	Upregulated
Npl	N-acetylneuraminate pyruvate lyase	Upregulated
Hk3	hexokinase 3	Upregulated
Adss	adenylosuccinate synthetase, non muscle	Upregulated
Efcab7	EF-hand calcium binding domain 7	Upregulated
Zbtb1	zinc finger and BTB domain containing 1	Upregulated
Cops2	COP9 (constitutive photomorphogenic) homolog, subunit 2 (Arabidopsis thaliana)	Upregulated
Cd2	CD2 antigen	Upregulated
Nfkbie	nuclear factor of kappa light polypeptide gene enhancer in B cells inhibitor, epsilon	Upregulated
Wfdc2	WAP four-disulfide core domain 2	Upregulated
Susd3	sushi domain containing 3	Upregulated
Pik3ap1	phosphoinositide-3-kinase adaptor protein 1	Upregulated
Iigp1	interferon inducible GTPase 1	Upregulated
Rap1a	RAS-related protein-1a	Upregulated
Armc3	armadillo repeat containing 3	Upregulated
Scfd1	Sec1 family domain containing 1	Upregulated

Il22ra2	interleukin 22 receptor, alpha 2	Upregulated
Gpr174	G protein-coupled receptor 174	Upregulated
Bend4	BEN domain containing 4	Upregulated
Tex9	testis expressed gene 9	Upregulated
Zdhhc2	zinc finger, DHHC domain containing 2	Upregulated
Kif11	kinesin family member 11	Upregulated
Ogfrl1	opioid growth factor receptor-like 1	Upregulated
Depdc7	DEP domain containing 7	Upregulated
Fanca	Fanconi anemia, complementation group A	Upregulated
Tnfaip8	tumor necrosis factor, alpha-induced protein 8	Upregulated
B3gnt2	UDP-GlcNAc:betaGal beta-1,3-N-acetylglucosaminyltransferase 2	Upregulated
F13a1	coagulation factor XIII, A1 subunit	Upregulated
Nlrc5	NLR family, CARD domain containing 5	Upregulated
Tmem2	transmembrane protein 2	Upregulated
Ms4a1	membrane-spanning 4-domains, subfamily A, member 1	Upregulated
Fblim1	filamin binding LIM protein 1	Upregulated
Gatm	glycine amidinotransferase (L-arginine:glycine amidinotransferase)	Upregulated
Shcbp1	Shc SH2-domain binding protein 1	Upregulated
Zfp7	zinc finger protein 7	Upregulated
Tes	testis derived transcript	Upregulated
Neil3	nei like 3 (E. coli)	Upregulated
Atad2	ATPase family, AAA domain containing 2	Upregulated
Zfp280c	zinc finger protein 280C	Upregulated
Tra2b	transformer 2 beta homolog (Drosophila)	Upregulated
Zfp809	zinc finger protein 809	Upregulated
Bcl3	B cell leukemia/lymphoma 3	Upregulated
Efhd2	EF hand domain containing 2	Upregulated
Zfp445	zinc finger protein 445	Upregulated
Fancd2	Fanconi anemia, complementation group D2	Upregulated
Mad2l1	MAD2 mitotic arrest deficient-like 1	Upregulated
Zmynd15	zinc finger, MYND-type containing 15	Upregulated
AA467197	expressed sequence AA467197	Upregulated
Dusp9	dual specificity phosphatase 9	Upregulated
Ms4a7	membrane-spanning 4-domains, subfamily A, member 7	Upregulated
Krt20	keratin 20	Upregulated
Espl1	extra spindle poles-like 1 (S. cerevisiae)	Upregulated
Vsig4	V-set and immunoglobulin domain containing 4	Upregulated
Mex3a	mex3 homolog A (C. elegans)	Upregulated
G2e3	G2/M-phase specific E3 ubiquitin ligase	Upregulated
Csf2rb2	colony stimulating factor 2 receptor, beta 2, low-affinity (granulocyte-macrophage)	Upregulated
Klk10	kallikrein related-peptidase 10	Upregulated
Slc7a7	solute carrier family 7 (cationic amino acid transporter, y+ system), member 7	Upregulated
Pggt1b	protein geranylgeranyltransferase type I, beta subunit	Upregulated
Spc25	SPC25, NDC80 kinetochore complex component, homolog (S. cerevisiae)	Upregulated
Lrr1	leucine rich repeat protein 1	Upregulated
Ect2	ect2 oncogene	Upregulated
Ctla2b	cytotoxic T lymphocyte-associated protein 2 beta	Upregulated
Gmfg	glia maturation factor, gamma	Upregulated
Ttk	Ttk protein kinase	Upregulated
Eif4a2	eukaryotic translation initiation factor 4A2	Upregulated
Lrrk2	leucine-rich repeat kinase 2	Upregulated
Nol8	nucleolar protein 8	Upregulated
Mcm10	minichromosome maintenance deficient 10 (S. cerevisiae)	Upregulated
Igf2bp3	insulin-like growth factor 2 mRNA binding protein 3	Upregulated
Cyth4	cytohesin 4	Upregulated
Ropn1l	ropporin 1-like	Upregulated
P2ry12	purinergic receptor P2Y, G-protein coupled 12	Upregulated
Emr4	EGF-like module containing, mucin-like, hormone receptor-like sequence 4	Upregulated
Tex30	testis expressed 30	Upregulated
Kif23	kinesin family member 23	Upregulated
Ifi47	interferon gamma inducible protein 47	Upregulated
Arpc5	actin related protein 2/3 complex, subunit 5	Upregulated
Mis18a	MIS18 kinetochore protein homolog A (S. pombe)	Upregulated
Trem3	triggering receptor expressed on myeloid cells 3	Upregulated
Clstn3	calsyntenin 3	Upregulated
E230025N22Rik	Riken cDNA E230025N22 gene	Upregulated
Htra4	HtrA serine peptidase 4	Upregulated
1700097N02Rik	RIKEN cDNA 1700097N02 gene	Upregulated
Gimap9	GTPase, IMAP family member 9	Upregulated
Tecpr2	tectonin beta-propeller repeat containing 2	Upregulated
Nusap1	nucleolar and spindle associated protein 1	Upregulated
Abracl	ABRA C-terminal like	Upregulated
2310008H04Rik	RIKEN cDNA 2310008H04 gene	Upregulated
3830406C13Rik	RIKEN cDNA 3830406C13 gene	Upregulated
Slamf9	SLAM family member 9	Upregulated
Mb21d1	Mab-21 domain containing 1	Upregulated
Fam107b	family with sequence similarity 107, member B	Upregulated
Gsg2	germ cell-specific gene 2	Upregulated
Cd6	CD6 antigen	Upregulated
Myo1f	myosin IF	Upregulated
Col8a1	collagen, type VIII, alpha 1	Upregulated
Ccdc112	coiled-coil domain containing 112	Upregulated
Map3k15	mitogen-activated protein kinase kinase kinase 15	Upregulated
Psma8	proteasome (prosome, macropain) subunit, alpha type, 8	Upregulated
Mpeg1	macrophage expressed gene 1	Upregulated
Krtap8-1	keratin associated protein 8-1	Upregulated
Mis12	MIS12 homolog (yeast)	Upregulated
Wdr76	WD repeat domain 76	Upregulated
Aspm	asp (abnormal spindle)-like, microcephaly associated (Drosophila)	Upregulated
Wdr60	WD repeat domain 60	Upregulated
Cenpj	centromere protein J	Upregulated
Tmem8	transmembrane protein 8 (five membrane-spanning domains)	Upregulated
Zfp365	zinc finger protein 365	Upregulated
Nmi	N-myc (and STAT) interactor	Upregulated
Rnase6	ribonuclease, RNase A family, 6	Upregulated
Gch1	GTP cyclohydrolase 1	Upregulated
Fnbp1l	formin binding protein 1-like	Upregulated
Psat1	phosphoserine aminotransferase 1	Upregulated

Pls3	plastin 3 (T-isoform)	Upregulated
Tra2a	transformer 2 alpha homolog (Drosophila)	Upregulated
Mcm6	minichromosome maintenance deficient 6 (MIS5 homolog, S. pombe) (S. cerevisiae)	Upregulated
Slc25a32	solute carrier family 25, member 32	Upregulated
Cenpq	centromere protein Q	Upregulated
AF251705	cDNA sequence AF251705	Upregulated
Ncf4	neutrophil cytosolic factor 4	Upregulated
Kctd12	potassium channel tetramerisation domain containing 12	Upregulated
Pqlc3	PQ loop repeat containing	Upregulated
Dennd2d	DENN/MADD domain containing 2D	Upregulated
Cep170	centrosomal protein 170	Upregulated
Cd3g	CD3 antigen, gamma polypeptide	Upregulated
Cd53	CD53 antigen	Upregulated
Aif1	allograft inflammatory factor 1	Upregulated
Sdcbp	syndecan binding protein	Upregulated
Ctdspl2	CTD (carboxy-terminal domain, RNA polymerase II, polypeptide A) small phosphatase like 2	Upregulated
Srgn	serglycin	Upregulated
Plin2	perilipin 2	Upregulated
9930111J21Rik2	RIKEN cDNA 9930111J21 gene 2	Upregulated
Wee1	WEE 1 homolog 1 (S. pombe)	Upregulated
Pola1	polymerase (DNA directed), alpha 1	Upregulated
Chml	choroideremia-like	Upregulated
Themis2	thymocyte selection associated family member 2	Upregulated
Nop58	NOP58 ribonucleoprotein	Upregulated
Litaf	LPS-induced TN factor	Upregulated
Rtp4	receptor transporter protein 4	Upregulated
Irg1	immunoresponsive gene 1	Upregulated
Tspan32	tetraspanin 32	Upregulated
Lair1	leukocyte-associated Ig-like receptor 1	Upregulated
Cd72	CD72 antigen	Upregulated
Lancl3	LanC lantibiotic synthetase component C-like 3 (bacterial)	Upregulated
Glipr1	GLI pathogenesis-related 1 (glioma)	Upregulated
Ada	adenosine deaminase	Upregulated
Kmo	kynurenine 3-monooxygenase (kynurenine 3-hydroxylase)	Upregulated
F2rl1	coagulation factor II (thrombin) receptor-like 1	Upregulated
Lpcat3	lysophosphatidylcholine acyltransferase 3	Upregulated
Sgms2	sphingomyelin synthase 2	Upregulated
Agfg1	ArfGAP with FG repeats 1	Upregulated
Rxfp3	relaxin family peptide receptor 3	Upregulated
Malt1	mucosa associated lymphoid tissue lymphoma translocation gene 1	Upregulated
Cetn3	centrin 3	Upregulated
P2ry10	purinergic receptor P2Y, G-protein coupled 10	Upregulated
Chaf1b	chromatin assembly factor 1, subunit B (p60)	Upregulated
Lrrc8c	leucine rich repeat containing 8 family, member C	Upregulated
Nostrin	nitric oxide synthase trafficker	Upregulated
Slc2a6	solute carrier family 2 (facilitated glucose transporter), member 6	Upregulated
Rictor	RPTOR independent companion of MTOR, complex 2	Upregulated
Dppa2	developmental pluripotency associated 2	Upregulated
Adm	adrenomedullin	Upregulated
Gpr18	G protein-coupled receptor 18	Upregulated
Plac8	placenta-specific 8	Upregulated
Apaf1	apoptotic peptidase activating factor 1	Upregulated
Fcgr1	Fc receptor, IgG, high affinity I	Upregulated
Prim1	DNA primase, p49 subunit	Upregulated
Ghsr	growth hormone secretagogue receptor	Upregulated
Slc24a5	solute carrier family 24, member 5	Upregulated
Gorab	golgin, RAB6-interacting	Upregulated
Clic1	chloride intracellular channel 1	Upregulated
Krt75	keratin 75	Upregulated
Dek	DEK oncogene (DNA binding)	Upregulated
Gje1	gap junction protein, epsilon 1	Upregulated
Kif15	kinesin family member 15	Upregulated
Ncaph	non-SMC condensin I complex, subunit H	Upregulated
Bzw1	basic leucine zipper and W2 domains 1	Upregulated
Hspa4l	heat shock protein 4 like	Upregulated
Gbp2	guanylate binding protein 2	Upregulated
Donson	downstream neighbor of SON	Upregulated
Tpm3	tropomyosin 3, gamma	Upregulated
Nup160	nucleoporin 160	Upregulated
Myh9	myosin, heavy polypeptide 9, non-muscle	Upregulated
Rasl11a	RAS-like, family 11, member A	Upregulated
Clec10a	C-type lectin domain family 10, member A	Upregulated
Ctsc	cathepsin C	Upregulated
Cep70	centrosomal protein 70	Upregulated
Dab2	disabled 2, mitogen-responsive phosphoprotein	Upregulated
Prim2	DNA primase, p58 subunit	Upregulated
Chaf1a	chromatin assembly factor 1, subunit A (p150)	Upregulated
Eif2s3y	eukaryotic translation initiation factor 2, subunit 3, structural gene Y-linked	Upregulated
Spic	Spi-C transcription factor (Spi-1/PU.1 related)	Upregulated
Sh3bgrl	SH3-binding domain glutamic acid-rich protein like	Upregulated
Slain1	SLAIN motif family, member 1	Upregulated
Pttg1	pituitary tumor-transforming gene 1	Upregulated
Plek2	pleckstrin 2	Upregulated
Cxcl1	chemokine (C-X-C motif) ligand 1	Upregulated
Il18bp	interleukin 18 binding protein	Upregulated
Lect2	leukocyte cell-derived chemotaxin 2	Upregulated
Batf2	basic leucine zipper transcription factor, ATF-like 2	Upregulated
Picalm	phosphatidylinositol binding clathrin assembly protein	Upregulated
Ly96	lymphocyte antigen 96	Upregulated
Smc4	structural maintenance of chromosomes 4	Upregulated
Itch	itchy, E3 ubiquitin protein ligase	Upregulated
Prdm1	PR domain containing 1, with ZNF domain	Upregulated
Ptgs2	prostaglandin-endoperoxide synthase 2	Upregulated
Areg	amphiregulin	Upregulated
Stk39	serine/threonine kinase 39	Upregulated
Plcg2	phospholipase C, gamma 2	Upregulated
Ccr5	chemokine (C-C motif) receptor 5	Upregulated
H2-M3	histocompatibility 2, M region locus 3	Upregulated

Stat1	signal transducer and activator of transcription 1	Upregulated
Itgam	integrin alpha M	Upregulated
Krtap26-1	keratin associated protein 26-1	Upregulated
Fpr2	formyl peptide receptor 2	Upregulated
Ch25h	cholesterol 25-hydroxylase	Upregulated
Tas2r136	taste receptor, type 2, member 136	Upregulated
Itm2b	integral membrane protein 2B	Upregulated
Myd88	myeloid differentiation primary response gene 88	Upregulated
Hdc	histidine decarboxylase	Upregulated
Alox5ap	arachidonate 5-lipoxygenase activating protein	Upregulated
Abi1	abl-interactor 1	Upregulated
Pms1	postmeiotic segregation increased 1 (S. cerevisiae)	Upregulated
Cxcl2	chemokine (C-X-C motif) ligand 2	Upregulated
Adam10	a disintegrin and metallopeptidase domain 10	Upregulated
Parp14	poly (ADP-ribose) polymerase family, member 14	Upregulated
Fcgr3	Fc receptor, IgG, low affinity III	Upregulated
Arpc1b	actin related protein 2/3 complex, subunit 1B	Upregulated
Tlr2	toll-like receptor 2	Upregulated
Ptprc	protein tyrosine phosphatase, receptor type, C	Upregulated
Rasgrp1	RAS guanyl releasing protein 1	Upregulated
Tlr1	toll-like receptor 1	Upregulated
Id3|LOC101056296|LOC1010	inhibitor of DNA binding 3|DNA-binding protein inhibitor ID-3-like|DNA-binding protein inhibitor I	Upregulated
Hprt	hypoxanthine guanine phosphoribosyl transferase	Upregulated
F3	coagulation factor III	Upregulated
Itgb2	integrin beta 2	Upregulated
Emr1	EGF-like module containing, mucin-like, hormone receptor-like sequence 1	Upregulated
Ifitm6	interferon induced transmembrane protein 6	Upregulated
Sell	selectin, lymphocyte	Upregulated
Psmb8	proteasome (prosome, macropain) subunit, beta type 8 (large multifunctional peptidase 7)	Upregulated
Gbp1	guanylate binding protein 1	Upregulated
Gata3	GATA binding protein 3	Upregulated
Ccl2	chemokine (C-C motif) ligand 2	Upregulated
Clec4n	C-type lectin domain family 4, member n	Upregulated
Serpina3n	serine (or cysteine) peptidase inhibitor, clade A, member 3N	Upregulated
Mx1	myxovirus (influenza virus) resistance 1	Upregulated
Fcer1g	Fc receptor, IgE, high affinity I, gamma polypeptide	Upregulated
M6pr	mannose-6-phosphate receptor, cation dependent	Upregulated
Arhgdib	Rho, GDP dissociation inhibitor (GDI) beta	Upregulated
Cxcr3	chemokine (C-X-C motif) receptor 3	Upregulated
Ctss	cathepsin S	Upregulated
Nkg7	natural killer cell group 7 sequence	Upregulated
Slc35a3	solute carrier family 35 (UDP-N-acetylglucosamine (UDP-GlcNAc) transporter), member 3	Upregulated
Tlr3	toll-like receptor 3	Upregulated
Lipa	lysosomal acid lipase A	Upregulated
Kcne3	potassium voltage-gated channel, Isk-related subfamily, gene 3	Upregulated
Retnlg	resistin like gamma	Upregulated
Ddx60	DEAD (Asp-Glu-Ala-Asp) box polypeptide 60	Upregulated
Prkcd	protein kinase C, delta	Upregulated
Anxa1	annexin A1	Upregulated
Rfwd3	ring finger and WD repeat domain 3	Upregulated
Klra17	killer cell lectin-like receptor, subfamily A, member 17	Upregulated
Ggta1	glycoprotein galactosyltransferase alpha 1, 3	Upregulated
Atp6ap2	ATPase, H+ transporting, lysosomal accessory protein 2	Upregulated
Nfkbiz	nuclear factor of kappa light polypeptide gene enhancer in B cells inhibitor, zeta	Upregulated
Gpr39	G protein-coupled receptor 39	Upregulated
Ccnb1	cyclin B1	Upregulated
Cd63	CD63 antigen	Upregulated
Hmgn3	high mobility group nucleosomal binding domain 3	Upregulated
Gm4610	predicted gene 4610	Upregulated
Gm4636	predicted gene 4636	Upregulated
1190002F15Rik	RIKEN cDNA 1190002F15 gene	Upregulated
Zfp207	zinc finger protein 207	Upregulated
I830127L07Rik	RIKEN cDNA I830127L07 gene	Upregulated
Cklf	chemokine-like factor	Upregulated
Gls	glutaminase	Upregulated
Samd3	sterile alpha motif domain containing 3	Upregulated
Wdr67	WD repeat domain 67	Upregulated
Gmfg	glia maturation factor, gamma	Upregulated
Ms4a6c	membrane-spanning 4-domains, subfamily A, member 6C	Upregulated
Dclre1c	DNA cross-link repair 1C, PSO2 homolog (S. cerevisiae)	Upregulated
Cd247	CD247 antigen	Upregulated
Syce2	synaptonemal complex central element protein 2	Upregulated
Vill	villin-like	Upregulated
Gngt2	guanine nucleotide binding protein (G protein), gamma transducing activity polypeptide 2	Upregulated
Lat2	linker for activation of T cells family, member 2	Upregulated
Snx7	sorting nexin 7	Upregulated
Mthfd1l	methylenetetrahydrofolate dehydrogenase (NADP+ dependent) 1-like	Upregulated
I830012O16Rik	RIKEN cDNA I830012O16 gene	Upregulated
Tpd52	tumor protein D52	Upregulated
Lilrb4	leukocyte immunoglobulin-like receptor, subfamily B, member 4	Upregulated
Nae1	NEDD8 activating enzyme E1 subunit 1	Upregulated
Rasgef1b	RasGEF domain family, member 1B	Upregulated
Ptprc	protein tyrosine phosphatase, receptor type, C	Upregulated
Nabp1	nucleic acid binding protein 1	Upregulated
Slc7a5	solute carrier family 7 (cationic amino acid transporter, y+ system), member 5	Upregulated
Mnd1|Gm3833	meiotic nuclear divisions 1 homolog (S. cerevisiae)|meiotic nuclear divisions 1 homolog pseudo	Upregulated
Exo1	exonuclease 1	Upregulated
Hist1h1b	histone cluster 1, H1b	Upregulated
Naip5	NLR family, apoptosis inhibitory protein 5	Upregulated
Ncoa3	nuclear receptor coactivator 3	Upregulated
Slbp	stem-loop binding protein	Upregulated
Casp1	caspase 1	Upregulated
Sptlc2	serine palmitoyltransferase, long chain base subunit 2	Upregulated
Eif5|LOC100047658	eukaryotic translation initiation factor 5|eukaryotic translation initiation factor 5-like	Upregulated
Rgs19	regulator of G-protein signaling 19	Upregulated
Map4k3	mitogen-activated protein kinase kinase kinase kinase 3	Upregulated
Uhrf1	ubiquitin-like, containing PHD and RING finger domains, 1	Upregulated
Lair1	leukocyte-associated Ig-like receptor 1	Upregulated

Gm13237	predicted gene 13237	Upregulated
Zfp266	zinc finger protein 266	Upregulated
Ticrr	TOPBP1-interacting checkpoint and replication regulator	Upregulated
Tmem194b	transmembrane protein 194B	Upregulated
Cenpw	centromere protein W	Upregulated
Frk	fyn-related kinase	Upregulated
Matr3	matrin 3	Upregulated
Bub1	budding uninhibited by benzimidazoles 1 homolog (S. cerevisiae)	Upregulated
Tmem173	transmembrane protein 173	Upregulated
Isg15	ISG15 ubiquitin-like modifier	Upregulated
Gtf2e2	general transcription factor II E, polypeptide 2 (beta subunit)	Upregulated
Ch25h	cholesterol 25-hydroxylase	Upregulated
Phf11a|Phf11b|Gm6904	PHD finger protein 11A|PHD finger protein 11B|predicted gene 6904	Upregulated
Ticam2	toll-like receptor adaptor molecule 2	Upregulated
4930506M07Rik	RIKEN cDNA 4930506M07 gene	Upregulated
Parpbp	PARP1 binding protein	Upregulated
Lbr	lamin B receptor	Upregulated
Irx2	Iroquois related homeobox 2 (Drosophila)	Upregulated
Tnfaip8	tumor necrosis factor, alpha-induced protein 8	Upregulated
Lyn	Yamaguchi sarcoma viral (v-yes-1) oncogene homolog	Upregulated
Racgap1	Rac GTPase-activating protein 1	Upregulated
Iqgap1	IQ motif containing GTPase activating protein 1	Upregulated
Ptpro	protein tyrosine phosphatase, receptor type, O	Upregulated
Usp1	ubiquitin specific peptidase 1	Upregulated
Nap1l1	nucleosome assembly protein 1-like 1	Upregulated
Chd1	chromodomain helicase DNA binding protein 1	Upregulated
Cd53	CD53 antigen	Upregulated
Pnpt1|LOC100505160	polyribonucleotide nucleotidyltransferase 1|polyribonucleotide nucleotidyltransferase 1, mitocho	Upregulated
Rnf213	ring finger protein 213	Upregulated
Mtmr12	myotubularin related protein 12	Upregulated
Abcg1	ATP-binding cassette, sub-family G (WHITE), member 1	Upregulated
Pde12	phosphodiesterase 12	Upregulated
Dsn1	DSN1, MIND kinetochore complex component, homolog (S. cerevisiae)	Upregulated
Zswim6	zinc finger SWIM-type containing 6	Upregulated
Spc24	SPC24, NDC80 kinetochore complex component, homolog (S. cerevisiae)	Upregulated
Eri1	exoribonuclease 1	Upregulated
Plbd1	phospholipase B domain containing 1	Upregulated
Msr1	macrophage scavenger receptor 1	Upregulated
E2f7	E2F transcription factor 7	Upregulated
Blm	Bloom syndrome, RecQ helicase-like	Upregulated
Adora2b	adenosine A2b receptor	Upregulated
Usp18	ubiquitin specific peptidase 18	Upregulated
Snrpd1|Gm14277	small nuclear ribonucleoprotein D1|predicted gene 14277	Upregulated
Larp7	La ribonucleoprotein domain family, member 7	Upregulated
Hs3st3b1	heparan sulfate (glucosamine) 3-O-sulfotransferase 3B1	Upregulated
Cdk8	cyclin-dependent kinase 8	Upregulated
Cfi	complement component factor i	Upregulated
Slc30a7	solute carrier family 30 (zinc transporter), member 7	Upregulated
Gbp9	guanylate-binding protein 9	Upregulated
Ska1	spindle and kinetochore associated complex subunit 1	Upregulated
Rock1	Rho-associated coiled-coil containing protein kinase 1	Upregulated
BC052040	cDNA sequence BC052040	Upregulated
Epcam	epithelial cell adhesion molecule	Upregulated
Gzma	granzyme A	Upregulated
Tifa	TRAF-interacting protein with forkhead-associated domain	Upregulated
Zfp760	zinc finger protein 760	Upregulated
Tbc1d15	TBC1 domain family, member 15	Upregulated
6720489N17Rik|2810408B13	RIKEN cDNA 6720489N17 gene|RIKEN cDNA 2810408B13 gene	Upregulated
Zfp386	zinc finger protein 386 (Kruppel-like)	Upregulated
Actn1	actinin, alpha 1	Upregulated
Vav1	vav 1 oncogene	Upregulated
Thbs1	thrombospondin 1	Upregulated
Lox	lysyl oxidase	Upregulated
Pion	pigeon homolog (Drosophila)	Upregulated
Alg13	asparagine-linked glycosylation 13	Upregulated
Sema3e	sema domain, immunoglobulin domain (Ig), short basic domain, secreted, (semaphorin) 3E	Upregulated
Rtp4	receptor transporter protein 4	Upregulated
Trim21	tripartite motif-containing 21	Upregulated
Emb	embigin	Upregulated
Rap1b	RAS related protein 1b	Upregulated
Gbp7	guanylate binding protein 7	Upregulated
Gm14137	predicted gene 14137	Upregulated
Phf14	PHD finger protein 14	Upregulated
Foxf1	forkhead box F1	Upregulated
Cdk17	cyclin-dependent kinase 17	Upregulated
Trim30d	tripartite motif-containing 30D	Upregulated
Gpr77	G protein-coupled receptor 77	Upregulated
Nudcd1	NudC domain containing 1	Upregulated
Tmed5	transmembrane emp24 protein transport domain containing 5	Upregulated
Smek1	SMEK homolog 1, suppressor of mek1 (Dictyostelium)	Upregulated
Lrrcc1	leucine rich repeat and coiled-coil domain containing 1	Upregulated
Itm2b	integral membrane protein 2B	Upregulated
Anp32e	acidic (leucine-rich) nuclear phosphoprotein 32 family, member E	Upregulated
Sat1	spermidine/spermine N1-acetyl transferase 1	Upregulated
Gins3	GINS complex subunit 3 (Psf3 homolog)	Upregulated
Cpsf2	cleavage and polyadenylation specific factor 2	Upregulated
Igj	immunoglobulin joining chain	Upregulated
Epsti1	epithelial stromal interaction 1 (breast)	Upregulated
Herc6	hect domain and RLD 6	Upregulated
Fbxo5	F-box protein 5	Upregulated
Rac2	RAS-related C3 botulinum substrate 2	Upregulated
Icos	inducible T cell co-stimulator	Upregulated
Csf3r	colony stimulating factor 3 receptor (granulocyte)	Upregulated
Aldh9a1	aldehyde dehydrogenase 9, subfamily A1	Upregulated
Sp140	Sp140 nuclear body protein	Upregulated
Irg1	immunoresponsive gene 1	Upregulated
B4galt6	UDP-Gal:betaGlcNAc beta 1,4-galactosyltransferase, polypeptide 6	Upregulated
Ccnb1ip1	cyclin B1 interacting protein 1	Upregulated

Pum2	pumilio 2 (Drosophila)	Upregulated
Cxcl5	chemokine (C-X-C motif) ligand 5	Upregulated
Rnf138	ring finger protein 138	Upregulated
Nsmce2	non-SMC element 2 homolog (MMS21, S. cerevisiae)	Upregulated
Tmod3	tropomodulin 3	Upregulated
Rnase6	ribonuclease, RNase A family, 6	Upregulated
Lonrf3	LON peptidase N-terminal domain and ring finger 3	Upregulated
Asap1	ArfGAP with SH3 domain, ankyrin repeat and PH domain1	Upregulated
Cx3cr1	chemokine (C-X3-C) receptor 1	Upregulated
Ncl	nucleolin	Upregulated
Fam60a	family with sequence similarity 60, member A	Upregulated
Stx11	syntaxin 11	Upregulated
Wdr43	WD repeat domain 43	Upregulated
Stmn1|Stmn1-rs1	stathmin 1|stathmin 1, related sequence 1	Upregulated
Alcam	activated leukocyte cell adhesion molecule	Upregulated
Serpina3j	serine (or cysteine) peptidase inhibitor, clade A (alpha-1 antiproteinase, antitrypsin), member 3J	Upregulated
Niacr1	niacin receptor 1	Upregulated
Tmeff1	transmembrane protein with EGF-like and two follistatin-like domains 1	Upregulated
Cxcl9	chemokine (C-X-C motif) ligand 9	Upregulated
Nxpe3	neurexophilin and PC-esterase domain family, member 3	Upregulated
Cd244	CD244 natural killer cell receptor 2B4	Upregulated
Gm5643|Hnrnpa1|LOC10105	heterogeneous nuclear ribonucleoprotein A1 pseudogene|heterogeneous nuclear ribonucleopro	Upregulated
Pycard	PYD and CARD domain containing	Upregulated
Snrnp48	small nuclear ribonucleoprotein 48 (U11/U12)	Upregulated
Ccr2	chemokine (C-C motif) receptor 2	Upregulated
Tpbg	trophoblast glycoprotein	Upregulated
Treml2	triggering receptor expressed on myeloid cells-like 2	Upregulated
Celf4	CUGBP, Elav-like family member 4	Upregulated
Ccrn4l	CCR4 carbon catabolite repression 4-like (S. cerevisiae)	Upregulated
Tpx2	TPX2, microtubule-associated protein homolog (Xenopus laevis)	Upregulated
Naa15	N(alpha)-acetyltransferase 15, NatA auxiliary subunit	Upregulated
Sgk3	serum/glucocorticoid regulated kinase 3	Upregulated
Grap2	GRB2-related adaptor protein 2	Upregulated
Cul4b	cullin 4B	Upregulated
Ptplb	protein tyrosine phosphatase-like (proline instead of catalytic arginine), member b	Upregulated
Kpna2	karyopherin (importin) alpha 2	Upregulated
Abce1	ATP-binding cassette, sub-family E (OABP), member 1	Upregulated
Selplg	selectin, platelet (p-selectin) ligand	Upregulated
Ifi204	interferon activated gene 204	Upregulated
Plscr1	phospholipid scramblase 1	Upregulated
Ddx10	DEAD (Asp-Glu-Ala-Asp) box polypeptide 10	Upregulated
Rnf13	ring finger protein 13	Upregulated
Commd8	COMM domain containing 8	Upregulated
Grhl1	grainyhead-like 1 (Drosophila)	Upregulated
Marcksl1	MARCKS-like 1	Upregulated
Kif2c	kinesin family member 2C	Upregulated
Mcm3	minichromosome maintenance deficient 3 (S. cerevisiae)	Upregulated
Lnx1	ligand of numb-protein X 1	Upregulated
Hnrnpa2b1	heterogeneous nuclear ribonucleoprotein A2/B1	Upregulated
Nsmce2	non-SMC element 2 homolog (MMS21, S. cerevisiae)	Upregulated
Tyw5	tRNA-yW synthesizing protein 5	Upregulated
Gins1	GINS complex subunit 1 (Psf1 homolog)	Upregulated
Ankrd55	ankyrin repeat domain 55	Upregulated
Ccne2	cyclin E2	Upregulated
Cenpl	centromere protein L	Upregulated
Mthfd1l	methylenetetrahydrofolate dehydrogenase (NADP+ dependent) 1-like	Upregulated
Sgol2	shugoshin-like 2 (S. pombe)	Upregulated
Skap1	src family associated phosphoprotein 1	Upregulated
Snx7	sorting nexin 7	Upregulated
Kcnn4	potassium intermediate/small conductance calcium-activated channel, subfamily N, member 4	Upregulated
Ptpn2	protein tyrosine phosphatase, non-receptor type 2	Upregulated
Fcgr2b	Fc receptor, IgG, low affinity IIb	Upregulated
Sltm	SAFB-like, transcription modulator	Upregulated
Fancb	Fanconi anemia, complementation group B	Upregulated
Gimap4	GTPase, IMAP family member 4	Upregulated
A530040E14Rik|LOC100503	RIKEN cDNA A530040E14 gene|uncharacterized LOC100503889	Upregulated
Gm8995	predicted gene 8995	Upregulated
Snord2	small nucleolar RNA, C/D box 2	Upregulated
Snord95	small nucleolar RNA, C/D box 95	Upregulated
Gm12238	predicted gene 12238	Upregulated
Snora41	small nucleolar RNA, H/ACA box 41	Upregulated
Gm11190	predicted gene 11190	Upregulated
BC064078	cDNA sequence BC064078	Upregulated
Pvt1	plasmacytoma variant translocation 1	Upregulated
Scarna8	small Cajal body-specific RNA 8	Upregulated
Snord33	small nucleolar RNA, C/D box 33	Upregulated
Nespas	neuroendocrine secretory protein antisense	Upregulated
Snora20	small nucleolar RNA, H/ACA box 20	Upregulated
F630028O10Rik	RIKEN cDNA F630028O10 gene	Upregulated
Rps3a	ribosomal protein S3A	Upregulated
Tmeff1	transmembrane protein with EGF-like and two follistatin-like domains 1	Upregulated
Ccrn4l	CCR4 carbon catabolite repression 4-like (S. cerevisiae)	Upregulated
Maf	avian musculoaponeurotic fibrosarcoma (v-maf) AS42 oncogene homolog	Upregulated
Magea8|Magea5	melanoma antigen, family A, 8|melanoma antigen, family A, 5	Upregulated
Pbx3	pre B cell leukemia homeobox 3	Upregulated
Gvin1|Gm4070|Gm8989|Gm8	GTPase, very large interferon inducible 1|predicted gene 4070|very large inducible GTPase 1 p	Upregulated
Trim21	tripartite motif-containing 21	Upregulated
BC003331	cDNA sequence BC003331	Upregulated
Mov10	Moloney leukemia virus 10	Upregulated
Kng2|Kng1	kininogen 2|kininogen 1	Upregulated
Gas2l3	growth arrest-specific 2 like 3	Upregulated
Klrc2|Klrc3|Klrc1	killer cell lectin-like receptor subfamily C, member 2|killer cell lectin-like receptor subfamily C, m	Upregulated
Tpd52	tumor protein D52	Upregulated
Trim30d|Trim30a	tripartite motif-containing 30D|tripartite motif-containing 30A	Upregulated
Grhl1	grainyhead-like 1 (Drosophila)	Upregulated
Baz1a|LOC100048557	bromodomain adjacent to zinc finger domain 1A|bromodomain adjacent to zinc finger domain p	Upregulated
Klra8|LOC100862437|Klra9|K	killer cell lectin-like receptor, subfamily A, member 8|killer cell lectin-like receptor 3-like|killer cel	Upregulated
Nasp	nuclear autoantigenic sperm protein (histone-binding)	Upregulated

Table S2 List of DJ/GC combination-related genes					
**Genes upregulated in HCC ascites mouse model compared to normal control, but downregulated after the treatment of DJ alone**	**Genes upregulated in HCC ascites mouse model compared to normal control, but downregulated after the treatment of DJ/GC synergy combination**	**Genes upregulated in both HCC ascites mouse model and DJ/GC-antagonism compared to normal control**	**Genes downregulated in HCC ascites mouse model compared to normal control, but upregulated after the treatment of DJ alone**	**Genes downregulated in HCC ascites mouse model compared to normal control, but upregulated after the treatment of DJ/GC synergy combination**	**Genes downregulated in both HCC ascites mouse model and DJ/GC-antagonism compared to normal control**
1100001G20Rik	1700097N02Rik	8430408G22Rik	1700020A23Rik	2310047D07Rik	Btg2
1700001L05Rik	4933406F09Rik	Gm13476	Adi1	2810416G20Rik|LOC101056638	
1700097N02Rik	AA667203	Rab33a	Clta	7530428D23Rik	
4921539H07Rik	Atp6v1e2	Tmem252	Cox6a2	Acsl6	
A230006K03Rik	C1qtnf3		Gm3646	Amhr2	
AA667203	Caly		Krt14	Art5	
BC030307	Ccdc103		Mylk4	Asb10	
Bsn	Dclre1c		NA	Chac1	
Ctag2	Dmrtc1a		Rsph1	Clta	
Dmrtc1a	E230025N22Rik		Themis3	Fbxl22	
Ephx3	Epha8			Fga	
Eps15	Eps15			Gamt	
Frk	Fmo9			Gm16119	
Gm11190	Frk			Gm3646	

Krtap11-1	Gm11190			Gmpr	
Lats1	Grip1			Hhatl	
Mirlet7e	Hbb-bh1			Itgb1bp2	
Rpl39l	Igj			Ky	
Slamf6	Krtap4-6			Mylk4	
Srsf11	Lce1d			Myoz3	
Stard4	Mak			NA	
Syce3	Mia3			Phkg1	
Tor1a	Mirlet7e			Pvalb	
Vmn1r230	Myo1f			Themis3	
Wbscr28	NA			Tmem233	
	Nespas			Tpm2	
	Prss44				
	Samd3				
	Serpina3f				
	Spic				
	Syt6				
	Tor1a				
	Ush2a				
	Vmn1r230				
	Zfp933				

Table S3 Interaction network of HCC ascites-related gene-DJ/GC combination-related gene-known therapeutic target gene for ascites

Node1	Node2
Clspn	Gins2
Plcg2	Cd180
Pik3c2a	Pi4k2b
Tlr2	Hck
Chtf18	Gins2
Adam8	Lpxn
Cenpi	Kif23
Hist1h2ac	Top2a
Arhgef39	Cenpn
Lig1	Kif4
Mis18bp1	Tpx2
Naip2	Casp1
Shcbp1	Mcm7
Shcbp1	Kif11
Ctss	Tlr7
Lilrb4	Slc11a1
Bub1	Ska3
Lcp2	Skap1
C1qc	Ms4a6d
Ccne2	Myc
Vav1	Hck
Abi1	Rac2
Rad51	Mcm7
Gbp1	Vcam1
Foxm1	Mcm10
Gbp1	Psmb8
Mad2l1	Plk1
Prc1	Ccnf
Prc1	Fbxo5
Coro1a	Aif1
Prc1	Cenpa
Ifi203	Gbp3
Pbk	Mcm7
Spc24	Casc5
Ggta1	St3gal3
Mad2l1	Kif23
Cdca5	Uhrf1
Nr1d1	Ppara
Parp9	Irf7
Ghsr	Kiss1
Psmb9	Parp14
Palb2	Fancd2
Ifi44	Ifih1
Tacc3	Trip13
Chaf1b	Cdt1
Arpc1b	Actr2
Kif20a	Cdca3
Mki67	Cdt1
Ccl9	Gnai3
Igtp	Parp12
Batf2	Zbp1
Rad51	Cenpk
Esco2	Kif11
Skp2	Foxm1
Arhgdib	Casp1
Mcm3	Gins2
Nusap1	Bub1
Blm	Brca1
Ly86	Emr1
Ccdc99	Cenph
Ntf3	Npy
Clspn	Pbk
Bub1b	Fam64a
Spp1	Timp1
Gins1	Hells
Foxm1	Kif11
Ccnb2	Racgap1
Dsn1	Esco2
Fignl1	Mcm4
Atad2	Mcm4
Kif4	Plk1
Gatm	Aldh9a1
Dbf4	Ccnb1
Rilp	Tuba8
Ccdc99	Kif11
Casc5	Ska3
Rad54l	Mcm3
Chtf18	Uhrf1
Mcm5	Trip13
Pgm2	Prps2
Lyve1	Hmmr
Hells	Hmmr
Igsf6	Aif1
Samd9l	Rtp4
Mad2l1	Prim1
Igsf6	Cybb
Btk	Tyrobp
Fcgr2b	Hck
Satb2	Hdac9
Kif4	Hmmr
Iigp1	Ifi44
Ccnb1	Prkcb
Cenpa	Shcbp1
Pbk	Gmnn
Clspn	Fen1
Ptprc	Casp1
Ccnf	Mcm10
Alox5ap	Cysltr1
Lrr1	Prim1
Kif20b	Plk4
Fcer1g	Fcgr2b
Cdc6	Mcm7

Aif1	Clec4n
Exo1	Hmmr
Rad51c	Rad51
Msn	Ncf1
Gsg2	Bub1
Zbp1	Ifih1
Arf6	Lpxn
Cdc6	Racgap1
Parpbp	Racgap1
Chrm5	Gpr65
Nek2	Asf1b
Anln	Bub1b
Ccr6	Rxfp3
Fyb	Cd53
Rgs18	Cxcl13
Blm	Fancd2
Lck	Prkcb
Fcgr3	Slc11a1
Cdkn3	Parpbp
Fpr1	Rgs18
Cenpq	Kif2c
Ifit2	Parp14
Cenpa	Racgap1
Kif20b	Parpbp
Mad2l1	Chtf18
Kcnn4	Cav3
Arhgap30	Nckap1l
Cdca5	Plk2
Mcm5	Chaf1b
Cxcl9	Ccl6
Orc6	Cdt1
Spc24	Ska1
Mad2l1	Kif4
Ifih1	Casp1
Clec4a3	Pld4
Vcam1	Ccl2
Mylpf	Ppp1r27
Cenph	Hmmr
Cenpa	Tacc3
Tlr2	Ifih1
Bub1	Fen1
Exo1	Prim1
Cxcl9	Fcgr4
Rbm38	Myog
Kif2c	Asf1b
Rxfp3	Ccr2
Prc1	Tacc3
Anxa1	Ccl9
Ptgs2	Timp1
Mpeg1	Ncf4
Ifit2	Ddx60
Samhd1	Ifi47
Stat1	Btk
Spc24	Plk4
Exo1	Gins2
Kif20a	Shcbp1
Plk4	Incenp
Cdkn3	Ccna2
Kif20a	Depdc1b
Nusap1	Fbxo5
Ifit1	Irgm2
Cenpa	Kif2c
Stxbp1	Exoc3l4
Kif4	Mki67
Ect2	Bub1b
AF25170	Aif1
Kif20b	Spc25
Igtp	Ifih1
Fcgr4	Fcgr1
Cenpa	Cdca2
Cenpl	Cenpk
Cdc25c	Mcm7
Kif20a	Kif2c
Dlgap5	Racgap1
Cd68	Itgam
Pcx	Mthfd1l
Mylpf	Pvalb
Ncf4	Tlr2
Cdca2	Kif20b
Prkcb	Rictor
Kif2c	Bub1b
Kif23	Plk2
Kif20a	Kif23
Aspm	Casc5
Foxm1	Fam64a
Rac2	Rps6ka2
Kif20b	Exo1
Cenph	Fbxo5
Plk1	Cdca5
Pbk	Spc25
Orc1	Prim1
Depdc1a	Cdca5
C1qb	Fcgr1
Cd68	C1qb
Kcnc1	Kcna7
Coro1a	Cd52
Nckap1l	Emr1
Kcnc1	Kcne3
Dlgap5	Hmmr
Adam8	Clec4d
Diap3	Kif20b
Ncapg2	Esco2
Kif4	Rad51
Themis2	Fcgr1
Ccr5	Ccl9

Dtna	Sgcg
Cxcl2	Ccl3
Camk2b	Cdc25c
Ttk	Ska3
Cdc6	Esco2
Spc24	Bub1b
Mcm3	Orc6
Irf8	Fcgr1
Fam26f	Parp14
Stat1	Casp1
Ctss	Fcer1g
Ect2	Cdca5
Birc5	Rrm2
Atad2	Hmmr
Plk1	Mastl
Kif4	Smc4
Hmmr	Asf1b
Iigp1	Gbp1
Pld4	Ly86
Isg15	Irf8
Retnlg	Vcam1
Arhgdib	Racgap1
Prc1	Lrr1
Nckap1l	Coro1a
Ccdc99	Trip13
Myod1	Smarcd3
Retnlg	Npy
Cdkn3	Sgol2
Aspm	Fbxo5
Dhrs7c	Myom2
Rad54l	Ccnb1
Skp2	Fbxo15
Cdca5	Pbk
Sgol2	Spc25
Gbp7	Ifi47
Sstr5	Ccl5
Shcbp1	Pbk
Hist2h3c2	Asf1b
Nr1d1	Rxrg
Usp18	Gbp3
Csf2rb	Lyn
Kif4	Uhrf1
Gbp7	Psmb8
Ezh2	Top2a
Rnf213	Irgm2
Ncf4	Csf2rb2
Clta	Rps6ka2
Mcm3	Racgap1
Mad2l1	Ttk
Ms4a6c	Ms4a6d
Lyn	Themis2
Kif20a	Spc24
Nusap1	Shcbp1
Atp8b4	Depdc7
Rac2	Sash3
Tmod1	Myl3
Ppara	Mcm7
Prc1	Ezh2
Ifit2	Usp18
Ryr1	Myc
Tpx2	Mcm10
Gbp2	Parp14
Stat1	Mx1
Kif20b	Mcm7
Inpp5d	Plcg2
Plcg2	Nckap1l
Ccnb1	Gsg2
Top2a	Prim1
Kif2c	Gsg2
Parpbp	Rad51
Plk5	Kif23
Tacc3	Incenp
Ms4a6d	Aif1
Itga7	Tspan32
Kif20a	Arhgap11a
Tyrobp	Fcgr2b
Cxcl2	Ccr2
Ccr6	Cxcl1
Ifi203	Ifi44
Ifit2	Ifih1
Wasf2	Fcgr2b
Il18bp	Vcam1
Cxcl2	Anxa1
Cdkn3	Plk4
Ccnf	Kif11
Rad54l	Ska3
Scn1b	Scn4b
Tlr4	Btk
Stat1	Ifi44
Kif20a	Ttk
Kif20b	Birc5
Cdca2	Mcm3
Ect2	Shcbp1
Depdc1a	Mis18bp1
Batf2	Irf7
Gbp2	Irf8
Prc1	Ect2
Csf3r	Ptpn6
Ezh2	Mcm3
Mad2l1	Tacc3
Fignl1	Cenpn
Ryr1	Cacna1s
Hells	Pbk
Stat1	Fgfr4
Rtp4	Ifi44

Cenpn	Spc25
AF25170	Ly86
Hrc	Hfe2
Bub1	Hmmr
Shcbp1	Ska3
Six2	Otx1
Itpr3	Vav1
Itgam	Ptgs2
Ccna2	Rbl1
Lrr1	Dlgap5
Ttk	Rad51
Arap2	Rac2
Gamt	Ckmt2
Aspm	Ska1
Ms4a7	Ms4a6d
Pld4	Itgb2
Cdca7	Hells
Mad2l1	Cenpi
Ttk	Trip13
Ccnb2	Asf1b
Kif20a	Plk2
Ifi47	Zbp1
Ccl6	Gnai3
Lrr1	Cenpi
Fpr2	Rgs18
Clspn	Ccna2
Tlr4	Fcgr2b
Cxcr7	P2ry12
Mcm10	Fen1
Cdkn3	Nek2
Smarcd3	Ankrd1
Kif23	Spc25
Nfkbiz	Map3k8
Ank1	Obscn
Mad2l1	Pbk
Phf11d	Irf7
Mcm5	Cenph
Troap	Cenpn
Ifit2	Igtp
Sla	Lyn
Orc6	Mcm10
Dnttip2	Nop58
Tacc3	Uhrf1
Cdca2	Cdca3
Fcgr3	Cd3g
Stat1	Irf8
Cdc6	Tacc3
F13a1	Srgn
Arpc5	Actr3
Plk4	Cenpk
Cdca2	Cdca7
Clspn	Kif11
Ttk	Mcm4
Cdca2	Aspm
Clspn	Mcm10
Ms4a4c	Sell
Ccnb1	Cdca5
Cdkn3	Trip13
Mad2l1	Ccdc99
Oasl1	Irg1
Mcm10	Mcm7
Ptprc	Frk
Tacc3	Racgap1
Kif20a	Aspm
Ctss	Casp1
F3	F2rl1
Ccnb2	Cenpk
Cenpk	Uhrf1
Rxfp3	Anxa1
Mad2l1	Top2a
Ccnb2	Nek2
Cxcl2	Cx3cr1
Igsf6	Slc11a1
Plk4	Hmmr
Ms4a6d	Emr1
Tpx2	Rrm2
Ccna2	Rad51
Dlgap5	Pbk
Cenph	Cdc25c
Ifi204	Ifit2
Arhgap11	Mad2l1
Topbp1	Ccna2
Ddx60	Gbp3
Tacc3	Kif4
Lck	Btk
Ect2	Rac2
Fignl1	Tpx2
Mcm5	Plk1
Troap	Fam64a
Prc1	Kif23
Blm	Rad54l
Lrr1	Birc5
Fbxo5	Asf1b
Wdhd1	Cdca5
Ttk	Arhgef39
Cxcl1	Ccl9
Sirpa	Tyrobp
Hist1h4i	Hist1h2ao
Kif20a	Gins2
Wdhd1	Dlgap5
Dbf4	Pola1
Cenpa	Hmmr
Mad2l1	Cenpk
Psmb9	Mad2l1
Asf1b	Spc25

Aspm	Anln
Depdc1a	Bub1b
Cxcl5	Cxcl13
Irgm2	Gbp1
Birc5	Trip13
Ccnb1	Orc6
Cd55	Emr1
C1qc	Ly86
Fen1	Cdt1
Ank1	Itpr3
Msn	C1qc
Pbk	Cenpk
Cenpn	Hmmr
Arpc1b	Actr3
Cdc6	Ska1
Hrc	Ckmt2
Diap3	Kif23
Prf1	Ptprc
Nusap1	Plk4
Clspn	Rad51
Mcm4	Uhrf1
Sgol2	Plk4
Mcm5	Troap
Cdc7	Mcm4
Aspm	Hells
P2ry12	Npy
Ncoa3	Nr1d1
Gbp7	Ifih1
Top2a	Rbl1
Ncapg2	Rad51
Kif20a	Rad51
Plek	Vav1
Lig1	Pbk
Clspn	Sgol2
Rasa1	Smek2
Scn4b	Scn4a
Ifit1	Ifih1
Ccl9	Ccl6
Cdca5	Cdca3
Anln	Cdca5
Ttk	Kif15
Ctss	Ly86
Ect2	Nek2
Ncapg2	Aspm
Prkag3	Phkg1
Foxm1	Bub1b
F3	Tfpi
Ttk	Kif4
Parp9	Irgm2
Myom2	Itgb1bp2
Cenpa	Sgol2
Ccnb1	Arhgef39
Ezh2	Myod1
Ctss	Coro1a
Dbf4	Orc6
Ccnf	Aspm
Lcp2	Ptprc
Igtp	Ifi47
Coro1a	Vav1
Cdca3	Asf1b
Lcp2	Fcgr1
Ccna2	Brca1
Incenp	Racgap1
Lig1	Fbxo5
Casc5	Shcbp1
Cenpi	Fignl1
Arhgap11	Cenpi
Kif20a	Mcm7
Mcm5	Kpna2
Kif20a	Pbk
Fcgr4	Ly86
Birc5	Rad51
Bub1b	Casc5
Phf11d	Gbp3
Cxcl9	Cxcl1
Foxm1	Asf1b
Ppara	Ptgs2
Sgol2	Fam64a
Nusap1	Arhgef39
Tlr2	Casp1
Aspm	Bub1b
Psmb9	Cxcl9
Ccnb1	Rbl1
Cdca5	Cdt1
Mcm5	Ccne2
Tlr2	Emr1
Ttn	Tmod1
Myo18b	Myom1
Top2a	Lin54
Kif2c	Gins2
Ccna2	Trip13
Top2a	Shcbp1
Ccr5	Ms4a6d
Bub1b	Mcm7
Top2a	Smc4
Ifih1	Dhx58
Mad2l1	Anln
Prc1	Plk1
Ccnb2	Ccna2
Racgap1	Cdt1
Ttn	Actc1
Lepr	Ptpn6
Fcgr3	Cd52
Fam57b	Asphd1
Arhgap11	Tacc3

Samhd1	Igtp
Cdkn1c	Igf2
Ncapg2	Tacc3
Cdca2	Ccna2
Mis18bp1	Anln
Birc5	Spc25
Niacr1	Cxcl5
Cenph	Casc5
Stat1	Iigp1
Gins2	Mcm10
Ccnf	Fbxo15
Apobec2	Mylpf
Ncf1	Vcam1
Top2a	Hells
Cd69	Emr1
Psmb9	Lyn
Kif20a	Sgol2
Rnf213	Irf7
Anln	Rrm2
Themis2	Ptprc
Ptpn6	Fasl
Dnahc2	Dnaic1
Mad2l1	Clspn
Ccdc99	Uhrf1
Kif20a	Cenpk
Ccnf	Plk1
Camk2b	Plk1
Mis18bp1	Casc5
Rad51	Spc25
Btk	Vav1
Cenph	Ccnb1
Nusap1	Incenp
Ppara	Myc
Tpx2	Kpna2
Cx3cr1	Ccl3
Cdca2	Clspn
Ccdc99	Cenpk
Samd9l	Ifit2
Cdkn3	Aspm
Parpbp	Mki67
Exo1	Bub1b
Mis18bp1	Asf1b
Kif20b	Cenph
Kif23	Cdca5
Ezh2	Vav1
Gbp7	Ifi44
Mcm5	Bub1b
Ms4a6c	Hck
Cdc6	Cenpn
Ccdc99	Fignl1
Bub1	Mcm6
Itga7	Mcm7
Ccnb1	Top2a
P2ry12	Cxcl1
Kif11	Mcm7
Nckap1l	Ptpn6
Arhgef39	Mki67
Mcm5	Pbk
Gins2	Uhrf1
Prc1	Depdc1b
Arhgef39	Sgol2
Fcgr4	Slc15a3
Ptprc	Cybb
Prc1	Pbk
Parp9	Parp14
Prkcb	Grin1
Clspn	Trip13
Actc1	Coro1a
Cenpa	Hist1h4i
Ticam2	Tlr1
Spc24	Cdc6
Plk4	Kif11
Esco2	Fbxo5
Ccne2	Mcm10
Racgap1	Plk2
Exo1	Pola1
Smarcd3	Hells
Fam64a	Spc25
Kif2c	Trip13
Nek2	Pbk
Mcm3	Ccnb2
Stat1	Ifi47
Sell	Serpinb9b
Dsn1	Kif11
Mis18bp1	Cenpk
Cav3	Ptgs2
Ccna2	Fen1
Esm1	Coro1a
Lig1	Exo1
Cd53	Ptpn6
Ccr5	Gpr18
Tex30	Kpna2
Orc1	Prim2
Ncapg2	Kif4
Irgm2	Herc6
Lair1	Emr1
Isg15	Rtp4
Plk4	Mcm7
Spc24	Clspn
Mcm5	Orc1
Lrr1	Kif4
Clta	Eps15
Ccr2	Ccl6
Diap3	Rac2
Ect2	Bub1

Cacna1d	Gnai3
Cdkn3	Cdc25c
Phospho:	Ache
Cdca5	Gmnn
Depdc1a	Arhgap11a
Nek2	Cdca5
Igtp	Irf7
Rad51	Uhrf1
Ccnb2	Mki67
Lyn	Ptpn6
Gins2	Chaf1b
Ifit2	Herc6
Clec4a3	Alox5ap
Aspm	Exo1
Exo1	Rad51l3
Ect2	Ccna2
Mus81	Fen1
Igsf6	Hck
Mis18bp1	Plk4
Fcgr2b	Ms4a6d
Birc5	Tuba1c
Frk	Fasl
Ccna2	Nek2
Bub1	Mcm10
Phf11d	Igtp
Gpr18	Gnai3
Ttk	Fbxo5
Gins1	Cdt1
Casc5	Rad51
Prc1	Cenpk
Bub1b	Cenpn
Parp12	Zbp1
Dbf4	Plk2
Lmnb1	Brca1
Irgm2	Igtp
Fyb	Arhgap30
Top2a	Plk1
Cenpn	Cenpk
Cd55	Emr4
Il18	Timp1
Rtp4	Zbp1
Gpr18	Ccl5
Kif2c	Dlgap5
Ifit1	Rnf213
Orc6	Mcm4
Spc24	Nusap1
Ccr6	Ccl6
Ccnb1	Mcm4
Pik3c2a	Plcg2
Cdkn3	Ttk
Orc1	E2f8
Fcgr2b	Ptpn6
Cx3cr1	Gnai3
Nckap1l	Vav1
Ptpn6	Hck
Nsmce2	Top2a
Cd48	Ccl5
Cd72	Ptprc
Depdc1b	Tpx2
Rtp4	Parp14
Ccnb2	Bub1
Kcne3	Kcnc4
Parpbp	Fam64a
Rad51	Shcbp1
Ddx60	Irf7
Mcm5	Kif20b
Ect2	Cenpk
Bub1b	Chaf1a
Depdc1b	Arhgdib
Cdc6	Bub1
Lck	Itgam
Birc5	Cenpn
Fcgr3	Inpp5d
Cxcl2	Ccl4
Tacc3	Shcbp1
Mapk12	Hspb2
Gpr18	Cxcl1
Psmb9	Gbp3
Akr1a1	Aldh9a1
Casp3	Ldb3
Gbp7	Irf7
Topbp1	Bard1
Bzw1	Ncl
Prc1	Hmmr
Nek2	Rrm2
Cdca3	Racgap1
Ncf4	Fyb
Gpr65	Anxa1
Ly96	Cd180
Plk4	Mcm4
Aspm	Cdca3
Ect2	Mcm6
Mad2l1	Topbp1
Birc5	Gsg2
Mad2l1	Lig1
Arhgef39	Uhrf1
Ncf4	Vcam1
Cenph	Cenpk
Fpr1	Ccl6
Inpp5d	Csf2rb2
Cxcl9	Irgm2
Dbf4	Kif11
Obsl1	Ttn
Gbp1	Rsad2
Esco2	Uhrf1

Tuba1c	Mapre3
Ifit2	Mx1
Tyrobp	Clec4d
Cd6	Sdcbp
Arhgap11	Ccnb2
Orc1	Trip13
Myl3	Myod1
Anln	Cd2ap
Prkcb	Itpr3
Mcm3	Topbp1
Nusap1	Plk1
Mcm3	Asf1b
Fignl1	Incenp
Syce2	Syce3
Top2a	Rad51l3
Rtp4	Iigp1
Ncapg2	Rbl1
Obscn	Mylpf
Myc	Vmp1
Kif2c	Mastl
Cenpi	Tpx2
Mus81	Exo1
Camk2a	Stat1
Ikzf1	Sell
Mki67	Tpx2
Ska1	Mki67
Serpina3	Casp1
Arhgef39	Bub1
Cenpi	Cenph
Ttk	Cenpk
Cx3cr1	Ccl6
Trip13	Tex11
Ect2	Mki67
Blm	Fen1
Lrr1	Sgol2
Ifi204	Ifi203
Kif2c	Rrm2
Ect2	Ezh2
Myo18b	Myod1
Mastl	Hmmr
Ccr5	C3ar1
Lat	Tyrobp
Cdca5	Fam64a
Mki67	Mcm7
Mapk12	Ptpn7
Orc1	Gmnn
Itgam	Ccl2
Topbp1	Cdc7
Top2a	Pif1
Prc1	Gins1
Dsn1	Trip13
Kif20a	Ccdc99
Ccnb1	Mastl
Actc1	Obscn
Racgap1	Cenpk
Cdca2	Arhgef39
Lcp2	Cd3g
Serpine1	Tlr2
Tpd52	Myc
Mcm3	Ska3
Fignl1	Racgap1
Tacc3	Dlgap5
Racgap1	Cybb
Ccna2	Bub1
Lrr1	Racgap1
Ccnb2	Hmmr
Chtf18	Rad51
Irgm2	Irf7
Blm	Dhx58
Cdkn3	Rasgef1b
Cenph	Cdca5
Rac2	Coro1a
Ttn	Capn3
Stat1	Kpna2
Ptpn22	Ptprc
Ddx60	Isg15
Topbp1	Gins1
Camp	Hdac9
Mcm5	Stat1
Plaur	Itgb2
Srl	Casq1
Top2a	Bub1
Ttk	Depdc1b
Kif23	Ccnb2
Msn	Itgb2
Tyrobp	Ly86
Prim2	Pola1
Foxm1	Dlgap5
Nusap1	Sgol2
Dbf4	Orc1
Fpr1	Ccl9
Rad54l	Cdca5
Cdc6	Kif20b
Exo1	Nek2
Ccnb1	Mis18bp1
Lcn2	Thbs1
Birc5	Fen1
Mastl	Racgap1
Stat1	Frk
Mcm5	Top2a
Serpine1	Cfb
Vav1	Ptpn6
Smc4	Pola1
Psmb9	Gbp1
Parpbp	Kif11

Cenpi	Casc5
Mad2l1	Hells
Plk1	Cdca3
Clec4n	Ccl6
Cenpa	Cenpk
Ccnb1	Asf1b
Parpbp	Kif23
Depdc1b	Hmmr
Ncapg2	Tpx2
AF25170	Fcer1g
Ddx10	Trnt1
Fcer1g	Hck
Odf3l2	Birc5
Ccne2	Cdca5
P2ry12	Cxcl5
Mad2l1	Mcm3
Casp3	Lats1
Rbl1	Brca1
Cdc6	Aspm
Ect2	Trim59
Shcbp1	Kpna2
Mybph	Mylpf
Ifit2	Ifi47
Lpxn	Frk
Parp14	Irf7
Troap	Ccna2
Cdca7	Ccnb1
Top2a	Nek2
Cdc6	Gins1
Ifit2	Oasl1
Bub1	Racgap1
Ccr2	Cxcl5
Mad2l1	Trip13
Ccr6	Niacr1
Ifi47	Rtp4
Mcm3	Mcm10
Lig1	Rad51
Usp18	Irf7
Cenpi	Birc5
Ccr6	Xcl1
Depdc1a	Ttk
Ifit2	Ifi44
Iigp1	Zbp1
Kif20b	Anln
Ccdc99	Ccnb2
Chaf1b	Pbk
Birc5	Cdca3
Hells	Pola1
Lig1	Shcbp1
Tacc3	Fbxo5
Incenp	Asf1b
Cd53	Sash3
Arhgap25	Arhgdib
Ifi44	Zbp1
Kif2c	Plk4
Birc5	Cenpk
Top2a	Atad2
Sirpa	Fyb
Cenpi	Gsg2
Ska1	Exo1
Cenph	Mastl
Cenpa	Plk4
Palb2	Rad51
Rasgrp1	Rap1b
Casc5	Sgol2
Rictor	Map3k8
Casc5	Cdca5
Depdc1a	Ccne2
Ifi47	Parp12
Lat	Trem2
Cenpi	Ccnb2
Pla2g4e	Plcg2
Plcg2	Tlr4
Lck	Map4k1
Kif23	Pbk
Myo1f	Tyrobp
Col22a1	Emid2
Lck	Fyb
Csf2rb2	Csf2ra
Nusap1	Kif2c
Grip1	Ncoa3
Mcm3	Mcm4
Fpr2	Sstr5
Ifit1	Gbp1
Kif4	Cenpn
Prc1	Casc5
Ccr6	Ccl2
Depp	Npy
Usp1	Dek
Orc1	Skp2
Cdkn3	Rrm2
Nsmce2	Smc4
Ccnb1	Depdc1b
Cenph	Ttk
Cdca2	Fam64a
Cenpa	Spc24
Prc1	Mcm7
Ctss	C1qb
Chtf18	Prim2
Casq2	Srl
Ncf4	Cd53
Prf1	Gzmb
Cdca5	Hmmr
Fignl1	Kif11
Prc1	Uhrf1

Cxcr6	Tlr4
Mypn	Ldb3
Arf6	Epha2
Parp9	Ifi44
Esco2	Brca1
Lcp2	Cd69
Mad2l1	Brca1
Tpx2	Kif11
Cdkn3	Shcbp1
Itgam	Plaur
Inpp5d	Btk
Depdc1b	Nek2
Casp3	Prf1
Isg15	Usp18
Il24	Csf2ra
Cd68	Ptprc
Kif20b	Cdt1
Depdc1a	Uhrf1
Cdc7	Kif11
Fga	Calca
Mapk12	Rap1b
Skap1	Rap1b
Cdc7	Mcm7
Ncf1	Cybb
Dbf4	Topbp1
Ckmt2	Trim54
Cxcl9	Cx3cr1
Stxbp1	Rab3a
Kif20a	Chtf18
Kif4	Shcbp1
Cdc25c	Rrm2
Npy	Ccl6
Plk1	Kpna2
Cdkn3	Top2a
Cenpn	Rad51
Rnf213	Igtp
Cd68	C1qc
Tacc3	Plk1
C1qb	Clec4n
Ifit1	Usp18
Cenpk	Trip13
Evi2a	Cd48
Mpeg1	Pld4
Epcam	Itga6
Syce2	Terf1
Psmb9	Skp2
Ccnf	Plk2
Irg1	Ccl4
Kcnc1	Kcng4
Tacc3	Exo1
Cxcr6	Ccl7
Lair1	Aif1
Ccnf	Kif2c
Vav1	Cd3g
Gamt	Gatm
Cd48	Ly86
Ect2	Ccnb1
Mcm6	Gmnn
Hrc	Ldb3
Fam64a	Kif11
Arhgap11	Bub1
Orc1	Mad2l1
Ezh2	Suz12
Cacna1d	Camk2b
Mus81	Rad51l3
Wnk2	Stradb
Cenpl	Cenpn
Cd53	Tyrobp
Sgcg	Cav3
Cdc6	Atad2
Gsg2	Cenpn
Mcm5	Ska3
Plk1	Pola1
Fcgr2b	Cd3g
Usp18	Herc6
Abi1	Frk
Clspn	Orc6
Lrr1	Lig1
Oasl1	Gbp3
Nusap1	Spc25
Birc5	Mcm6
Top2a	Rad51
Plk1	Mcm6
Nek2	Ska3
Oasl1	Gbp1
Lrr1	Clspn
Depdc1b	Cdca5
Fcer1g	Ptpn6
Dlgap5	Uhrf1
Dctpp1	Pola1
Ctss	Cd68
Mcm6	Racgap1
Gbp7	Oasl1
Lilrb4	Emr1
Mcm5	Ccnf
Lck	Hck
Mki67	Ska3
Plk1	Rrm2
Ccl9	Kiss1
Stat1	Tlr7
Anln	Plk1
Ncapg2	Trip13
Cdca2	Ska1
Ctss	Igsf6
Ect2	Asf1b

Plek	Ptprc
Kif23	Mcm4
Cxcl9	Ccr5
Lcp2	Tyrobp
Cacna1s	Cacnb1
Cenpl	Dsn1
Osmr	Ptpn6
Fcgr1	Msr1
Stxbp1	Rab8b
Themis2	Coro1a
Alox5ap	Itgb2
Wdhd1	Cdc6
Mis18bp1	Ccna2
Ccnb1	Btg2
Cd3d	Pdcd1
Themis2	Rac2
Cdca2	Parpbp
Cenpl	Ccdc99
Cxcl9	Ifi47
Cxcr7	Gpr18
Asf1b	Mcm7
Fpr1	Cxcl13
Pla2g1b	Pla2g7
Exo1	Kif11
Rbl1	Myc
Ptprc	Ltb
Irf8	Ly86
Rac2	Nckap1l
Lin54	Rbl1
Spc24	Fam64a
Ifit1	Ddx60
Dock2	Rac2
Mapk12	Gnai3
Ppara	Tlr4
Ccr6	Cxcl5
Eif1a	Bzw1
Skap1	Ptprc
Tlr1	Ptprc
Cd52	Itgb2
Psmb9	Zbp1
Retnlg	Ucp3
Myo1f	Actr2
Cenpi	Esco2
Cdc6	Ccl2
Ptgs2	Myc
Epha2	Fgf6
Usp1	Hells
Foxm1	Plk2
Rps6ka2	Apaf1
Parp12	Oasl1
Mcm3	Incenp
Sgol2	Trip13
Ifit1	Stat1
Spc24	Rrm2
Cdc25c	Kif23
Fyb	Themis2
Anln	Mcm4
Lck	Havcr2
Dlgap5	Incenp
Gins2	Asf1b
Kif20b	Cenpn
Lyn	Fcgr1
Ccne2	Cdc6
Prim1	Trip13
Nusap1	Nek2
Kif4	Fam64a
Kif2c	Tpx2
Tap1	B2m
Glipr1	Smarcd3
Fcgr3	Clec4n
Skp2	Pbk
Ect2	Smc4
Mad2l1	Fbxo5
Top2a	Mki67
Cd68	Msr1
Tacc3	Bub1
Ncf4	Mapk12
Arf6	Acap1
Hdac9	Dyrk1b
Ccr6	Gnai3
Ccr6	Cxcr6
Cd48	Rac2
Abi1	Rasgef1b
Atad2	Racgap1
Stat1	Dhx58
Mus81	Rad54l
Chtf18	Bub1
Mcm6	Mcm4
Ccna2	Cdca3
E2f8	Apaf1
Myo1f	Nckap1l
Trim59	Pbk
Gbp2	Rtp4
Foxm1	Trip13
Ccnb1	Rrm2
Ifit1	Mx1
Foxm1	Top2a
Inhbe	Inha
Irgm2	Oasl1
Bub1b	Tpx2
Cdca3	Pbk
Hist1h2a	Top2a
Topbp1	Trip13
Tlr7	Irf8
Nckap1l	Sash3

Kif20b	Kif23
Plek	Ncf4
Cfb	Serpina3n
Mcm10	Gmnn
Cenpa	Casc5
Mad2l1	Aspm
Parp9	Ifit1
Fbxo5	Mcm7
Rad51	Fen1
Gbp2	Isg15
Lrr1	Pbk
Fbxo5	Cdt1
Rpl39l	Rpl19
Arhgap11	Esco2
Sla	Lck
Fcgr4	Hck
Ifi47	Rsad2
Cenpi	Cdkn3
Anln	Ska3
Igf2	Gsk3a
Fam26f	Ifi44
Ezh2	Abcb4
Ect2	Nusap1
Birc5	Troap
Skap1	Hck
Mad2l1	Foxm1
Obscn	Pvalb
Gins2	Cenpn
Spp1	Itga6
Prc1	Kif2c
Smc4	Mcm7
Dbf4	Shcbp1
Skp2	Uhrf1
Cdc6	Ppp2r3a
Ncf4	Lpxn
Tpx2	Trip13
Ccr6	Fpr2
Coro1a	Actr2
Arhgap11	Mis18bp1
Fcgr3	Ctss
Birc5	Incenp
Prim1	Hells
Fam26f	Iigp1
Kif23	Bub1
Rad51	Pbk
Top2a	Chaf1a
Dsn1	Cdca5
Slc2a3	Myc
Ccna2	Hmmr
Ccnb2	Prim1
Tacc3	Cenpk
Ncapg2	Asf1b
Rod1	Casp3
Stat1	Igtp
Cdca2	Ccnb1
Itgam	Tlr7
Skp2	Rrm2
Runx3	Dusp8
Iigp1	Usp18
Cdca5	Esco2
Il24	Il20rb
Ccl5	Fasl
Tlr2	Slc11a1
Bub1b	Plk2
Myc	Timp1
Rxfp3	Ccl5
Spc24	Ccnb1
Bub1b	Plk4
Lcn2	Tlr2
Aif1	Cd52
Parpbp	Bub1
Ccnf	Skp2
Cenpa	Incenp
Cenpa	Dlgap5
Mki67	Fam64a
Nup54	Kpna2
Prim2	Rrm2
Dbf4	Smc4
Birc5	Cdca5
Ptpn7	Dusp4
Ezh2	Dnmt3l
Ifit2	Ifi203
Exo1	Asf1b
Fignl1	Bub1
Irgm2	Dhx58
Mcm5	Mcm3
Clec4a3	Nckap1l
Lcp2	Vav1
Depdc1a	Cdc25c
Rnf213	Parp12
Exo1	Fbxo5
Exo1	Fignl1
Ttk	Kif11
Ccdc99	Gins2
Lrr1	Mcm3
Ncf1	Ncf4
Parpbp	Trip13
Depdc1b	Cdca3
Cdca2	Exo1
Chtf18	Kif11
Fga	B2m
Bub1	Hells
Serpine1	Plaur
C3ar1	Cxcl13
Cxcl9	Psmb8

Igf2	Hdac9
Plek	Cd53
Cdc6	Incenp
Cdca7	Ccna2
Ezh2	Mcm7
Ifit1	Ifi47
Kif23	Tpx2
Smc4	Kif15
Top2a	Fam64a
Rbl1	Mcm7
Lyn	Cd3g
Ccnb1	Cdc25c
C1qc	Fcgr1
Pik3r5	Hck
Fyb	Cd3g
Arhgap11	Racgap1
Mki67	Ccna2
Cxcr6	Ccl5
Themis2	Hck
Kif20a	Mcm4
Gpr65	Cysltr1
Pif1	Fen1
Anln	Pbk
Fen1	Asf1b
Bub1	Spc25
Plk1	Esco2
Plek	Nckap1l
Il18	Il18bp
Cd3d	Cd3g
Mis18bp1	Ska3
Tlr12	Ticam2
Fam64a	Uhrf1
Bub1	Rps6ka2
Mpeg1	C1qc
Kif20b	Plk5
Depdc1a	Ccnb1
Csf2rb	Stat1
Skp2	Birc5
Lig1	Kif11
Exo1	Mcm4
Aspm	Smc4
Ccl5	Cxcl13
Parp12	Rsad2
Nsmce2	Rad51l3
Depdc1a	Nusap1
Bub1	Rad51
F13a1	Serpine1
Niacr1	P2ry12
Cenpa	Mki67
Kif4	Fen1
Dsn1	Hmmr
Map3k8	Cetn3
Gins2	Ska3
Fcgr3	Fcer1g
Dlgap5	Tpx2
Ska1	Cenpn
Cd68	Timp1
Skp2	Rbl1
Hells	Cdt1
Ncf4	Tyrobp
Ptprc	Itgb2
Isg15	Irf7
Plek	Ms4a6d
Depp	Ucp3
Cenpn	Gins1
Psmb9	Ubd
Tacc3	Mcm3
Cenph	Rad51
Rasgrp1	Skap1
Gpr65	Ccl6
Rps3a	Rpl19
Rrm2	Cdt1
Cenpa	Hells
Ect2	Racgap1
Anln	Nek2
Myog	Fgf6
Ppapdc3	Tmem38a
Ccnb1	Fam64a
Rad51	Hmmr
Cysltr1	Ghsr
Igsf6	Themis2
Rad54l	Trip13
Fcgr3	Oscar
Ms4a4c	Pyhin1
Mis18bp1	Uhrf1
Ms4a4c	Rtp4
Cenpq	Casc5
Samd9l	Calca
Fcer1g	Tyrobp
Orc1	Topbp1
Cdca3	Spc25
Rtp4	Irf7
Ezh2	Myc
Mcm5	Cdc7
Cdca2	Hells
Itgam	Fcgr2b
Kcnn4	Kcne3
Aspm	Nek2
Birc5	Hells
Parpbp	Ska3
Cxcl13	Ccl9
Pdcd1	Cd3g
Cxcl9	Ccl2
Gins1	Plk4
Ccdc99	Casc5

Cx3cr1	Ccl9
Psmb9	Parp9
Depdc1a	Cdc6
Fcgr3	Abi1
Depdc1b	Kif11
Cdca5	Fen1
Nusap1	Cdc25c
Ska1	Nek2
Kif20b	Esco2
Cenpq	Spc25
Asph	Casq1
Vcam1	Irf7
Mis18bp1	Pbk
Ednrb	F2rl1
Cdc6	Apaf1
Ddx60	Ifih1
Phf11d	Ifi47
Skp2	Mcm7
Lyn	Epha2
Cd3d	Ptpn6
Cdc25c	Plk5
Parp9	Epsti1
Mthfd1l	Gmps
Ect2	Arhgap11a
Gbp7	Ifi203
Kif20b	Mis18bp1
Slc15a3	Itgb2
Mcm10	Prim2
Birc5	Cdt1
Tap1	Irgm2
Cenpa	Mcm6
Mcm5	Asf1b
Ccnb2	Spc25
Prc1	Bub1b
Dbf4	Top2a
Itpr3	Gnai3
Prkcb	Gnai3
Fen1	Pola1
Lig1	Birc5
Exo1	Cenpn
Prc1	Aspm
Adam10	Timp1
Lepr	Npy
Tpx2	Cdca3
Kif20b	Ccnb1
Usp18	Gmps
Irgm2	Usp18
Mcm4	Mcm7
Ppip5k2	Asf1b
Nek2	Plk4
Cdkn1c	Skp2
Kpna2	Asf1b
Nusap1	Cenpn
Dsn1	Casc5
Thbs1	Myc
Cdc6	Plk1
Oasl1	Ifih1
Fam64a	Asf1b
Cxcl2	Cxcl5
Cenpi	Fbxo5
Il24	Stat1
Phf11d	Dhx58
Cxcl9	Cxcl13
Fcer1g	Ms4a6d
Grip1	Cdh4
Parpbp	Pbk
Ccnb2	Cdca3
Cdca2	Asf1b
Lyn	Vav1
Srgn	Timp1
Dlx4	Fgf6
Ttk	Gins1
Themis2	Vav1
Stat1	Irf7
Fndc5	Ucp3
Gbp2	Psmb8
Cdc25c	Rps6ka2
Mcm6	Kpna2
Cdca2	Plk4
Ska3	Rrm2
Clspn	Prim1
Srsf11	Cstf3
Ptpn22	Lpxn
Clec4d	Ms4a6d
Smc4	Bub1
Gsg2	Sgol2
Mis18bp1	Bub1
Kif20a	Prim1
Mad2l1	Mki67
Wasf2	Nckap1l
Usp18	Zbp1
Ncapg2	Pbk
Prc1	Rad51
Hdac9	Fam60a
Camk2b	Grin1
Cdc25c	Bub1b
Cxcl5	Ccl2
Hist2h3c2	Hist1h2ao
Nusap1	Anln
Dsn1	Incenp
Psmb9	Bub1b
Stat1	Rsad2
Prim1	Cdca5
Coro1a	Arhgdib
Prc1	Esco2

C3ar1	Ccl6
Dsn1	Tacc3
Fcer1g	Btk
Smc4	Ccnb2
Hells	Fen1
Exo1	Ccnb2
Herc6	Rsad2
Ccnb1	Fbxo5
Atad2	Spc25
Sgca	Sgcg
Dbf4	Plk1
Fcgr1	Fcgr2b
Aspm	Parpbp
Slc16a7	Emb
Cenpi	Fam64a
Lrr1	Fbxo5
Tyrobp	Vav1
Lig1	Asf1b
Kif2c	Kpna2
Cxcl2	Fpr1
Lig1	Mki67
Clspn	Cenpk
Isg15	Gbp1
Orc6	Gins1
Cxcl1	Ccl7
Sgol2	Cdca5
Ch25h	Cyp7b1
Ccne2	Mcm4
Cdc6	Hmmr
Stat1	Vcam1
Ifit2	Gbp3
Ccnb1	Tpx2
Aldh3a2	Gatm
Capzb	Actr3
Rac2	Tyrobp
Anln	Esco2
Esco2	Asf1b
Retnlg	Tlr2
Ptprc	Emr1
Cdkn1c	Myc
Hells	Trip13
Kif20b	Pbk
Ezh2	Runx3
Birc5	Pbk
Plcg2	Rac2
Cdca2	Cenpn
Aif1	Cybb
Slc16a7	Slc2a3
Gpr18	Ccl6
Anln	Trip13
Abi1	Vav1
Wnt9a	Frzb
Fignl1	Hmmr
Dsn1	Rad51
Cdkn3	Cdca5
Ifit1	Gbp7
Ttn	Plek
Cish	Stat1
Fcgr1	Hck
Sgol2	Ska3
Mcm4	Kif11
Slc11a1	Cybb
Pola1	Mcm7
Il13ra2	Ptpn6
Kif20a	Mki67
Bub1	Mcm7
Cd2ap	Cdh4
Ubd	Usp18
Ccdc99	Cdca3
Cd53	Lpxn
Mki67	Cenpk
Ddx60	Fam26f
C3ar1	Rgs18
Cenpq	Sgol2
Ska3	Kif11
Stat1	Ptprc
Cd68	Cybb
Mcm3	Donson
Dlgap5	Bub1
Syce3	Las2
Incenp	Uhrf1
Inpp5d	Cd2ap
Isg15	Gmps
Ttk	Cetn3
Spc24	Uhrf1
Stxbp1	Exoc3l2
Gls	Oplah
Lig1	Cdc7
Cox6a2	Ldb3
Cacna1s	Gnai3
Stat1	Casp8
Cenpq	Smc4
Dlgap5	Ccna2
Lig1	Cdc6
Gbp2	Samhd1
Plk4	Shcbp1
Tpx2	Prim1
Kif23	Cenpn
Cenpn	Cdca3
Ptprc	Ly86
Cxcr6	Niacr1
Dlgap5	Rrm2
Gbp2	Cxcl9
Cenpi	Hells
Pld4	Emr1

Kcna7	Kcnc4
Ms4a6c	Fcgr4
Ect2	Top2a
Wasf2	Gmfg
Myl3	Mb
Psmb9	Gmnn
Fcgr3	Clec4a3
Ccdc99	Tacc3
Lair1	Igsf6
Ccnb1	Dlgap5
Ccdc99	Racgap1
Stat1	Rac2
Kif20a	Cenph
Plk1	Gins1
Kif4	Clspn
Bsn	Grin1
Cacna1d	Cacnb1
Troap	Kif23
Cenpl	Spc24
Isg15	Herc6
Trim72	Cav3
Parpbp	Shcbp1
Cenpn	Sgol2
Ncf4	Arhgdib
Clec4d	Slc11a1
Tpx2	Esco2
Ccl7	Ccl4
Orc1	Mcm6
Pla2g4a	Pld4
Lck	Ugcg
Fpr2	Cysltr1
Rxfp3	Rgs18
Wdhd1	Bub1
Lat	Cd3g
Mylpf	Ckmt2
Lck	Stat1
Mad2l1	Ccnb1
Dclre1c	Cstf3
Tpx2	Nek2
Cav3	Srl
Ska3	Spc25
Fignl1	Mcm7
Ctss	Ptprc
Mad2l1	Shcbp1
Plk1	Ccna2
C1qb	Ms4a6d
Foxm1	Nek2
Plk1	Fen1
Ifi204	Rtp4
Cdc6	Plk2
Top2a	Chtf18
Kif20b	Kpna2
Ccl5	Npy
Prc1	Plk5
Msn	Rac2
Herc6	Zbp1
Ptgs2	Ccl2
Cxcl5	Anxa1
Cxcr7	Cxcl1
Kif20a	Kif20b
Ccne2	Rrm2
Cenpa	Smc4
Incenp	Hmmr
Fcgr4	Slc11a1
Pik3r5	Vav1
Ccnb1	Kif23
Mpp3	Lyn
Arhgap30	Coro1a
Tpx2	Asf1b
Abi1	Actr3
Depdc1a	Mki67
Top2a	Uhrf1
Kif11	Uhrf1
Kif2c	Fignl1
Kcnj11	Cav3
Cdc25c	Casc5
Cdca2	Incenp
Cd5	Ptpn6
Mad2l1	Usp1
Trip13	Kif11
Scn1b	Scn4a
Casp8	Gzmb
Itgam	Tlr2
Mcm4	Cdt1
Fpr2	Ccl6
Kif23	Mcm6
Ccna2	Esco2
Ccnb2	Esco2
Gbp3	Rsad2
Top2a	Anln
Incenp	Kif11
Birc5	Ccnb1
Tacc3	Cdca3
Myod1	Myog
Cdc25c	Racgap1
Myl3	Ckmt2
C1qb	Cd53
Ccnf	Bub1
Cxcl9	Il18bp
Orc1	Myc
Plek	Lilrb4
Pgm1	Prps2
Phf11d	Rsad2
Ttk	Mcm10
Birc5	Anln

Clspn	Mki67
Fignl1	Hells
Birc5	Kif2c
Top2a	Asf1b
Hist1h4h	Hist1h2ag
Tlr4	Cybb
Cd48	Hck
Lcp2	Rasgrp1
Dhrs7c	Itgb1bp2
Stat1	Ptpn6
Srl	Ldb3
Ect2	Ncapg2
Tpm2	Tmod1
Sct	Calca
Inpp5d	Sash3
Lig1	Gins1
Tacc3	Cenph
Ppara	Ankrd1
Cdc6	Chtf18
Orc1	Cdc6
Mcm5	Cdca5
Topbp1	Mcm4
Gpr65	Aif1
Dbf4	Plk4
Cenpq	Cdca5
Plk1	Spc25
Cdca7	Mcm7
Mad2l1	Ccnb2
Pola1	Cdt1
Vcam1	Itgb2
Clspn	Plk1
Gpr18	Anxa1
Top2a	Cdca3
Cdc6	Top2a
Wdhd1	Mcm6
Mis18bp1	Hells
Ccne2	Hells
Fignl1	Shcbp1
Ttk	Ccnb2
Cd53	Sell
Stat1	Irgm2
Ect2	Lig1
Igtp	Gbp1
Cdca2	Hmmr
Lilrb4	Fcgr1
Mis18bp1	Kif23
Txk	Ptprc
Birc5	Ttk
Mcm5	Gmnn
Serpine1	Timp1
Cenph	Trip13
Satb2	Bhlhe22
Bub1b	Shcbp1
Cenpi	Cdc25c
Smc4	Hmmr
Ifi47	Irf7
Cdc7	Bub1
Aif1	Emr1
Sgol2	Shcbp1
Cxcr7	Rxfp3
Psmb9	Gbp2
Cxcr7	Sstr5
Ezh2	Birc5
Gins1	Kpna2
Lat	Pik3r5
Cxcl1	Ccl3
Skp2	Mcm4
B2m	Fcgr2b
Gpr18	Ccr2
Dhrs7c	Ttn
Prc1	Cdca5
Dok7	Rapsn
Mad2l1	Mastl
Cdc6	Kif4
Cenpa	Kif23
Tlr2	Irg1
Ccdc99	Mis18bp1
Cytip	Ptprc
Cenpl	Bub1b
Kif4	Ska3
Mki67	Cdca5
Bub1	Nek2
Ncapg2	Fignl1
Esco2	Spc25
Chtf18	Fen1
Troap	Bub1
Csf2rb2	Cish
Mpeg1	Cd68
Nae1	Cetn3
Racgap1	Pbk
Ccr6	Gpr18
Tacc3	Ska1
Cacna1d	Ppp2r3a
Cenpl	Casc5
Mis18a	Cenpi
Fpr2	F2rl1
Ppara	Ucp3
Fcer1g	Fcgr4
Myoz3	Ldb3
Spc24	Kif11
Ddx60	Apaf1
Ctss	Ncf4
C3ar1	Msr1
Ccdc99	Sgol2
Ccnb1	Chaf1b

Nkg7	Gzmb
Ska1	Kif4
Fpr2	Ms4a6d
Trim59	Kif20b
Cxcl2	Sstr5
Cdca7	Fbxo5
Kif2c	Ccna2
Ccr6	C3ar1
Stat1	Fam26f
Cenpi	Ccnb1
Mad2l1	Dlgap5
Prc1	Dlgap5
Cd68	Cd53
Ccnb1	Mcm10
Cxcr7	Fpr2
Ccna2	Fbxo5
Cenpq	Birc5
Blm	Asf1b
Ect2	Cenph
Myc	Kpna2
Ect2	Esco2
Stat1	Racgap1
Ifit2	Ifit1
Parpbp	Mastl
Fcgr2b	Vav1
Ncapg2	Cenph
Smc4	Plk2
Ccnf	Cdt1
Cdca3	Trip13
Exo1	Hells
Ch25h	Sqle
Itgam	Ptprc
Parp12	Ifi44
Gsg2	Incenp
F3	Ccl2
Fen1	Mcm4
Spc24	Mki67
Mcm4	Rrm2
Smc4	Anln
Lck	Mpp3
Mcm3	Plk1
Arhgdib	Itgb2
Arhgef39	Parpbp
Cxcl9	Zbp1
Ms4a6c	Ms4a4c
Ccr6	Sstr5
Mcm5	Spc25
Mcm5	Hells
Myom2	Trim54
Irgm2	Gbp3
Cdca7	Top2a
Prc1	Clspn
Ms4a6d	Ccl6
Kif20a	Lig1
Fga	Cfi
Ifit1	Ifi203
Kif2c	Mcm10
Tlr4	Emr1
Fpr1	Npy
Prc1	Brca1
Fyb	Cd3d
Megf10	Coro1a
Birc5	Gins1
Depdc1b	Cdc25c
Lig1	Gins2
Bub1b	Rad51
Aspm	Ccna2
Smarcd3	Abcb4
Cdkn1c	Ascl2
Ifit1	Zbp1
Coro1a	Tyrobp
Bub1	Asf1b
Kcng4	Kcnc4
Hdac9	Myc
Prim1	Esco2
Plcg2	Prkcb
Cenpi	Bub1
Hk3	Slc11a1
Ncf4	Sash3
Gzma	Gzmb
Ncf4	Ptprc
Lat	Cd244
Ms4a4c	Fpr2
Serpine1	Srgn
Ppara	G0s2
Ptprc	Ccl4
Cenpn	Uhrf1
Dbf4	Mcm6
Prc1	Ccnb2
Cysltr1	F2rl1
Samd9l	Ifih1
Mki67	Myc
Skp2	Kif11
Anxa1	Kiss1
Spc24	Racgap1
Cdc6	Gins2
Fpr2	Rxfp3
Dsn1	Kif2c
Dlx4	Clhc1
Parpbp	Fignl1
Itga7	Lamc2
Lcp2	Lyn
Mcm6	Incenp
Bub1	Prim1
Fignl1	Ska3

Orc1	Pola1
Depdc1a	Fbxo5
Incenp	Trip13
B2m	Stat1
Irf7	Rsad2
Plk5	Kif11
Rxfp3	Gpr18
Clspn	Asf1b
Ccnb1	Lmnb1
Mad2l1	Plk2
Chrm5	Ccl9
Nusap1	Kif11
Mis18bp1	Ska1
Il18	Cd69
Lig1	Kif23
Tacc3	Mcm7
Itga6	Ptprc
Lig1	Uhrf1
Kif20a	Kif15
Hck	Gnai3
Spc24	Tacc3
Orc1	Rad51
Kif2c	Esco2
Prc1	Kif4
Top2a	Kpna2
Cenph	Rrm2
Oasl1	Zbp1
Prim1	Gmnn
Kif20b	Cenpi
Anln	Uhrf1
Birc5	Cdkn3
Ncapg2	Mis18bp1
Top2a	Cdc25c
Cdca2	Troap
Ttn	Myom2
Hdac9	Rbl1
Sstr5	Npy
Top2a	Ddx10
Alox5ap	Nckap1l
Glipr1	Nckap1l
Cxcl2	Ccl6
Map3k10	Map2k6
Gins1	Rad51
Kif20a	Troap
Prim1	Brca1
Nckap1l	Slc15a3
Prkab2	Stradb
Cxcl5	Timp1
Dusp8	Dusp9
Cd69	Sell
Mki67	Esco2
Grip1	Grin1
Ccr6	P2ry12
Sgol2	Esco2
Ryr1	Jph2
Wdhd1	Pola1
Rrm2	Uhrf1
Nek2	Fbxo5
Rasa1	Epha2
Mad2l1	Gins2
AF25170	Clec4a3
Tacc3	Rad51
Mcm4	Pbk
Foxm1	Otx1
Mapk12	Cdc25c
Stat1	Serpina3g
Kif20b	Ccnb2
S100a9	Ly96
Spp1	Racgap1
Rasa1	Birc5
Incenp	Pbk
Racgap1	Uhrf1
Oasl1	Ccl4
Ncf1	Pla2g4a
Shcbp1	Asf1b
Cdc6	Ncapg2
Anln	Ccna2
Racgap1	Brca1
Sstr5	Rgs18
Cenpq	Cenph
Rad54l	Arhgef39
Cd68	Clec4a3
Lilrb4	Igsf6
Fancb	Fancd2
Mcm5	Cenpi
Rrm2	Kpna2
Mcm3	Chaf1a
Prim1	Pbk
Psmb8	Gmnn
Coro1a	Itgb2
Ddx10	Nop58
Cdca2	Birc5
Smyd1	Myog
Gsg2	Plk1
Mad2l1	Cdc25c
Ska1	Hmmr
Mcm5	Chaf1a
Plk4	Trip13
Cenpa	Tpx2
Ccdc99	Pbk
Samd9l	Ifi47
Clspn	Parpbp
Kif20b	Cdc25c
Plek	Gpr65
Cxcl9	Anxa1

Hspb3	Thbs1
Incenp	Cenpk
Ccnf	Orc1
Pla2g4a	Myc
Kif23	Hmmr
Ghsr	Anxa1
Lig1	Fen1
Ppara	Tbxas1
Isg15	Ifi44
Cotl1	Coro1a
Ska1	Esco2
Lck	Themis2
Lck	Ikzf1
Parp9	Ifit2
Foxm1	Ccna2
Myom1	Trim54
Cenpi	Racgap1
Spc24	Gins1
Fancd2	Topbp1
Cenpa	Ccnb2
Cd63	Abcb4
Ncapg2	Cenpk
Arhgap15	Rab8b
Cdca2	Tacc3
Arpc1b	Arpc5
Ccne2	Rbl1
Prim1	Hmmr
Samd9l	Rsad2
Cdca5	Trip13
Fignl1	Mcm6
Cenpa	Asf1b
Ccne2	Bub1
Depdc1a	Aspm
Trip13	Hmmr
Ccnf	Plk5
Ncf4	Slc15a3
Mcm5	Orc6
Ptpn22	Hck
Ccr5	Ccr6
Wdhd1	Mcm7
Top2a	Ccnb2
Cenph	Bub1b
Stat1	Fcgr1
Rad54l	Troap
Mki67	Bub1
Pla2g4a	Grin1
Casp8	Apaf1
Nusap1	Casc5
Usp18	Psmb8
Lig1	Bub1b
Parp9	Usp18
Plk4	Pbk
Incenp	Shcbp1
Ncapg2	Bub1b
C1qb	Ccl9
Alox5ap	Clec4d
Atp1a4	Frk
Lrr1	Gins2
Top2a	Rrm2
Ncapg2	Bub1
Vcam1	Cdh4
Ezh2	Smc4
Dlx1	Dlx2
Ect2	Rrm2
Ccnb1	Cdt1
Fbxo5	Gmnn
Msr1	Ms4a6d
Mcm5	Plk4
Ddx60	Igtp
Foxo6	Myc
Parp12	Dhx58
Clta	Kif4
Bub1b	Kif23
Cdca2	Depdc1b
Lrr1	Gsg2
Ms4a6c	Clec4n
Ccne2	Cdt1
Foxm1	Ska3
Stxbp1	Itpr3
Bub1b	Psmb8
Smc4	Kif23
Ttn	Mb
Niacr1	Fpr1
Epcam	Myc
Clspn	Cdca3
Cdc6	Cdkn3
Trim59	Ccnb1
Cenph	Gins1
Prdm1	Myc
Cdc7	Trip13
Sgol2	Hells
Ccnb2	Rbl1
Kif20b	Atad2
Bub1	Kpna2
Cacna1s	Mylpf
Arhgap11	Ska3
Cenpa	Ezh2
Prc1	Ncapg2
Cdkn3	Ccnb2
Fcgr3	Actr3
Gins1	Prim1
Lrr1	Ttk
Bub1b	Mcm6
Gins2	Plk1
Lyn	Btk

B2m	Vcam1
Gbp1	Mx1
Mcm3	Prim2
Mx1	Rsad2
Epha2	Hck
Gsk3a	Myc
Cysltr1	Rgs18
Mpeg1	Fcgr4
Ccne2	Plk4
Rasa1	Epha6
Psmb9	Stat1
Ttk	Prim1
Casp3	Rad51
Mcm3	Cdt1
Plk4	Kpna2
Arhgef39	Hmmr
Orc1	Atad2
Kif20a	Mcm5
Lrr1	Plk4
Gpr18	Niacr1
Birc5	Chtf18
Kif23	Ccna2
Pik3c2a	Pik3r5
Rtp4	Slfn2
Tacc3	Foxm1
Lig1	Trip13
Cenph	Sgol2
Smc4	Racgap1
E2f8	Kif11
Ctss	Gpr65
Mrgprh	Emb
Slc11a1	Msr1
Mad2l1	Fam64a
Myo1f	Coro1a
Kif2c	Cdca3
Depdc1a	Ccnb2
Cenpa	Ect2
Fyb	Skap2
Camk2b	Cacnb1
Itgam	Stat1
Stat1	Runx3
Foxo6	Sgk3
Tap1	Ifi47
Chaf1b	Asf1b
Ect2	Depdc7
Chtf18	Cdca5
Mis18bp1	Spc25
Mpeg1	Cd53
Ect2	Uhrf1
Top2a	Kif2c
Fcgr3	Ms4a6d
Ect2	Birc5
Bub1b	Mki67
Irgm2	Ifi44
Ttk	Cenpn
Ncapg2	Mcm4
Dsn1	Birc5
Il18	Pycard
Pfkfb1	Hk3
Aspm	Racgap1
Irf8	Cybb
Bub1	Shcbp1
Mastl	Kif11
Arhgap11	Mki67
Cenpa	Cenpl
Gmps	Nop58
Samhd1	Parp14
Btk	Ptprc
Nusap1	Tpx2
Cxcr6	Npy
Rad54l	Ncapg2
Kif23	Mcm10
Ctss	Cd52
Fcgr3	Ncf4
Cdkn3	Anln
Lrr1	Tpx2
Ccne2	Uhrf1
Pbk	Asf1b
Arhgef39	Pbk
Arhgap30	Arhgdib
Usp18	Ifih1
Anxa3	Anxa1
Diap3	Wasf2
Prc1	Chtf18
Kif20b	Ttk
Mpeg1	Cd52
Ccna2	Pola1
Cdca2	Kif23
Cenpa	Bub1
Arhgap15	Rac2
Kif11	Spc25
Gins2	Fen1
Foxm1	Tpx2
Rtp4	Gbp3
Lcp2	B2m
Rad54l	Kif2c
Depdc1a	Lrr1
Aspm	Tpx2
Ccdc99	Top2a
Kif4	Mcm6
Plk2	Kpna2
Cdc6	Cenph
Mapk12	Dusp4
Smc4	Plk4
Tacc3	Cdc25c

Tpx2	Bub1
Clspn	Dlgap5
Dusp4	Vcam1
Mcm5	Wdhd1
Lpxn	Ptpn6
Fcer1g	Pld4
Rad51	Frk
Mcm3	Fignl1
Cenph	Nusap1
Top2a	Fignl1
Cdc6	Anln
Tlr2	Ly96
Cxcl1	Cxcl13
Cdc6	Rbl1
Kif2c	Mis18bp1
Tacc3	Sgol2
Kif20b	Tpx2
Themis2	Tyrobp
Stat1	Ifih1
Bub1b	Esco2
Kif20a	Ska3
Top2a	Ttk
Mad2l1	Plk5
Orc1	Cdca5
Birc5	Mki67
Niacr1	Anxa1
Clec4a3	Cd48
Bub1	Gmnn
Ccne2	Plk2
Plk5	Ccnb2
Bub1b	Pbk
Ect2	Ttk
Ddx60	Rtp4
Ccnb2	Fbxo5
Tlr2	Clec4n
Casp3	Racgap1
Spc24	Kif4
Mastl	Sgol2
Mis18a	Ncapg2
Ms4a6c	Fcer1g
Fignl1	Cdca5
Bub1b	Troap
Fcgr3	Cd68
Cd69	Ccl4
Depdc1a	Ska3
Fpr1	Anxa1
Kif20a	Ccnb1
Casc5	Pbk
Tyrobp	Ccl6
Tacc3	Hmmr
Ifi47	Iigp1
Ncf4	Fcgr1
Csf2rb	Ptpn6
Ncf1	Prkcb
Arhgap11	Arhgdib
Cdc6	Ccdc99
Ugcg	Degs2
Cenpi	Sgol2
Cdkn1c	Ccne2
Igfn1	Dupd1
Pbk	Trip13
Ccnb2	Cdt1
Ifit1	Rsad2
Psmb9	Ifi47
Cd68	Aif1
Pld4	Slc11a1
Top2a	Esco2
Cenph	Ccna2
F13a1	Timp1
Depdc1a	Kif20b
Ctss	Itgb2
Kcnc1	Kif5a
Mad2l1	Cenpn
Eif2s3y	Eif1a
Ska1	Kif11
P2ry12	Gnai3
Prc1	Mcm3
Prpf39	Prpf40a
Prc1	Ccnb1
P2ry12	Ccl6
Fcgr3	Alox5ap
Dlx1	Clhc1
Kif20b	Incenp
Orc1	Fignl1
Mcm5	Cenpn
Rac2	Pik3r5
Tacc3	Prim1
Evi2a	Themis2
Cenpi	Cenpn
Cxcl9	Ccl9
Arpc1b	Wasf2
Cd53	Arhgdib
Cenpa	Dsn1
Cenpl	Mad2l1
Prkcb	Lyn
Irf8	Myc
Ncf4	Ms4a6d
Dlgap5	Plk4
Ptprc	Cd52
Ghsr	Ccl6
Lig1	Cenpn
Mcm5	Ttk
Cxcl1	Gnai3
Lck	Lyn
Gbp7	Iigp1

Ccr2	C3ar1
Atad2	Pbk
Racgap1	Spc25
Cdca2	Nek2
Tacc3	Aspm
Cenpn	Cdca5
Clec4a3	Tyrobp
Dlgap5	Kif15
Fcgr4	Ms4a6d
Cdca7	Mcm6
Cxcr7	Cxcl13
Skp2	Plk4
Blm	Topbp1
Irgm2	Rsad2
Exo1	Myc
Cyp7b1	Sqle
Arhgap11	Kif23
Tyrobp	Itgb2
Top2a	Mcm6
Tlr4	Lyn
Ska1	Dlgap5
Tlr2	Ptprc
Arap2	Arhgdib
Ccna2	Sgol2
Ccdc99	Parpbp
Frk	Plaur
Spc24	Mcm7
Cenpl	Cenpi
Wasf2	Skap2
Depdc1a	Trip13
Chtf18	Ccnb2
Ttk	Shcbp1
Camp	Fpr2
Myc	Brca1
Ms4a6c	Ly86
Cdc6	Gmnn
Nek2	Hells
Birc5	Cenph
Rad51	Kif11
Orc1	Fbxo5
Pik3c2a	Rac2
Psmb9	H2-M3
Dlgap5	Casc5
Ccnb2	Gins2
Parp9	Ifih1
Ncapg2	Nusap1
Rasgrp1	Vav1
Evi2a	Ptprc
Il24	Osmr
Ddx60	Stat1
Plk1	Terf1
Foxm1	Rrm2
Clspn	Hells
Prkcb	Rac2
Psmb9	Iigp1
Ryr1	Casq1
Cenpn	Pbk
Mad2l1	Ska1
Prf1	Cd3g
Mki67	Fbxo5
Ect2	Kif15
Depdc1b	Bub1b
Nek2	Hmmr
Parpbp	Cenpn
Mki67	Brca1
Fpr1	C3ar1
Lig1	Cenpi
Ncf1	Itgam
Fcgr3	Lyn
Cdc6	Kif23
Racgap1	Mcm4
Ifit1	Tlr7
Lat	Ptprc
Foxm1	Racgap1
Cxcl2	Niacr1
Ccnb1	Plk1
Plcg2	Itpr3
Sct	Avpr2
Asb10	Hhatl
Cdkn1c	Orc1
Stat1	Hck
Skp2	Cdca5
Cdkn3	Bub1
C1qb	C1qc
Hells	Fbxo5
Cxcr7	Npy
Ncf4	Pld4
Ppara	Acsl5
Dusp8	Dusp4
Cxcl9	Cxcl5
Rtp4	Herc6
Hdac9	Irf8
Gpr18	Rgs18
Wdhd1	Fen1
ENSMUS	Odf3l2
Smc4	Hells
Cenpi	Clspn
Spp1	Runx3
Parpbp	Uhrf1
Klrd1	Ptprc
Rtp4	Psmb8
Nusap1	Asf1b
Kif20a	Tpx2
Psmb9	Orc1
Plcg2	Pla2g4a

Psmb8	Cdt1
Igsf6	Emr1
ENSMUS	Birc5
Fpr2	Irg1
Fen1	Kif11
Cenpi	Top2a
Actc1	Myo1f
Ect2	Spc25
Cdc7	Esco2
Gbp7	Parp14
Cdkn1c	Myod1
Cenph	Prim1
Kif20b	Ncapg2
Kif20a	Ccnb2
Rad51	Esco2
Gbp2	Ifi47
Ms4a6c	Clec4a3
Ccne2	Plk1
Cdca3	Fam64a
Aspm	Ska3
Kif4	Trip13
E2f8	Rrm2
Cxcl1	Ccl2
Mad2l1	Psmb8
Depdc1a	Cdkn3
Kif20b	Bub1
Fam64a	Hmmr
Lcp2	Rac2
Mpeg1	Ptpn6
Plk1	Uhrf1
Casc5	Hells
Sgol2	Prim1
Tyrobp	Cd52
Lig1	Racgap1
Pif1	Chtf18
Stat1	Samhd1
Cenpn	Plk4
Cenph	Kif2c
Prpf39	Hnrnpa2b1
Lig1	Rrm2
Oasl1	Fcgr1
Terf1	Brca1
Sstr5	C3ar1
Tacc3	Anln
Ryr1	Casq2
Top2a	Depdc1b
Fcgr3	Ly86
Hist1h4i	Hist1h2ab
Dlgap5	Kif23
Fcgr3	Nckap1l
Cdca5	Rrm2
Myo18b	Mylpf
Kif2c	Shcbp1
Mcm3	Atad2
Ptgs2	Hpgds
Irf8	Ptprc
Chtf18	Incenp
Tyrobp	Emr1
Ctss	B2m
Usp1	Ncl
Kif23	Fbxo5
Tacc3	Clspn
Plk1	Psmb8
Ryr1	Cacna1d
Ccna2	Gins1
Rad54l	Esco2
Wdhd1	Rad51
Birc5	Kif15
Cdca7	Prim1
Ccnf	E2f8
Itpr3	Tas1r1
Ryr1	Homer2
Prdm1	Tlr2
Ncapg2	Plk4
Kif2c	Cenpk
Anxa1	F2rl1
Lcp2	Fcer1g
Smyd1	Myod1
Ect2	Clspn
Nusap1	Ccna2
Mis18bp1	Clspn
Evi2a	Ms4a6d
Ccnb1	Plk2
Tacc3	Kif2c
Tlr4	Serpinb9b
Top2a	Gmps
Chtf18	Mcm4
Mis18bp1	Rad51
Stat1	Vmp1
Exo1	Ccna2
Cenpn	Fen1
Rrm2	Hmmr
Orc1	Top2a
Ptpn2	Lat
Tacc3	Casc5
Myod1	Rbl1
Ms4a6d	Clec4n
E2f8	Hmmr
Sgk3	Gsk3a
Nusap1	Kif4
Gpr18	Sell
Ect2	Spc24
Xcl1	Rgs18
Cenpq	Bub1
Fancb	Blm

Mapk12	Dusp8
Gbp7	Gbp3
Cdca7	Gins2
Cacna1s	Gngt2
Itgam	Sell
Fen1	Mcm7
Igtp	Oasl1
Ccnb1	Ttk
Cxcr6	Fpr1
Myom1	Itgb1bp2
Ms4a7	Aif1
Oasl1	Dhx58
Kif20b	Nusap1
Gins1	Shcbp1
Kif20b	Hmmr
Ttk	Uhrf1
Mad2l1	Parpbp
Lst1	Aif1
Cxcl1	Rgs18
Mcm3	Kif4
Bub1	Fam64a
Rnf213	Ifi47
Fignl1	Plk1
Ncapg2	Clspn
Arhgap11	Obscn
Arhgap11	Top2a
Ccne2	Skp2
Fcgr4	Oscar
Ppara	Ccl2
Kif2c	Racgap1
Cst13	Cst6
Ccna2	Mcm6
Smc4	Fbxo5
Obscn	Depdc7
Srsf11	Tra2a
Orc6	Pola1
Arhgap11	Mcm4
Ifit1	Gbp3
Rtp4	Usp18
Ifi44	Irf7
Rtp4	Parp12
Mpeg1	Tyrobp
Clspn	Chtf18
Gbp2	Igtp
Ccnb2	Mcm7
Ccr5	Cxcl1
Ppp2r3a	Cacnb1
Lrr1	Gins1
Lig1	Hells
Bub1	Plk2
Ccnb1	Cdca3
Cxcl9	Rxfp3
Fcgr2b	Actr2
Epha2	Vav1
Depdc1a	Rrm2
Tlr1	Casp1
Blm	Lig1
Mapk12	Myog
Isg15	Mx1
Fcgr3	Vav1
Ms4a6c	Emr1
Depdc1b	Dlgap5
Mad2l1	Gins1
Kif4	Casc5
Birc5	Cdc25c
Gsg2	Pbk
Cenph	Hells
Birc5	Plk1
Cdca2	Rrm2
Pbk	Brca1
Cybb	Emr1
Niacr1	Cxcl13
Ccnb2	Casc5
Nckap1l	Lpxn
Cdca2	Ska3
Plk1	Incenp
Cenph	Ska3
Ccnb2	Kpna2
Bard1	Brca1
Mcm3	Chtf18
Kif2c	Exo1
Kif20b	Mcm3
Map4k1	Skap2
Depdc1b	Vav1
Spc24	Cenpi
Actc1	Ldb3
Cenpa	Foxm1
Ctss	Cd48
Cd48	Srgn
Sell	Ltb
Kif20b	Top2a
Spc24	Mcm10
Foxm1	Ttk
Ms4a6c	Tyrobp
Acvr2b	Inhbe
Depdc1a	Dlgap5
Runx3	Cfb
Kif15	Plk1
Ptprc	Cd5
Ccdc99	Ccnb1
Lrr1	Ect2
Ifi47	Parp14
Cdca2	Pbk
Trim59	Mad2l1
Arhgap11	Cenpk

Vgll2	Dlx1
Birc5	Hmmr
Fga	F13a1
Arhgap15	Obscn
Cenpl	Cenpq
Ect2	Ccdc99
Cdc6	Clspn
Noxin	Hmmr
Evi2a	Clec4a3
Rgs18	Kiss1
Chrm5	Kiss1
Tlr4	Ifih1
Npy	Cxcl1
Gmps	Ada
Lck	Lpxn
Kif20a	Cdkn3
Psmb9	Psmb8
Aspm	Trip13
Dbf4	Plk5
Rad51	Mcm6
Clec4a3	Itgb2
Gbp2	Stat1
Ect2	Mis18bp1
Orc1	Chtf18
Fyb	Ptpn6
Mad2l1	Plk4
Plk1	Trip13
Ptprc	Ccl2
Top2a	Mthfd1l
Lrr1	Ska3
Cenpi	Exo1
Herc6	Mx1
Lrr1	Esco2
Lck	Rasa1
Ccnb1	Cenpn
Top2a	Cdt1
Pld4	Nckap1l
Cenph	Pbk
Coro1a	Lpxn
AW11201	Depp
Ect2	Parpbp
Parpbp	Cdca3
Alox5ap	Pld4
Pla2g4a	Gnai3
Cdc25c	Arhgef39
Chaf1a	Mcm7
Adam10	Sell
Rnf213	Oasl1
Ccnb1	Fignl1
Vcam1	Cybb
Gbp7	Igtp
Mad2l1	Kif15
Hrc	Trim54
Rad54l	Fancd2
Gbp2	Gbp1
Arhgap30	Rac2
Birc5	Kpna2
Ppm1l	Ripk3
Fpr1	P2ry12
Mcm5	Cdca7
Tpx2	Racgap1
Capzb	Cd2ap
Lck	Ptpn6
Fcer1g	Vav1
Ptpn2	Stat1
Orc6	Ccna2
Ttn	Itgb1bp2
Cdc25c	Dlgap5
Ccl5	Ccl3
Mcm3	Trip13
Cd53	Cd52
Lrr1	Cdc6
Cdca2	Spc25
Cenpq	Plk1
Top2a	Mcm3
Arhgef39	Plk1
Depdc1b	Anln
Fignl1	Plk4
Ccne2	Mad2l1
Ttk	Cdca5
Mcm4	Fbxo5
Spc24	Birc5
Troap	Hmmr
Lyn	Cd72
Kif4	Kif23
Cd48	Nckap1l
Ccnb1	Prim1
Cdc25c	Kif4
Mapk12	Myc
Fbxo5	Pola1
Lrr1	Cdca5
Ppara	Plin5
Ncoa3	Smarcd3
Top2a	Cenpk
Exo1	Chaf1b
Blm	Hells
Ccnb1	Trip13
Kif4	Ccnb2
Fcgr4	Igsf6
Fpr2	Ccl5
Fpr2	C3ar1
Asns	Gatm
Lrr1	Mad2l1
Ifit2	Rnf213
Lck	Ptprc

Ccnb1	Kif2c
Mis18bp1	Rrm2
Ttn	Cd2ap
Psmb9	Irf8
Incenp	Spc25
Ccdc99	Ncapg2
Irgm2	Ifih1
Havcr2	Hck
Anln	Kif15
Trip13	Cdt1
Lyn	Fcgr2b
Lrr1	Rrm2
Niacr1	Ccl5
Foxm1	Aspm
B2m	Hck
Ccne2	Shcbp1
Prim1	Mcm4
Cdkn3	Bub1b
Kif15	Kif23
Arhgap25	Rac2
Kif11	Cdt1
Actr3	Mx1
Gins2	Cdca3
Csf2rb	Csf2ra
Mapk12	Dusp9
Plk5	Ccna2
Aspm	Mki67
Cenph	Kif4
Clec4a3	C1qb
Gbp3	Irf7
Ms4a4c	Slfn2
Bub1b	Cdt1
Cd68	Itgb2
Naip2	Incenp
Rasa1	Rap2c
Ncf4	Alox5ap
AF25170	Emr1
Bub1b	Hmmr
Esco2	Ska3
Ttk	Kif23
Fcgr1	Aif1
Tyrobp	Hck
Cxcl9	Cxcl2
Ms4a7	C1qc
Kif20a	Mcm3
Mcm4	Asf1b
Exo1	Cenpk
Depdc1a	Casc5
Ezh2	Uhrf1
Aldh3a2	Asns
Ska1	Parpbp
Gpr65	Cd48
Lck	Cd3d
Oasl1	Rsad2
Top2a	Casc5
Mad2l1	Ska3
Cdc6	Nek2
Mki67	Plk1
Pld4	Themis2
Top2a	Dlx4
Depdc1a	Cenph
Mcm5	Rad54l
Klhl6	Sash3
Bard1	Rad51l3
P2ry12	Sstr5
Ccl7	Ccl2
Ccnb2	Incenp
Parp9	Psmb8
Chtf18	Pola1
Fignl1	Asf1b
Mcm3	Cenpn
Neu2	Npl
Plk1	Cenpk
Ctss	Lgmn
Ccnb1	Plk4
Cenpi	Mki67
Mad2l1	Bub1
Bub1	Sgol2
Mcm5	Fbxo5
Ttk	Exo1
Slc11a1	Vav1
Nusap1	Pbk
Cxcr7	C3ar1
Ect2	Brca1
Igtp	Ifi44
Mutyh	Fen1
Irf8	Slc11a1
Orc6	Gmnn
Gpr65	Ghsr
Capn3	Rps6ka2
Lck	Fasl
Sgca	Trim54
Prim1	Shcbp1
Mis18bp1	Plk1
Cacna1s	Rasgrp1
Dlgap5	Exo1
Fpr2	Kiss1
Ifit1	Psmb8
Ccdc99	Spc25
Plk4	Prim1
Ccnb1	Kif15
Tpx2	Plk4
Plcg2	Pi4k2b
Isg15	Rsad2
Itgam	Lyn

Spc24	Cdca3
Rxfp3	C3ar1
Birc5	Clspn
Casp3	Ptprc
Ska1	Pbk
Soat1	Ch25h
Dsn1	Sgol2
Depdc1b	Shcbp1
Ptprc	Vav1
Rad51c	Fancd2
Arhgef39	Mis18bp1
Fcer1g	Slc15a3
Ccne2	Esco2
Ccnb1	Hells
Mcm6	Shcbp1
Stat1	Zbp1
Fcgr1	Ms4a6d
Tacc3	Nek2
Itgam	Tyrobp
Amica1	Cxadr
Adam9	Pacsin3
Ly86	Cd52
Cenph	Incenp
Exo1	Esco2
Cd53	Cybb
Actc1	Hells
Oasl1	Mx1
Ms4a4c	Zbp1
Cenpa	Cenpi
Fignl1	Cenpk
Plk1	Brca1
Ccnf	Hmmr
Myod1	Eif4a2
Ppm1l	Frk
Cenpl	Bub1
B2m	Prf1
Cxcl9	Cxcr7
Spc24	Ccnb2
Kcna7	Kcnh2
Fcgr3	AF251705
Dbf4	Cdca5
Tmem17:	Tlr2
Ccnb2	Mcm6
Ccl4	Emr1
Cxcl2	Cxcl1
Clec4a3	Fcgr1
Cdca5	Mcm7
Bub1b	Spc25
Prc1	Cdca2
Ccne2	Ncapg2
Gbp2	Ifit1
Kif4	Troap
Gins1	Mcm6
Diap3	Anln
Ncf1	Mapk12
Eps15	Fgfr4
Top2a	Lmnb1
Ska1	Sgol2
Mcm3	Mki67
Mcm5	Ect2
Ifi47	Serpina3g
Clspn	Plk4
Cenpa	Atad2
Plk1	Rad51
Blm	Exo1
Igf2	Igf2bp3
Prc1	Mcm6
Kif20a	Ect2
Cdc6	Shcbp1
Fcgr3	Igsf6
Ccdc99	Bub1
Irf8	Gbp1
Wdr43	Ncl
Trp63	Fancd2
Ccnb1	Ska1
Spc24	Asf1b
Ttn	Lmod3
Depdc1b	Parpbp
Nusap1	Prim1
Cdca2	Plk1
Sstr5	Anxa1
Cd68	Tlr2
Rxfp3	Fpr1
Parp9	Ddx60
Mcm3	Hmmr
Plk1	Plk4
Mcm6	Cdca5
Mcm3	Kif11
Mcm3	Kif23
Slc11a1	Hck
Psmb9	B2m
Pacsin3	Mx1
Atad2	Bub1b
Myl3	Ldb3
Ccl5	Cxcl5
Cdca2	Sgol2
Top2a	Troap
Rnf213	Dhx58
Parp14	Zbp1
Lck	Epha2
Nusap1	Smc4
Bub1	Mcm4
Pla2g4e	Pld4
Plk1	Shcbp1
Mcm5	Tacc3

Irf8	Vcam1
Mak	Ccnf
Cenpn	Incenp
Ttn	Hrc
Orc1	Cdt1
Gbp2	Ifih1
Clspn	Uhrf1
Kif20a	Mad2l1
Clec4a3	Gpr65
Cdc6	Cdca5
Oasl1	Vcam1
Spc24	Exo1
Itga7	Thbs1
Arhgap11	Smc4
Cenpi	Asf1b
Ccnb2	Rad51
Prf1	Casp8
Ccr5	Cxcr7
Ifit1	Phf11d
Adam8	Anln
Cdc6	Smc4
Ccna2	Kpna2
Rbl1	Uhrf1
Ccl5	Ccl9
Mlkl	Ripk3
Rap1b	Pik3r5
Map3k8	Krt14
Arhgap11	Mcm3
Stat1	Rtp4
Ddx60	Herc6
Tlr4	Casp1
Niacr1	Sstr5
Ifi44	Dhx58
Rxfp3	Npy
Gins1	Bub1
Cenpl	Incenp
Mcm5	Foxm1
Ccr2	Ccl3
Ttk	Nek2
Rad54l	Bub1
Mapk12	Ptpn22
Chtf18	Ccna2
Ttk	Ska1
Fpr2	Gnai3
Sstr5	Cxcl5
B2m	Irf8
Herc6	Ifih1
Prim1	Pola1
Ifit2	Stat1
Ttn	Tpm2
Casc5	Spc25
Usp18	Ifi44
Skp2	Hells
Dbf4	Cdc7
Cdc6	Casc5
Lcp2	Cd3d
Myo1f	Sash3
Foxm1	Clspn
Serpine1	F3
Cxcl9	P2ry12
Fcgr3	Lilrb4
Mthfd1l	Rad51
Hmmr	Spc25
Racgap1	Hmmr
Ifi44	Rsad2
Herc6	Oasl1
Arhgap11	Rad51
Kif23	Ska3
Cxcl2	Fpr2
Birc5	Gmnn
Kcnj11	Kcne3
Fyb	Rac2
Diap3	Ect2
Orc1	Bub1
Vav1	Itgb2
Gpr65	Cd53
Cenpa	Rrm2
Actc1	Rac2
Tap1	Parp14
Prc1	Foxm1
Ccnb1	Myc
Gpr18	Cxcl5
Gins2	Spc25
Bub1b	Brca1
Prc1	Nek2
Cxcl9	Gpr18
Ddx60	Ifi47
Itgam	Cybb
Exo1	Casc5
Ccnb2	Mcm4
Rad54l	Parpbp
Mis18bp1	Incenp
Birc5	Kif23
Prc1	Mcm4
Ttn	Ldb3
Igf2	AI464131
Fcgr3	Cd53
Cenpn	Racgap1
Parpbp	Sgol2
Cxcl2	Npy
Asph	Casq2
Casp3	Unc5a
Fignl1	Esco2
Gpd1	Gpt
Cdca2	Rad51

Kif4	Dlgap5
Plk1	Sgol2
Mcm6	Asf1b
Sstr5	Ccr2
Ncapg2	Shcbp1
Ctss	Psmb8
Cxcl1	Anxa1
Casp1	Apaf1
Orc1	Orc6
Ifit2	Parp12
Evi2a	Ly86
Lck	Plcg2
Stxbp1	Anxa3
Lpxn	Ly86
Ikzf1	Btk
Casp3	Casp8
Exo1	Brca1
Aspm	Kif23
Lrr1	Ncapg2
Cdc6	Tpx2
Mcm3	Rbl1
Kif15	Tpx2
Plcg2	Inpp5j
Cdkn3	Plk1
Irgm2	Iigp1
Bub1b	Kif15
Parpbp	Tpx2
Ifit1	Oasl1
Mcm5	Prim2
Fpr2	Cxcl13
Rasa1	Epha8
Tlr7	Tyrobp
Arf6	Rac2
Obscn	Srl
Mad2l1	Tpx2
Arhgap11	Gmnn
Orc1	Clspn
Trip13	Mcm7
Cdca2	Esco2
Ccl5	Gnai3
Ttn	Srl
Cenpa	Mis18bp1
Rasa1	Fyb
Spc24	Ska3
Tacc3	Hells
Ms4a4c	Ms4a6d
Cenph	Mis18bp1
Ccne2	Orc1
C3ar1	Npy
Mcm5	Cdc6
Ttk	Fignl1
Fignl1	Gins1
Cdca3	Rrm2
Cenpa	Fam64a
Myc	Rrm2
Ptgs2	Pla2g1b
Lig1	Cdca5
Cenpa	Spc25
Nusap1	Ska3
Dsn1	Cenpi
Cenph	Shcbp1
Lilrb4	Msr1
Cenph	Ccnb2
Top2a	Mcm7
Bub1	Rrm2
Ttk	Cdca3
Cxcl2	Nfkbiz
Lyn	Tyrobp
Cenpa	Mad2l1
Lck	Cd69
Cdkn3	Tpx2
Aspm	Bub1
Plk1	Racgap1
Nusap1	Cdca5
Ifi204	Stat1
Fcgr3	Arpc5
Prc1	Mcm5
Cd68	Mki67
Ccdc99	Ska1
Ccnf	Kif4
Sell	Itgb2
Orc6	Tex30
Gbp2	Parp9
Parp14	Ifi44
Cenpa	Ska1
Lrr1	Nek2
Cdca3	Shcbp1
Phf11d	Usp18
Hrc	Mb
Foxm1	Cdca5
Ccnb1	Mcm7
Ifi204	Ifi44
Cdkn3	Kif4
Aspm	Plk4
Birc5	Foxm1
Cdca2	Fignl1
Ncf4	Itgb2
Mpeg1	Ms4a6d
Lig1	Ccnb1
Stat1	Gbp1
Ifit1	Rtp4
Mpeg1	Slc11a1
Fcgr1	Itgb2
AF25170	Igsf6
Pgm1	Pgm2

Rtp4	Rsad2
Bub1b	Rrm2
Macrod1	Pola1
Rac2	Ptgs2
Cdkn3	Kif23
Kif20a	Arhgef39
Top2a	Pola1
Spc24	Nek2
Wdhd1	Lig1
Satb2	Fam60a
Rad51	Racgap1
Ect2	Ccnb2
Ppm1l	Pbk
Lcp2	Rap1b
Clta	Clhc1
Ect2	Plk4
B2m	Psmb8
Kif20a	Esco2
Arhgap11	Hells
Ttk	Mki67
Mpp5	Frk
Map3k10	Ppm1l
Dbf4	Kif20b
Mpeg1	Rac2
Eps15	Clhc1
Cxcr6	Ccl3
Usp18	Rsad2
Ank1	Chl1
Atad2	Shcbp1
Sell	Emr1
Plek	Tyrobp
C1qc	Emr1
Irf7	Psmb8
Il18	Itgam
Cenpi	Tacc3
Ncf4	Fcgr4
Fcgr3	Aif1
Ncapg2	Cdca5
Igsf6	Slc15a3
Cdc6	Sgol2
Dsn1	Ccdc99
Casp3	Myc
Mki67	Rbl1
Lilrb4	Tyrobp
Mpeg1	Plek
Prc1	Prim1
Mki67	Pbk
Mapk12	Rac2
Prc1	Rrm2
Kif20a	Parpbp
Eps15	Birc5
Mcm5	Lig1
Gins2	Kif11
Cdc25c	Anln
Parp14	Dhx58
Cdca5	Fbxo5
Ccnb2	Cenpn
Mad2l1	Rad51
Fpr2	P2ry12
Ccne2	Cdc25c
Cenph	Parpbp
Hells	Racgap1
C3ar1	Ms4a6d
Msn	Cd48
Depdc1a	Ncapg2
Ifit2	Isg15
Samhd1	Fam26f
Depdc1b	Rac2
Lrr1	Uhrf1
Ttn	Mylpf
Ccna2	Mcm4
Mpeg1	Clec4a3
Blm	Top2a
Ccr5	Cxcr6
Lrr1	Arhgap11a
Exo1	Kif23
Lrr1	Ccna2
Gngt2	Gnai3
Fcgr1	Slc11a1
P2ry12	Rgs18
Mcm6	Fen1
Tlr1	Ly96
Samd9l	Usp18
Lck	Tyrobp
Cdc25c	Trip13
Mcm5	Ccnb2
Cxcl9	Niacr1
Parp9	Iigp1
Lrr1	Fignl1
Spc24	Cenph
Cd2ap	Acap1
Ifi204	Oasl1
Gbp2	Fam26f
Tyrobp	Trem2
Lrr1	Trip13
Chrm5	Cysltr1
Rad54l	Clspn
Lyn	Hck
Stat1	Usp18
Rac2	Gnai3
Blm	Ifih1
Ppara	Smarcd3
Cenpa	Chaf1b
P2ry12	C3ar1
Cenpi	Trip13

Chaf1b	Mcm4
Kif2c	Parpbp
Ifi47	Irf8
Plk4	Spc25
Stat1	Oasl1
Cxcl2	Ccl5
Lrr1	Asf1b
Ccna2	Pbk
Rad54l	Chtf18
Clspn	Fignl1
Cdca2	Kif4
Ppm1l	Hck
Mis18bp1	Kif4
Spc24	Dlgap5
Casc5	Esco2
Casp1	Emr1
Tpx2	Hells
Nusap1	Mki67
Kif20b	Shcbp1
Ccr2	Anxa1
Clspn	Topbp1
Gbp2	Parp12
Rps6ka2	Myc
Lilrb4	Cybb
Casp3	Hspb2
Ect2	Mcm3
Lig1	Mcm6
Sash3	Coro1a
Cenpn	Rrm2
Depdc1a	Bub1
Ska1	Casc5
Orc6	Cdc7
Tpm2	Myl6b
Clec4a3	Ms4a6d
Isg15	Pla2g4a
Ccnf	Mcm3
Cxcl2	Gnai3
Abi1	Actr2
Runx3	Myc
Mcm3	Chaf1b
Fcer1g	Aif1
Ddx60	Zbp1
Cdc25c	Rad51
Inpp5d	Csf2ra
Ncapg2	Prim1
Ccnf	Ccnb1
Pla2g4e	Gnai3
Ncf4	Vav1
Depdc1a	Birc5
Ncf4	Prkcb
Stxbp1	Stx11
Arhgap11	Cdc6
Phf11d	Stat1
Cenpa	Trip13
Cd48	Cd52
Rad54l	Plk1
H2-M3	Psmb8
B2m	Serpinb9b
Cdc6	Ccnb2
Casc5	Cdca3
Ccdc99	Plk4
Nusap1	Fam64a
Kif20b	Mcm6
Kif23	Cdca3
Mcm5	Sgol2
Cd48	Lpxn
Gpr65	Myc
Gbp2	Zbp1
Atad2	Rrm2
Cd53	Ly86
Tpx2	Pbk
Prc1	Incenp
Mcm5	Rrm2
Ncapg2	Anln
Cdc6	Orc6
Top2a	Pbk
Ect2	Sgol2
Incenp	Cdt1
Cenpa	Cdca5
Ifit1	Irf7
Ccnf	Mcm6
Top2a	Fbxo5
Nusap1	Kif15
Niacr1	Ccl9
Casq2	Ankrd1
Ctss	Alox5ap
Cenpa	Kif11
Msn	Srgn
Gbp2	Herc6
Ifit1	Parp14
Cdc25c	Incenp
Ncf1	S100a9
Blm	Chtf18
C3ar1	Cxcl5
Ccdc99	Cdca5
Tuba1c	Plk1
Mcm6	Trip13
Parp14	Gbp3
Cenpq	Bub1b
Rad51c	Fen1
Evi2a	Ncf4
Cenph	Ska1
Il18bp	Zbp1
Plk5	Racgap1
Casc5	Mastl

Kif2c	Fam64a
Lck	Dock2
Top2a	Fen1
Gmps	Prps2
Rxfp3	Ccl9
Smc4	Sgol2
Esco2	Cenpk
Ank3	Chl1
Donson	Mcm4
Anln	Cenpn
Ddx60	Oasl1
Mcm6	Spc25
Kif20b	Trip13
Stat1	Lepr
Rab3a	Grin1
Arhgap11	Shcbp1
Myo1f	Rac2
Tpx2	Cdca5
Cd69	Ldb3
Fignl1	Gins2
Blm	Mcm7
Lcp2	Plek
Ncf4	Aif1
Aqp4	Dse
Pgm1	Hk3
Cenpk	Rrm2
Mis18a	Cenpn
Rxfp3	P2ry12
Myc	Hck
Fcgr3	Itgb2
Mad2l1	Exo1
Cdc25c	Mki67
Gins1	Mcm10
Map3k10	Rac2
Cenph	Exo1
Ccnb1	Ccnb2
Birc5	Fbxo5
Mcm3	Prim1
Ccr6	Anxa1
Kif20b	Asf1b
Cdca2	Ccnb2
Rap1b	Map2k6
Lyn	Pik3r5
Myo1f	Itgb2
Tacc3	Kif23
Cdc6	Rrm2
Cxcl2	Ccl9
Ccna2	Hells
Cdca2	Foxm1
Birc5	Ccna2
Prc1	Plk4
Birc5	Shcbp1
Lipg	Ppara
Racgap1	Rrm2
Ccr5	Ccl3
Arap2	Vav1
Parpbp	Hmmr
Slc11a1	Slc15a3
Nags	Asns
Ezh2	Hells
Ldb3	Mb
Prim2	Prim1
Bub1b	Bub1
Gins1	Uhrf1
Alox5ap	Cd53
Ifi44	Epsti1
Arpc5	Actr2
Sash3	Ms4a6d
Txk	Vav1
Chrm5	Ghsr
Ms4a6d	Ccl9
Ccnb1	Rad51
Mastl	Plk4
Lmod3	Trim54
Prc1	Arhgap11a
AF25170	Ms4a7
Ghsr	Npy
Ncapg2	Cenpn
Gpr65	Rgs18
Arhgef39	Kif23
Foxm1	Cdc25c
Smarcd3	G0s2
Fancd2	Usp1
Prc1	Fen1
Ttk	Fam64a
Skp2	Cdc7
Cdc7	Plk2
Gsk3a	Ppm1l
Cenpa	Nek2
Parp9	Stat1
Itgam	Vcam1
Ptgs2	Vav1
Cdca5	Brca1
Cenpi	Kif4
Ttn	Myom1
Lyn	Lat
Irf7	Dhx58
Sgol2	Fbxo5
Rad51	Incenp
Ntf3	Isl2
Ccr2	Cxcl1
Cdc6	Cdt1
Inpp5d	Pik3c2a
Mki67	Hells
Ect2	Trip13

Rrm2	Asf1b
Hmmr	Kif11
Bub1b	Plk1
Prc1	Spc25
Cdca2	Rad54l
Nap1l1	Ncl
Cenpi	Prim1
Cenpi	Chtf18
Camk2b	Racgap1
Fignl1	Pbk
Ppara	Abcg1
AF25170	Lair1
Tpx2	Sgol2
Kif20a	Cenpn
Kif2c	Aspm
Bub1b	Racgap1
Lilrb4	Clec4d
Rad54l	Bub1b
Fpr1	Ccl5
Smc4	Rad51
Ccdc99	Ska3
Mcm10	Prim1
Mcm5	Kif23
Isg15	Ubd
Mis18bp1	Mki67
Capzb	Coro1a
Mki67	Prim1
Top2a	Cdca5
Cenpq	Cenpi
Birc5	Ska1
Orc1	Ccnb2
Fignl1	Cdca3
Kif2c	Mcm7
Nusap1	Hells
Aspm	Uhrf1
Cxcl9	Iigp1
Nusap1	Aspm
Cdca7	Cdc6
Cdca2	Racgap1
Kif15	Ccnb2
Cenpl	Plk1
Mcm10	Ska3
Orc1	Ccna2
Mki67	Kif11
Ncapg2	Ccnb2
Smc4	Shcbp1
Ccnb1	Cdc7
Mis18bp1	Shcbp1
Tyrobp	Slc11a1
Ccnb1	Bub1b
Arhgap11	Ttk
Racgap1	Esco2
Ednrb	Gnai3
Kif20a	Exo1
Rnf213	Usp18
Gbp3	Psmb8
Cdc6	Mcm4
Gbp1	Fcgr1
Hfe2	Ldb3
Cdca7	Uhrf1
Ptgs2	Casp8
Rnf123	Skp2
Irgm2	Ifi47
Prc1	Trip13
Ccnb1	Brca1
Wdhd1	Esco2
Top2a	Plk4
Ccr2	Ccl2
Depdc1a	Kif11
Gins1	Kif11
Mad2l1	Skp2
Aspm	Spc25
Gins1	Mcm7
Brca1	Mcm7
Cdc25c	Chtf18
Lck	Txk
Aspm	Asf1b
Spc24	Cenpn
Parp9	Rnf213
Kif20a	Anln
Cd48	Emr1
Prc1	Kpna2
Myom2	Ckmt2
Casc5	Fbxo5
Ttk	Sgol2
Gins2	Mcm6
Shcbp1	Trip13
Syce3	Tex11
Cenpl	Spc25
Kif20a	Cdca2
Cd68	Slc15a3
Cdc7	Cdt1
Cdca2	Top2a
Cxcr6	Gnai3
Birc5	Brca1
Cdkn1c	Cdc6
Rab3a	Racgap1
Birc5	Smc4
Mki67	Trip13
Arhgap11	Kif4
Kif20a	Hells
Lck	Lat
Rad51	Prim1
Kif2c	Plk1
Cenpn	Trip13

Cenph	Spc25
Trip13	Fbxo5
Ect2	Mad2l1
Hmmr	Fbxo5
Spc24	Kif2c
Txk	Ppm1l
Ccr6	Npy
Ccna2	Racgap1
Bub1	Cdca5
Parp12	Irf7
Cdc25c	Fam64a
Wasf2	Vav1
B2m	Vav1
Bub1b	Fignl1
Tlr7	Ifih1
Tap1	Igtp
Kif2c	Uhrf1
Pld4	Pla2g1b
Gbp3	Ifi44
Sirpa	Skap2
Arhgap11	Vav1
Ccr5	Ccl6
Acsl6	Lipg
Wasf2	Actr3
Parp9	Herc6
Mcm3	Plk4
Cdc6	Parpbp
Nusap1	Parpbp
Mad2l1	Bub1b
Iigp1	Herc6
Hells	Rrm2
Mcm5	Chtf18
Ccdc99	Mki67
Ifih1	Mx1
Spc24	Cenpq
Cenpa	Kif4
Cdc6	Foxm1
Acsl6	Ppara
Mcm3	Fbxo5
Cdt1	Asf1b
Ncf1	Racgap1
Cdc7	Mcm6
Ppm1l	Fgfr4
Plk1	Hmmr
Mcm5	Atad2
Arap2	Arf6
Ccdc99	Rad51
Ccdc99	Ccna2
Kif20a	Incenp
Ccnb1	Anln
Tacc3	Plk4
Cacna1s	Ppp2r3a
Mcm5	Prim1
Mapk12	Map2k6
Mis18a	Cenpq
Cdt1	Uhrf1
Ryr1	Asph
Top2a	Kif4
Bub1b	Hells
Arpc5	Nckap1l
Ctss	Ms4a6d
Atad2	Cenpk
Msn	Vcam1
Ccnb1	Sgol2
Cxcl9	Fpr1
Prc1	Cdt1
Kif23	Mcm7
Mcm5	Kif4
Rpl39l	Rps3a
Mad2l1	Mcm6
Serpine1	F2rl1
Mcm3	Rrm2
Sgol2	Asf1b
Arhgef39	Kif4
Cenph	Top2a
Ttn	Ankrd1
Tlr4	Ptgs2
Foxo6	Prkag3
Tlr4	Trem2
Fam26f	Zbp1
Rac2	Vcam1
Ccnb1	Mki67
Irg1	Ccl3
Abi1	Arpc5
Use1	F2rl1
Prpf40a	Smc4
Kif2c	Plk2
Bub1b	Cdca5
Ifit1	Parp12
Stat1	Tlr4
Gpr65	Tyrobp
Mastl	Pbk
Rad54l	Uhrf1
Fpr2	Cxcr6
Ezh2	Myog
Clspn	Shcbp1
Niacr1	Gnai3
Kif20a	Kif4
Tacc3	Pbk
Kif2c	Mapre3
Spp1	Fam20c
Orc1	Exo1
Themis2	Itgb2
Ccr5	Rgs18
Fyb	Nckap1l

Cenpn	Ska3
Birc5	Racgap1
Cdc6	Prim1
Rad54l	Pbk
Exo1	Ska3
Ednrb	Ccl9
Prc1	Exo1
Chaf1b	Btg2
Timp1	Ccl2
Ncapg2	Ccnb1
Lig1	Mcm3
Clec4n	Emr1
Cenpq	Mis18bp1
Mis18bp1	Racgap1
Ttk	Plk1
Arhgef39	Esco2
Cd84	Nckap1l
Myc	Dlx4
Hspb3	Abcb4
Ccdc99	Kif4
Ccnb2	Mastl
Prkag3	Gmps
Mad2l1	Birc5
Ccne2	Mcm7
Kif20a	Kif11
Ptgs2	F3
Ect2	Mcm4
Fcgr3	Fcgr4
Ccdc99	Hmmr
Niacr1	Rgs18
Ptprc	Lpxn
Tacc3	Mis18bp1
Casp3	Mapk12
Evi2a	Tyrobp
Slc11a1	Aif1
Kif20a	Cenpa
Lig1	Pola1
Clspn	Cdc7
Rrm2	Gmnn
Map4k1	Hck
Rad54l	Ccna2
Serpine1	Ptgs2
Foxm1	Ccnb1
Ttk	Smc4
Gpr65	Emr1
Spc24	Bub1
Gins2	Cdt1
Foxm1	Plk4
Cdc7	Chaf1a
Cenph	Cdca3
Cenpi	Dlgap5
Txk	Pik3r5
Cxcl2	Rxfp3
Atad2	Asf1b
Hells	Asf1b
Ccr2	Ccl5
Ptpn6	Il7r
Cxcr7	Cxcr6
Mcm3	Pola1
Mad2l1	Pola1
Cd69	Cd5
Cenpa	Depdc1a
Cd68	Fcer1g
Gpr65	Kiss1
Bub1b	Cenpk
Ccnb1	Cenpk
Rxfp3	Gnai3
Lin54	Gins1
Dock2	Vav1
Cd69	Ptprc
C1qb	Emr1
Mad2l1	Fen1
Cx3cr1	Ccl2
Clec4a3	Aif1
Nusap1	Ska1
Herc6	Irf7
Depdc1a	Kif23
Mki67	Asf1b
Rnf213	Rtp4
Fcgr3	C1qc
Lig1	Bub1
Tlr13	Ly96
Gpr18	Fpr1
C1qb	Mrc1
Spc24	Sgol2
Kif20a	Mis18bp1
Cenpn	Asf1b
Mki67	Sgol2
Kif20a	Dlgap5
Rad54l	Lig1
Oasl1	Ifi44
Neurl1a	Asb2
Ccnf	Plk4
Mcm3	Hells
Nusap1	Cenpk
Arhgap25	Ect2
Birc5	Mcm4
Mbp	Dyrk3
Cyp7b1	Lbr
Mcm5	Cdt1
Sgol2	Cdca3
Ect2	Ska1
Grhl1	Ppara
Birc5	Asf1b
Bub1b	Mastl

Ccnf	Cdc25c
Mad2l1	Mis18a
Psmb9	Igtp
Tlr2	Map3k8
Cdc25c	Bub1
Plk5	Fbxo5
Depdc1a	Cenpn
E2f8	Ccnb2
Prc1	Tpx2
Dsn1	Racgap1
Cdca2	Casc5
Lmod3	Myom2
Emr1	Hck
Bub1	Incenp
Gmps	Rad51
Serpine1	Ccl2
Plcg2	Trem2
Inpp5d	Fcgr2b
Cxcr6	Cxcl5
Ccr2	Ccl4
Kif4	Esco2
Dlgap5	Parpbp
Procr	Rrbp1
Blm	Orc1
Cdc25c	Asf1b
Cxcl9	Parp14
Ifi44	Mx1
Itgam	Rap1b
Pla2g4a	Pla2g1b
Mcm3	Mcm6
Depdc1a	Cenpk
Smc4	Pbk
Camk2a	Grin1
Gbp2	Usp18
Rad51c	Brca1
Ms4a6c	Ctss
Mcm10	Kif11
Foxm1	Chtf18
Exo1	Mcm6
Rgma	Hfe2
Ect2	Kpna2
Inpp5d	Pik3r5
Mcm3	Bub1
Fxyd5	Ncf4
Foxm1	Plaur
Prf1	Gzma
Mcm3	Brca1
Gins2	Esco2
Mcm5	Nek2
Mad2l1	Arhgef39
Nusap1	Bub1b
Kif20a	Cdca5
Fpr2	Gpr18
Exo1	Cdt1
Ncapg2	Nek2
Ucp3	Npy
Cdc25c	Troap
Ccl5	Ccl4
Smc4	Incenp
Ccnf	Cdkn3
Ncapg2	Kif11
Rad51	Pola1
Nusap1	Fignl1
Spc24	Ccdc99
Npy	Gnai3
Clspn	Ccnb2
Grhl1	Smarcd3
Rac2	Sell
Runx3	Prf1
Ctss	Lilrb4
Blm	Mad2l1
Ccdc99	Kif2c
Lrr1	Cenpk
Cenpa	Mcm7
Kif4	Mcm4
Ptgs2	Tlr2
Bub1	Mastl
C1qb	Tyrobp
Ccl5	Ccl6
Ccl3	Ccl2
Cdca2	Ttk
Parp14	Iigp1
Cd53	Ptprc
Mcm5	Cenpk
Ccnb1	Exo1
Gins1	Trip13
Cenpa	Cenpq
Gpr65	Ms4a6d
Kif20b	Uhrf1
Depdc1b	Uhrf1
Phf11d	Mx1
Birc5	Runx3
Casp8	Fasl
Cxcl9	Fam26f
Itgam	Cd69
Ccdc99	Cdc25c
Ccl7	Ccl3
Ncapg2	Rrm2
C3ar1	Ccl9
Anln	Hmmr
Troap	Pbk
Ccna2	Gmnn
Plcg2	Tyrobp
Dbf4	Mcm10
Sirpa	Ptpn6

Cysltr1	Ccl6
Lrr1	Hmmr
Mpeg1	Cd48
Mad2l1	Spc25
Nckap1l	Ptprc
Dnajc2	Nop58
Rap1b	Itgb2
Clspn	Casc5
Sgk3	Rictor
Dsn1	Cenpq
Mcm4	Trip13
Ccdc99	Kif20b
Lrr1	Bub1b
Gpr18	Sstr5
Fancb	Dhx58
Smc4	Topbp1
Ccl4	Ccl2
Lst1	Fcgr4
Ifit1	Iigp1
Tacc3	Gins1
Rad54l	Fen1
Depdc1a	Ect2
Fen1	Racgap1
Ncf4	Irf8
Ptgs2	Areg
Ccne2	E2f8
Kif20a	Lrr1
Fcer1g	Itgb2
Smc4	Nek2
Syce2	Trip13
Lrrc25	Plac8
Cenpq	Incenp
Depdc1a	Pbk
Birc5	Exo1
Lck	Cish
Myom1	Ckmt2
Hck	Cd3g
Ccnb2	Cdc7
Mcm6	Uhrf1
Kif15	Nek2
Ccnb1	Pbk
Cdc6	Ccnb1
Cenph	Mcm3
Dlx2	Clhc1
Ccr5	Ccl2
Mcm3	Gins1
Fcer1g	Pik3r5
Mcm5	Gins2
Ccdc99	Shcbp1
Birc5	Ptgs2
Cenpn	Ccna2
Gmfg	Coro1a
Cdc6	E2f8
Il18	Stat1
Cysltr1	Kiss1
Lck	Pdcd1
Cd68	Slc11a1
Mcm3	Cdc25c
Myo1f	Lpxn
Depdc1a	Plk4
Lrr1	Parpbp
Itgam	Casp3
Gbp7	Zbp1
Slc15a3	Emr1
Mcm5	Shcbp1
Fpr2	Xcl1
Lrr1	Spc24
Arhgef39	Asf1b
Cdca2	Ect2
Ank1	Spnb1
Prkab2	Mlxipl
Gbp7	Rtp4
Incenp	Mcm4
Dsn1	Spc24
Cox7a1	Cox6a2
Ttn	Sgca
Pbk	Fam64a
Btk	Tlr2
Bub1b	Asf1b
Arhgef39	Cdca3
Prc1	Ska3
Troap	Incenp
Wdhd1	Ccna2
Rac2	Cd53
Orc1	Ccnb1
Cenpn	Kif11
Rap1b	P2ry12
Cd3d	Ptprc
Kif4	Mcm7
Inpp5j	Itpr3
Kif2c	Nek2
Fpr2	Ednrb
Msr1	Emr1
Birc5	Apaf1
Lrr1	Shcbp1
Cx3cr1	Ccl5
Cenpa	Plk1
Lig1	Top2a
Lair1	Ptpn6
Rasgrp1	Cacnb1
Plek	Slc15a3
Ccnb2	Cdca5
Rac2	Racgap1
Cdc6	Cenpi
Bub1b	Kif11

Anln	Fbxo5
Cox6a2	Mb
Kif2c	Ccnb2
Mcm3	Fen1
Casp3	Stat1
Obscn	Rac2
Phf11d	Hells
Top2a	Mastl
Dusp4	Dusp9
Tlr2	Ccl2
Birc5	Myc
Abi1	Nckap1l
Mcm6	Kif11
Ncapg2	Mcm7
Irg1	Ccl7
Chaf1b	Chaf1a
Usp1	Kpna2
Ezh2	Brca1
Fyb	Vav1
Cdc6	Mcm6
Ccne2	Ccnb1
Ttk	Spc25
Cenpa	Rad51
Cenph	Plk1
Parp14	Rsad2
Cenpl	Cdca5
Dock2	Lyn
Ccnb1	Aspm
Tacc3	Mki67
Mad2l1	Cdkn3
Tmem17:	Dhx58
Tyrobp	Tlr2
Fxyd5	Tyrobp
Mx1	Dhx58
Anln	Kif23
Mcm3	Cdca3
Exo1	Mcm7
Isg15	Dhx58
Ccnb1	Gmnn
Lrr1	Kif11
Anxa1	Cxcl13
Kif20b	Kif2c
Mad2l1	Sgol2
Aspm	Rad51
Cd3d	Hck
Themis2	Aif1
Epha6	Lyn
Top2a	Sgol2
Rxfp3	Cxcl1
C1qc	Ccl6
Ccnb1	Ccna2
Chrm5	Fpr2
Trip13	Rrm2
Ncoa3	Myod1
Depdc1a	Mastl
Cenpi	Cdca5
Mis18bp1	Cenpn
Kif4	Ccna2
Cdca2	Kif11
Mcm6	Rrm2
Mis18bp1	Bub1b
Ect2	Tacc3
Racgap1	Kpna2
Rac2	Slc11a1
Fignl1	Ccnb2
Ccnf	Kif23
Mis18bp1	Cdca5
Rad54l	Top2a
Lck	Skap1
Arhgap11	Pbk
Cacna1s	Camk2b
Tlr2	Ccl4
Kif4	Plk4
Ptpn6	Cd3g
Pld4	Fcgr1
Prc1	Shcbp1
Bub1	Brca1
Parpbp	Ccna2
Ikzf1	Myc
Cxcr6	Xcl1
Ccnf	Cdc6
Cdkn3	Dlgap5
Npy	Cxcl5
Aspm	Cdc25c
Depdc1a	Spc25
Parpbp	Mcm10
Mki67	Rrm2
Kif23	Mki67
Wdhd1	Mcm3
Shcbp1	Fam64a
Ect2	Arhgap15
Top2a	Parpbp
Fen1	Rad51l3
Mus81	Dhx58
Birc5	Aspm
Ryr1	Cav3
Smc4	Kif11
Top2a	Ska1
Hist2h3c2	Chaf1b
Cxcr6	Cxcl13
Mcm5	Anln
P2ry12	Anxa1
Foxm1	Pbk
Stat1	Herc6
Arhgap15	Vav1

Gbp2	Irgm2
Top2a	Incenp
Ablim2	Rac2
Igtp	Gbp3
Anln	Asf1b
Aspm	Sgol2
Fpr1	Cxcl1
Clspn	Cdt1
Cxcl5	Gnai3
Parp14	Usp18
Mki67	Mcm6
Dlgap5	Mcm10
Sgca	Cav3
Sash3	Lpxn
Birc5	Sgol2
Exo1	Gins1
Racgap1	Fbxo5
Top2a	Kif23
Kif20b	Ska3
Cenph	Fignl1
Ctss	Mpeg1
Plk4	Fbxo5
Smc4	Ccna2
Cdc6	Pola1
Lcp2	Fyb
Myo1f	Vav1
Spc24	Gins2
Depdc1a	Shcbp1
Ect2	Plk1
Troap	Tpx2
Ifi47	Psmb8
Orc1	Hells
Trnt1	Dnttip2
Exo1	Bub1
Fcgr3	Hck
Mcm3	Spc25
Ncapg2	Racgap1
Fignl1	Degs2
Mcm10	Mcm6
Arhgef39	Kif11
Ccr5	Niacr1
Csf2rb2	Ptpn6
Cep170	Nek2
Skap2	Lyn
Foxm1	Anln
Kif4	Bub1
Spc24	Top2a
Ect2	Cdca3
Tap1	Psmb8
Cenpi	Kif2c
C3ar1	Anxa1
Ccr5	Ccl4
Ncf4	Cd48
Cd48	C1qc
Alox5ap	Ly86
Cdca5	Mcm4
Ska1	Uhrf1
Gins1	Rrm2
Mad2l1	Racgap1
Parpbp	Bub1b
Casp1	Eif4a2
Parpbp	Ccnb2
Prc1	Asf1b
Kif20a	Prc1
Lck	Mx1
Cdc25c	Cenpn
Cdca7	Cdt1
Ccnb2	Hells
Mcm6	Mcm7
Tacc3	Brca1
Lilrb4	Slc15a3
B2m	Ptprc
Mcm5	Mcm10
Fcgr1	Vcam1
Cdc6	Plk4
Dsn1	Pbk
Cxcl1	Ccl6
Phka1	Phkg1
Arhgap11	Mcm6
Lrr1	Ccnb1
Hells	Spc25
Fpr2	Ccr2
Diap3	Abi1
Chtf18	Cdt1
Birc5	Fignl1
Sh3bgrl	BC005537
Spc24	Trip13
Retnlg	Serpine1
Stat1	Gbp7
Kif2c	Ska3
Cenpi	Parpbp
Wasf2	Rac2
Clspn	Bub1b
Lyn	Sell
Cdca5	Kif11
Mcm5	Mcm6
Aspm	Kif15
Igtp	Usp18
Cx3cr1	Xcl1
Fcgr1	Ly86
Tacc3	Smc4
Kif11	Gmnn
Obscn	Ldb3
Kif2c	Anln
Nusap1	Dlgap5

Ttk	Kif2c
Cdca2	Cdkn3
Mus81	Fancd2
Mad2l1	Mcm4
Cdkn3	Hmmr
Niacr1	Ccr2
Cxcr7	Gnai3
Emr1	Ccl2
Itpr3	Btk
Fpr1	Gnai3
Casc5	Hmmr
Mad2l1	Nek2
Tacc3	Arhgef39
Tyrobp	Clec4n
Hells	Ska3
Ncf4	Ptpn6
Samd9l	Parp9
Kif20a	Plk1
Clspn	Mcm6
Fcer1g	C1qb
Lck	Fcer1g
Depdc1a	Hells
Ttk	Clspn
Anln	Rad51
Itga7	Itga6
Ccnb2	Tpx2
Ubd	Psmb8
Ccr5	Sstr5
Depdc1a	Parpbp
Cenpn	Shcbp1
Kcna7	Kcng4
Birc5	Bub1b
Hck	Itgb2
Cysltr1	Ccl9
Herc6	Gbp3
Tuba8	Mapre3
Ifi47	Gbp3
Spc24	Tpx2
Kpna2	Gmnn
Phf11d	Ifi44
Cenpi	Pbk
Cxcl2	P2ry12
Mus81	Ifih1
Fcgr3	Emr1
Blm	Pif1
Tacc3	Cdca5
Tacc3	Kif11
Rad54l	Shcbp1
Lcp2	Txk
Terf1	Fen1
Prc1	Plk2
Ccna2	Cdca5
Txk	Cd3d
Arhgap11	Ccdc99
Tpx2	Fen1
Srl	Ckmt2
Ghsr	Rgs18
Parp9	Igtp
Dbf4	Mcm5
Fcgr3	C1qb
Kif4	Gins2
Rbl1	Hells
Plek	Cd48
Ccna2	Spc25
Cenpi	Ska3
Gsg2	Cdca5
Ttn	Hspb3
Mcm5	Kif11
Xcl1	Ccl9
Hist2h3c2	Hdac9
Cdc6	Kif2c
Racgap1	Shcbp1
Cenpn	Plk1
Cenpq	Plk4
Ifi47	Oasl1
Bub1	Cdca3
Mki67	Spc25
Cdc6	Cdc25c
Mis18bp1	Hmmr
Parp14	Ifih1
Hist2h3c2	Hist1h2ag
Gbp2	B2m
Ttk	Mastl
Lpxn	Hck
Cxcl9	Gbp7
Kif2c	Hmmr
Gbp2	Oasl1
Cdca7	Bub1b
Mis18a	Mis18bp1
Sstr5	Ccl6
Oasl1	Psmb8
Chtf18	Mcm6
Trip13	Uhrf1
Ect2	Prim1
Cdca2	Gsg2
Tacc3	Nusap1
Cdca3	Ska3
Gpr18	C3ar1
Ccna2	Plk2
Btk	Ptpn6
Ifit2	Phf11d
Cdca2	Bub1
Fcgr3	Tlr4
Smc4	Mcm6
Tlr2	Slc15a3

Hrc	Myl3
Tyrobp	Fcgr1
Cdca2	Tpx2
Coro1a	Ptprc
Srsf11	Hnrnpa2b1
Plk4	Uhrf1
Plk1	Asf1b
Chtf18	Mcm7
Cd68	Ccl2
Ttk	Casc5
Arhgap11	Bub1b
Rbl1	Asf1b
Slc11a1	Clec4n
Rad54l	Rad51l3
Ect2	Arhgap30
Rnf149	Lonrf3
Eif4a2	Myc
Inpp5d	Lpxn
Mad2l1	Gmnn
Cd69	Tlr2
Itgam	Pik3r5
Zbp1	Psmb8
Depdc1a	Anln
Dsn1	Spc25
Ncapg2	Cdt1
Cd63	Timp1
Orc1	Mcm10
Fbxo5	Uhrf1
Kif23	Trip13
Lck	Vav1
Cenpk	Hmmr
Slc15a3	Ms4a6d
Kif4	Sgol2
Csf2ra	Lyn
Ccr2	Rgs18
Smc4	Kpna2
Igf2	F13a1
Ccr5	Cxcl13
Cenpn	Nek2
Ccne2	Kif11
Ccr5	Fpr2
Ifi204	Runx3
Ctss	C1qc
Skp2	Cdc6
Cxcr7	Cxcl5
Cdkn3	Pbk
Ect2	Incenp
Mcm5	Mad2l1
Ttk	Tpx2
C1qc	Aif1
Bub1b	Mapre3
Prkcb	Pdk2
Plk2	Kif11
Plcg2	Ly96
Plek	Hck
Cenpi	Shcbp1
Runx3	Dlx2
Hist1h2al	Top2a
Hist1h4h	Hist1h2ab
Depdc1a	Hmmr
Rad51c	Trip13
Sell	Il7r
Ubd	Gmps
Ccdc99	Plk1
Chaf1a	Uhrf1
Cdc25c	Pbk
Casp8	Eif4a2
Slc11a1	Emr1
Dbf4	Mcm7
Kif20b	Foxm1
Ccl7	Timp1
Ccnb1	Shcbp1
Mki67	Mcm4
Kcne1l	Kcne3
Birc5	Uhrf1
Cdc25c	Fbxo5
Hells	Kif11
Anxa1	Ccl6
Dctpp1	Prim2
Dsn1	Plk1
Itgam	Mrc1
Mcm5	Racgap1
Depdc1a	Nek2
Trp53inp2	Vmp1
Sgol2	Hmmr
Mki67	Nek2
Gpr65	Ptprc
Ccr6	Ccl5
Myom1	Mybph
Cxcr6	Cxcl1
Parpbp	Plk1
Ifit1	Dhx58
C1qc	Tyrobp
Kif11	Pola1
Ccnb1	Gins2
Cdca5	Ska3
Rac2	Cybb
Smc4	Mcm4
Mis18bp1	Trip13
Vcam1	Frk
Arhgap11	Rrm2
Bub1b	Ska3
Top2a	Trip13
Chrm5	Ccl6
Nek2	Cdca3

AF25170	Clec4n
Abi1	Fcgr2b
Ccr6	Fpr1
Ctss	Clec4a3
Depdc1b	Sgol2
Cenpa	Nusap1
Cdc6	Fignl1
Dlgap5	Cdca5
Kif2c	Incenp
Fcgr1	Irf7
Cx3cr1	Ccl7
Prc1	Mad2l1
Ttn	Casq2
Trp63	Rbm38
Racgap1	Trip13
Kif2c	Rad51
Ska1	Tpx2
Kif2c	Mki67
Igsf6	Tyrobp
Nusap1	Cdca3
Fen1	Trip13
Cenph	Gins2
Atad2	Uhrf1
Ect2	Fbxo5
Smc4	Plk1
Ptpn2	Mapk12
Dlgap5	Shcbp1
Racgap1	Vav1
Tuba1c	Rilp
Ccl6	Kiss1
Prc1	Kif20b
Kif20b	Fignl1
Ifit2	Gbp1
Bub1	Esco2
Lig1	Incenp
Rad51l3	Brca1
Prim1	Rrm2
Depdc1a	Rad51
Dlgap5	Fbxo5
Clta	Arap2
Lilrb4	Ptpn6
Ska3	Uhrf1
C3ar1	Gnai3
Gins1	Cenpk
Prc1	Sgol2
Ankrd1	Ldb3
Kif20a	Bub1
Cdc6	Kif11
Parp12	Ifih1
Arhgef39	Cdca5
Kif2c	Chtf18
Rtp4	Ifih1
Cdca2	Cenpk
Prc1	Cdkn3
Klrd1	H2-M3
Topbp1	Hells
Mcm3	Bub1b
Foxm1	Kif23
Cxcr6	Ccl4
Mcm5	Kif2c
Cdca7	Mad2l1
Casp3	Eif4a2
Aldh3a2	Akr1a1
Ccna2	Ska3
Lrr1	Rad51
Ccnb1	Incenp
Pbk	Uhrf1
Cxcr6	Ccl9
Ddx10	Dnttip2
Wdhd1	Orc1
Npy	Cxcl13
Atad2	Exo1
Stard4	Sqle
Ccnb1	Spc25
Kif20a	Gins1
Kif20a	Foxm1
Ghsr	Ccl9
Ttk	Incenp
Gzma	F2rl1
Ptgs2	Cybb
Prkcb	Gngt2
Fancb	Topbp1
Casp3	Fasl
Tlr7	Lgmn
Lcp2	Lat
Arhgap30	Obscn
Parpbp	Casc5
Ccnb2	Plk1
Myo18b	Unc45b
Mis18bp1	Esco2
Cxcl13	Ccl6
Kif20b	Hells
Aspm	Fignl1
Mylk4	Mylpf
Tlr7	Emr1
Cxcl2	Cxcl13
Kif2c	Casc5
Arhgap11	Birc5
Mcm4	Shcbp1
Bub1	Plk4
Stat1	Timp1
P2ry12	Ccl5
Fyb	Ptprc
Herc6	Dhx58
Cxcl5	Rgs18

Incenp	Aim1
Wdr43	Gmps
Spc24	Plk1
Rad54l	Tpx2
Gpr65	Igsf6
Cdc25c	Cdca5
Dbf4	Ccnb2
Lrr1	Ccnb2
Lig1	Cdt1
Kif20a	Cenpi
Hmmr	Brca1
Birc5	Dlgap5
Exo1	Trip13
Mcm5	Gins1
Lig1	Smc4
Cdc6	Gsg2
Actc1	Myo18b
Rap1b	Racgap1
Tyrobp	Ms4a6d
Kif23	Plk4
Tpx2	Cenpk
Ccnb1	Hmmr
Mapre3	Kif11
Kif23	Plk1
Mpeg1	Ly86
Cenpi	Cenpk
Orc1	Mcm7
Gins1	Cdca5
Depdc1a	Top2a
Rrm2	Fbxo5
Ccdc99	Cenpn
Cxcl9	Igtp
Nckap1l	Actr2
Ryr1	Srl
Ctss	Themis2
Ezh2	Ccna2
Ccdc99	Smc4
Tacc3	Ccnb2
Ccna2	Fam64a
C3ar1	Cfb
Hells	Cdca5
Aspm	Cenpn
Lck	Cd3g
Ifi204	Ifit1
Kif20b	Clspn
Plcg2	Ly86
Kif15	Ccna2
Casp3	Casp1
Cdkn1c	Hdac9
Wdhd1	Mcm10
Ankrd1	Casq1
Ifit2	Zbp1
Cd53	Srgn
Cenpi	Incenp
Bub1b	Incenp
Myl3	Myom1
Aspm	Cdca5
Myog	Six2
Cdc7	Mcm10
Klrd1	H2-M10.4
Dlgap5	Plk1
Fcgr3	Ptprc
Cenpa	Cenph
Cenph	Tpx2
Syce2	Cdca5
Foxm1	Nusap1
Pla2g4a	Lyn
Ccnf	Fbxo31
Gins1	Asf1b
Myod1	Myc
Tmem17:	Ifit1
Cenpa	Bub1b
Niacr1	C3ar1
Snx2	Gbp1
Dnahc2	Wdr67
Igtp	Zbp1
Hspb2	Rps6ka2
Ska1	Racgap1
Nek2	Shcbp1
Stat1	Prf1
Fancd2	Clspn
Aspm	Esco2
Igtp	Rtp4
Ncl	Nop58
Atad2	Mki67
Kif2c	Smc4
Ubd	Herc6
Gins2	Cdca5
Fen1	Rrm2
Kif20b	Kif4
Shcbp1	Uhrf1
Top2a	Aspm
Mpeg1	AF251705
Cenpa	Ccnb1
Anln	Hells
Plcg2	Ptpn6
Dsn1	Cenpk
Dbf4	Esco2
Igsf6	Itgb2
Cenpk	Asf1b
Ccnb1	Mcm3
Diap3	Racgap1
B2m	Oasl1
P2ry12	Cxcl13
Fignl1	Rrm2

Cenpa	Mis18a
Igf2	Timp1
Mgam	Hk3
Cdc6	Kpna2
Racgap1	Kif11
Il24	Il7r
Bub1b	Uhrf1
Serpine1	Myc
Fancd2	Brca1
Ckmt2	Mb
Rad51	Sgol2
Mki67	Racgap1
Inpp5d	Prkcb
Slc11a1	Myc
Mad2l1	Mapre3
Ccnb2	Kif11
Incenp	Esco2
Gsk3a	Rictor
Chtf18	Plk1
Dsn1	Bub1
Gins2	Trip13
Pik3r5	Gnai3
Cd55	Cfb
Top2a	Racgap1
Cenpi	Kif11
Casp8	Ptpn6
Cxcr6	Sstr5
Gpr18	Cxcr6
Xcl1	Kiss1
Cdca7	Fen1
Ska1	Ska3
Depdc1b	Pbk
Anln	Kif11
Clspn	Esco2
Nusap1	Mis18bp1
Sash3	Ptpn6
Cxcr7	Niacr1
Cenpi	Anln
Orc6	Mcm6
Blm	Mus81
Ska1	Cdca3
Rad51	Asf1b
Tlr4	Timp1
Ccnb2	Trip13
Kcnn4	Ikzf1
Foxo6	Mapk12
Tlr7	Casp1
Samd9l	Ifi44
Ttn	Myl3
Ddx60	Dhx58
Lcp2	Plcg2
Ppapdc3	Tmem201
Dbf4	Clspn
Birc5	Parpbp
Anln	Mki67
Evi2a	Nckap1l
Mpeg1	Irf8
Cdkn3	Kif2c
Gbp2	Ifit2
Fignl1	Fen1
Blm	Rad51l3
Ccdc99	Birc5
Ddx60	Ifi44
AW1120	Ms4a4c
Ghsr	F2rl1
Ptpn2	Mbp
Ifit1	Isg15
Prc1	Cdc6
Actr3	Actr2
Ccr5	Anxa1
Trp63	Gls
Cenpa	Anln
Cacna1s	Cav3
Myom1	Ldb3
Kif20b	Plk1
Mad2l1	Casc5
Cdc25c	Spc25
Myod1	Frzb
Sct	Adm
Ncapg2	Top2a
Prc1	Hells
Ccr5	Ccl7
Mcm4	Pola1
Tlr1	Tlr2
Psmb9	Cdc6
C1qb	Ccl6
Prim1	Mcm7
Ezh2	Asf1b
Ccl5	Ccl7
Mad2l1	Kif2c
Ccdc99	Tpx2
Arhgap25	Obscn
Nckap1l	Itgb2
Kif20a	Birc5
Trip13	Spc25
Tap1	Irf7
Ccdc99	Clspn
Lig1	Tacc3
Gbp7	Usp18
Cenpi	Uhrf1
Plk2	Mcm7
Cenpa	Hist1h2ao
Ska3	Trip13
Btg2	Chaf1a
Ccr5	Xcl1

Fcer1g	Igsf6
Cenpn	Fam64a
Hdac9	Tuba1c
Birc5	Bub1
Cd48	Cd53
Kif20b	Cdca5
Kif20a	Fbxo5
Cdkn3	Lats1
Lig1	Prim1
Ccnb1	Cdkn3
Eif1a	Wdr43
Prc1	Gsg2
Inpp5d	Ptprc
Themis2	Slc15a3
Trim59	Smc4
Ccnb1	Mcm6
Mad2l1	Chaf1b
Nusap1	Ttk
Mad2l1	Cdca3
Ccnb2	Btg2
Cxcr7	Ccl6
Mpeg1	Emr1
Ska1	Incenp
Fen1	Incenp
Rrm2	Pola1
Cdc6	Mcm10
Shcbp1	Esco2
Cybb	Itgb2
Cd48	Coro1a
Bub1b	Cdca3
Ncapg2	Mcm3
Fcgr3	Fcgr2b
Arhgap11	Nek2
Ccnb1	Nek2
Rad54l	Brca1
Cdca2	Anln
Arhgap30	Vav1
Ptprcap	Ptprc
Kif20a	Mcm6
Ccr6	Cxcl13
Kif2c	Kif11
Spc24	Incenp
Pbk	Rrm2
Parp14	Psmb8
Gpr18	Cxcl13
C3ar1	Ccl5
Mcm3	Pbk
Prc1	Cdc25c
Itgam	Tlr4
Ttn	Myod1
Cenpi	Rrm2
Chtf18	Exo1
Foxm1	Anxa1
Fcgr1	Slc15a3
Fcgr3	Mrc1
Skp2	Mcm3
Sgol2	Cenpk
Ddx60	Iigp1
Depdc1b	Rad51
Cxcr6	Ccr2
Rxfp3	Cxcl5
Tyrobp	Ptpn6
Ccnb1	Parpbp
Cenpn	Mcm7
Kif4	Cenpk
Ncapg2	Hells
Ifi204	Ifih1
Mis18bp1	Ccnb2
B2m	Fcgr1
Rrm2	Spc25
Bub1	Uhrf1
Rad51	Cdca5
Fam26f	Themis2
Smc4	Mki67
Isg15	Psmb8
Lyn	Itgb2
Bub1b	Sgol2
Kif20a	Cdc6
Shcbp1	Gmnn
Txk	Stat1
Ccna2	Shcbp1
Dlgap5	Gins1
Evi2a	Emr1
Cenpk	Kif11
Nek2	Terf1
Prc1	Depdc1a
Mcm6	Pbk
Ccl5	Cxcl1
Zbp1	Rsad2
Anln	Pacsin3
Prkag3	Pfkfb1
Rnf123	Ubac1
Dlx1	Fgf6
Tmem173	Ifi204
Myog	Cdh4
Rap1b	Actr3
Fignl1	Ccna2
Npy	Cd63
Kif4	Kif15
Parp9	Gbp3
Wdhd1	Mcm4
Ptprc	Il7r
Ect2	Hmmr
Ccnb2	Pbk
Gmps	Ptprc

Cenpi	Mcm7
Parp9	Dhx58
Rasa1	Lyn
Foxm1	Arhgef39
Ms4a7	Tyrobp
Mcm5	Brca1
Cd68	Alox5ap
Fgfr4	Fgf6
Ccnb2	Rrm2
Clspn	Incenp
Ccna2	Cenpk
Parp14	Mx1
Racgap1	Asf1b
Cenpa	Mcm10
Incenp	Mcm7
Terf1	Lmnb1
Mcm3	Uhrf1
Ttn	Obscn
Cish	Lepr
Npy	Rgs18
Wdhd1	Mad2l1
Mad2l1	Cdc6
Wdhd1	Cdc7
Casp8	Myc
Clec4a3	Ly86
Ect2	Tpx2
Ttn	Cox6a2
Plk2	Fbxo5
Depdc1a	Ccna2
Atp1a4	Atp1b2
Tpx2	Uhrf1
Mki67	Ptprc
Ska1	Trip13
Ncf4	Nckap1l
Myod1	Runx3
Parp9	Isg15
Ect2	Fignl1
Kcng4	Kcnh2
Chtf18	Asf1b
Mcm5	Mcm7
Blm	Gins1
Tacc3	Top2a
Mcm6	Prim2
Skp2	Ccnb1
Birc5	Casp8
Skp2	Trip13
Actc1	Smarcd3
Sla	Hck
Ednrb	Ccl6
C3ar1	Cxcl1
Mki67	Plk4
Parpbp	Cdca5
Ncapg2	Spc25
Dctpp1	Prim1
Ddx60	Rsad2
Arhgdib	Vav1
Ccnf	Ccna2
Cenpk	Spc25
Dbf4	Bub1b
Mcm10	Pola1
Kif2c	Fen1
Mis18a	Pbk
Itgam	F3
Ska1	Asf1b
Tacc3	Esco2
Kif20b	Tacc3
Ncapg2	Birc5
Gbp7	Rsad2
Tacc3	Fignl1
Ttk	Pbk
Mad2l1	Cenph
Lcp2	Hck
Depdc1b	Kif4
Stat1	Hspb3
Troap	Uhrf1
Rad51	Mcm4
Kif20b	Rrm2
Ccr2	Xcl1
Stat1	Epsti1
Cenph	Racgap1
Apobec2	Unc45b
Cenpa	Ccna2
Nusap1	Hmmr
Dlgap5	Mki67
Parpbp	Cenpk
Ncapg2	Mki67
Casc5	Racgap1
Cxcr6	P2ry12
Kif20a	Fam64a
Map3k8	Map2k6
Cenpa	Wdhd1
Topbp1	Mcm7
Stat1	Lyn
Wipf3	Actr2
Ccnf	Ccne2
Cenpi	Mcm6
Lck	Cd5
Fcgr2b	Ptprc
Stat1	Parp12
Camp	Stat1
Ccna2	Lmnb1
Gbp2	Ifi203
Bub1	Kif11
Ttn	Trim54
Pif1	Rad51

C1qc	Mrc1
Ntf3	Ikzf1
Plk4	Hells
Kif20a	Depdc1a
Ccr5	Cxcl2
Orc1	Mcm3
Skap1	Fyb
Depdc1a	Sgol2
Ect2	Exo1
Prc1	Fam64a
Rac2	Hck
Psmb9	Isg15
Cdca2	Dlgap5
Nusap1	Esco2
Ttk	Mis18bp1
Tacc3	Bub1b
Ncf4	Coro1a
Ttk	Racgap1
Cdca3	Kif11
Pla2g4e	Pla2g7
Kif20b	Plk2
Fyb	Lyn
Ncf1	Rac2
Cenph	Clspn
Blm	Mcm10
Cenph	Aspm
Ctss	Ms4a7
Fcgr3	Lat
Epha6	Ppm1l
Topbp1	Rad51
Birc5	Nusap1
Kif20b	Rad51
Ccnf	Mastl
Myc	Apaf1
Lat	Ptpn6
Mis18a	Shcbp1
Cenpa	Uhrf1
Ghsr	Xcl1
Sstr5	Cxcl13
Xcl1	Anxa1
Ncf4	Lilrb4
Top2a	Arhgef39
Ccnb2	Myc
Tpm2	Myl3
Sash3	Cd3g
Gbp2	Iigp1
Dbf4	Mad2l1
B2m	Calca
Sgol2	Incenp
Ttk	Dlgap5
Mad2l1	Cdca5
Kif20a	Smc4
Ncapg2	Mcm6
Fcgr4	Itgb2
Kif2c	Ska1
Dsn1	Ccna2
Mad2l1	Kpna2
Il18	Il18r1
Kif15	Cdca5
Il18	Vcam1
Gins1	Esco2
Myc	Btg2
Cox6a2	Ckmt2
Lig1	Chtf18
Cenph	Uhrf1
Ppara	Rxrg
Kif2c	Cenpn
Smim15	Cetn3
Exo1	Pbk
Mki67	Hmmr
Ccr2	Ccl7
Uhrf1	Mcm7
Rab8b	Exoc3l2
Spc24	Shcbp1
Ect2	Atad2
Kif20a	Plk5
Fen1	Pbk
Wdhd1	Gins1
Cdc25c	Tpx2
Hfe2	Ckmt2
Aspm	Ccnb2
Kif4	Tpx2
Ccna2	Asf1b
Cxcl9	Stat1
Kcnc1	Kcnc4
Ifit1	Herc6
Prdm1	Gzmb
Ccr6	Ccr2
Aldh3a2	Ppara
Fancd2	Rad51
Pik3r5	Itgb2
Shcbp1	Hmmr
Ptprc	Ptpn6
Ccnb1	E2f8
Rgs18	F2rl1
Smc4	Prim1
Anln	Racgap1
Cdc6	Mcm3
Rps3a	Eif4a2
Cenpa	Hist1h4h
Aspm	Mis18bp1
Usp18	Krt14
Ncapg2	Kif23
Cenpa	Cdc25c
Gins2	Shcbp1

Mcm3	Tpx2
Clspn	Plk5
Gsg2	Bub1b
Nek2	Racgap1
Evi2a	Fyb
B2m	Gbp1
Slc15a3	Ccl3
Arhgef39	Ska3
Ccnf	Spc24
Kif15	Shcbp1
Dyrk1b	Myog
Cx3cr1	Cxcl13
Iigp1	Gbp3
Fcgr2b	Actr3
Rrm2	Mcm7
Ctss	Srgn
Ifi204	Zbp1
Cdca5	Chaf1b
Cacna1d	Rasgrp1
Ctss	Tyrobp
Ccne2	Pbk
Parp12	Parp14
Rad51	Cdt1
Tacc3	Tpx2
Exo1	Sgol2
Clspn	Exo1
Tyrobp	Ptprc
Ccr5	Ccl5
Mki67	Shcbp1
Cd48	Tyrobp
Rad51	Cdca3
Mcm3	Esco2
Gzmb	Serpinb9b
Ttk	Esco2
Foxo6	Prkab2
Birc5	Ska3
Cxcl9	Serpina3g
Gmfg	Myom2
Cenpi	Foxm1
Foxm1	Hmmr
Cdca3	Cdt1
Themis2	Slc11a1
Clec4a3	C1qc
Bub1	Pbk
Itga6	Mcm7
Ncf4	Ly86
Cdkn3	Racgap1
Atad2	Cdca5
Cybb	Hck
Kif2c	Kif23
Usp1	Brca1
Irf8	Ptpn6
Mis18bp1	Mastl
Tpx2	Spc25
Clec4a3	Cybb
Txk	Plcg2
Ccdc99	Bub1b
Parpbp	Asf1b
Ryr1	Mylpf
Cdc6	Bub1b
Parp9	Rtp4
Myod1	Fgf6
Top2a	Bub1b
Cdc25c	Rbl1
Ttk	Asf1b
Mad2l1	Fignl1
Arhgdib	Hck
Anln	Ccnb2
Kif20b	Aspm
Gbp1	Irf7
Fcgr1	Emr1
Hells	Cenpk
Fen1	Chaf1b
Ramp1	Calca
Spc24	Parpbp
Irgm2	Psmb8
Cdca3	Hmmr
Mapk12	Ptpn6
Cxcr7	Ccl9
Kif20a	Uhrf1
Ccnb1	Esco2
Cdkn1c	Rbl1
AI464131	Ppapdc3
Rgs18	Gnai3
Cdca2	Cenpi
Rgs18	Ccl9
Mcm3	Smc4
Slc11a1	Ms4a6d
Parp9	Oasl1
Spc24	Rad51
Ly96	Ly86
Cxcl9	Ccr2
Itgam	Slc11a1
Dbf4	Pbk
Vcam1	Mb
Tuba1c	Anln
Clspn	Mcm4
Orc6	Mcm7
Cdca2	Nusap1
Chrm5	Ednrb
Ms4a7	C1qb
Cdca2	Shcbp1
Prim1	Racgap1
Sgol2	Rrm2
Rad54l	Cenpn

Mad2l1	Cdt1
Ect2	Pbk
Cdc25c	Shcbp1
Ptprc	Hck
Prim2	Mcm4
Aspm	Troap
Skp2	Ccnb2
Wnt9a	Fzd9
Lst1	Tyrobp
Camk2a	Camk2b
Prc1	Arhgef39
Cxcl2	Cxcr7
Fpr2	Anxa1
Themis2	Nckap1l
Lck	Myc
Fcer1g	Emr1
Npy	Kiss1
Mapk12	Myod1
Dbf4	Ccna2
Cenpi	Mcm3
Fcgr3	Ccl6
Alox5ap	C1qb
Cdc6	Cdca3
Gbp1	Ifih1
Bub1b	Nek2
Ccne2	Orc6
Gngt2	Pik3r5
Cxcl9	Ubd
Arhgef39	Tpx2
Srgn	Gzmb
Ly86	Cybb
Ncapg2	Ttk
Cdca2	Cdc25c
Pld4	C1qc
Niacr1	Ccl6
Psmb9	Irf7
Nek2	Trip13
Ccdc99	Cenpq
Topbp1	Brca1
Cenpi	Spc25
Myc	Fasl
Bub1	Cdt1
Itgam	Serpinb9b
Lipg	Apoc2
Foxm1	Uhrf1
Fancd2	Rad51l3
Barx2	Myod1
Cenpi	Gins2
Kif15	Bub1
Casp3	Ngfrap1
Cdc25c	Mastl
Plk4	Racgap1
Lyn	Lpxn
Isg15	Ifih1
Thbs1	Cd69
Csf2ra	Ptpn6
Ccna2	Kif11
Cenpi	Plk1
Ptprc	Cd3g
Ctss	Pld4
Plk1	Bub1
Mcm5	Clspn
Prc1	Ska1
Tlr7	Dhx58
Ctss	Cd53
Slc15a3	Cybb
Ska1	Bub1b
Cdca5	Spc25
Lcp2	Ncf4
Kif20b	Mki67
Aif1	Ly86
Mcm10	Cdt1
Cx3cr1	Cxcl5
Fpr1	Ccr2
Cxcl5	Ccl9
Clspn	Spc25
Chtf18	Cdc7
Aspm	Incenp
Birc5	Tpx2
Gins2	Tpx2
Kif20b	Bub1b
Ccr6	Cxcr7
Kif20b	Ccna2
Fignl1	Uhrf1
Ccnb1	Casc5
Ncapg2	Exo1
Cenpi	Ttk
Cenph	Kif11
Depdc1a	Tacc3
Kif20a	Casc5
Cd48	Ptprc
Mki67	Rad51
Fpr2	Ghsr
Mcm5	Cdca2
Parp9	Ifi47
Aspm	Rrm2
Ifih1	Rsad2
Ctss	Evi2a
Psmb9	Cdt1
Map4k1	Rac2
Mcm3	Cdca5
Kcnc1	Kcnh2
Plk1	Tpx2
Ccdc99	Mcm3
Il20rb	Ptpn6

Mcm6	Cdt1
Cd180	Ly86
Gbp2	Vcam1
Troap	Asf1b
Cxcr7	Anxa1
Ctss	Plek
Esco2	Hmmr
Myo18b	Homer2
Ccr6	Ccl4
Tmem173	Ifit2
Fyb	Itgb2
Cenph	Cenpn
E2f8	Ccna2
Ctss	Aif1
Mpeg1	C1qb
Lck	Sell
Slfn2	Zbp1
Topbp1	Pola1
Incenp	Cdca3
Il24	Il13ra2
Gsg2	Ska3
B2m	Myc
Skp2	Fbxo5
Kif2c	Depdc1b
Mapk12	Cybb
Sstr5	Ccl9
Grip1	Myod1
Arhgap11	Uhrf1
Arhgap11	Ccnb1
Parp9	Parp12
Myl3	Casq2
Mad2l1	Smc4
Kif2c	Bub1
Frzb	Myoc
Cenpa	Cdca3
Ms4a6c	Ms4a7
Fignl1	Cdt1
Cd68	Ms4a6d
Nusap1	Ccnb2
Tpx2	Cdt1
Ect2	Kif11
Fbxo15	Abcb4
Ezh2	Mki67
Cdc6	Trip13
Spc24	Cenpk
Ahnak	Coro1a
Ska1	Shcbp1
Arhgap11	Cenph
Cdc25c	Plk4
Gins2	Ccna2
Prc1	Kif15
Ccna2	Mastl
Hk3	Tigar
Esco2	Rrm2
Sgol2	Pbk
Avpr2	Calca
B3gnt2	Ggta1
Ncl	G0s2
Isg15	Parp14
F2rl1	Ccl6
Plk1	Mcm4
C1qb	Aif1
Rxfp3	Sstr5
Atad2	Bub1
Clspn	Tpx2
Lilrb4	Itgb2
Cdc25c	Ska3
Cdca2	Mcm7
Cenpa	Cenpn
Ccne2	Ccnb2
Nek2	Esco2
Cdc6	Ttk
Abi1	Wasf2
Ezh2	Bub1
Cdc6	Fbxo5
Lrr1	Tacc3
Cdc6	Exo1
Brca1	Uhrf1
Rod1	Hnrnpa2b1
Spp1	Retnlg
Prim2	Mcm7
Tacc3	Fen1
Cdc6	Brca1
Plcg2	Lyn
Itga6	Tspan32
Aspm	Pbk
Irg1	Ccl2
Ldb3	Ckmt2
Gpr18	Ccl9
Mis18bp1	Sgol2
Dusp4	Pbk
Anxa1	Ednrb
Hist1h4h	Hist1h2ao
Mad2l1	Spc24
Spc24	Mcm3
Casp8	Ripk3
Ect2	Anln
Npy	Ccl9
Trem3	Tyrobp
Nek2	Cep192
Cdkn3	Plk2
Mpeg1	Igsf6
Gbp2	Irf7
Tyrobp	Slc15a3
Ddx10	Wdr43

Lilrb4	Cd53
Adam8	Lilrb4
Cx3cr1	Ccl4
Rad51c	Exo1
Kif20b	Dlgap5
Myc	Cdca7l
F2rl1	Kiss1
Lat	Hck
Mcm5	Bub1
Pla2g4e	Ptgs2
Tacc3	Parpbp
Mcm5	Cdca3
Ncapg2	Hmmr
Wdhd1	Exo1
Pbk	Hmmr
Casc5	Cenpk
Gm6377	AA467197
Clspn	Rrm2
Plk1	Cep192
Bub1b	Trip13
Mis18bp1	Cdca3
Dnajc2	Gmps
Lrr1	Cdc25c
Mcm5	Fignl1
Mad2l1	Kif20b
Cd53	Fcgr1
Ms4a6c	Gpr65
Ccr6	Rgs18
Mcm5	Arhgef39
Kif23	Incenp
Mpeg1	Lilrb4
Ccdc99	Ttk
C1qc	Srgn
Phf11d	Irgm2
Parpbp	Spc25
Myc	Pbk
Mcm10	Pbk
Cxcl9	Fpr2
Casc5	Kif11
Lck	B2m
Kif20a	Plk4
Ifit2	Rtp4
Birc5	Arhgef39
Mad2l1	Mcm10
Irgm2	Zbp1
Ccnf	Incenp
Inpp5d	Nckap1l
Mcm5	Smc4
Sgol2	Uhrf1
Ska1	Ccna2
Itgam	Hck
Mcm6	Hells
Odf3l2	Tuba1c
Gins2	Rad51
Rrm2	Kif11
Ms4a6c	Fcgr1
Ccdc99	Aspm
Dlgap5	Ska3
Adm	Calca
Fcer1g	Fcgr1
Gins2	Pbk
Sell	Ptprc
Clta	Lgmn
Ncoa3	Rxrg
Mcm3	Plk2
Gbp7	Herc6
Clspn	Bub1
Cxcr7	Fpr1
Thbs1	Rrm2
Prf1	Sell
Fancd2	Bard1
Dock2	Hck
Emr1	Itgb2
Lcp2	Ptpn6
Cenpn	Tpx2
Casp3	Birc5
Cdc6	Hells
Arpc5	Fcgr2b
Gmps	Gmpr
Ak1	Rrm2
Mpeg1	Themis2
Parpbp	Nek2
Ttk	Hmmr
Asf1b	Chaf1a
Ncf4	Rac2
Ccnb1	Clspn
Cenpa	Kif20b
Prf1	Ccl2
Anln	Clspn
Psmb9	Tap1
Il24	Csf2rb
Lyn	Itpr3
Itgam	Ccl4
Ccr6	Ccl7
Cdca5	Asf1b
Csf2rb2	Stat1
Troap	Cdca5
Usp18	Mx1
Mcm5	Mcm4
Themis2	Cd53
Cenpa	Cdc6
Cdc6	Pbk
Plcg2	Btk
Aspm	Clspn
Kif2c	Kif4

Rxfp3	Ccl6
Fancb	Ifih1
Cenpn	Bub1
Isg15	Oasl1
Ccnf	Mad2l1
Bub1	Cenpk
Tlr4	Prss16
Ccna2	Prim1
Cdkn3	Nusap1
Niacr1	Cxcl1
Mad2l1	Esco2
Racgap1	Myc
Ppara	F3
Mki67	Casc5
Kif20a	Top2a
Kif20a	Rrm2
Atad2	Kif11
Kcnh2	Kcne3
Depdc1a	Fam64a
Ccnb2	Sgol2
Mpeg1	Cybb
Cd3d	Sash3
Lrr1	Brca1
Rap1b	Frk
Cdc6	Ska3
B2m	Fcgr4
Kif4	Rrm2
Il18	Casp1
Shcbp1	Cenpk
Wdr43	Dnttip2
Ttk	Plk4
Klrb1	Hck
Kif15	Hmmr
Rbl1	Mcm4
Cenpi	Rad51
Mki67	Incenp
Ccnb1	Racgap1
Dppa2	Retnlg
Cxcl2	C3ar1
Msn	Actc1
Foxm1	Rad51
Mcm5	Rbl1
Cxcr7	Ccl5
Wdr43	Nop58
Arhgef39	Ccna2
Gmnn	Cdt1
Ikzf1	Fgfr4
Hist2h3c2	Hist1h4i
Ezh2	Atad2
Spc24	Ccna2
Fcgr3	Plcg2
Ccnf	Rbl1
E2f8	Bub1
Usp18	Dhx58
Esco2	Trip13
Gins1	Sgol2
Cd69	Serpinb9b
Depdc1b	Racgap1
Mad2l1	Asf1b
Asf1b	Uhrf1
Dsn1	Mad2l1
Kif4	Racgap1
Kif2c	Arhgef39
B2m	Slc11a1
Arhgap11	Trip13
Rad54l	Gins1
Top2a	Exo1
Exo1	Racgap1
Dlgap5	Kif11
Lrr1	Cenph
Arhgdib	Depdc7
Lpxn	Itgb2
Ect2	Dlgap5
Il18	Cd48
Plek	Ccl3
Wdhd1	Chtf18
Hells	Mcm7
Csf2rb2	Ms4a6d
Orc1	Psmb8
Kpna2	Kif11
Kif4	Cdca3
ENSMUS	Tuba1c
Cxcl9	Ccr6
Ttk	Atad2
Ctss	Nckap1l
Cdca2	Mad2l1
Cdca2	Gins2
Atad2	Sgol2
Mastl	Esco2
Cdc25c	Nek2
Cysltr1	Anxa1
Sla	Epha2
Xcl1	Ccl6
Tmem173	Ifih1
Fmr1	Papd4
Tpx2	Fam64a
Cdc6	Asf1b
Lcp2	Cd53
Rtp4	Oasl1
Cd53	Itgb2
Casp1	Fasl
Birc5	Top2a
Cenpa	Ttk
Fcer1g	Alox5ap
Cenpa	Kif15

Birc5	Kif11
Ccna2	Rrm2
Cxcr6	Rgs18
Casp3	Ptgs2
Irgm2	Rtp4
Casp3	Arhgdib
Ifi47	Gbp1
Kif23	Asf1b
Ect2	Cenpn
Mcm5	Ccdc99
Phf11d	Parp14
Ccnb1	Kif4
Ttk	Bub1b
Mpeg1	Slc15a3
Ccr5	Ccr2
Rnf213	Ifih1
Cdc6	Ccna2
Arhgap11	Hmmr
Trim59	Mki67
Birc5	Fam64a
Wdhd1	Clspn
Coro1a	Ptpn6
Nusap1	Rad51
Pld4	Ptpn6
Prc1	Parpbp
Fcgr3	Pld4
Fpr2	Slfn2
Ncf4	Hck
Msn	Tlr4
Anln	Dlgap5
Dbf4	Mcm3
Cenph	Bub1
Mlkl	Ppm1l
Mcm6	Prim1
Hdac9	Myod1
Cxcl9	Ccl5
Klrd1	B2m
Ifih1	Irf7
Aspm	Plk1
Cxcl13	Gnai3
C1qc	Clec4n
Arhgap11	Ncapg2
Aspm	Dlgap5
Cenpa	Hdac9
Ccl5	Rgs18
Stat1	Ptgs2
Cenpi	Bub1b
Gbp2	Rsad2
Fignl1	Prim1
Mcm5	Cenpa
Plk1	Kif11
Cdca2	Kif2c
Spc24	Ttk
Pbk	Kif11
Ttk	Rrm2
Ccnb1	Smc4
Cenpa	Birc5
Evi2a	Itgb2
E2f8	Rbl1
Depp	Serpine1
Bub1	Fbxo5
Chrm5	Rgs18
Mpeg1	Ptprc
Ccnb2	Ska3
Nek2	Kif11
Kif20a	Spc25
Kif23	Uhrf1
Csf2rb2	Csf2rb
Mis18a	Cenpk
Pla2g4a	Ptgs2
E2f8	Uhrf1
Ifi47	Slfn2
Gins1	Fen1
Cdca2	Trip13
Pfkfb1	Prkab2
Blm	Wdhd1
Cxcl2	Rgs18
Cdc25c	Uhrf1
Anxa1	Gnai3
Hdac9	Bub1b
Fyb	Lat
Kif23	Esco2
Arhgap11	Plk4
Gbp3	Ifih1
Arhgap11	Cdc25c
Gins1	Gmnn
Shcbp1	Spc25
Epcam	Ptprc
Tacc3	Asf1b
Fcer1g	Lyn
Chaf1b	Mcm7
Cenpa	Hist1h2ab
Riiad1	Spata26
Anln	Bub1
B2m	Casp3
Bub1b	Ccna2
Fpr2	Ccl9
Rad51	Fbxo5
Msn	Casp8
Lrr1	Mis18bp1
Depdc1a	Kif2c
Hells	Mcm4
Ccdc99	Incenp
Cxcr6	Ccl2
Rap1b	Rac2

Igtp	Iigp1
Arhgef39	Bub1b
Ccna2	Plk4
Pbk	Kpna2
Rad54l	Exo1
Top2a	Tpx2
Mis18a	Mcm4
Stat1	Tlr2
Dbf4	Ect2
Itgam	Aif1
Pyhin1	Epsti1
Clec4a3	Fcgr4
Igf2	Serpine1
Ncapg2	Uhrf1
Palb2	Rad51c
Kif4	Asf1b
Plk1	Cdt1
Plk1	Cdc7
Fignl1	Spc25
Inpp5d	Coro1a
Cdc7	Pola1
Alox5ap	Ms4a6d
Igtp	Dhx58
Arhgap15	Ptprc
Blm	Pola1
Rad51	Bard1
Fga	Itgb2
Prf1	Tyrobp
Plk1	Casc5
Ccnf	Mcm7
Mis18bp1	Parpbp
Wipf3	Actr3
Kif20b	Racgap1
Rad51	Trip13
Irgm2	Fam26f
Ccnb2	Plk2
Birc5	Aim1
Lck	Skap2
Fpr1	Cxcl5
Psmb9	Gbp7
Ttk	Anln
Cenpa	Ccdc99
Pld4	Rac2
E2f8	Mki67
Cd6	Cd5
Kif20a	Tacc3
Ttn	Ckmt2
Gbp2	Fcgr1
Samd9l	Ddx60
Mcm5	Fen1
Fcgr3	Fcgr1
Slamf8	Fam26f
Isg15	Ldb3
Wdr43	Tex13
Lck	Ppm1l
Kif2c	Sgol2
Skp2	Psmb8
Ccna2	Casc5
Trim34a	Trim30d
Dlgap5	Cenpn
Ccne2	Gmnn
Pld4	Coro1a
Kif20a	Asf1b
Ccnb1	Bub1
Cdca7	Mcm4
Hells	Kpna2
Pbk	Cdt1
Evi2a	Coro1a
Kif20b	Cenpk
Ccr6	Ccl3
Depdc1a	Plk1
Naip2	Apaf1
Lcp2	Lck
Cdkn3	Plk5
Plcg2	Lat
Cxcl9	Gnai3
Top2a	Kif11
Cenpl	Ska1
Top2a	Topbp1
Gpr65	Ccl9
Vgll2	Dlx4
Cenpl	Kif2c
Mad2l1	Mcm7
C3ar1	C1qb
Plk5	Bub1
Dusp4	Grin1
Plk5	Bub1b
Ect2	Hells
Cdc25c	Esco2
Ncf4	Myo1f
Fcgr4	Fam26f
Lcp2	Itgb2
Arhgap11	Anln
Retnlg	Ppara
Casc5	Uhrf1
Slc11a1	Itgb2
Mad2l1	Ccna2
Cenpq	Cenpk
Fcgr3	Tyrobp
Ska1	Cenpk
Cdca2	Depdc1a
Lilrb4	Ms4a6d
Top2a	Eif4a2
Lrr1	Plk1
Fpr2	Cxcl5

Kif15	Plk4
Map4k1	Cd3g
Igtp	Psmb8
Iigp1	Psmb8
Ect2	Kif20b
Tacc3	Mcm4
Kif20a	Nek2
Epha8	Ppm1l
Ska1	Rrm2
Ncl	Myc
Prc1	Top2a
Colq	Ache
Prc1	Mis18bp1
Camk2b	Plcg2
Mcm5	Skp2
Ccr5	Npy
Arhgap11	Plk1
Txk	Ptpn6
Rac2	Ptprc
Hmmr	Uhrf1
Fcgr4	Tyrobp
Klrd1	Gzma
Isg15	Tlr7
Kif20b	Prim1
Racgap1	Fam64a
Cenpn	Casc5
Dbf4	Mcm4
Ttn	Mypn
Tpx2	Shcbp1
Ccnb1	Kif11
Lcp2	Map4k1
Mis18bp1	Kif11
Prim1	Uhrf1
Tmem38a	Srl
Ccl4	Ccl3
Ect2	Kif23
Ddx60	Usp18
Pbk	Fbxo5
Stat1	Ccl2
Lig1	Fignl1
Cdc6	Birc5
Foxm1	Troap
Birc5	Mis18bp1
Cenpa	Cdkn3
Ppara	Mlxipl
Wdhd1	Gins2
Orc1	Lig1
Top2a	Smarcd3
Prc1	Cenpi
Cdca2	Cenph
Tyrobp	Cybb
Gins2	Mcm7
Cdkn3	Cenpn
Skp2	Fbxo31
Kif4	Anln
Klrd1	Tyrobp
Lck	Klrb1
Ska3	Asf1b
Ms4a6c	Ccl6
Pld4	Tyrobp
Stat1	Parp14
Klrd1	Prf1
Cenpi	Ccna2
Prf1	Cd69
Ezh2	Ccnb1
AF25170	Trem3
Prc1	Mapre3
Xcl1	Ednrb
Fignl1	Mki67
Dbf4	Bub1
Nckap1l	Tyrobp
Arap2	Obscn
Atad2	Rad51
Tpx2	Hmmr
Pla2g4a	Ubd
Cd68	Ncf4
Cysltr1	Xcl1
Ccdc99	Esco2
Kif23	Racgap1
Usp1	Mcm4
Sqle	Lbr
Cd3d	Vav1
Cxcr7	Ccr2
Kif4	Exo1
Spc24	Pbk
Alox5ap	Emr1
Ms4a6c	Mpeg1
Smc4	Atad2
Evi2a	Cd53
Ncf4	Cd52
Arhgap25	Vav1
Cdc6	Dlgap5
Atad2	Fbxo5
Parp9	Zbp1
Ms4a6c	Aif1
Prim1	Cenpk
Abi1	Ldb3
Eps15	Racgap1
Atad2	Prim1
Rac2	Clec10a
Tacc3	Spc25
Ccr2	Ccl9
Lck	Rac2
Cd5	Hck
Exo1	Uhrf1

Dtna	Sgca
Lyn	Gnai3
Ms4a4c	Fam26f
Myo18b	Obscn
Ptpn6	Itgb2
Cdca3	Uhrf1
Isg15	Nae1
Kif2c	Spc25
Cdc7	Ccna2
Inpp5d	Fcer1g
Txk	Cd3g
Dbf4	Prim1
Lrr1	Exo1
Mad2l1	Atad2
Cdc6	Chaf1a
Cenpa	Troap
Kif5a	Myo18b
Clspn	Racgap1
Arf6	Racgap1
Skap1	Lat
Ezh2	Mcm4
Depdc1b	Plk1
B2m	Tyrobp
Tacc3	Kpna2
Gins2	Hmmr
Gpr65	Xcl1
Gpr18	P2ry12
Plk4	Cdca5
Casp3	Gzmb
Gins1	Pola1
Fcgr4	Aif1
Ms4a6c	Alox5ap
Cdc7	Plk4
Hpgds	Tbxas1
Inpp5d	Rac2
Nckap1l	Ly86
Nusap1	Racgap1
Cx3cr1	Cxcl1
Kif4	Chtf18
Cdca2	Bub1b
Ifi47	Ifih1
Chtf18	Bub1b
Kif20a	Trip13
Foxm1	Kif4
Top2a	Spc25
Plk1	Nek2
Ppm1l	Epha2
Prim1	Spc25
Ncf4	Cybb
Mcm3	Mcm7
Itgam	Itgb2
Fcgr2b	Itgb2
Arhgef39	Racgap1
Skp2	Plk1
Lcp2	Trem2
Cenpi	Plk4
Lrrc30	Megf10
Zbp1	Dhx58
Blm	Smc4
Plcg2	Fcer1g
Ect2	Arap2
Skp2	Cdt1
Mis18a	Casc5
Herc6	Ifi44
Cd68	Fcgr1
Nsmce2	Rad51
Sgol2	Kif11
Cd53	Coro1a
Cdca3	Esco2
Rsad2	Dhx58
Kif23	Sgol2
Sstr5	Gnai3
Pld4	Aif1
Kif15	Pbk
Ncapg2	Sgol2
Rad51	Rrm2
Rnf213	Zbp1
Fbxo5	Kif11
Cdc25c	Clspn
Il24	Lepr
Gins2	Mcm4
Parp12	Usp18
Gbp2	Gbp7
Itga6	Itgb2
Cep170	Plk1
Lcp2	Btk
Ccna2	Tpx2
Dsn1	Ccnb1
Lig1	Mcm7
Nckap1l	Aif1
Myod1	Ptgs2
Lmod3	Itgb1bp2
Ccnf	Rrm2
Rad54l	Rad51
Lig1	Plk1
Ifit2	Gbp7
Psmb9	Irgm2
Cenpq	Ska1
Mad2l1	Kif11
Fcgr3	Actr2
Chrm5	Anxa1
Lck	Epha6
Casp3	Mki67
Nusap1	Rrm2
Gbp2	Snx2

Evi2a	Lpxn
Tacc3	Ska3
Cdc25c	Ska1
Top2a	Mis18bp1
Ppip5k2	Ip6k3
Ccna2	Mcm10
Ncf4	Racgap1
Plk1	Ska3
Lig1	Spc25
Rad51	Brca1
Foxm1	Bub1
Cdca2	Cdc6
Parpbp	Rrm2
Thbs1	Timp1
Sdcbp	Cd63
Cdca5	Cenpk
Lig1	Prim2
Eif2s3y	Gmps
Pi4k2b	Pik3r5
Btk	Lat
Irf8	Irf7
Ccr2	Npy
Ect2	Ska3
Inpp5d	Lyn
Rxfp3	Cxcr6
Blm	Mcm3
Tacc3	Chtf18
Exo1	Mcm10
Rac2	Vav1
Clec4a3	Igsf6
Cdca2	Uhrf1
Fcgr4	C1qb
Brca1	Kif11
Mad2l1	Cenpq
Dclre1c	Exo1
Pkp3	Anln
Bub1	Chaf1a
Kif4	Cdca5
Ccr2	Gnai3
Mcm5	Ccnb1
Parpbp	Esco2
Arhgap11	Rac2
Fxyd5	Alox5ap
Itga7	Spp1
Fpr2	Npy
Bub1b	Kpna2
Samd9l	Ifit1
Orc6	Prim1
Plcg2	Pik3r5
Cystm1	Mthfd1l
Ttk	Ccna2
Pld4	C1qb
Plk4	Pola1
Spc24	Spc25
Ifit2	Iigp1
Kif20b	Casc5
Lilrb4	Ccl3
Mad2l1	Hmmr
Inpp5d	Vav1
Cdca2	Cdca5
Cdkn1c	Ccna2
Cxcl2	Gpr18
Ska1	Bub1
Igtp	Rsad2
Stat1	Psmb8
Dpf3	Smarcd3
Mad2l1	Orc6
Chaf1b	Uhrf1
Depdc1a	Esco2
Spc24	Esco2
Clspn	Hmmr
Ska1	Gins2
Cdc6	Rad51
Cxcl9	C3ar1
Camp	S100a9
Ect2	Rad51
Map4k1	Cd3d
Kif20a	Hmmr
Ednrb	Kiss1
Kif23	Kif11
Tex13	Tex11
Kif20b	Fam64a
Exoc3l4	Rab8b
Cenph	Plk4
Hspb3	Anxa1
Cenpi	Ska1
C3ar1	Emr1
Depdc1b	Bub1
Ska1	Cdca5
Ccr5	Gnai3
Bub1	Casc5
Mis18a	Cenph
Kif20b	Sgol2
Ccnb1	Gins1
Cdc25c	Ccna2
H2-M10.4	H2-M3
Lig1	Ccnb2
Ttk	Bub1
Smc4	Dek
Ccna2	Incenp
Mad2l1	Mis18bp1
Ddx60	Parp14
Ncf4	Slc11a1
B2m	Irf7
Itga6	Lamc2

Cysltr1	Ednrb
Mpp3	Hck
Ect2	Casc5
Cdc6	Psmb8
Rbl1	Mcm6
Ccl5	Anxa1
Map4k1	Lat
Rgs18	Ccl6
Mapk12	Gngt2
Gpr65	Fpr2
Prf1	Fasl
Sgol2	Racgap1
Camk2b	Scn4b
Cenpa	Hist1h2ag
Igtp	Parp14
Tlr2	Ms4a6d
Cd68	Tyrobp
Kif20a	Ccnf
Ifi204	Casp1
Acer3	Degs2
Lig1	Tpx2
Neurl1a	Camk2b
Prc1	Birc5
Tpm2	Actc1
Smc4	Exo1
Itgam	Emr1
Cdt1	Mcm7
Stat1	Arg1
Depdc1a	Tpx2
Ttk	Aspm
Cdc25c	Parpbp
Chrm5	Xcl1
Depdc1b	Aspm
Hrc	Srl
Itgam	B2m
Mcm6	Pola1
Birc5	Casc5
Rad54l	Cdc6
Mcm5	Aspm
Topbp1	Prim1
Wasf2	Actr2
Ctss	Slc11a1
Birc5	Esco2
Plek	Rac2
Foxm1	Cdca3
Casp3	Apaf1
Cdc25c	Gins2
Cenpl	Cenph
Ifi47	Usp18
Sstr5	Cxcl1
Ifit2	Irgm2
Anln	Parpbp
Racgap1	Ska3
Mylk4	Myl6b
Depdc1a	Racgap1
Scn1b	Camk2b
Tlr4	Ptprc
Ctla2b	Ctla2a
Cd48	Itgb2
Hist2h3c2	Hist1h4h
Spc24	Lig1
Cdc6	Fen1
Gbp2	Mx1
Top2a	Clspn
Depdc1a	Ccdc99
Ddx10	Gmps
Kif2c	Clspn
Arhgap15	Arhgdib
Aspm	Kif11
Psmb9	Plk1
Tpx2	Mcm4
Mcm4	Kpna2
Igsf6	Ms4a6d
Kif4	Kif11
Spp1	Irf7
Kif20a	Ncapg2
Ccdc99	Asf1b
Plcg2	Fcgr2b
Wdhd1	Rrm2
Samd9l	Parp14
Kif20a	Bub1b
Atad2	Hells
Anln	Shcbp1
Esco2	Pbk
Lig1	Ncapg2
Kif23	Rrm2
Prc1	Ccdc99
Cenpq	Cenpn
Aspm	Fam64a
Fcgr4	Fpr2
Top2a	Hmmr
Mcm3	Cdc7
Tlr4	Tlr2
Kif2c	Cdca5
Mcm3	Sgol2
Ddx60	Mx1
Mcm10	Cdca5
Fcgr4	Hk3
Mcm5	Incenp
Spc24	Cdc25c
Clec4a3	Themis2
Csf2rb2	Ptprc
P2ry12	Ccl9
Ppara	Abcb4
Nckap1l	Ms4a6d

Kif23	Nek2
Bub1b	Mcm4
Orc6	Rrm2
Rinl	Epha8
Themis2	Ly86
Kif2c	Kif15
Mcm3	Kpna2
Prc1	Anln
Fpr1	Sstr5
Ntf3	Fmr1
Hspb3	Gzma
Spc25	Uhrf1
Chtf18	Prim1
Prc1	Spc24
Cd68	Emr1
Stat1	Myc
Dsn1	Cenph
Mak	Ccna2
Palb2	Brca1
Kif2c	Mcm3
Trp63	Dek
Chaf1b	Rrm2
Blm	Rad51
Exo1	Mastl
Arhgap11	Sgol2
Ncapg2	Smc4
Depdc1b	Ccna2
Bub1b	Fbxo5
Ms4a7	Emr1
Tyrobp	Aif1
Spc24	Cdca5
Cish	Hck
Mad2l1	Uhrf1
Exo1	Shcbp1
Wdr43	Ada
Cenpl	Birc5
Clec4d	Irg1
Oasl1	Gmps
Arhgef39	Incenp
Mcm3	Exo1
Ska1	Kif23
Ctss	Emr1
Exo1	Rad51
Rad51	Hells
Kif4	Nek2
Pla2g4a	Oasl1
Gins1	Pbk
Pla2g4e	Prkcb
Irgm2	Parp14
Exo1	Plk1
Wdhd1	Asf1b
Cenpn	Esco2
Casp1	Myc
Cdca2	Ccdc99
Aspm	Mastl
Lck	Itgb2
Ghsr	Ednrb
B2m	Cd3d
Samd9l	Irgm2
Lck	Gmps
Depdc1b	Obscn
Ncapg2	Gins2
Ccdc99	Cenpi
Cdc6	Lin54
Cdc25c	Hmmr
Aspm	Shcbp1
Dlgap5	Sgol2
Arhgap11	Ccna2
Fcgr3	Mpeg1
Plcg2	Fgfr4
Birc5	Mcm3
Arhgap11	Tpx2
Rnf213	Parp14
Top2a	Cenpn
Niacr1	Npy
Plk4	Cep192
Anln	Tpx2
Kif2c	Troap
Clec4a3	Clec4n
Ttk	Mcm3
Ncf4	Emr1
Lrr1	Casc5
Ccnf	Bub1b
Arpc1b	Nckap1l
Top2a	Dlgap5
Wdhd1	Prim2
Cd48	Cd244
Pycard	Casp1
Lyn	Ppm1l
Rac2	Arhgdib
Ccnb1	Fen1
Ccnb1	Atad2
Ska1	Spc25
Rnf213	Rsad2
Thbs1	Itga6
Mpeg1	Alox5ap
Tacc3	Ccnb1
Nusap1	Depdc1b
Ncapg2	Fbxo5
Mcm5	Topbp1
Depdc1a	Spc24
Tacc3	Fam64a
Cdc6	Topbp1
Oasl1	Irf7
Ect2	Cdc6

Kcnh2	Kcnc4
Camp	Fpr1
Eps15	Mx1
Itga7	Itgb2
Ect2	Mastl
Gmnn	Mcm7
Vcam1	Ccl7
Anln	Sgol2
Prc1	Mki67
Birc5	Tacc3
Hells	Uhrf1
Tnfaip8	Casp8
Rac2	Depdc7
Slc16a7	Ppara
Plk1	Chaf1b
Mcm4	Gmnn
Clec4a3	C3ar1
Oscar	Fcgr2b
Gsk3a	Tlr4
Casq2	Ckmt2
Exo1	Cdca5
Ifit1	Ifi44
Prc1	Ttk
Myo1f	Aif1
Bub1b	Fen1
Parpbp	Plk4
Clspn	Plk2
Prim1	Kif11
Mcm3	Gmnn
Cenpa	Aspm
Avpr2	Adm
Mcm5	Tpx2
Fcgr4	Themis2
Nusap1	Trip13
Mis18bp1	Fignl1
Lrr1	Ska1
Wasf2	Arpc5
Fignl1	Trip13
Atad2	Plk4
Ticam2	Tlr2
Plk1	Pbk
Arhgap11	Nusap1
Parp14	Oasl1
Hnrnpa2b	Cstf3
Syce2	Lmnb1
P2ry12	Ccr2
Plk1	Mcm7
Birc5	Depdc1b
Ncapg2	Plk1
Lmod3	Fsd2
C1qb	Itgb2
Hist2h3c2	Hist1h2ab
Kif20b	Troap
Gpr65	F2rl1
Irgm2	Gbp7
Prc1	Kif11
Lrr1	Cenpn
Parp9	Rsad2
Ect2	Mcm7
Ect2	Cdc25c
Prc1	Fignl1
Myod1	Fgfr4
Myod1	Stat1
Tacc3	Rrm2
Wipf3	Arpc5
Fen1	Uhrf1
Cdc25c	Ccnb2
Ccnb1	Uhrf1
Prim1	Fbxo5
Exo1	Tpx2
Isg15	Irgm2
Lrr1	Orc1
Kif20b	Cdc7
Hist1h4i	Hist1h2ag
Cdc25c	Brca1
Dlx2	Fgf6
Fmr1	Igf2bp3
Kif20a	Racgap1
Plk1	Fam64a
Ccne2	Ccna2
Fcgr4	Pld4
Mcm5	Rad51
Fignl1	Rad51
Mcm3	Clspn
Ezh2	Kif11
Fen1	Esco2
Arhgap11	Kif11
Cxcl9	Slamf8
Lig1	Clspn
Fpr2	Niacr1
Casc5	Incenp
Tlr4	Ly86
AF25170	Tyrobp
Cenpi	Nusap1
Ccr2	Cxcl13
Npy	Anxa1
Rad51	Ska3
Parp9	Gbp7
Orc1	Rrm2
Spc24	Hmmr
Ifit2	Irf7
Kif15	Mki67
Cenph	Esco2
Btk	Pik3r5
Orc1	Ttk

Depdc1a	Exo1
Pgm2	Hk3
Foxm1	Mki67
Incenp	Rrm2
Cenpa	Mcm3
Dlgap5	Bub1b
Fcer1g	Lat
Xcl1	Cxcl13
Top2a	Kif15
Mcm5	Esco2
Stat1	Cd69
Pif1	Rad51l3
Hdac9	Ptgs2
Smc4	Rrm2
Cxcr6	C3ar1
Ttn	Hfe2
Mcm3	Ccna2
Birc5	Mcm7
Aspm	Atad2
Ect2	Kif4
Mcm5	Mki67
Ect2	Plk2
Cfi	Cfb
Lig1	Ccna2
Tpx2	Ska3
Foxm1	Plk1
Cenpa	Top2a
Gmfg	Actr2
Prc1	Lig1
Sash3	Ptprc
Ccr5	Rxfp3
Aspm	Hmmr
Mcm5	Nusap1
Cd68	Pld4
Gmps	Cct6b
Cd53	Emr1
Ttk	Hells
Ezh2	Rbl1
Plk4	Ska3
Rxfp3	Niacr1
Hells	Shcbp1
Hells	Chaf1a
Clspn	Fam64a
Itgam	Prf1
Btk	Trem2
Lrr1	Bub1
Cdc6	Cdc7
Smc4	Bub1b
Parp12	Gbp3
Ncf4	Themis2
Coro1a	Ltb
Ncoa3	Ppara
Kif4	Parpbp
Ikzf1	Ptprc
Dbf4	Cdt1
Prim1	Fen1
Arhgap11	Fbxo5
Ptgs2	Tbxas1
Glipr1	Ppara
Depdc1a	Mad2l1
Prc1	Troap
Arhgap11	Asf1b
Dbf4	Cdc6
Cenpa	Cdt1
Gpr18	Npy
Alox5ap	Tyrobp
Map2k6	Ccl2
Kif4	Bub1b
Smc4	Usp1
Skp2	Myc
Gsg2	Casc5
Ncapg2	Gins1
Cenpi	Hmmr
Mcm5	Spc24
Cd72	Ptpn6
Cd53	Hck
Ccna2	Mcm7
Irf7	Ldb3
Hdac9	Stat1
Orc1	Asf1b
Zbp1	Mx1
Prkag3	Prkab2
Prim1	Cdt1
Kif20b	Smc4
Dsn1	Ska1
Plcg2	Vav1
Top2a	Gins1
Cxcl9	Npy
Tlr1	Ifih1
Epha6	Hck
Kif11	Asf1b
Orc6	Prim2
Rtp4	Dhx58
Cenpi	Arhgef39
Dlgap5	Nek2
Fcgr2b	Emr1
Hrc	Myom1
Rac2	Aif1
Exo1	Fen1
Wdhd1	Kif11
Vgll2	Dlx2
Cdkn3	Mki67
Cox6a2	Casq2
Shcbp1	Fbxo5
Ccr5	Fpr1

Chtf18	Esco2
Ska3	Hmmr
Tacc3	Cenpn
Tlr4	Map3k8
Icos	Pik3r5
Irf8	Tyrobp
Skp2	Ccna2
Kif20b	Nek2
Depdc1a	Ska1
Ccnb2	Fam64a
Birc5	Nek2
Ccnb1	Ska3
Oscar	Fcgr1
Bub1b	Gins1
Capzb	Actc1
Trip13	Asf1b
Exo1	Rrm2
Bub1b	Gins2
Mcm10	Mcm4
Irf7	Epsti1
Btk	Ppm1l
Foxm1	Ccnb2
Glipr1	Cd53
Atad2	Casc5
Prc1	Suz12
Mpeg1	Itgb2
Ccnb1	Troap
Anln	Plk4
Ccna2	Uhrf1
Top2a	Ska3
Nusap1	Kif23
Skp2	Exo1
Cxcl1	Cxcl5
Mcm5	Pola1
Fmr1	Mbp
Foxm1	Incenp
Ccnb2	Uhrf1
Cdc25c	Plk1
Cdc6	Uhrf1
Themis2	Emr1
Lyn	Ptprc
Kif23	Casc5
Mcm4	Esco2
Rad51c	Rad51l3
Ms4a7	C3ar1
Depp	Ppara
S100a9	Tlr4
Lck	Ptpn22
Prc1	Bub1
Lig1	Kif20b
Kif4	Spc25
Pla2g4a	Pla2g7
Top2a	Ccna2
Cdca2	Mki67
Mcm3	Rad51
Rasa1	Hck
Cd68	Ccl4
Cep170	Plk4
Rac2	Map2k6
Kif15	Racgap1
Cdc25c	Mis18bp1
Cd53	Ms4a6d
Ska1	Ccnb2
Phf11d	Oasl1
Ms4a6c	Igsf6
Bub1b	Prim1
Asns	Aldh9a1
Adam10	Cdh4
Tlr7	Ly86
Cdkn1c	Ezh2
Ccnb1	Kpna2
Fpr2	Cxcl1
Cd3d	Lat
Blm	Mcm5
Inpp5d	Ptpn6
Csf2rb2	Lyn
Mki67	Uhrf1
Cxcl2	Cxcr6
Cxcl9	Rgs18
Kif20b	Kif15
Suz12	Brca1
Clec4a3	Cd53
Cdca5	Racgap1
Mcm5	Ncapg2
Mcm5	Uhrf1
Kif15	Kif11
Ect2	Aspm
Tlr7	Ptprc
Ptpn2	Cdh4
Ly86	Itgb2
Plk4	Esco2
Kif20b	Mastl
Cdca2	Spc24
Cdca7	Myc
Top2a	Nusap1
F2rl1	Ccl9
Clspn	Chaf1b
Cdc7	Prim1
Igf2bp3	Myc
Apol11b	Apol10a
Cdkn3	Kif11
Plcg2	Hck
Rad54l	Asf1b
Isg15	Stat1
Mcm5	Ccna2

Cdc25c	Plk2
Cxcl9	Cxcr6
Kif20a	Nusap1
Irgm2	Parp12
Kif20b	Fbxo5
Gins1	Mcm4
Ccnb1	Nusap1
Tlr2	Nfkbie
Cxcl1	Ccl4
Irf7	Mx1
Prc1	Racgap1
Cenpa	Pbk
Samhd1	Irgm2
Birc5	Kif4
Anln	Incenp
Cd53	Aif1
Mrc1	Emr1
B2m	Ptpn6
Igsf6	Fcgr1
Rgs18	Ednrb
Wdhd1	Hells
Ccnb2	Fen1
Fcgr1	Cd3g
Gpr65	Nckap1l
Kif20a	Ccna2
Cacna1d	Gngt2
Prc1	Cenph
Prc1	Nusap1
Cdca2	Mis18bp1
Parpbp	Gins2
Tmem20[illegible]	Tmem38a
Stat1	Gbp3
Ifit2	Rsad2
Rictor	Rps6ka2
Lrr1	Hells
Ms4a6d	Ly86
B2m	H2-M3
Arhgef39	Shcbp1
Foxm1	Kif2c
Cxcr6	Ccl6
Ncapg2	Cenpi
Exo1	Cdc7
Gbp2	Ifi44
Pcx	Ldhb
Usp18	Oasl1
Aspm	Kif4
Arhgap30	Sash3
Ccnb2	Plk4
Cenph	Asf1b
Pla2g4a	Prkcb
Mcm5	Exo1
Smc4	Trip13
Rac2	Lpxn
Cdca5	Shcbp1
Nek2	Sgol2
Dlgap5	Trip13
Bub1	Trip13
Ifi47	Ifi44
Ddx60	Gbp7
Cd53	Fcgr2b
Mapk12	Stat1
Orc1	Mcm4
Dsn1	Bub1b
Ptprc	Slc11a1
Arhgap11	Cdca5
Fcgr4	Emr1
Ttk	Brca1
Mapk12	Lpxn
Tpx2	Incenp
Klrd1	Ptpn6
Kpna2	Mcm7
Camk2b	Stat1
Cd68	Lilrb4
Ncapg2	Ccna2
Ttk	Parpbp
Adam10	Grin1
Kif4	Pbk
Ccr5	Cxcl5
Ccnf	Ccnb2
Zbp1	Irf7
Ticam2	Tlr4
Parp14	Herc6
Rnf213	Stat1
Fam26f	Ifi47
Clec4a3	Slc11a1
Mus81	Pola1
Lig1	Mcm4
Clspn	Mcm7
Ms4a6d	Itgb2
Iigp1	Ifih1
Igf2	Klk1b16
Myc	Epha2
Mad2l1	Ncapg2
Mus81	Rad51
Wdhd1	Orc6
Top2a	Mcm4
Ptgs2	F2rl1
Bub1b	Mcm10
Ddx60	Trim30d
Scimp	Cd53
Spp1	Thbs1
Prc1	Cdca3
Cxcr7	Rgs18
Obscn	Racgap1
Kif4	Fignl1

Zbp1	Ripk3
Mad2l1	Rrm2
Tpx2	Rad51
Kif20b	Cdca3
Ect2	Depdc1b
Map4k1	Vav1
Nckap1l	Actr3
Clec4a3	Emr1
Ugcg	Abcb4
Mad2l1	Incenp
Myod1	Ascl2
Dlgap5	Ccnb2
Birc5	Plk2
Cdc6	Mki67
Mcm5	Arhgap11a
Gins2	Gins1
Lipg	Acsl5
Shcbp1	Rrm2
Troap	Ska3
Tlr2	Cybb
Cdc25c	Kif11
Cdkn1c	Trp63
Wdhd1	Prim1
Cdc25c	Cdca3
Cenpl	Sgol2
Kbtbd13	Klhl6
Ifit2	Dhx58
Cdc25c	Sgol2
Lats1	Wwc1
Tacc3	Ccna2
Tlr4	Cd180
Rad51	Plk4
Cxcl2	Irg1
Ccr5	P2ry12
Rxfp3	Cxcl13
Gimap4	Gimap8
Trim59	Ccna2
Rad54l	Birc5
Nusap1	Uhrf1
Cdc6	Cenpk
Atad2	Ccna2
Birc5	Prim1
Skap2	Ptpn22
Ccr6	Cxcl2
Ect2	Cenpi
Hrc	Cox6a2
Nek2	Uhrf1
Rnf213	Ifi44
Pdcd1	Ptpn6
Kif20a	Cdc25c
Tlr4	Ly96
Cd200r1	Tyrobp
Ccnb1	Chtf18
Ccnf	Foxm1
Bub1b	Ccnb2
Tpx2	Casc5
Mcm5	Lrr1
Topbp1	Mcm6
Gins2	Bub1
Slc2a3	Slc5a1
Frk	Ptpn6
Prc1	Ccna2
Topbp1	Mcm10
Ddx60	Parp12
Kif2c	Pbk
Cxcl9	Sstr5
Troap	Cdca3
Kif20a	Ska1
Cdca7	Mcm3
Orc1	Cdc7
Depdc1a	Kif15
Kif23	Shcbp1
Pbk	Ska3
Kif20b	Mcm4
Plek	Igsf6
Tacc3	Ttk
Ncapg2	Incenp
Ddx60	Irgm2
Gins2	Rrm2
Prim1	Asf1b
Pyhin1	Zbp1
Lat	Vav1
Topbp1	Usp1
Gsk3a	Fzd9
B2m	Cd3g
Birc5	Plk4
Depdc1a	Kif4
Fcgr3	Itgam
Mcm5	Cdc25c
Fyb	Frk
Birc5	Gins2
Top2a	Brca1
Kif2c	Cdc25c
Exo1	Parpbp
Ms4a6c	Cd53
Fcgr3	Wasf2
Tpx2	Mcm7
Top2a	Rad51c
Actc1	Myl3
Mad2l1	Nusap1
Prc1	Cenpn
Epsti1	Rsad2
Rgs18	Anxa1
Mpeg1	Fcer1g
Ptpn2	Frk

Cenpi	Mis18bp1
Smc4	Esco2
Rad54l	Rad51c
Rac2	Ptpn6
Plk4	Rrm2
Nfkbiz	Cxcl1
Ifit1	Igtp
Aspm	Cenpk
Birc5	Ccnb2
Depdc7	Vav1
Chrm5	F2rl1
Foxm1	Cenpn
Troap	Plk1
Hells	Esco2
Rac2	Ly86
Cenpk	Fbxo5
Clspn	Cenpn
Arf6	Snx2
Foxm1	Cdkn3
Cd68	Mrc1
Kif4	Incenp
Cdkn1c	Runx3
Mcm3	Shcbp1
Ccnb1	Plk5
Klrd1	Sell
Rac2	Itgb2
Racgap1	Mcm7
Lck	Trem2
Prpf40a	Usp1
Prc1	Smc4
Kdm2a	Kdm3b
Mpeg1	Aif1
Ticam2	Tlr7
Cdca5	Incenp
Cdkn1c	Hspb3
Clspn	Cdca5
Depdc1a	Cenpi
Igf2	Srgn
Ch25h	Lbr
Arhgap11	Spc25
Casc5	Plk4
Msn	Ccl7
Cxcl2	Ccl2
Ska1	Plk1
Kif4	Fbxo5
Ttk	Cdc25c
Samd9l	Stat1
Cdc25c	Myc
C1qb	Ly86
Ccna2	Cdt1
Prdm1	Unc5a
Mcm5	Birc5
Cd53	Nckap1l
Cxcr6	Anxa1
Dsn1	Cenpn
Cdc6	Myc
Ccnb2	Shcbp1
Prkag3	Stradb
Ccr6	Ccl9
Plek	Itgb2
Il24	Csf3r
Gbp2	Gbp3
Ifi47	Herc6
Irf8	Oasl1
Dlgap1	Grin1
Gpr65	Ednrb
Ptprc	Serpinb9b
Mki67	Cdca3
Skp2	Mki67
Plk1	Fbxo5
Fignl1	Sgol2
Xcl1	F2rl1
Fpr2	Fpr1
Ptprc	Aif1
Fbxo31	Fbxo15
Cxcl5	Ccl6
Cand2	Myog
Msn	Fasl
Tap1	Stat1
Kif20b	Kif11
Ect2	Kif2c

Table S4 Hub interaction network

Node1	Node2
Dtna	Aqp4
Neurl1a	Aqp4
Aqp4	Avpr2
Aqp4	Top2a
Aqp4	Avpr2
Inpp5d	Avpr2
Spp1	Avpr2
Avpr2	Calca
Ccnb1	Btg2
Ccnb2	Btg2
Chaf1b	Btg2
Myc	Btg2
Nfkbiz	Btg2
Skp2	Btg2
Btg2	Chaf1a
Avpr2	Calca
B2m	Calca
Fga	Calca
Myl3	Calca
Samd9l	Calca
Clta	Acap1
Clta	Arap2
Clta	Clhc1
Clta	Eps15
Clta	Kif4
Clta	Lgmn
Clta	Pcsk6
Clta	Rps6ka2
Cox6a2	Asb2
Cox6a2	Casq2
Cox6a2	Ckmt2
Actc1	Cox6a2
Apobec2	Cox6a2
Cacna1s	Cox6a2
Cav3	Cox6a2
Hfe2	Cox6a2
Hrc	Cox6a2
Hspb3	Cox6a2
Lmod3	Cox6a2
Myl3	Cox6a2
Myo18b	Cox6a2
Myom1	Cox6a2
Myom2	Cox6a2
Mypn	Cox6a2
Obscn	Cox6a2
Sgca	Cox6a2

Synpo2l	Cox6a2
Ttn	Cox6a2
Cox6a2	Ldb3
Cox6a2	Mb
Cox6a2	Mylpf
Cox6a2	Popdc2
Cox6a2	Srl
Cox6a2	Trim54
Dclre1c	Exo1
Dclre1c	Hist1h2ab
Dclre1c	Lig1
Dclre1c	Rad51
Dclre1c	Rad51l3
Mpp3	Epha8
Mpp5	Epha8
Rasa1	Epha8
Epha8	Ppm1l
Eps15	Arf6
Eps15	Birc5
Eps15	Cd63
Eps15	Clhc1
Clta	Eps15
Eps15	Fgfr4
Eps15	Mx1
Eps15	Racgap1
Eps15	Ubd
Fga	B2m
Fga	Calca
Fga	F13a1
Fga	Itga6
Fga	Itgam
Fga	Itgb2
Fga	Neurl1a
Frk	Ccl2
Frk	Cd3g
Frk	Epha2
Frk	Fasl
Abi1	Frk
Arf6	Frk
Arhgdib	Frk
B2m	Frk
Camk2a	Frk
Camk2b	Frk
Ccl7	Frk
Ccna2	Frk
Ccne2	Frk
Cd3d	Frk
Cd63	Frk

Cfb	Frk
Clhc1	Frk
Epha6	Frk
Fcer1g	Frk
Fyb	Frk
Grin1	Frk
Hspb3	Frk
Lat	Frk
Lck	Frk
Lpxn	Frk
Lyn	Frk
Map2k6	Frk
Mpp3	Frk
Mpp5	Frk
Msn	Frk
Nckap1l	Frk
Pkp3	Frk
Plcg2	Frk
Ppm1l	Frk
Ptpn2	Frk
Ptprc	Frk
Rac2	Frk
Rad51	Frk
Rap1b	Frk
Rapsn	Frk
Rasa1	Frk
Sgk3	Frk
Skap2	Frk
Sla	Frk
Stat1	Frk
Tlr2	Frk
Trp63	Frk
Vcam1	Frk
Wasf2	Frk
Frk	Hck
Frk	Plaur
Frk	Ptpn6
Frk	Vav1
Grip1	Cdh4
Grip1	Grin1
Grip1	Mapre3
Grip1	Myod1
Grip1	Myog
Grip1	Ncoa3
Grip1	Racgap1
Grip1	Sdcbp
Hhatl	Casq1
Cav3	Hhatl

Dhrs7c	Hhatl
Kcna7	Hhatl
Obscn	Hhatl
Hhatl	Myom2
Hhatl	Trim54
Inpp5d	AF251705
Inpp5d	Abcg1
Inpp5d	Aif1
Inpp5d	Alox5ap
Inpp5d	Arhgap25
Inpp5d	Arhgap30
Inpp5d	Arhgdib
Inpp5d	Avpr2
Inpp5d	Btk
Inpp5d	Cd2ap
Inpp5d	Cd48
Inpp5d	Cd52
Inpp5d	Cd53
Inpp5d	Cd84
Inpp5d	Clec4a3
Inpp5d	Coro1a
Inpp5d	Cotl1
Inpp5d	Csf2ra
Inpp5d	Csf2rb2
Inpp5d	Ctss
Inpp5d	Emr1
Inpp5d	Evi2a
Inpp5d	Fcer1g
Inpp5d	Fcgr2b
Inpp5d	Fxyd5
Inpp5d	Fyb
Inpp5d	Gmfg
Inpp5d	Gpr65
Inpp5d	Havcr2
Inpp5d	Hck
Inpp5d	Igsf6
Fcgr3	Inpp5d
Inpp5d	Irf8
Inpp5d	Itgb2
Inpp5d	Klhl6
Inpp5d	Lcp2
Inpp5d	Lpxn
Inpp5d	Lrmp
Inpp5d	Ly86
Inpp5d	Lyn
Inpp5d	Mpeg1
Inpp5d	Myo1f
Inpp5d	Ncf4

Inpp5d	Nckap1l
Inpp5d	Pik3c2a
Inpp5d	Pik3r5
Inpp5d	Plcg2
Inpp5d	Pld4
Inpp5d	Plek
Inpp5d	Ppp2r3a
Inpp5d	Prkcb
Inpp5d	Ptpn22
Inpp5d	Ptpn6
Inpp5d	Ptprc
Inpp5d	Pycard
Inpp5d	Rac2
Inpp5d	Rasa1
Inpp5d	Sash3
Inpp5d	Slc11a1
Inpp5d	Snx20
Inpp5d	Themis2
Inpp5d	Tyrobp
Inpp5d	Vav1
Alpk3	Itgb1bp2
Cav3	Itgb1bp2
Dhrs7c	Itgb1bp2
Hrc	Itgb1bp2
Hspb3	Itgb1bp2
Lmod3	Itgb1bp2
Myom1	Itgb1bp2
Myom2	Itgb1bp2
Synpo2l	Itgb1bp2
Ttn	Itgb1bp2
Itgb1bp2	Ldb3
Itgb1bp2	Srl
Itgb1bp2	Trim54
Gsk3a	Krt14
Itga6	Krt14
Map3k8	Krt14
Pkp3	Krt14
Trp63	Krt14
Usp18	Krt14
Lats1	Herc6
Casp3	Lats1
Cdkn3	Lats1
Gm4980	Lats1
Lingo3	Lats1
Lrr1	Lats1
Lrrc15	Lats1
Lrrc30	Lats1
Lrrc38	Lats1

Lrrc4	Lats1
Lrrc8c	Lats1
Lrtm1	Lats1
Lrtm2	Lats1
Plk5	Lats1
Podnl1	Lats1
Lats1	Map3k8
Lats1	Plk1
Lats1	Plk2
Lats1	Plk4
Mapk12	Mylk4
Scn4b	Mylk4
Mylk4	Mylpf
Mylk4	Pvalb
Mylk4	Vcam1
Myo1f	Actr2
Myo1f	Actr3
Myo1f	Aif1
Myo1f	Alox5ap
Myo1f	Arhgap30
Myo1f	Arpc5
Myo1f	C1qb
Myo1f	Cd48
Myo1f	Cd52
Myo1f	Cd53
Myo1f	Cd84
Myo1f	Coro1a
Myo1f	Cotl1
Myo1f	Cybb
Myo1f	Emr1
Myo1f	Fcer1g
Myo1f	Fcgr1
Myo1f	Fcgr4
Myo1f	Glipr1
Myo1f	Gpr65
Myo1f	Hck
Myo1f	Hk3
Myo1f	Igsf6
Myo1f	Itgb2
Myo1f	Lair1
Myo1f	Lilrb4
Myo1f	Lpxn
Myo1f	Ly86
Myo1f	Ms4a6d
AF251705	Myo1f
Actc1	Myo1f
Arhgap25	Myo1f
Cd68	Myo1f

Clec4a3	Myo1f
Ctss	Myo1f
Evi2a	Myo1f
Fcgr3	Myo1f
Fxyd5	Myo1f
Inpp5d	Myo1f
Lst1	Myo1f
Mpeg1	Myo1f
Ncf1	Myo1f
Ncf4	Myo1f
Plek	Myo1f
Tpm2	Myo1f
Myo1f	Nckap1l
Myo1f	Pld4
Myo1f	Ptpn6
Myo1f	Ptprc
Myo1f	Pvalb
Myo1f	Rac2
Myo1f	Rictor
Myo1f	Sash3
Myo1f	Slc11a1
Myo1f	Slc15a3
Myo1f	Tbxas1
Myo1f	Themis2
Myo1f	Tyrobp
Myo1f	Unc45b
Myo1f	Vav1
Pvalb	Casq1
Actc1	Pvalb
Cacna1s	Pvalb
Cetn3	Pvalb
Ckmt2	Pvalb
Lmod3	Pvalb
Mylk4	Pvalb
Mylpf	Pvalb
Myo18b	Pvalb
Myo1f	Pvalb
Npy	Pvalb
Obscn	Pvalb
Palb2	Pvalb
Ryr1	Pvalb
Ttn	Pvalb
Rpl39l	Oasl1
Isg15	Rpl39l
Nap1l1	Rpl39l
Rpl39l	Rps3a
Rpl39l	Ubd
Slamf6	Ccr6

Slamf6	Cd3d
Slamf6	Cd3g
Slamf6	Cytip
Slamf6	Gpr18
Slamf6	Il7r
Slamf6	Klrd1
Slamf6	Ltb
Slamf6	Ms4a4c
Slamf6	Ptpn6
Slamf6	Ptprc
Slamf6	Rac2
Slamf6	Sash3
Slamf6	Sell
Slamf6	Txk
Itgam	Spic
Marco	Spic
Myc	Spic
Prf1	Spic
Ptprc	Spic
Slc11a1	Spic
Spp1	Avpr2
Spp1	Cav3
Spp1	Ccl2
Spp1	Ednrb
Spp1	Irf7
Spp1	Itga6
Spp1	Itgb2
Spp1	Racgap1
Spp1	Retnlg
Spp1	Runx3
Spp1	Slc11a1
B2m	Spp1
Cd68	Spp1
Itga7	Spp1
Spp1	Thbs1
Spp1	Timp1
Srsf11	Hnrnpa2b1
Srsf11	Prpf40a
Tpm2	Actc1
Tpm2	Actr3
Tpm2	Ckmt2
Tpm2	Myl3
Tpm2	Mylpf
Tpm2	Myo18b
Tpm2	Myo1f
Tpm2	Myom2
Tpm2	Obscn
Tpm2	Tmod1

Ttn	Tpm2
Tpm2	Vmp1

Table S5 Major chemical components of DJ and GC detected by Ultra-performance liquid chromatography tandem mass-spectrometry

Herbs	Chemical components	Molecular formulas	2D structural formulas
Glycyrrhiza	Liquiritin	$C_{21}H_{22}O_9$	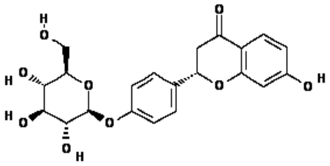
Glycyrrhiza	Isoliquiritin	$C_{21}H_{22}O_9$	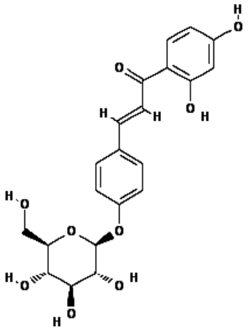

Glycyrrhiza	Liquiritigenin	$C_{15}H_{12}O_4$	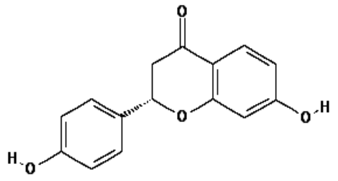
Glycyrrhiza	Isoliquiritigenin	$C_{15}H_{12}O_4$	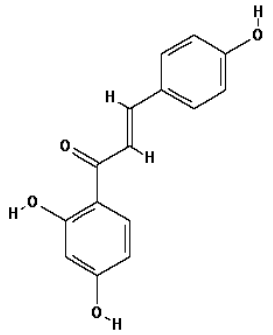

Glycyrrhiza	Glycyrrhizic acid	$C_{42}H_{62}O_{16}$	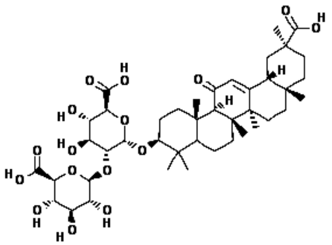
Euphorbia Pekinensis	Pekinenal	$C_{20}H_{30}O_2$	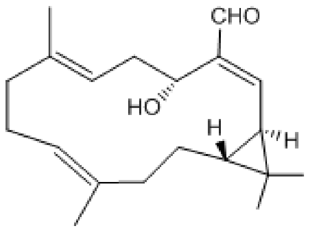
Euphorbia Pekinensis	Pekinenins C	$C_{20}H_{30}O_2$	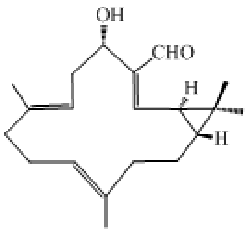

Euphorbia Pekinensis	Pekinenal D	$C_{20}H_{30}O_3$	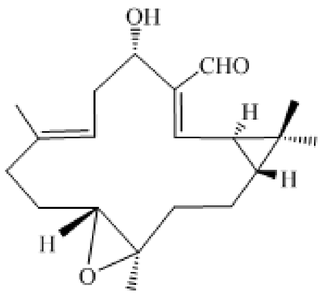
Euphorbia Pekinensis	3β,12α,13α)-3,12-dihydroxy-pimara-7,15-diene-2-one	$C_{20}H_{30}O_3$	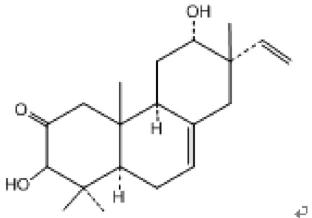

Table S6 Sequences of the primers using in this study

(GAPDH and Rpl13a were used as reference genes)

Gene Name	Primer name	Primer sequence
FRK	FRK-F	GAGAGGTGGCTGTTCTTTGTT
	FRK-R	TGGGCACCGAACAATAAGTAA
Arhgdib	Arhgdib -F	ATCGGAAGATAGGCAGAGCA
	Arhgdib -R	CCTTCTCCGTCATCTTGATTGC
Inpp5d	Inpp5d -F	TCCAAGAATGGTCCTGGCAC
	Inpp5d -R	TGGTCTTCAGTGTGGCGTAG
AVPR V2	AVPR V2-F	TCCTCCCTCTGTCTGTCTCC
	AVPR V2-R	CAGCTTCTGTGCCCACCATA
Aqp4	Aqp4 -F	TCAGCATCGCTAAGTCCGTC
	Aqp4 -R	CGTGGTGACTCCCAATCCTC
GAPDH	GAPDH-F	AAGGTCGGTGTGAACGGATT
	GAPDH-R	GTGAGTGGAGTCATACTGGAACAT
Rpl13a	Rpl13a-F	GACAGCCACTCTGGAGGAGAAA
	Rpl13a-R	TCTGCCTGTTTCCGTAACCTCAAGA
